# Supplementary material for: Centrosome guides spatial activation of Rac to control cell polarization and directed cell migration
Source: Life Sci Alliance. 2019 Feb 8;2(1):e201800135. doi: 10.26508/lsa.201800135 (PMC6369537; doi:10.26508/lsa.201800135)
Supplement: Supplementary file 5 [file LSA-2018-00135_TableS2.docx]

**Table S2. Proteins reproducibly identified from proteomic analysis of FAs in RPEp53^-/-^SAS6^-/-^ cells.**

| **Gene Name** | **Common Protein Name** |
| --- | --- |
| *A1BG* | Alpha-1B-glycoprotein |
| *A2M* | Alpha-2-macroglobulin |
| *ABCA13* | ATP-binding cassette sub-family A member 13 |
| *ABCA6* | ATP-binding cassette sub-family A member 6 |
| *ABCB4* | Phosphatidylcholine translocator ABCB4 |
| *ABCF1* | ATP-binding cassette sub-family F member 1 |
| *ACO1* | Cytoplasmic aconitate hydratase |
| *ACOX1* | Peroxisomal acyl-coenzyme A oxidase 1 |
| *ACSL4* | Long-chain-fatty-acid--CoA ligase 4 |
| *ACTBL2* | Beta-actin-like protein 2 |
| *ACTC1* | Actin, alpha cardiac muscle 1 |
| *ACTG1* | Actin, cytoplasmic 2 |
| *ACTN1* | Alpha-actinin-1 |
| *ACTN4* | Alpha-actinin-4 |
| *ACTR2* | Actin-related protein 2 |
| *ACTR3* | Actin-related protein 3 |
| *ADAM10* | Disintegrin and metalloproteinase domain-containing protein 10 |
| *ADAM19* | Disintegrin and metalloproteinase domain-containing protein 19 |
| *ADAM8* | Disintegrin and metalloproteinase domain-containing protein 8 |
| *ADAM9* | Disintegrin and metalloproteinase domain-containing protein 9 |
| *ADAMTS1* | A disintegrin and metalloproteinase with thrombospondin motifs 1 |
| *ADAMTS15* | A disintegrin and metalloproteinase with thrombospondin motifs 15 |
| *ADAMTS5* | A disintegrin and metalloproteinase with thrombospondin motifs 5 |
| *ADAMTSL1* | ADAMTS-like protein 1 |
| *ADD1* | Alpha-adducin |
| *ADD3* | Gamma-adducin |
| *ADGRL1* | Adhesion G protein-coupled receptor L1 |
| *ADM* | ADM |
| *AFMID* | Kynurenine formamidase |
| *AFP* | Alpha-fetoprotein |
| *AGBL5* | Cytosolic carboxypeptidase-like protein 5 |
| *AGFG1* | Arf-GAP domain and FG repeat-containing protein 1 |
| *AGPAT1* | 1-acyl-sn-glycerol-3-phosphate acyltransferase alpha |
| *AGRN* | Agrin |
| *AHNAK* | Neuroblast differentiation-associated protein AHNAK |
| *AHNAK2* | Protein AHNAK2 |
| *AHSG* | Alpha-2-HS-glycoprotein |
| *AKAP12* | A-kinase anchor protein 12 |
| *AKAP13* | A-kinase anchor protein 13 |
| *AKAP17A* | A-kinase anchor protein 17A |
| *AKAP2* | A-kinase anchor protein 2 |
| *ALB* | Serum albumin |
| *ALCAM* | CD166 antigen |
| *ALDH18A1* | Delta-1-pyrroline-5-carboxylate synthase |
| *ALDOA* | Fructose-bisphosphate aldolase A |
| *AMMECR1L* | AMMECR1-like protein |
| *ANGPT1* | Angiopoietin-1 |
| *ANK3* | Ankyrin-3 |
| *ANKRD18A* | Ankyrin repeat domain-containing protein 18A |
| *ANKRD50* | Ankyrin repeat domain-containing protein 50 |
| *ANO2* | Anoctamin-2 |
| *ANOS1* | Anosmin-1 |
| *ANPEP* | Aminopeptidase N |
| *ANXA1* | Annexin A1 |
| *ANXA2* | Annexin A2 |
| *ANXA4* | Annexin A4 |
| *ANXA5* | Annexin A5 |
| *ANXA6* | Annexin A6 |
| *AP2A1* | AP-2 complex subunit alpha-1 |
| *AP2A2* | AP-2 complex subunit alpha-2 |
| *AP2B1* | AP-2 complex subunit beta |
| *AP2M1* | AP-2 complex subunit mu |
| *AP2S1* | AP-2 complex subunit sigma |
| *AP3B1* | AP-3 complex subunit beta-1 |
| *AP5M1* | AP-5 complex subunit mu-1 |
| *APC2* | Adenomatous polyposis coli protein 2 |
| *APOA1* | Apolipoprotein A-I |
| *APOA4* | Apolipoprotein A-IV |
| *APOB* | Apolipoprotein B-100 |
| *APOC3* | Apolipoprotein C-III |
| *APOE* | Apolipoprotein E |
| *APOH* | Beta-2-glycoprotein 1 |
| *APOM* | Apolipoprotein M |
| *APP* | Amyloid beta A4 protein |
| *ARF4* | ADP-ribosylation factor 4 |
| *ARFIP1* | Arfaptin-1 |
| *ARG1* | Arginase-1 |
| *ARHGAP22* | Rho GTPase-activating protein 22 |
| *ARHGDIA* | Rho GDP-dissociation inhibitor 1 |
| *ARHGEF2* | Rho guanine nucleotide exchange factor 2 |
| *ARHGEF6* | Rho guanine nucleotide exchange factor 6 |
| *ARHGEF7* | Rho guanine nucleotide exchange factor 7 |
| *ARL6IP1* | ADP-ribosylation factor-like protein 6-interacting protein 1 |
| *ARL6IP5* | PRA1 family protein 3 |
| *ARPC1A* | Actin-related protein 2/3 complex subunit 1A |
| *ARPC1B* | Actin-related protein 2/3 complex subunit 1B |
| *ARPC2* | Actin-related protein 2/3 complex subunit 2 |
| *ARPC3* | Actin-related protein 2/3 complex subunit 3 |
| *ARPC4* | Actin-related protein 2/3 complex subunit 4 |
| *ARPC5* | Actin-related protein 2/3 complex subunit 5 |
| *ARPC5L* | Actin-related protein 2/3 complex subunit 5-like protein |
| *ARSJ* | Arylsulfatase J |
| *ASAP2* | Arf-GAP with SH3 domain, ANK repeat and PH domain-containing protein 2 |
| *ASPH* | Aspartyl/asparaginyl beta-hydroxylase |
| *ATIC* | Bifunctional purine biosynthesis protein PURH |
| *ATL3* | Atlastin-3 |
| *ATP1A1* | Sodium/potassium-transporting ATPase subunit alpha-1 |
| *ATP1A2* | Sodium/potassium-transporting ATPase subunit alpha-2 |
| *ATP1A3* | Sodium/potassium-transporting ATPase subunit alpha-3 |
| *ATP1B1* | Sodium/potassium-transporting ATPase subunit beta-1 |
| *ATP1B3* | Sodium/potassium-transporting ATPase subunit beta-3 |
| *ATP2A1* | Sarcoplasmic/endoplasmic reticulum calcium ATPase 1 |
| *ATP2B1* | Plasma membrane calcium-transporting ATPase 1 |
| *ATP2B2* | Plasma membrane calcium-transporting ATPase 2 |
| *ATP2B4* | Plasma membrane calcium-transporting ATPase 4 |
| *ATP4A* | Potassium-transporting ATPase alpha chain 1 |
| *ATP5A1* | ATP synthase subunit alpha, mitochondrial |
| *ATP5B* | ATP synthase subunit beta, mitochondrial |
| *ATP5E* | ATP synthase subunit epsilon, mitochondrial |
| *ATP5H* | ATP synthase subunit d, mitochondrial |
| *ATP6V0A4* | V-type proton ATPase 116 kDa subunit a isoform 4 |
| *ATP6V1D* | V-type proton ATPase subunit D |
| *ATP7A* | Copper-transporting ATPase 1 |
| *ATRX* | Transcriptional regulator ATRX |
| *AXL* | Tyrosine-protein kinase receptor UFO |
| *AZGP1* | Zinc-alpha-2-glycoprotein |
| *B2M* | Beta-2-microglobulin |
| *BAIAP2* | Brain-specific angiogenesis inhibitor 1-associated protein 2 |
| *BASP1* | Brain acid soluble protein 1 |
| *BAZ1A* | Bromodomain adjacent to zinc finger domain protein 1A |
| *BAZ2B* | Bromodomain adjacent to zinc finger domain protein 2B |
| *BCAP31* | B-cell receptor-associated protein 31 |
| *BCL10* | B-cell lymphoma/leukemia 10 |
| *BDNF* | Brain-derived neurotrophic factor |
| *BDP1* | Transcription factor TFIIIB component B'' homolog |
| *BEND7* | BEN domain-containing protein 7 |
| *BHMG1* | Basic helix-loop-helix and HMG box domain-containing protein 1 |
| *BLK* | Tyrosine-protein kinase Blk |
| *BLVRB* | Flavin reductase (NADPH) |
| *BMP1* | Bone morphogenetic protein 1 |
| *BRCA1* | Breast cancer type 1 susceptibility protein |
| *BRD9* | Bromodomain-containing protein 9 |
| *BRWD3* | Bromodomain and WD repeat-containing protein 3 |
| *BSG* | Basigin |
| *BTBD10* | BTB/POZ domain-containing protein 10 |
| *BTF3* | Transcription factor BTF3 |
| *C1QBP* | Complement component 1 Q subcomponent-binding protein, mitochondrial |
| *C1QTNF3* | Complement C1q tumor necrosis factor-related protein 3 |
| *C20orf27* | UPF0687 protein C20orf27 |
| *C2orf42* | Uncharacterized protein C2orf42 |
| *C3* | Complement C3 |
| *C3orf58* | Deleted in autism protein 1 |
| *C4A* | Complement C4-A |
| *C6orf10* | Uncharacterized protein C6orf10 |
| *C9* | Complement component C9 |
| *CA9* | Carbonic anhydrase 9 |
| *CACNA2D1* | Voltage-dependent calcium channel subunit alpha-2/delta-1 |
| *CACTIN-AS1* | Putative uncharacterized protein encoded by CACTIN-AS1 |
| *CALD1* | Caldesmon |
| *CALM1* | Calmodulin |
| *CALML5* | Calmodulin-like protein 5 |
| *CALR* | Calreticulin |
| *CALU* | Calumenin |
| *CAMK1D* | Calcium/calmodulin-dependent protein kinase type 1D |
| *CAMK4* | Calcium/calmodulin-dependent protein kinase type IV |
| *CAMTA1* | Calmodulin-binding transcription activator 1 |
| *CANX* | Calnexin |
| *CAP1* | Adenylyl cyclase-associated protein 1 |
| *CAPN1* | Calpain-1 catalytic subunit |
| *CAPNS1* | Calpain small subunit 1 |
| *CAPRIN1* | Caprin-1 |
| *CAPZA1* | F-actin-capping protein subunit alpha-1 |
| *CAPZB* | F-actin-capping protein subunit beta |
| *CASK* | Peripheral plasma membrane protein CASK |
| *CASP12* | Inactive caspase-12 |
| *CASP14* | Caspase-14 |
| *CAT* | Catalase |
| *CAV1* | Caveolin-1 |
| *CAV2* | Caveolin-2 |
| *CBR1* | Carbonyl reductase [NADPH] 1 |
| *CC2D1A* | Coiled-coil and C2 domain-containing protein 1A |
| *CCDC124* | Coiled-coil domain-containing protein 124 |
| *CCDC14* | Coiled-coil domain-containing protein 14 |
| *CCDC158* | Coiled-coil domain-containing protein 158 |
| *CCDC47* | Coiled-coil domain-containing protein 47 |
| *CCDC50* | Coiled-coil domain-containing protein 50 |
| *CCDC62* | Coiled-coil domain-containing protein 62 |
| *CCDC80* | Coiled-coil domain-containing protein 80 |
| *CCDC96* | Coiled-coil domain-containing protein 96 |
| *CCIN* | Calicin |
| *CCL18* | C-C motif chemokine 18 |
| *CCL26* | C-C motif chemokine 26 |
| *CCNY* | Cyclin-Y |
| *CD101* | Immunoglobulin superfamily member 2 |
| *CD109* | CD109 antigen |
| *CD151* | CD151 antigen |
| *CD247* | T-cell surface glycoprotein CD3 zeta chain |
| *CD276* | CD276 antigen |
| *CD44* | CD44 antigen |
| *CD55* | Complement decay-accelerating factor |
| *CD58* | Lymphocyte function-associated antigen 3 |
| *CD59* | CD59 glycoprotein |
| *CD81* | CD81 antigen |
| *CD82* | CD82 antigen |
| *CD9* | CD9 antigen |
| *CD97* | CD97 antigen |
| *CD99* | CD99 antigen |
| *CD99L2* | CD99 antigen-like protein 2 |
| *CDC42* | Cell division control protein 42 homolog |
| *CDC42EP1* | Cdc42 effector protein 1 |
| *CDC42EP3* | Cdc42 effector protein 3 |
| *CDC42EP4* | Cdc42 effector protein 4 |
| *CDH13* | Cadherin-13 |
| *CDH2* | Cadherin-2 |
| *CDH23* | Cadherin-23 |
| *CDH6* | Cadherin-6 |
| *CDK8* | Cyclin-dependent kinase 8 |
| *CDT1* | DNA replication factor Cdt1 |
| *CELSR1* | Cadherin EGF LAG seven-pass G-type receptor 1 |
| *CEP131* | Centrosomal protein of 131 kDa |
| *CEP192* | Centrosomal protein of 192 kDa |
| *CEP295NL* | CEP295 N-terminal-like protein |
| *CEP89* | Centrosomal protein of 89 kDa |
| *CETN3* | Centrin-3 |
| *CFAP43* | Cilia- and flagella-associated protein 43 |
| *CFH* | Complement factor H |
| *CFL1* | Cofilin-1 |
| *CFL2* | Cofilin-2 |
| *CHD7* | Chromodomain-helicase-DNA-binding protein 7 |
| *CHMP4B* | Charged multivesicular body protein 4b |
| *CHMP6* | Charged multivesicular body protein 6 |
| *CHRDL1* | Chordin-like protein 1 |
| *CHST2* | Carbohydrate sulfotransferase 2 |
| *CIRBP* | Cold-inducible RNA-binding protein |
| *CKAP5* | Cytoskeleton-associated protein 5 |
| *CLASP1* | CLIP-associating protein 1 |
| *CLDND1* | Claudin domain-containing protein 1 |
| *CLEC3B* | Tetranectin |
| *CLIC1* | Chloride intracellular channel protein 1 |
| *CLIC4* | Chloride intracellular channel protein 4 |
| *CLIP1* | CAP-Gly domain-containing linker protein 1 |
| *CLMP* | CXADR-like membrane protein |
| *CLTA* | Clathrin light chain A |
| *CLTB* | Clathrin light chain B |
| *CLTC* | Clathrin heavy chain 1 |
| *CMYA5* | Cardiomyopathy-associated protein 5 |
| *CNGB1* | Cyclic nucleotide-gated cation channel beta-1 |
| *CNN2* | Calponin-2 |
| *CNN3* | Calponin-3 |
| *CNP* | 2',3'-cyclic-nucleotide 3'-phosphodiesterase |
| *CNTN3* | Contactin-3 |
| *COBL* | Protein cordon-bleu |
| *COL12A1* | Collagen alpha-1(XII) chain |
| *COL17A1* | Collagen alpha-1(XVII) chain |
| *COL1A1* | Collagen alpha-1(I) chain |
| *COL4A1* | Collagen alpha-1(IV) chain |
| *COL4A2* | Collagen alpha-2(IV) chain |
| *COL4A5* | Collagen alpha-5(IV) chain |
| *COL6A1* | Collagen alpha-1(VI) chain |
| *COL6A2* | Collagen alpha-2(VI) chain |
| *COL7A1* | Collagen alpha-1(VII) chain |
| *COL8A1* | Collagen alpha-1(VIII) chain |
| *COQ6* | Ubiquinone biosynthesis monooxygenase COQ6, mitochondrial |
| *CORO1B* | Coronin-1B |
| *CORO1C* | Coronin-1C |
| *COTL1* | Coactosin-like protein |
| *CPLX1* | Complexin-1 |
| *CPNE2* | Copine-2 |
| *CPNE3* | Copine-3 |
| *CPSF1* | Cleavage and polyadenylation specificity factor subunit 1 |
| *CPZ* | Carboxypeptidase Z |
| *CR1* | Complement receptor type 1 |
| *CRACR2A* | EF-hand calcium-binding domain-containing protein 4B |
| *CREB5* | Cyclic AMP-responsive element-binding protein 5 |
| *CRK* | Adapter molecule crk |
| *CRKL* | Crk-like protein |
| *CRLF3* | Cytokine receptor-like factor 3 |
| *CROCC* | Rootletin |
| *CRYBG3* | Very large A-kinase anchor protein |
| *CSF3R* | Granulocyte colony-stimulating factor receptor |
| *CSK* | Tyrosine-protein kinase CSK |
| *CSPG4* | Chondroitin sulfate proteoglycan 4 |
| *CSRP1* | Cysteine and glycine-rich protein 1 |
| *CSRP2* | Cysteine and glycine-rich protein 2 |
| *CSTA* | Cystatin-A |
| *CTAGE1* | cTAGE family member 2 |
| *CTGF* | Connective tissue growth factor |
| *CTNNA1* | Catenin alpha-1 |
| *CTNNB1* | Catenin beta-1 |
| *CTNND1* | Catenin delta-1 |
| *CTTN* | Src substrate cortactin |
| *CTTNBP2* | Cortactin-binding protein 2 |
| *CTTNBP2NL* | CTTNBP2 N-terminal-like protein |
| *CUTA* | Protein CutA |
| *CWC22* | Pre-mRNA-splicing factor CWC22 homolog |
| *CYFIP1* | Cytoplasmic FMR1-interacting protein 1 |
| *CYR61* | Protein CYR61 |
| *DAB2* | Disabled homolog 2 |
| *DAG1* | Dystroglycan |
| *DBN1* | Drebrin |
| *DBNL* | Drebrin-like protein |
| *DCAF12L2* | DDB1- and CUL4-associated factor 12-like protein 2 |
| *DCBLD2* | Discoidin, CUB and LCCL domain-containing protein 2 |
| *DCD* | Dermcidin |
| *DCST2* | DC-STAMP domain-containing protein 2 |
| *DDR2* | Discoidin domain-containing receptor 2 |
| *DDX46* | Probable ATP-dependent RNA helicase DDX46 |
| *DENND6A* | Protein DENND6A |
| *DERL2* | Derlin-2 |
| *DES* | Desmin |
| *DHRS7C* | Dehydrogenase/reductase SDR family member 7C |
| *DHX33* | Putative ATP-dependent RNA helicase DHX33 |
| *DIAPH1* | Protein diaphanous homolog 1 |
| *DKK1* | Dickkopf-related protein 1 |
| *DLC1* | Rho GTPase-activating protein 7 |
| *DLG1* | Disks large homolog 1 |
| *DNAH2* | Dynein heavy chain 2, axonemal |
| *DNAH5* | Dynein heavy chain 5, axonemal |
| *DNAH8* | Dynein heavy chain 8, axonemal |
| *DNAH9* | Dynein heavy chain 9, axonemal |
| *DNAI1* | Dynein intermediate chain 1, axonemal |
| *DNAJA2* | DnaJ homolog subfamily A member 2 |
| *DNER* | Delta and Notch-like epidermal growth factor-related receptor |
| *DNHD1* | Dynein heavy chain domain-containing protein 1 |
| *DNM2* | Dynamin-2 |
| *DNMT1* | DNA (cytosine-5)-methyltransferase 1 |
| *DOCK6* | Dedicator of cytokinesis protein 6 |
| *DOCK7* | Dedicator of cytokinesis protein 7 |
| *DOPEY1* | Protein dopey-1 |
| *DPYSL2* | Dihydropyrimidinase-related protein 2 |
| *DRG1* | Developmentally-regulated GTP-binding protein 1 |
| *DSC1* | Desmocollin-1 |
| *DSC2* | Desmocollin-2 |
| *DSC3* | Desmocollin-3 |
| *DSG1* | Desmoglein-1 |
| *DSG2* | Desmoglein-2 |
| *DSP* | Desmoplakin |
| *DSTN* | Destrin |
| *DUOX2* | Dual oxidase 2 |
| *DYNC1H1* | Cytoplasmic dynein 1 heavy chain 1 |
| *DYNLL1* | Dynein light chain 1, cytoplasmic |
| *DYSF* | Dysferlin |
| *E2F7* | Transcription factor E2F7 |
| *ECE1* | Endothelin-converting enzyme 1 |
| *EDEM3* | ER degradation-enhancing alpha-mannosidase-like protein 3 |
| *EDIL3* | EGF-like repeat and discoidin I-like domain-containing protein 3 |
| *EEF1A1* | Elongation factor 1-alpha 1 |
| *EEF1B2* | Elongation factor 1-beta |
| *EEF1D* | Elongation factor 1-delta |
| *EEF1G* | Elongation factor 1-gamma |
| *EEF2* | Elongation factor 2 |
| *EFHD1* | EF-hand domain-containing protein D1 |
| *EFHD2* | EF-hand domain-containing protein D2 |
| *EGFR* | Epidermal growth factor receptor |
| *EHBP1L1* | EH domain-binding protein 1-like protein 1 |
| *EHD1* | EH domain-containing protein 1 |
| *EHD2* | EH domain-containing protein 2 |
| *EHD3* | EH domain-containing protein 3 |
| *EHD4* | EH domain-containing protein 4 |
| *EIF1AX* | Eukaryotic translation initiation factor 1A, X-chromosomal |
| *EIF2S2* | Eukaryotic translation initiation factor 2 subunit 2 |
| *EIF3CL* | Eukaryotic translation initiation factor 3 subunit C-like protein |
| *EIF3G* | Eukaryotic translation initiation factor 3 subunit G |
| *EIF4A1* | Eukaryotic initiation factor 4A-I |
| *EIF4A3* | Eukaryotic initiation factor 4A-III |
| *EIF4G1* | Eukaryotic translation initiation factor 4 gamma 1 |
| *EIF4H* | Eukaryotic translation initiation factor 4H |
| *ELL2* | RNA polymerase II elongation factor ELL2 |
| *EMB* | Embigin |
| *EML6* | Echinoderm microtubule-associated protein-like 6 |
| *ENAH* | Protein enabled homolog |
| *ENDOD1* | Endonuclease domain-containing 1 protein |
| *ENO1* | Alpha-enolase |
| *EPB41* | Protein 4.1 |
| *EPB41L2* | Band 4.1-like protein 2 |
| *EPB41L3* | Band 4.1-like protein 3 |
| *EPHA2* | Ephrin type-A receptor 2 |
| *EPHA3* | Ephrin type-A receptor 3 |
| *EPHA4* | Ephrin type-A receptor 4 |
| *EPHA6* | Ephrin type-A receptor 6 |
| *EPHB2* | Ephrin type-B receptor 2 |
| *EPN1* | Epsin-1 |
| *EPS15L1* | Epidermal growth factor receptor substrate 15-like 1 |
| *EPS8* | Epidermal growth factor receptor kinase substrate 8 |
| *ERBB2* | Receptor tyrosine-protein kinase erbB-2 |
| *ERBIN* | Erbin |
| *ERLIN2* | Erlin-2 |
| *ERP29* | Endoplasmic reticulum resident protein 29 |
| *ESCO1* | N-acetyltransferase ESCO1 |
| *ESM1* | Endothelial cell-specific molecule 1 |
| *ESR2* | Estrogen receptor beta |
| *ESYT1* | Extended synaptotagmin-1 |
| *ESYT2* | Extended synaptotagmin-2 |
| *ETF1* | Eukaryotic peptide chain release factor subunit 1 |
| *EVA1A* | Protein eva-1 homolog A |
| *EVA1B* | Protein eva-1 homolog B |
| *EXOC8* | Exocyst complex component 8 |
| *EXTL1* | Exostosin-like 1 |
| *EZR* | Ezrin |
| *F10* | Coagulation factor X |
| *F11R* | Junctional adhesion molecule A |
| *F13A1* | Coagulation factor XIII A chain |
| *F13B* | Coagulation factor XIII B chain |
| *F2* | Prothrombin |
| *F3* | Tissue factor |
| *F5* | Coagulation factor V |
| *F8* | Coagulation factor VIII |
| *FABP5* | Fatty acid-binding protein, epidermal |
| *FAM117A* | Protein FAM117A |
| *FAM120C* | Constitutive coactivator of PPAR-gamma-like protein 2 |
| *FAM129B* | Niban-like protein 1 |
| *FAM160A1* | Protein FAM160A1 |
| *FAM160B2* | Protein FAM160B2 |
| *FAM186A* | Protein FAM186A |
| *FAM49B* | Protein FAM49B |
| *FANK1* | Fibronectin type 3 and ankyrin repeat domains protein 1 |
| *FAS* | Tumor necrosis factor receptor superfamily member 6 |
| *FAT2* | Protocadherin Fat 2 |
| *FBLIM1* | Filamin-binding LIM protein 1 |
| *FBLN1* | Fibulin-1 |
| *FBN1* | Fibrillin-1 |
| *FBN2* | Fibrillin-2 |
| *FCHO1* | F-BAR domain only protein 1 |
| *FCHSD2* | F-BAR and double SH3 domains protein 2 |
| *FERMT2* | Fermitin family homolog 2 |
| *FERMT3* | Fermitin family homolog 3 |
| *FGD4* | FYVE, RhoGEF and PH domain-containing protein 4 |
| *FGFRL1* | Fibroblast growth factor receptor-like 1 |
| *FGG* | Fibrinogen gamma chain |
| *FH* | Fumarate hydratase, mitochondrial |
| *FHL1* | Four and a half LIM domains protein 1 |
| *FHL2* | Four and a half LIM domains protein 2 |
| *FHL3* | Four and a half LIM domains protein 3 |
| *FHOD1* | FH1/FH2 domain-containing protein 1 |
| *FKBP1A* | Peptidyl-prolyl cis-trans isomerase FKBP1A |
| *FKBP3* | Peptidyl-prolyl cis-trans isomerase FKBP3 |
| *FLG2* | Filaggrin-2 |
| *FLII* | Protein flightless-1 homolog |
| *FLNA* | Filamin-A |
| *FLNB* | Filamin-B |
| *FLNC* | Filamin-C |
| *FLOT1* | Flotillin-1 |
| *FMN2* | Formin-2 |
| *FMNL3* | Formin-like protein 3 |
| *FN1* | Fibronectin |
| *FNDC1* | Fibronectin type III domain-containing protein 1 |
| *FSCN1* | Fascin |
| *FSIP2* | Fibrous sheath-interacting protein 2 |
| *FST* | Follistatin |
| *FXR1* | Fragile X mental retardation syndrome-related protein 1 |
| *FZD7* | Frizzled-7 |
| *G3BP1* | Ras GTPase-activating protein-binding protein 1 |
| *GALNT12* | Polypeptide N-acetylgalactosaminyltransferase 12 |
| *GANAB* | Neutral alpha-glucosidase AB |
| *GAPDH* | Glyceraldehyde-3-phosphate dehydrogenase |
| *GC* | Vitamin D-binding protein |
| *GDF5* | Growth/differentiation factor 5 |
| *GDF6* | Growth/differentiation factor 6 |
| *GDI2* | Rab GDP dissociation inhibitor beta |
| *GGCT* | Gamma-glutamylcyclotransferase |
| *GGT2* | Inactive gamma-glutamyltranspeptidase 2 |
| *GIT1* | ARF GTPase-activating protein GIT1 |
| *GLIPR1* | Glioma pathogenesis-related protein 1 |
| *GLIPR2* | Golgi-associated plant pathogenesis-related protein 1 |
| *GLS* | Glutaminase kidney isoform, mitochondrial |
| *GLYR1* | Putative oxidoreductase GLYR1 |
| *GMFB* | Glia maturation factor beta |
| *GNA11* | Guanine nucleotide-binding protein subunit alpha-11 |
| *GNA12* | Guanine nucleotide-binding protein subunit alpha-12 |
| *GNA13* | Guanine nucleotide-binding protein subunit alpha-13 |
| *GNAI2* | Guanine nucleotide-binding protein G(i) subunit alpha-2 |
| *GNAI3* | Guanine nucleotide-binding protein G(k) subunit alpha |
| *GNAO1* | Guanine nucleotide-binding protein G(o) subunit alpha |
| *GNAQ* | Guanine nucleotide-binding protein G(q) subunit alpha |
| *GNAS* | Guanine nucleotide-binding protein G(s) subunit alpha isoforms XLas |
| *GNB1* | Guanine nucleotide-binding protein G(I)/G(S)/G(T) subunit beta-1 |
| *GNB2* | Guanine nucleotide-binding protein G(I)/G(S)/G(T) subunit beta-2 |
| *GNB4* | Guanine nucleotide-binding protein subunit beta-4 |
| *GNG12* | Guanine nucleotide-binding protein G(I)/G(S)/G(O) subunit gamma-12 |
| *GNLY* | Granulysin |
| *GOLGA4* | Golgin subfamily A member 4 |
| *GOLGA7* | Golgin subfamily A member 7 |
| *GPC1* | Glypican-1 |
| *GPD2* | Glycerol-3-phosphate dehydrogenase, mitochondrial |
| *GPI* | Glucose-6-phosphate isomerase |
| *GPR176* | Probable G-protein coupled receptor 176 |
| *GPRC5A* | Retinoic acid-induced protein 3 |
| *GPRIN1* | G protein-regulated inducer of neurite outgrowth 1 |
| *GRB2* | Growth factor receptor-bound protein 2 |
| *GREM1* | Gremlin-1 |
| *GRIN2D* | Glutamate receptor ionotropic, NMDA 2D |
| *GSN* | Gelsolin |
| *GSTA3* | Glutathione S-transferase A3 |
| *GSTP1* | Glutathione S-transferase P |
| *GTF2E1* | General transcription factor IIE subunit 1 |
| *GTF2IRD1* | General transcription factor II-I repeat domain-containing protein 1 |
| *GTF3C1* | General transcription factor 3C polypeptide 1 |
| *GULP1* | PTB domain-containing engulfment adapter protein 1 |
| *H1FX* | Histone H1x |
| *HABP2* | Hyaluronan-binding protein 2 |
| *HAL* | Histidine ammonia-lyase |
| *HBA1* | Hemoglobin subunit alpha |
| *HBB* | Hemoglobin subunit beta |
| *HBE1* | Hemoglobin subunit epsilon |
| *HBEGF* | Proheparin-binding EGF-like growth factor |
| *HDAC7* | Histone deacetylase 7 |
| *HERC3* | Probable E3 ubiquitin-protein ligase HERC3 |
| *HERC5* | E3 ISG15--protein ligase HERC5 |
| *HHIP* | Hedgehog-interacting protein |
| *HHLA1* | HERV-H LTR-associating protein 1 |
| *HIP1R* | Huntingtin-interacting protein 1-related protein |
| *HIST1H1B* | Histone H1.5 |
| *HIST1H1C* | Histone H1.2 |
| *HIST1H2AJ* | Histone H2A type 1-J |
| *HIST1H2BA* | Histone H2B type 1-A |
| *HIST1H4A* | Histone H4 |
| *HIST2H2BF* | Histone H2B type 2-F |
| *HK1* | Hexokinase-1 |
| *HLA-B* | HLA class I histocompatibility antigen, B-48 alpha chain |
| *HLA-C* | HLA class I histocompatibility antigen, Cw-17 alpha chain |
| *HLA-DRB1* | HLA class II histocompatibility antigen, DRB1-12 beta chain |
| *HMGA1* | High mobility group protein HMG-I/HMG-Y |
| *HMGB1* | High mobility group protein B1 |
| *HMGB2* | High mobility group protein B2 |
| *HPCA* | Neuron-specific calcium-binding protein hippocalcin |
| *HPCAL1* | Hippocalcin-like protein 1 |
| *HRG* | Histidine-rich glycoprotein |
| *HRNR* | Hornerin |
| *HSP90AA1* | Heat shock protein HSP 90-alpha |
| *HSP90AA2P* | Heat shock protein HSP 90-alpha A2 |
| *HSP90AB1* | Heat shock protein HSP 90-beta |
| *HSP90AB2P* | Putative heat shock protein HSP 90-beta 2 |
| *HSP90AB3P* | Putative heat shock protein HSP 90-beta-3 |
| *HSP90AB4P* | Putative heat shock protein HSP 90-beta 4 |
| *HSP90B1* | Endoplasmin |
| *HSP90B2P* | Putative endoplasmin-like protein |
| *HSPA1L* | Heat shock 70 kDa protein 1-like |
| *HSPA2* | Heat shock-related 70 kDa protein 2 |
| *HSPA5* | 78 kDa glucose-regulated protein |
| *HSPA6* | Heat shock 70 kDa protein 6 |
| *HSPA8* | Heat shock cognate 71 kDa protein |
| *HSPA9* | Stress-70 protein, mitochondrial |
| *HSPB1* | Heat shock protein beta-1 |
| *HSPD1* | 60 kDa heat shock protein, mitochondrial |
| *HSPE1* | 10 kDa heat shock protein, mitochondrial |
| *HTRA1* | Serine protease HTRA1 |
| *ICAM3* | Intercellular adhesion molecule 3 |
| *IGF2* | Insulin-like growth factor II |
| *IGF2R* | Cation-independent mannose-6-phosphate receptor |
| *IGFBP2* | Insulin-like growth factor-binding protein 2 |
| *IGFBP3* | Insulin-like growth factor-binding protein 3 |
| *IGFBP4* | Insulin-like growth factor-binding protein 4 |
| *IGFBP5* | Insulin-like growth factor-binding protein 5 |
| *IGFBP7* | Insulin-like growth factor-binding protein 7 |
| *IGFN1* | Immunoglobulin-like and fibronectin type III domain-containing protein 1 |
| *IGSF8* | Immunoglobulin superfamily member 8 |
| *ILK* | Integrin-linked protein kinase |
| *IMPDH2* | Inosine-5'-monophosphate dehydrogenase 2 |
| *INA* | Alpha-internexin |
| *ING2* | Inhibitor of growth protein 2 |
| *IQGAP1* | Ras GTPase-activating-like protein IQGAP1 |
| *IQGAP2* | Ras GTPase-activating-like protein IQGAP2 |
| *ISOC1* | Isochorismatase domain-containing protein 1 |
| *ITGA2* | Integrin alpha-2 |
| *ITGA2B* | Integrin alpha-IIb |
| *ITGA3* | Integrin alpha-3 |
| *ITGA5* | Integrin alpha-5 |
| *ITGAV* | Integrin alpha-V |
| *ITGB1* | Integrin beta-1 |
| *ITGB2* | Integrin beta-2 |
| *ITGB5* | Integrin beta-5 |
| *ITIH2* | Inter-alpha-trypsin inhibitor heavy chain H2 |
| *ITIH3* | Inter-alpha-trypsin inhibitor heavy chain H3 |
| *ITIH4* | Inter-alpha-trypsin inhibitor heavy chain H4 |
| *ITSN1* | Intersectin-1 |
| *ITSN2* | Intersectin-2 |
| *JUP* | Junction plakoglobin |
| *KANK2* | KN motif and ankyrin repeat domain-containing protein 2 |
| *KAT6A* | Histone acetyltransferase KAT6A |
| *KAT6B* | Histone acetyltransferase KAT6B |
| *KATNAL2* | Katanin p60 ATPase-containing subunit A-like 2 |
| *KCNK10* | Potassium channel subfamily K member 10 |
| *KIAA0556* | Protein KIAA0556 |
| *KIAA1462* | Junctional protein associated with coronary artery disease |
| *KIF17* | Kinesin-like protein KIF17 |
| *KIF18A* | Kinesin-like protein KIF18A |
| *KIF20B* | Kinesin-like protein KIF20B |
| *KIF26B* | Kinesin-like protein KIF26B |
| *KIF5A* | Kinesin heavy chain isoform 5A |
| *KIF5B* | Kinesin-1 heavy chain |
| *KLHL17* | Kelch-like protein 17 |
| *KPNA2* | Importin subunit alpha-1 |
| *KRT1* | Keratin, type II cytoskeletal 1 |
| *KRT10* | Keratin, type I cytoskeletal 10 |
| *KRT14* | Keratin, type I cytoskeletal 14 |
| *KRT15* | Keratin, type I cytoskeletal 15 |
| *KRT16* | Keratin, type I cytoskeletal 16 |
| *KRT17* | Keratin, type I cytoskeletal 17 |
| *KRT18* | Keratin, type I cytoskeletal 18 |
| *KRT2* | Keratin, type II cytoskeletal 2 epidermal |
| *KRT25* | Keratin, type I cytoskeletal 25 |
| *KRT3* | Keratin, type II cytoskeletal 3 |
| *KRT31* | Keratin, type I cuticular Ha1 |
| *KRT4* | Keratin, type II cytoskeletal 4 |
| *KRT5* | Keratin, type II cytoskeletal 5 |
| *KRT6A* | Keratin, type II cytoskeletal 6A |
| *KRT6B* | Keratin, type II cytoskeletal 6B |
| *KRT71* | Keratin, type II cytoskeletal 71 |
| *KRT75* | Keratin, type II cytoskeletal 75 |
| *KRT78* | Keratin, type II cytoskeletal 78 |
| *KRT8* | Keratin, type II cytoskeletal 8 |
| *KRT80* | Keratin, type II cytoskeletal 80 |
| *KRT85* | Keratin, type II cuticular Hb5 |
| *KRT9* | Keratin, type I cytoskeletal 9 |
| *KSR2* | Kinase suppressor of Ras 2 |
| *LAMA1* | Laminin subunit alpha-1 |
| *LAMB1* | Laminin subunit beta-1 |
| *LAMC1* | Laminin subunit gamma-1 |
| *LAMP2* | Lysosome-associated membrane glycoprotein 2 |
| *LANCL1* | LanC-like protein 1 |
| *LARP7* | La-related protein 7 |
| *LASP1* | LIM and SH3 domain protein 1 |
| *LATS1* | Serine/threonine-protein kinase LATS1 |
| *LBP* | Lipopolysaccharide-binding protein |
| *LCN1* | Lipocalin-1 |
| *LDHA* | L-lactate dehydrogenase A chain |
| *LDHB* | L-lactate dehydrogenase B chain |
| *LGALS1* | Galectin-1 |
| *LGI1* | Leucine-rich glioma-inactivated protein 1 |
| *LHFPL2* | Lipoma HMGIC fusion partner-like 2 protein |
| *LIMA1* | LIM domain and actin-binding protein 1 |
| *LIMCH1* | LIM and calponin homology domains-containing protein 1 |
| *LIMD1* | LIM domain-containing protein 1 |
| *LIMS1* | LIM and senescent cell antigen-like-containing domain protein 1 |
| *LIN7C* | Protein lin-7 homolog C |
| *LLGL1* | Lethal(2) giant larvae protein homolog 1 |
| *LMO7* | LIM domain only protein 7 |
| *LNPEP* | Leucyl-cystinyl aminopeptidase |
| *LOXL2* | Lysyl oxidase homolog 2 |
| *LPP* | Lipoma-preferred partner |
| *LPXN* | Leupaxin |
| *LRMP* | Lymphoid-restricted membrane protein |
| *LRP1* | Prolow-density lipoprotein receptor-related protein 1 |
| *LRPPRC* | Leucine-rich PPR motif-containing protein, mitochondrial |
| *LRRC17* | Leucine-rich repeat-containing protein 17 |
| *LRRC8A* | Volume-regulated anion channel subunit LRRC8A |
| *LRRIQ3* | Leucine-rich repeat and IQ domain-containing protein 3 |
| *LRRK2* | Leucine-rich repeat serine/threonine-protein kinase 2 |
| *LSM14A* | Protein LSM14 homolog A |
| *LTBP1* | Latent-transforming growth factor beta-binding protein 1 |
| *LTBP4* | Latent-transforming growth factor beta-binding protein 4 |
| *LTF* | Lactotransferrin |
| *LYZ* | Lysozyme C |
| *MACF1* | Microtubule-actin cross-linking factor 1, isoforms 1/2/3/5 |
| *MAD1L1* | Mitotic spindle assembly checkpoint protein MAD1 |
| *MAGI3* | Membrane-associated guanylate kinase, WW and PDZ domain-containing protein 3 |
| *MAN2C1* | Alpha-mannosidase 2C1 |
| *MANF* | Mesencephalic astrocyte-derived neurotrophic factor |
| *MAP1A* | Microtubule-associated protein 1A |
| *MAP2* | Microtubule-associated protein 2 |
| *MAP4* | Microtubule-associated protein 4 |
| *MAP6* | Microtubule-associated protein 6 |
| *MAP7D2* | MAP7 domain-containing protein 2 |
| *MARCKS* | Myristoylated alanine-rich C-kinase substrate |
| *MARCKSL1* | MARCKS-related protein |
| *MARK2* | Serine/threonine-protein kinase MARK2 |
| *MASP1* | Mannan-binding lectin serine protease 1 |
| *MATN2* | Matrilin-2 |
| *MCM9* | DNA helicase MCM9 |
| *MDK* | Midkine |
| *MDN1* | Midasin |
| *MET* | Hepatocyte growth factor receptor |
| *METTL7B* | Methyltransferase-like protein 7B |
| *MFGE8* | Lactadherin |
| *MGAM2* | Probable maltase-glucoamylase 2 |
| *MICAL2* | [F-actin]-methionine sulfoxide oxidase MICAL2 |
| *MIF* | Macrophage migration inhibitory factor |
| *MLLT6* | Protein AF-17 |
| *MME* | Neprilysin |
| *MMP14* | Matrix metalloproteinase-14 |
| *MMP15* | Matrix metalloproteinase-15 |
| *MMRN1* | Multimerin-1 |
| *MMS22L* | Protein MMS22-like |
| *MOB1A* | MOB kinase activator 1A |
| *MPP1* | 55 kDa erythrocyte membrane protein |
| *MPP6* | MAGUK p55 subfamily member 6 |
| *MPRIP* | Myosin phosphatase Rho-interacting protein |
| *MPZL1* | Myelin protein zero-like protein 1 |
| *MRC2* | C-type mannose receptor 2 |
| *MSN* | Moesin |
| *MTAP* | S-methyl-5'-thioadenosine phosphorylase |
| *MTHFD2* | Bifunctional methylenetetrahydrofolate dehydrogenase/cyclohydrolase, mitochondrial |
| *MTOR* | Serine/threonine-protein kinase mTOR |
| *MTPN* | Myotrophin |
| *MUC16* | Mucin-16 |
| *MUC20* | Mucin-20 |
| *MXRA8* | Matrix-remodeling-associated protein 8 |
| *MYADM* | Myeloid-associated differentiation marker |
| *MYCBP* | C-Myc-binding protein |
| *MYH10* | Myosin-10 |
| *MYH11* | Myosin-11 |
| *MYH14* | Myosin-14 |
| *MYH9* | Myosin-9 |
| *MYL12A* | Myosin regulatory light chain 12A |
| *MYL6* | Myosin light polypeptide 6 |
| *MYL9* | Myosin regulatory light polypeptide 9 |
| *MYLK* | Myosin light chain kinase, smooth muscle |
| *MYO18B* | Unconventional myosin-XVIIIb |
| *MYO19* | Unconventional myosin-XIX |
| *MYO1A* | Unconventional myosin-Ia |
| *MYO1B* | Unconventional myosin-Ib |
| *MYO1C* | Unconventional myosin-Ic |
| *MYO5A* | Unconventional myosin-Va |
| *MYO5B* | Unconventional myosin-Vb |
| *MYO9A* | Unconventional myosin-IXa |
| *MYOF* | Myoferlin |
| *MYPN* | Myopalladin |
| *MYT1L* | Myelin transcription factor 1-like protein |
| *NAB1* | NGFI-A-binding protein 1 |
| *NACA* | Nascent polypeptide-associated complex subunit alpha, muscle-specific form |
| *NAPSA* | Napsin-A |
| *NCEH1* | Neutral cholesterol ester hydrolase 1 |
| *NCK1* | Cytoplasmic protein NCK1 |
| *NCL* | Nucleolin |
| *NCOR1* | Nuclear receptor corepressor 1 |
| *NCOR2* | Nuclear receptor corepressor 2 |
| *NDUFS8* | NADH dehydrogenase [ubiquinone] iron-sulfur protein 8, mitochondrial |
| *NEBL* | Nebulette |
| *NECAP2* | Adaptin ear-binding coat-associated protein 2 |
| *NECTIN2* | Nectin-2 |
| *NEUROG3* | Neurogenin-3 |
| *NEXN* | Nexilin |
| *NFRKB* | Nuclear factor related to kappa-B-binding protein |
| *NGF* | Beta-nerve growth factor |
| *NLN* | Neurolysin, mitochondrial |
| *NME1* | Nucleoside diphosphate kinase A |
| *NOTCH2* | Neurogenic locus notch homolog protein 2 |
| *NOV* | Protein NOV homolog |
| *NPR2* | Atrial natriuretic peptide receptor 2 |
| *NPTN* | Neuroplastin |
| *NRIP1* | Nuclear receptor-interacting protein 1 |
| *NRK* | Nik-related protein kinase |
| *NRP1* | Neuropilin-1 |
| *NSDHL* | Sterol-4-alpha-carboxylate 3-dehydrogenase, decarboxylating |
| *NT5C1B* | Cytosolic 5'-nucleotidase 1B |
| *NT5E* | 5'-nucleotidase |
| *NTN4* | Netrin-4 |
| *NUMB* | Protein numb homolog |
| *NUMBL* | Numb-like protein |
| *OCC1* | Overexpressed in colon carcinoma 1 protein |
| *ODF2* | Outer dense fiber protein 2 |
| *OR1B1* | Olfactory receptor 1B1 |
| *OR6Y1* | Olfactory receptor 6Y1 |
| *OSBPL8* | Oxysterol-binding protein-related protein 8 |
| *P2RX5* | P2X purinoceptor 5 |
| *P4HB* | Protein disulfide-isomerase |
| *PA2G4* | Proliferation-associated protein 2G4 |
| *PABPC1* | Polyadenylate-binding protein 1 |
| *PABPC4* | Polyadenylate-binding protein 4 |
| *PACSIN2* | Protein kinase C and casein kinase substrate in neurons protein 2 |
| *PACSIN3* | Protein kinase C and casein kinase substrate in neurons protein 3 |
| *PAK2* | Serine/threonine-protein kinase PAK 2 |
| *PALD1* | Paladin |
| *PALLD* | Palladin |
| *PAN2* | PAB-dependent poly(A)-specific ribonuclease subunit PAN2 |
| *PARK7* | Protein DJ-1 |
| *PARN* | Poly(A)-specific ribonuclease PARN |
| *PARVA* | Alpha-parvin |
| *PAWR* | PRKC apoptosis WT1 regulator protein |
| *PCBP1* | Poly(rC)-binding protein 1 |
| *PCBP2* | Poly(rC)-binding protein 2 |
| *PCDH11X* | Protocadherin-11 X-linked |
| *PCDH15* | Protocadherin-15 |
| *PCSK5* | Proprotein convertase subtilisin/kexin type 5 |
| *PCSK9* | Proprotein convertase subtilisin/kexin type 9 |
| *PDAP1* | 28 kDa heat- and acid-stable phosphoprotein |
| *PDCD6IP* | Programmed cell death 6-interacting protein |
| *PDE1C* | Calcium/calmodulin-dependent 3',5'-cyclic nucleotide phosphodiesterase 1C |
| *PDGFC* | Platelet-derived growth factor C |
| *PDIA3* | Protein disulfide-isomerase A3 |
| *PDIA6* | Protein disulfide-isomerase A6 |
| *PDLIM1* | PDZ and LIM domain protein 1 |
| *PDLIM2* | PDZ and LIM domain protein 2 |
| *PDLIM4* | PDZ and LIM domain protein 4 |
| *PDLIM5* | PDZ and LIM domain protein 5 |
| *PDLIM7* | PDZ and LIM domain protein 7 |
| *PDZRN3* | E3 ubiquitin-protein ligase PDZRN3 |
| *PEBP1* | Phosphatidylethanolamine-binding protein 1 |
| *PFDN2* | Prefoldin subunit 2 |
| *PFN1* | Profilin-1 |
| *PFN2* | Profilin-2 |
| *PGAM1* | Phosphoglycerate mutase 1 |
| *PGK1* | Phosphoglycerate kinase 1 |
| *PGM1* | Phosphoglucomutase-1 |
| *PGRMC1* | Membrane-associated progesterone receptor component 1 |
| *PHF10* | PHD finger protein 10 |
| *PHLDA1* | Pleckstrin homology-like domain family A member 1 |
| *PHLDB1* | Pleckstrin homology-like domain family B member 1 |
| *PHLDB2* | Pleckstrin homology-like domain family B member 2 |
| *PICALM* | Phosphatidylinositol-binding clathrin assembly protein |
| *PIP* | Prolactin-inducible protein |
| *PIP4K2A* | Phosphatidylinositol 5-phosphate 4-kinase type-2 alpha |
| *PKM* | Pyruvate kinase PKM |
| *PKP2* | Plakophilin-2 |
| *PLA1A* | Phospholipase A1 member A |
| *PLAG1* | Zinc finger protein PLAG1 |
| *PLAT* | Tissue-type plasminogen activator |
| *PLAU* | Urokinase-type plasminogen activator |
| *PLAUR* | Urokinase plasminogen activator surface receptor |
| *PLEC* | Plectin |
| *PLEK* | Pleckstrin |
| *PLG* | Plasminogen |
| *PLP2* | Proteolipid protein 2 |
| *PLS1* | Plastin-1 |
| *PLS3* | Plastin-3 |
| *PLSCR1* | Phospholipid scramblase 1 |
| *PLXNA3* | Plexin-A3 |
| *PLXNB2* | Plexin-B2 |
| *PODXL* | Podocalyxin |
| *POLK* | DNA polymerase kappa |
| *POLR2H* | DNA-directed RNA polymerases I, II, and III subunit RPABC3 |
| *POLR3A* | DNA-directed RNA polymerase III subunit RPC1 |
| *POSTN* | Periostin |
| *POTEF* | POTE ankyrin domain family member F |
| *PPFIA1* | Liprin-alpha-1 |
| *PPFIA3* | Liprin-alpha-3 |
| *PPFIBP1* | Liprin-beta-1 |
| *PPIA* | Peptidyl-prolyl cis-trans isomerase A |
| *PPIAL4A* | Peptidyl-prolyl cis-trans isomerase A-like 4A |
| *PPIB* | Peptidyl-prolyl cis-trans isomerase B |
| *PPIE* | Peptidyl-prolyl cis-trans isomerase E |
| *PPIP5K2* | Inositol hexakisphosphate and diphosphoinositol-pentakisphosphate kinase 2 |
| *PPP1CC* | Serine/threonine-protein phosphatase PP1-gamma catalytic subunit |
| *PPP1R12A* | Protein phosphatase 1 regulatory subunit 12A |
| *PPP1R16A* | Protein phosphatase 1 regulatory subunit 16A |
| *PPP1R18* | Phostensin |
| *PPP2R1A* | Serine/threonine-protein phosphatase 2A 65 kDa regulatory subunit A alpha isoform |
| *PPP2R3A* | Serine/threonine-protein phosphatase 2A regulatory subunit B'' subunit alpha |
| *PRAMEF7* | PRAME family member 7 |
| *PRDM1* | PR domain zinc finger protein 1 |
| *PRDX1* | Peroxiredoxin-1 |
| *PRDX2* | Peroxiredoxin-2 |
| *PRDX6* | Peroxiredoxin-6 |
| *PRG4* | Proteoglycan 4 |
| *PRKCSH* | Glucosidase 2 subunit beta |
| *PRLR* | Prolactin receptor |
| *PRNP* | Major prion protein |
| *PROCR* | Endothelial protein C receptor |
| *PRSS12* | Neurotrypsin |
| *PRSS23* | Serine protease 23 |
| *PRSS3* | Trypsin-3 |
| *PSEN1* | Presenilin-1 |
| *PSMA3* | Proteasome subunit alpha type-3 |
| *PSMD7* | 26S proteasome non-ATPase regulatory subunit 7 |
| *PTK2* | Focal adhesion kinase 1 |
| *PTK7* | Inactive tyrosine-protein kinase 7 |
| *PTPN13* | Tyrosine-protein phosphatase non-receptor type 13 |
| *PTPRA* | Receptor-type tyrosine-protein phosphatase alpha |
| *PTPRJ* | Receptor-type tyrosine-protein phosphatase eta |
| *PTPRK* | Receptor-type tyrosine-protein phosphatase kappa |
| *PTRF* | Polymerase I and transcript release factor |
| *PTTG1IP* | Pituitary tumor-transforming gene 1 protein-interacting protein |
| *PTX3* | Pentraxin-related protein PTX3 |
| *PVR* | Poliovirus receptor |
| *PXDN* | Peroxidasin homolog |
| *PXN* | Paxillin |
| *QSOX1* | Sulfhydryl oxidase 1 |
| *QSOX2* | Sulfhydryl oxidase 2 |
| *QTRT2* | Queuine tRNA-ribosyltransferase accessory subunit 2 |
| *RAB10* | Ras-related protein Rab-10 |
| *RAB11A* | Ras-related protein Rab-11A |
| *RAB13* | Ras-related protein Rab-13 |
| *RAB1A* | Ras-related protein Rab-1A |
| *RAB21* | Ras-related protein Rab-21 |
| *RAB23* | Ras-related protein Rab-23 |
| *RAB2B* | Ras-related protein Rab-2B |
| *RAB34* | Ras-related protein Rab-34 |
| *RAB40C* | Ras-related protein Rab-40C |
| *RAB5C* | Ras-related protein Rab-5C |
| *RAB7A* | Ras-related protein Rab-7a |
| *RABGAP1* | Rab GTPase-activating protein 1 |
| *RAC1* | Ras-related C3 botulinum toxin substrate 1 |
| *RACK1* | Receptor of activated protein C kinase 1 |
| *RAI14* | Ankycorbin |
| *RALA* | Ras-related protein Ral-A |
| *RALB* | Ras-related protein Ral-B |
| *RAN* | GTP-binding nuclear protein Ran |
| *RAP1A* | Ras-related protein Rap-1A |
| *RAP1B* | Ras-related protein Rap-1b |
| *RAP2B* | Ras-related protein Rap-2b |
| *RAPH1* | Ras-associated and pleckstrin homology domains-containing protein 1 |
| *RASA2* | Ras GTPase-activating protein 2 |
| *RASA3* | Ras GTPase-activating protein 3 |
| *RCC1* | Regulator of chromosome condensation |
| *RCN1* | Reticulocalbin-1 |
| *RDX* | Radixin |
| *RFLNB* | Refilin-B |
| *RFNG* | Beta-1,3-N-acetylglucosaminyltransferase radical fringe |
| *RHOA* | Transforming protein RhoA |
| *RHOG* | Rho-related GTP-binding protein RhoG |
| *RIMBP3* | RIMS-binding protein 3A |
| *RNASE4* | Ribonuclease 4 |
| *RNF40* | E3 ubiquitin-protein ligase BRE1B |
| *RNH1* | Ribonuclease inhibitor |
| *RNPEPL1* | Aminopeptidase RNPEPL1 |
| *RORB* | Nuclear receptor ROR-beta |
| *RP2* | Protein XRP2 |
| *RPL10* | 60S ribosomal protein L10 |
| *RPL11* | 60S ribosomal protein L11 |
| *RPL12* | 60S ribosomal protein L12 |
| *RPL13* | 60S ribosomal protein L13 |
| *RPL14* | 60S ribosomal protein L14 |
| *RPL18* | 60S ribosomal protein L18 |
| *RPL19* | 60S ribosomal protein L19 |
| *RPL21* | 60S ribosomal protein L21 |
| *RPL22* | 60S ribosomal protein L22 |
| *RPL23* | 60S ribosomal protein L23 |
| *RPL24* | 60S ribosomal protein L24 |
| *RPL26* | 60S ribosomal protein L26 |
| *RPL27* | 60S ribosomal protein L27 |
| *RPL28* | 60S ribosomal protein L28 |
| *RPL29* | 60S ribosomal protein L29 |
| *RPL3* | 60S ribosomal protein L3 |
| *RPL30* | 60S ribosomal protein L30 |
| *RPL37A* | 60S ribosomal protein L37a |
| *RPL38* | 60S ribosomal protein L38 |
| *RPL4* | 60S ribosomal protein L4 |
| *RPL5* | 60S ribosomal protein L5 |
| *RPL6* | 60S ribosomal protein L6 |
| *RPL7* | 60S ribosomal protein L7 |
| *RPL7A* | 60S ribosomal protein L7a |
| *RPL8* | 60S ribosomal protein L8 |
| *RPL9* | 60S ribosomal protein L9 |
| *RPLP0* | 60S acidic ribosomal protein P0 |
| *RPLP1* | 60S acidic ribosomal protein P1 |
| *RPLP2* | 60S acidic ribosomal protein P2 |
| *RPN1* | Dolichyl-diphosphooligosaccharide--protein glycosyltransferase subunit 1 |
| *RPS10* | 40S ribosomal protein S10 |
| *RPS10P5* | Putative 40S ribosomal protein S10-like |
| *RPS11* | 40S ribosomal protein S11 |
| *RPS12* | 40S ribosomal protein S12 |
| *RPS13* | 40S ribosomal protein S13 |
| *RPS14* | 40S ribosomal protein S14 |
| *RPS15A* | 40S ribosomal protein S15a |
| *RPS16* | 40S ribosomal protein S16 |
| *RPS17* | 40S ribosomal protein S17 |
| *RPS18* | 40S ribosomal protein S18 |
| *RPS19* | 40S ribosomal protein S19 |
| *RPS2* | 40S ribosomal protein S2 |
| *RPS20* | 40S ribosomal protein S20 |
| *RPS21* | 40S ribosomal protein S21 |
| *RPS23* | 40S ribosomal protein S23 |
| *RPS25* | 40S ribosomal protein S25 |
| *RPS27A* | Ubiquitin-40S ribosomal protein S27a |
| *RPS27L* | 40S ribosomal protein S27-like |
| *RPS28* | 40S ribosomal protein S28 |
| *RPS29* | 40S ribosomal protein S29 |
| *RPS3* | 40S ribosomal protein S3 |
| *RPS3A* | 40S ribosomal protein S3a |
| *RPS4X* | 40S ribosomal protein S4, X isoform |
| *RPS5* | 40S ribosomal protein S5 |
| *RPS6* | 40S ribosomal protein S6 |
| *RPS7* | 40S ribosomal protein S7 |
| *RPS8* | 40S ribosomal protein S8 |
| *RPS9* | 40S ribosomal protein S9 |
| *RPSA* | 40S ribosomal protein SA |
| *RRAS* | Ras-related protein R-Ras |
| *RRAS2* | Ras-related protein R-Ras2 |
| *RSF1* | Remodeling and spacing factor 1 |
| *RSU1* | Ras suppressor protein 1 |
| *RTN4* | Reticulon-4 |
| *S100A10* | Protein S100-A10 |
| *S100A13* | Protein S100-A13 |
| *S100A6* | Protein S100-A6 |
| *S100A7* | Protein S100-A7 |
| *S100A9* | Protein S100-A9 |
| *SACS* | Sacsin |
| *SBDS* | Ribosome maturation protein SBDS |
| *SBNO2* | Protein strawberry notch homolog 2 |
| *SCAMP1* | Secretory carrier-associated membrane protein 1 |
| *SCAMP3* | Secretory carrier-associated membrane protein 3 |
| *SCAMP4* | Secretory carrier-associated membrane protein 4 |
| *SCN11A* | Sodium channel protein type 11 subunit alpha |
| *SCRIB* | Protein scribble homolog |
| *SDC1* | Syndecan-1 |
| *SDC2* | Syndecan-2 |
| *SDPR* | Serum deprivation-response protein |
| *SEC22B* | Vesicle-trafficking protein SEC22b |
| *SEMA3A* | Semaphorin-3A |
| *SEMA3B* | Semaphorin-3B |
| *SEMA3C* | Semaphorin-3C |
| *SEMA7A* | Semaphorin-7A |
| *SEP10* | Septin-10 |
| *SEP11* | Septin-11 |
| *SEPT1* | Septin-1 |
| *SEPT2* | Septin-2 |
| *SEPT6* | Septin-6 |
| *SEPT7* | Septin-7 |
| *SEPT8* | Septin-8 |
| *SEPT9* | Septin-9 |
| *SERBP1* | Plasminogen activator inhibitor 1 RNA-binding protein |
| *SERF2* | Small EDRK-rich factor 2 |
| *SERPINA1* | Alpha-1-antitrypsin |
| *SERPINC1* | Antithrombin-III |
| *SERPIND1* | Heparin cofactor 2 |
| *SERPINE1* | Plasminogen activator inhibitor 1 |
| *SERPINF1* | Pigment epithelium-derived factor |
| *SERPINF2* | Alpha-2-antiplasmin |
| *SERPINH1* | Serpin H1 |
| *SETD3* | Histone-lysine N-methyltransferase setd3 |
| *SF3B2* | Splicing factor 3B subunit 2 |
| *SFRP1* | Secreted frizzled-related protein 1 |
| *SH3BP4* | SH3 domain-binding protein 4 |
| *SH3GL2* | Endophilin-A1 |
| *SHROOM3* | Protein Shroom3 |
| *SIM1* | Single-minded homolog 1 |
| *SIRPA* | Tyrosine-protein phosphatase non-receptor type substrate 1 |
| *SIX6OS1* | Protein SIX6OS1 |
| *SKP1* | S-phase kinase-associated protein 1 |
| *SLC12A1* | Solute carrier family 12 member 1 |
| *SLC13A3* | Solute carrier family 13 member 3 |
| *SLC16A1* | Monocarboxylate transporter 1 |
| *SLC16A3* | Monocarboxylate transporter 4 |
| *SLC1A5* | Neutral amino acid transporter B(0) |
| *SLC25A3* | Phosphate carrier protein, mitochondrial |
| *SLC25A5* | ADP/ATP translocase 2 |
| *SLC29A1* | Equilibrative nucleoside transporter 1 |
| *SLC2A1* | Solute carrier family 2, facilitated glucose transporter member 1 |
| *SLC38A2* | Sodium-coupled neutral amino acid transporter 2 |
| *SLC39A10* | Zinc transporter ZIP10 |
| *SLC39A12* | Zinc transporter ZIP12 |
| *SLC39A14* | Zinc transporter ZIP14 |
| *SLC3A2* | 4F2 cell-surface antigen heavy chain |
| *SLC44A1* | Choline transporter-like protein 1 |
| *SLC4A7* | Sodium bicarbonate cotransporter 3 |
| *SLC7A1* | High affinity cationic amino acid transporter 1 |
| *SLC7A5* | Large neutral amino acids transporter small subunit 1 |
| *SLC9A2* | Sodium/hydrogen exchanger 2 |
| *SLC9A3R1* | Na(+)/H(+) exchange regulatory cofactor NHE-RF1 |
| *SLFN14* | Protein SLFN14 |
| *SLIT1* | Slit homolog 1 protein |
| *SMARCA2* | Probable global transcription activator SNF2L2 |
| *SMARCA5* | SWI/SNF-related matrix-associated actin-dependent regulator of chromatin subfamily A member 5 |
| *SMC2* | Structural maintenance of chromosomes protein 2 |
| *SNAP23* | Synaptosomal-associated protein 23 |
| *SNRPD2* | Small nuclear ribonucleoprotein Sm D2 |
| *SNRPD3* | Small nuclear ribonucleoprotein Sm D3 |
| *SNRPE* | Small nuclear ribonucleoprotein E |
| *SNRPF* | Small nuclear ribonucleoprotein F |
| *SNTB2* | Beta-2-syntrophin |
| *SORBS2* | Sorbin and SH3 domain-containing protein 2 |
| *SORBS3* | Vinexin |
| *SORT1* | Sortilin |
| *SPECC1* | Cytospin-B |
| *SPEF2* | Sperm flagellar protein 2 |
| *SPTAN1* | Spectrin alpha chain, non-erythrocytic 1 |
| *SPTB* | Spectrin beta chain, erythrocytic |
| *SPTBN1* | Spectrin beta chain, non-erythrocytic 1 |
| *SREK1* | Splicing regulatory glutamine/lysine-rich protein 1 |
| *SRP9* | Signal recognition particle 9 kDa protein |
| *SRPX* | Sushi repeat-containing protein SRPX |
| *SRSF3* | Serine/arginine-rich splicing factor 3 |
| *SSC4D* | Scavenger receptor cysteine-rich domain-containing group B protein |
| *SSFA2* | Sperm-specific antigen 2 |
| *ST13P5* | Putative protein FAM10A5 |
| *ST7* | Suppressor of tumorigenicity 7 protein |
| *STC1* | Stanniocalcin-1 |
| *STEAP3* | Metalloreductase STEAP3 |
| *STIM2* | Stromal interaction molecule 2 |
| *STMN1* | Stathmin |
| *STRAP* | Serine-threonine kinase receptor-associated protein |
| *STX12* | Syntaxin-12 |
| *STX4* | Syntaxin-4 |
| *STX7* | Syntaxin-7 |
| *STXBP1* | Syntaxin-binding protein 1 |
| *STXBP3* | Syntaxin-binding protein 3 |
| *SVIP* | Small VCP/p97-interacting protein |
| *SYCP1* | Synaptonemal complex protein 1 |
| *SYK* | Tyrosine-protein kinase SYK |
| *SYNCRIP* | Heterogeneous nuclear ribonucleoprotein Q |
| *SYNE1* | Nesprin-1 |
| *SYNE2* | Nesprin-2 |
| *SYNJ2* | Synaptojanin-2 |
| *SYNM* | Synemin |
| *SYTL2* | Synaptotagmin-like protein 2 |
| *TAF4B* | Transcription initiation factor TFIID subunit 4B |
| *TAF7* | Transcription initiation factor TFIID subunit 7 |
| *TAGLN* | Transgelin |
| *TAGLN2* | Transgelin-2 |
| *TAGLN3* | Transgelin-3 |
| *TALDO1* | Transaldolase |
| *TAOK2* | Serine/threonine-protein kinase TAO2 |
| *TAX1BP1* | Tax1-binding protein 1 |
| *TBC1D16* | TBC1 domain family member 16 |
| *TBC1D24* | TBC1 domain family member 24 |
| *TBC1D8* | TBC1 domain family member 8 |
| *TBC1D8B* | TBC1 domain family member 8B |
| *TBL3* | Transducin beta-like protein 3 |
| *TDRD15* | Tudor domain-containing protein 15 |
| *TERT* | Telomerase reverse transcriptase |
| *TF* | Serotransferrin |
| *TFPI* | Tissue factor pathway inhibitor |
| *TFPI2* | Tissue factor pathway inhibitor 2 |
| *TFRC* | Transferrin receptor protein 1 |
| *TGFB1* | Transforming growth factor beta-1 |
| *TGFB1I1* | Transforming growth factor beta-1-induced transcript 1 protein |
| *TGFB2* | Transforming growth factor beta-2 |
| *TGFBI* | Transforming growth factor-beta-induced protein ig-h3 |
| *TGOLN2* | Trans-Golgi network integral membrane protein 2 |
| *THADA* | Thyroid adenoma-associated protein |
| *THBS1* | Thrombospondin-1 |
| *THBS2* | Thrombospondin-2 |
| *THBS4* | Thrombospondin-4 |
| *THRAP3* | Thyroid hormone receptor-associated protein 3 |
| *THSD4* | Thrombospondin type-1 domain-containing protein 4 |
| *THY1* | Thy-1 membrane glycoprotein |
| *TIMM8A* | Mitochondrial import inner membrane translocase subunit Tim8 A |
| *TIMP3* | Metalloproteinase inhibitor 3 |
| *TIPARP* | TCDD-inducible poly [ADP-ribose] polymerase |
| *TJAP1* | Tight junction-associated protein 1 |
| *TKFC* | Triokinase/FMN cyclase |
| *TKT* | Transketolase |
| *TLDC1* | TLD domain-containing protein 1 |
| *TLL1* | Tolloid-like protein 1 |
| *TLL2* | Tolloid-like protein 2 |
| *TLN1* | Talin-1 |
| *TLN2* | Talin-2 |
| *TLR5* | Toll-like receptor 5 |
| *TMBIM1* | Protein lifeguard 3 |
| *TMC3* | Transmembrane channel-like protein 3 |
| *TMEFF1* | Tomoregulin-1 |
| *TMEM109* | Transmembrane protein 109 |
| *TMEM123* | Porimin |
| *TMEM2* | Transmembrane protein 2 |
| *TMEM30A* | Cell cycle control protein 50A |
| *TMEM33* | Transmembrane protein 33 |
| *TMOD3* | Tropomodulin-3 |
| *TMPRSS4* | Transmembrane protease serine 4 |
| *TNC* | Tenascin |
| *TNFRSF12A* | Tumor necrosis factor receptor superfamily member 12A |
| *TNKS1BP1* | 182 kDa tankyrase-1-binding protein |
| *TNS1* | Tensin-1 |
| *TNS2* | Tensin-2 |
| *TNS3* | Tensin-3 |
| *TNS4* | Tensin-4 |
| *TPBG* | Trophoblast glycoprotein |
| *TPD52L2* | Tumor protein D54 |
| *TPI1* | Triosephosphate isomerase |
| *TPM1* | Tropomyosin alpha-1 chain |
| *TPM2* | Tropomyosin beta chain |
| *TPM3* | Tropomyosin alpha-3 chain |
| *TPM4* | Tropomyosin alpha-4 chain |
| *TPT1* | Translationally-controlled tumor protein |
| *TRAK1* | Trafficking kinesin-binding protein 1 |
| *TRAP1* | Heat shock protein 75 kDa, mitochondrial |
| *TRDN* | Triadin |
| *TRHDE* | Thyrotropin-releasing hormone-degrading ectoenzyme |
| *TRIM4* | E3 ubiquitin-protein ligase TRIM4 |
| *TRIM58* | E3 ubiquitin-protein ligase TRIM58 |
| *TRIM72* | Tripartite motif-containing protein 72 |
| *TRIO* | Triple functional domain protein |
| *TRIOBP* | TRIO and F-actin-binding protein |
| *TRMT10B* | tRNA methyltransferase 10 homolog B |
| *TRPC7* | Short transient receptor potential channel 7 |
| *TRPM2* | Transient receptor potential cation channel subfamily M member 2 |
| *TSNAXIP1* | Translin-associated factor X-interacting protein 1 |
| *TSPAN14* | Tetraspanin-14 |
| *TSPOAP1* | Peripheral-type benzodiazepine receptor-associated protein 1 |
| *TTBK1* | Tau-tubulin kinase 1 |
| *TTC17* | Tetratricopeptide repeat protein 17 |
| *TTC3* | E3 ubiquitin-protein ligase TTC3 |
| *TUBA1A* | Tubulin alpha-1A chain |
| *TUBA4A* | Tubulin alpha-4A chain |
| *TUBAL3* | Tubulin alpha chain-like 3 |
| *TUBB* | Tubulin beta chain |
| *TUBB1* | Tubulin beta-1 chain |
| *TUBB4B* | Tubulin beta-4B chain |
| *TXN* | Thioredoxin |
| *TXNDC5* | Thioredoxin domain-containing protein 5 |
| *TXNRD1* | Thioredoxin reductase 1, cytoplasmic |
| *TYK2* | Non-receptor tyrosine-protein kinase TYK2 |
| *TYMSOS* | TYMS opposite strand protein |
| *UACA* | Uveal autoantigen with coiled-coil domains and ankyrin repeats |
| *UBA1* | Ubiquitin-like modifier-activating enzyme 1 |
| *UBAP2* | Ubiquitin-associated protein 2 |
| *UBAP2L* | Ubiquitin-associated protein 2-like |
| *UBE2N* | Ubiquitin-conjugating enzyme E2 N |
| *UBE2V1* | Ubiquitin-conjugating enzyme E2 variant 1 |
| *UBR4* | E3 ubiquitin-protein ligase UBR4 |
| *UBTD2* | Ubiquitin domain-containing protein 2 |
| *UBTFL1* | Upstream-binding factor 1-like protein 1 |
| *UCHL1* | Ubiquitin carboxyl-terminal hydrolase isozyme L1 |
| *UGDH* | UDP-glucose 6-dehydrogenase |
| *UHRF1* | E3 ubiquitin-protein ligase UHRF1 |
| *UNC80* | Protein unc-80 homolog |
| *USH2A* | Usherin |
| *UTP20* | Small subunit processome component 20 homolog |
| *UTRN* | Utrophin |
| *VAMP3* | Vesicle-associated membrane protein 3 |
| *VASN* | Vasorin |
| *VASP* | Vasodilator-stimulated phosphoprotein |
| *VAT1* | Synaptic vesicle membrane protein VAT-1 homolog |
| *VAV1* | Proto-oncogene vav |
| *VCAN* | Versican core protein |
| *VCL* | Vinculin |
| *VCP* | Transitional endoplasmic reticulum ATPase |
| *VDAC2* | Voltage-dependent anion-selective channel protein 2 |
| *VIM* | Vimentin |
| *VIPR1* | Vasoactive intestinal polypeptide receptor 1 |
| *VTA1* | Vacuolar protein sorting-associated protein VTA1 homolog |
| *VTN* | Vitronectin |
| *VWA7* | von Willebrand factor A domain-containing protein 7 |
| *VWF* | von Willebrand factor |
| *WASF2* | Wiskott-Aldrich syndrome protein family member 2 |
| *WDR1* | WD repeat-containing protein 1 |
| *WNT5A* | Protein Wnt-5a |
| *WNT5B* | Protein Wnt-5b |
| *WNT7A* | Protein Wnt-7a |
| *WRN* | Werner syndrome ATP-dependent helicase |
| *XAF1* | XIAP-associated factor 1 |
| *XAGE5* | X antigen family member 5 |
| *YBX1* | Nuclease-sensitive element-binding protein 1 |
| *YES1* | Tyrosine-protein kinase Yes |
| *YWHAB* | 14-3-3 protein beta/alpha |
| *YWHAE* | 14-3-3 protein epsilon |
| *YWHAG* | 14-3-3 protein gamma |
| *YWHAH* | 14-3-3 protein eta |
| *YWHAQ* | 14-3-3 protein theta |
| *YWHAZ* | 14-3-3 protein zeta/delta |
| *ZBED9* | SCAN domain-containing protein 3 |
| *ZC2HC1B* | Zinc finger C2HC domain-containing protein 1B |
| *ZC3H15* | Zinc finger CCCH domain-containing protein 15 |
| *ZC3H7A* | Zinc finger CCCH domain-containing protein 7A |
| *ZMYND15* | Zinc finger MYND domain-containing protein 15 |
| *ZNF140* | Zinc finger protein 140 |
| *ZNF211* | Zinc finger protein 211 |
| *ZNF248* | Zinc finger protein 248 |
| *ZNF529* | Zinc finger protein 529 |
| *ZNF561* | Zinc finger protein 561 |
| *ZNF562* | Zinc finger protein 562 |
| *ZNF648* | Zinc finger protein 648 |
| *ZYG11B* | Protein zyg-11 homolog B |
| *ZYX* | Zyxin |
