# Supplementary material for: Centrosome guides spatial activation of Rac to control cell polarization and directed cell migration
Source: Life Sci Alliance. 2019 Feb 8;2(1):e201800135. doi: 10.26508/lsa.201800135 (PMC6369537; doi:10.26508/lsa.201800135)
Supplement: Supplementary file 7 [file LSA-2018-00135_TableS4.docx]

**Supplementary Table 4: The list of proteins reproducibly identified in FAs of either RPEp53^-/-^ and RPEp53^-/-^SAS6^-/-^ cells and their centrosome dependence ratios**

**Increased abundance in FA fraction isolated from RPEp53^-/-^ cells**

| **Gene name** | **Description** | **Gene information** | **Centrosome Dependence Ratio** | **p-value**  **(Student's t-test)** |
| --- | --- | --- | --- | --- |
| *A1BG* | Alpha-1B-glycoprotein | [*http://www.genecards.org/cgi-bin/carddisp.pl?gene=A1BG*](http://www.genecards.org/cgi-bin/carddisp.pl?gene=ACTR5) | 2.585536871 | 0.027697805 |
| *A2M* | Alpha-2-macroglobulin | http://www.genecards.org/cgi-bin/carddisp.pl?gene=A2M | 1.474134538 | 0.001064194 |
| *ACTG1* | Actin, cytoplasmic 2 | http://www.genecards.org/cgi-bin/carddisp.pl?gene=ACTG1 | 2.081837959 | 0.000473611 |
| *ACTN1* | Alpha-actinin-1 | http://www.genecards.org/cgi-bin/carddisp.pl?gene=ACTN1 | 1.711834968 | 0.003836311 |
| *ACTN4* | Alpha-actinin-4 | http://www.genecards.org/cgi-bin/carddisp.pl?gene=ACTN4 | 2.225240328 | 0.000102126 |
| *ACTR3* | Actin-related protein 3 | http://www.genecards.org/cgi-bin/carddisp.pl?gene=ACTR3 | 2.415765117 | 0.004970431 |
| *ADAM10* | Disintegrin and metalloproteinase domain-containing protein 10 | http://www.genecards.org/cgi-bin/carddisp.pl?gene=ADAM10 | 1.762479408 | 0.008787453 |
| *ADAM9* | Disintegrin and metalloproteinase domain-containing protein 9 | http://www.genecards.org/cgi-bin/carddisp.pl?gene=ADAM9 | 1.752193203 | 0.01758559 |
| *ADD1* | Alpha-adducin | http://www.genecards.org/cgi-bin/carddisp.pl?gene=ADD1 | 4.949410406 | 0.011034638 |
| *ADD3* | Gamma-adducin | http://www.genecards.org/cgi-bin/carddisp.pl?gene=ADD3 | 9.035375369 | 0.000941607 |
| *ADM* | ADM | http://www.genecards.org/cgi-bin/carddisp.pl?gene=ADM | 2.038520533 | 0.036302416 |
| *AFP* | Alpha-fetoprotein | http://www.genecards.org/cgi-bin/carddisp.pl?gene=AFP | 2.26010528 | 8.89834E-05 |
| *AHNAK* | Neuroblast differentiation-associated protein AHNAK | http://www.genecards.org/cgi-bin/carddisp.pl?gene=AHNAK | 1.582019781 | 0.033241473 |
| *AHNAK2* | Protein AHNAK2 | http://www.genecards.org/cgi-bin/carddisp.pl?gene=AHNAK2 | 1.744577181 | 0.010252302 |
| *AKAP12* | A-kinase anchor protein 12 | http://www.genecards.org/cgi-bin/carddisp.pl?gene=AKAP12 | 2.103517721 | 0.000195991 |
| *ALCAM* | CD166 antigen | http://www.genecards.org/cgi-bin/carddisp.pl?gene=ALCAM | 2.031991171 | 0.025192909 |
| *ANPEP* | Aminopeptidase N | http://www.genecards.org/cgi-bin/carddisp.pl?gene=ANPEP | 2.854011721 | 0.000627876 |
| *ANXA6* | Annexin A6 | http://www.genecards.org/cgi-bin/carddisp.pl?gene=ANXA6 | 1.654410335 | 0.041396136 |
| *APOB* | Apolipoprotein B-100 | http://www.genecards.org/cgi-bin/carddisp.pl?gene=APOB | 2.105543063 | 0.036884879 |
| *APP* | Amyloid beta A4 protein | http://www.genecards.org/cgi-bin/carddisp.pl?gene=APP | 3.608367998 | 0.001882209 |
| *ARHGAP22* | Rho GTPase-activating protein 22 | http://www.genecards.org/cgi-bin/carddisp.pl?gene=ARHGAP22 | 2.906636075 | 0.000109151 |
| *ARHGEF2* | Rho guanine nucleotide exchange factor 2 | http://www.genecards.org/cgi-bin/carddisp.pl?gene=ARHGEF2 | 1.320873629 | 0.03778765 |
| *ARHGEF6* | Rho guanine nucleotide exchange factor 6 | http://www.genecards.org/cgi-bin/carddisp.pl?gene=ARHGEF6 | 4.348964398 | 0.001498306 |
| *ARHGEF7* | Rho guanine nucleotide exchange factor 7 | http://www.genecards.org/cgi-bin/carddisp.pl?gene=ARHGEF7 | 2.798227547 | 0.003675237 |
| *ARPC1A* | Actin-related protein 2/3 complex subunit 1A | http://www.genecards.org/cgi-bin/carddisp.pl?gene=ARPC1A | 2.126162944 | 0.000408366 |
| *ARPC1B* | Actin-related protein 2/3 complex subunit 1B | http://www.genecards.org/cgi-bin/carddisp.pl?gene=ARPC1B | 1.852605265 | 0.005451446 |
| *ARPC2* | Actin-related protein 2/3 complex subunit 2 | http://www.genecards.org/cgi-bin/carddisp.pl?gene=ARPC2 | 2.21629361 | 0.00547618 |
| *ARPC3* | Actin-related protein 2/3 complex subunit 3 | http://www.genecards.org/cgi-bin/carddisp.pl?gene=ARPC3 | 2.538784847 | 0.002387886 |
| *ARPC4* | Actin-related protein 2/3 complex subunit 4 | http://www.genecards.org/cgi-bin/carddisp.pl?gene=ARPC4 | 2.417001894 | 9.40856E-05 |
| *ARPC5* | Actin-related protein 2/3 complex subunit 5 | http://www.genecards.org/cgi-bin/carddisp.pl?gene=ARPC5 | 2.44873032 | 0.009275107 |
| *ARPC5L* | Actin-related protein 2/3 complex subunit 5-like protein | http://www.genecards.org/cgi-bin/carddisp.pl?gene=ARPC5L | 2.028847496 | 0.019228642 |
| *ARSJ* | Arylsulfatase J | http://www.genecards.org/cgi-bin/carddisp.pl?gene=ARSJ | 1.988876703 | 0.000715272 |
| *ASPH* | Aspartyl/asparaginyl beta-hydroxylase | http://www.genecards.org/cgi-bin/carddisp.pl?gene=ASPH | 1.280897496 | 0.001106542 |
| *ATL3* | Atlastin-3 | http://www.genecards.org/cgi-bin/carddisp.pl?gene=ATL3 | 2.34592317 | 0.027401498 |
| *ATP1A1* | Sodium/potassium-transporting ATPase subunit alpha-1 | http://www.genecards.org/cgi-bin/carddisp.pl?gene=ATP1A1 | 1.563190056 | 0.00322545 |
| *ATP1B3* | Sodium/potassium-transporting ATPase subunit beta-3 | http://www.genecards.org/cgi-bin/carddisp.pl?gene=ATP1B3 | 1.407103927 | 0.008259362 |
| *ATP5A1* | ATP synthase subunit alpha, mitochondrial | http://www.genecards.org/cgi-bin/carddisp.pl?gene=ATP5A1 | 3.111226266 | 0.035080637 |
| *ATP5B* | ATP synthase subunit beta, mitochondrial | http://www.genecards.org/cgi-bin/carddisp.pl?gene=ATP5B | 2.331365785 | 0.006974947 |
| *ATP5E* | ATP synthase subunit epsilon, mitochondrial | http://www.genecards.org/cgi-bin/carddisp.pl?gene=ATP5E | 5.769744272 | 0.046172995 |
| *B2M* | Beta-2-microglobulin | http://www.genecards.org/cgi-bin/carddisp.pl?gene=B2M | 1.762558509 | 0.043193303 |
| *BASP1* | Brain acid soluble protein 1 | http://www.genecards.org/cgi-bin/carddisp.pl?gene=BASP1 | 1.526395453 | 0.008960669 |
| *BCL10* | B-cell lymphoma/leukemia 10 | http://www.genecards.org/cgi-bin/carddisp.pl?gene=BCL10 | 2.108811745 | 0.026098078 |
| *BRCA1* | Breast cancer type 1 susceptibility protein | http://www.genecards.org/cgi-bin/carddisp.pl?gene=BRCA1 | 1.243894126 | 0.018953916 |
| *BSG* | Basigin | http://www.genecards.org/cgi-bin/carddisp.pl?gene=BSG | 2.378040565 | 0.004402492 |
| *C3orf58* | Deleted in autism protein 1 | http://www.genecards.org/cgi-bin/carddisp.pl?gene=C3orf58 | 1.443404355 | 0.033289202 |
| *CA9* | Carbonic anhydrase 9 | http://www.genecards.org/cgi-bin/carddisp.pl?gene=CA9 | 2.502086981 | 0.002294612 |
| *CALD1* | Caldesmon | http://www.genecards.org/cgi-bin/carddisp.pl?gene=CALD1 | 1.545206849 | 0.000629263 |
| *CALM1* | Calmodulin | http://www.genecards.org/cgi-bin/carddisp.pl?gene=CALM1 | 1.705916973 | 0.021536338 |
| *CALR* | Calreticulin | http://www.genecards.org/cgi-bin/carddisp.pl?gene=CALR | 1.710051179 | 0.008424371 |
| *CAMK4* | Calcium/calmodulin-dependent protein kinase type IV | http://www.genecards.org/cgi-bin/carddisp.pl?gene=CAMK4 | 1.383063606 | 0.02961886 |
| *CANX* | Calnexin | http://www.genecards.org/cgi-bin/carddisp.pl?gene=CANX | 1.694104343 | 0.046089166 |
| *CAPZB* | F-actin-capping protein subunit beta | http://www.genecards.org/cgi-bin/carddisp.pl?gene=CAPZB | 2.213395207 | 5.12964E-05 |
| *CAV1* | Caveolin-1 | http://www.genecards.org/cgi-bin/carddisp.pl?gene=CAV1 | 3.203908427 | 0.000207713 |
| *CAV2* | Caveolin-2 | http://www.genecards.org/cgi-bin/carddisp.pl?gene=CAV2 | 4.511713928 | 0.007072839 |
| *CBR1* | Carbonyl reductase [NADPH] 1 | http://www.genecards.org/cgi-bin/carddisp.pl?gene=CBR1 | 1.341516022 | 0.04297361 |
| *CCDC80* | Coiled-coil domain-containing protein 80 | http://www.genecards.org/cgi-bin/carddisp.pl?gene=CCDC80 | 2.071438701 | 0.001009409 |
| *CCNY* | Cyclin-Y | http://www.genecards.org/cgi-bin/carddisp.pl?gene=CCNY | 46.74355505 | 0.005471729 |
| *CD276* | CD276 antigen | http://www.genecards.org/cgi-bin/carddisp.pl?gene=CD276 | 4.215273871 | 0.047359162 |
| *CD58* | Lymphocyte function-associated antigen 3 | http://www.genecards.org/cgi-bin/carddisp.pl?gene=CD58 | 1.348395012 | 0.017617783 |
| *CD81* | CD81 antigen | http://www.genecards.org/cgi-bin/carddisp.pl?gene=CD81 | 1.330959371 | 0.032179248 |
| *CDC42EP3* | Cdc42 effector protein 3 | http://www.genecards.org/cgi-bin/carddisp.pl?gene=CDC42EP3 | 3.966930719 | 0.009820891 |
| *CDH13* | Cadherin-13 | http://www.genecards.org/cgi-bin/carddisp.pl?gene=CDH13 | 1.315284396 | 0.037726141 |
| *CDH2* | Cadherin-2 | http://www.genecards.org/cgi-bin/carddisp.pl?gene=CDH2 | 1.754483402 | 0.02233838 |
| *CDH23* | Cadherin-23 | http://www.genecards.org/cgi-bin/carddisp.pl?gene=CDH23 | 5.839904725 | 0.020115708 |
| *CEP89* | Centrosomal protein of 89 kDa | http://www.genecards.org/cgi-bin/carddisp.pl?gene=CEP89 | 2.952906775 | 0.007117787 |
| *CHD7* | Chromodomain-helicase-DNA-binding protein 7 | http://www.genecards.org/cgi-bin/carddisp.pl?gene=CHD7 | 2.032160618 | 0.01499475 |
| *CHMP6* | Charged multivesicular body protein 6 | http://www.genecards.org/cgi-bin/carddisp.pl?gene=CHMP6 | 3.280638185 | 0.006173398 |
| *CLDND1* | Claudin domain-containing protein 1 | http://www.genecards.org/cgi-bin/carddisp.pl?gene=CLDND1 | 1.722663658 | 0.039818634 |
| *CLIP1* | CAP-Gly domain-containing linker protein 1 | http://www.genecards.org/cgi-bin/carddisp.pl?gene=CLIP1 | 1.426302111 | 0.018074799 |
| *CLTC* | Clathrin heavy chain 1 | http://www.genecards.org/cgi-bin/carddisp.pl?gene=CLTC | 1.120331377 | 0.048040549 |
| *CNN2* | Calponin-2 | http://www.genecards.org/cgi-bin/carddisp.pl?gene=CNN2 | 1.64469827 | 0.019293667 |
| *CNN3* | Calponin-3 | http://www.genecards.org/cgi-bin/carddisp.pl?gene=CNN3 | 2.679921899 | 0.048083816 |
| *COL4A5* | Collagen alpha-5(IV) chain | http://www.genecards.org/cgi-bin/carddisp.pl?gene=COL4A5 | 2.163521035 | 0.000176275 |
| *COL8A1* | Collagen alpha-1(VIII) chain | http://www.genecards.org/cgi-bin/carddisp.pl?gene=COL8A1 | 1.287087569 | 0.004416661 |
| *COQ6* | Ubiquinone biosynthesis monooxygenase COQ6, mitochondrial | http://www.genecards.org/cgi-bin/carddisp.pl?gene=COQ6 | 1.573193589 | 0.007629539 |
| *CORO1B* | Coronin-1B | http://www.genecards.org/cgi-bin/carddisp.pl?gene=CORO1B | 1.465408928 | 0.006308387 |
| *CORO1C* | Coronin-1C | http://www.genecards.org/cgi-bin/carddisp.pl?gene=CORO1C | 2.302810659 | 0.013042146 |
| *CRK* | Adapter molecule crk | http://www.genecards.org/cgi-bin/carddisp.pl?gene=CRK | 2.746409705 | 0.003202728 |
| *CRKL* | Crk-like protein | http://www.genecards.org/cgi-bin/carddisp.pl?gene=CRKL | 2.502390693 | 0.044619311 |
| *CSK* | Tyrosine-protein kinase CSK | http://www.genecards.org/cgi-bin/carddisp.pl?gene=CSK | 1.649108677 | 0.000977149 |
| *CSPG4* | Chondroitin sulfate proteoglycan 4 | http://www.genecards.org/cgi-bin/carddisp.pl?gene=CSPG4 | 3.813067687 | 0.000173501 |
| *CTNNA1* | Catenin alpha-1 | http://www.genecards.org/cgi-bin/carddisp.pl?gene=CTNNA1 | 1.722579337 | 0.001962219 |
| *CTNND1* | Catenin delta-1 | http://www.genecards.org/cgi-bin/carddisp.pl?gene=CTNND1 | 1.753926588 | 0.003165682 |
| *CTTN* | Src substrate cortactin | http://www.genecards.org/cgi-bin/carddisp.pl?gene=CTTN | 1.939040221 | 0.000192735 |
| *DAG1* | Dystroglycan | http://www.genecards.org/cgi-bin/carddisp.pl?gene=DAG1 | 1.966470586 | 0.027584066 |
| *DBN1* | Drebrin | http://www.genecards.org/cgi-bin/carddisp.pl?gene=DBN1 | 2.245453313 | 0.001135823 |
| *DBNL* | Drebrin-like protein | http://www.genecards.org/cgi-bin/carddisp.pl?gene=DBNL | 1.731156529 | 0.033058351 |
| *DCBLD2* | Discoidin, CUB and LCCL domain-containing protein 2 | http://www.genecards.org/cgi-bin/carddisp.pl?gene=DCBLD2 | 2.121321277 | 0.004380082 |
| *DHRS7C* | Dehydrogenase/reductase SDR family member 7C | http://www.genecards.org/cgi-bin/carddisp.pl?gene=DHRS7C | 1.585774659 | 0.046263351 |
| *DKK1* | Dickkopf-related protein 1 | http://www.genecards.org/cgi-bin/carddisp.pl?gene=DKK1 | 1.351393871 | 0.026130919 |
| *DLG1* | Disks large homolog 1 | http://www.genecards.org/cgi-bin/carddisp.pl?gene=DLG1 | 2.44587794 | 0.001057023 |
| *DNAH5* | Dynein heavy chain 5, axonemal | http://www.genecards.org/cgi-bin/carddisp.pl?gene=DNAH5 | 1.287131614 | 0.043312361 |
| *DNAH9* | Dynein heavy chain 9, axonemal | http://www.genecards.org/cgi-bin/carddisp.pl?gene=DNAH9 | 1.58108587 | 0.04264948 |
| *DOCK6* | Dedicator of cytokinesis protein 6 | http://www.genecards.org/cgi-bin/carddisp.pl?gene=DOCK6 | 2.604971285 | 0.043049247 |
| *DSG2* | Desmoglein-2 | http://www.genecards.org/cgi-bin/carddisp.pl?gene=DSG2 | 5.086254773 | 0.001623076 |
| *DSTN* | Destrin | http://www.genecards.org/cgi-bin/carddisp.pl?gene=DSTN | 1.250928343 | 0.00380274 |
| *DYNLL1* | Dynein light chain 1, cytoplasmic | http://www.genecards.org/cgi-bin/carddisp.pl?gene=DYNLL1 | 2.147918306 | 0.016765869 |
| *ECE1* | Endothelin-converting enzyme 1 | http://www.genecards.org/cgi-bin/carddisp.pl?gene=ECE1 | 3.116567478 | 0.015931499 |
| *EDEM3* | ER degradation-enhancing alpha-mannosidase-like protein 3 | http://www.genecards.org/cgi-bin/carddisp.pl?gene=EDEM3 | 2.60743843 | 0.001387435 |
| *EEF1G* | Elongation factor 1-gamma | http://www.genecards.org/cgi-bin/carddisp.pl?gene=EEF1G | 1.589816745 | 0.017457873 |
| *EFHD2* | EF-hand domain-containing protein D2 | http://www.genecards.org/cgi-bin/carddisp.pl?gene=EFHD2 | 2.081942933 | 0.01049888 |
| *EGFR* | Epidermal growth factor receptor | http://www.genecards.org/cgi-bin/carddisp.pl?gene=EGFR | 1.599433407 | 0.005815457 |
| *EHD4* | EH domain-containing protein 4 | http://www.genecards.org/cgi-bin/carddisp.pl?gene=EHD4 | 1.971011255 | 8.31593E-05 |
| *EIF4A1* | Eukaryotic initiation factor 4A-I | http://www.genecards.org/cgi-bin/carddisp.pl?gene=EIF4A1 | 1.794035573 | 0.027492032 |
| *EMB* | Embigin | http://www.genecards.org/cgi-bin/carddisp.pl?gene=EMB | 2.891976888 | 0.000900393 |
| *ENAH* | Protein enabled homolog | http://www.genecards.org/cgi-bin/carddisp.pl?gene=ENAH | 3.642670365 | 0.000402608 |
| *ENDOD1* | Endonuclease domain-containing 1 protein | http://www.genecards.org/cgi-bin/carddisp.pl?gene=ENDOD1 | 3.050531925 | 0.00345924 |
| *ENO1* | Alpha-enolase | http://www.genecards.org/cgi-bin/carddisp.pl?gene=ENO1 | 1.29471859 | 0.004218016 |
| *EPB41L2* | Band 4.1-like protein 2 | http://www.genecards.org/cgi-bin/carddisp.pl?gene=EPB41L2 | 1.777222747 | 0.001265363 |
| *EPHA2* | Ephrin type-A receptor 2 | http://www.genecards.org/cgi-bin/carddisp.pl?gene=EPHA2 | 1.924084746 | 0.001392304 |
| *EPHB2* | Ephrin type-B receptor 2 | http://www.genecards.org/cgi-bin/carddisp.pl?gene=EPHB2 | 4.02733231 | 2.50759E-05 |
| *EPN1* | Epsin-1 | http://www.genecards.org/cgi-bin/carddisp.pl?gene=EPN1 | 1.802220406 | 0.044280341 |
| *ERBIN* | Erbin | http://www.genecards.org/cgi-bin/carddisp.pl?gene=ERBIN | 1.715407382 | 0.018573859 |
| *ERLIN2* | Erlin-2 | http://www.genecards.org/cgi-bin/carddisp.pl?gene=ERLIN2 | 2.212313368 | 0.00139557 |
| *ERP29* | Endoplasmic reticulum resident protein 29 | http://www.genecards.org/cgi-bin/carddisp.pl?gene=ERP29 | 4.118421481 | 0.001135911 |
| *ESYT1* | Extended synaptotagmin-1 | http://www.genecards.org/cgi-bin/carddisp.pl?gene=ESYT1 | 2.261576985 | 0.001274513 |
| *EVA1A* | Protein eva-1 homolog A | http://www.genecards.org/cgi-bin/carddisp.pl?gene=EVA1A | 2.654696176 | 0.012858187 |
| *F11R* | Junctional adhesion molecule A | http://www.genecards.org/cgi-bin/carddisp.pl?gene=F11R | 16.40602556 | 0.021458295 |
| *F3* | Tissue factor | http://www.genecards.org/cgi-bin/carddisp.pl?gene=F3 | 2.918480727 | 4.80124E-05 |
| *FBLIM1* | Filamin-binding LIM protein 1 | http://www.genecards.org/cgi-bin/carddisp.pl?gene=FBLIM1 | 2.6099763 | 0.002662092 |
| *FERMT2* | Fermitin family homolog 2 | http://www.genecards.org/cgi-bin/carddisp.pl?gene=FERMT2 | 1.719673256 | 0.033143994 |
| *FHL2* | Four and a half LIM domains protein 2 | http://www.genecards.org/cgi-bin/carddisp.pl?gene=FHL2 | 2.565720774 | 5.90555E-05 |
| *FHL3* | Four and a half LIM domains protein 3 | http://www.genecards.org/cgi-bin/carddisp.pl?gene=FHL3 | 4.065415916 | 0.029247012 |
| *FLNA* | Filamin-A | http://www.genecards.org/cgi-bin/carddisp.pl?gene=FLNA | 2.06741471 | 0.004345154 |
| *FLNB* | Filamin-B | http://www.genecards.org/cgi-bin/carddisp.pl?gene=FLNB | 1.939252631 | 0.00344773 |
| *FLNC* | Filamin-C | http://www.genecards.org/cgi-bin/carddisp.pl?gene=FLNC | 2.476915082 | 0.000123823 |
| *FLOT1* | Flotillin-1 | http://www.genecards.org/cgi-bin/carddisp.pl?gene=FLOT1 | 1.809700782 | 0.011104049 |
| *FMNL3* | Formin-like protein 3 | http://www.genecards.org/cgi-bin/carddisp.pl?gene=FMNL3 | 5.742228477 | 0.01913791 |
| *FN1* | Fibronectin | http://www.genecards.org/cgi-bin/carddisp.pl?gene=FN1 | 1.257681121 | 0.029317565 |
| *FSCN1* | Fascin | http://www.genecards.org/cgi-bin/carddisp.pl?gene=FSCN1 | 2.014799899 | 0.000946566 |
| *FXR1* | Fragile X mental retardation syndrome-related protein 1 | http://www.genecards.org/cgi-bin/carddisp.pl?gene=FXR1 | 2.295853323 | 0.003429578 |
| *FZD7* | Frizzled-7 | http://www.genecards.org/cgi-bin/carddisp.pl?gene=FZD7 | 1.275273215 | 0.037691513 |
| *GANAB* | Neutral alpha-glucosidase AB | http://www.genecards.org/cgi-bin/carddisp.pl?gene=GANAB | 6.759312245 | 0.024795758 |
| *GGCT* | Gamma-glutamylcyclotransferase | http://www.genecards.org/cgi-bin/carddisp.pl?gene=GGCT | 1.653606602 | 0.011259187 |
| *GLS* | Glutaminase kidney isoform, mitochondrial | http://www.genecards.org/cgi-bin/carddisp.pl?gene=GLS | 5.89954355 | 0.043307739 |
| *GLYR1* | Putative oxidoreductase GLYR1 | http://www.genecards.org/cgi-bin/carddisp.pl?gene=GLYR1 | 2.910626216 | 0.00207375 |
| *GNA13* | Guanine nucleotide-binding protein subunit alpha-13 | http://www.genecards.org/cgi-bin/carddisp.pl?gene=GNA13 | 3.958239288 | 0.001279427 |
| *GNAI2* | Guanine nucleotide-binding protein G(i) subunit alpha-2 | http://www.genecards.org/cgi-bin/carddisp.pl?gene=GNAI2 | 1.7951701 | 0.021511528 |
| *GNAI3* | Guanine nucleotide-binding protein G(k) subunit alpha | http://www.genecards.org/cgi-bin/carddisp.pl?gene=GNAI3 | 4.102652962 | 0.006471223 |
| *GNAQ* | Guanine nucleotide-binding protein G(q) subunit alpha | http://www.genecards.org/cgi-bin/carddisp.pl?gene=GNAQ | 1.329917843 | 0.009318904 |
| *GNB1* | Guanine nucleotide-binding protein G(I)/G(S)/G(T) subunit beta-1 | http://www.genecards.org/cgi-bin/carddisp.pl?gene=GNB1 | 2.219438808 | 0.007906828 |
| *GNB2* | Guanine nucleotide-binding protein G(I)/G(S)/G(T) subunit beta-2 | http://www.genecards.org/cgi-bin/carddisp.pl?gene=GNB2 | 1.841512541 | 0.022445275 |
| *GNB4* | Guanine nucleotide-binding protein subunit beta-4 | http://www.genecards.org/cgi-bin/carddisp.pl?gene=GNB4 | 3.441815104 | 0.024242408 |
| *GNG12* | Guanine nucleotide-binding protein G(I)/G(S)/G(O) subunit gamma-12 | http://www.genecards.org/cgi-bin/carddisp.pl?gene=GNG12 | 1.405863331 | 0.003934676 |
| *GOLGA7* | Golgin subfamily A member 7 | http://www.genecards.org/cgi-bin/carddisp.pl?gene=GOLGA7 | 1.784210108 | 0.000599112 |
| *GPC1* | Glypican-1 | http://www.genecards.org/cgi-bin/carddisp.pl?gene=GPC1 | 3.550297581 | 0.004583328 |
| *GPD2* | Glycerol-3-phosphate dehydrogenase, mitochondrial | http://www.genecards.org/cgi-bin/carddisp.pl?gene=GPD2 | 1.360278987 | 0.002626611 |
| *GPR176* | Probable G-protein coupled receptor 176 | http://www.genecards.org/cgi-bin/carddisp.pl?gene=GPR176 | 3.240086081 | 3.6128E-05 |
| *GRM4* | Metabotropic glutamate receptor 4 | http://www.genecards.org/cgi-bin/carddisp.pl?gene=GRM4 | only present in RPEp53-/- FA | 0.014216115 |
| *HIP1R* | Huntingtin-interacting protein 1-related protein | http://www.genecards.org/cgi-bin/carddisp.pl?gene=HIP1R | 1.426786283 | 0.031913653 |
| *HIST1H1B* | Histone H1.5 | http://www.genecards.org/cgi-bin/carddisp.pl?gene=HIST1H1B | 1.897836209 | 0.044226755 |
| *HLA-B* | HLA class I histocompatibility antigen, B-48 alpha chain | http://www.genecards.org/cgi-bin/carddisp.pl?gene=HLA-B | 5.326577722 | 0.026365091 |
| *HRG* | Histidine-rich glycoprotein | http://www.genecards.org/cgi-bin/carddisp.pl?gene=HRG | 1.492535738 | 0.006187331 |
| *HSP90B1* | Endoplasmin | http://www.genecards.org/cgi-bin/carddisp.pl?gene=HSP90B1 | 2.0840723 | 0.008064203 |
| *HSPA5* | 78 kDa glucose-regulated protein | http://www.genecards.org/cgi-bin/carddisp.pl?gene=HSPA5 | 2.278829677 | 0.000511894 |
| *IGF2R* | Cation-independent mannose-6-phosphate receptor | http://www.genecards.org/cgi-bin/carddisp.pl?gene=IGF2R | 1.702122535 | 2.54771E-05 |
| *IGFBP3* | Insulin-like growth factor-binding protein 3 | http://www.genecards.org/cgi-bin/carddisp.pl?gene=IGFBP3 | 1.379079471 | 0.016298311 |
| *IGFBP5* | Insulin-like growth factor-binding protein 5 | http://www.genecards.org/cgi-bin/carddisp.pl?gene=IGFBP5 | 1.781896175 | 0.005017871 |
| *ILK* | Integrin-linked protein kinase | http://www.genecards.org/cgi-bin/carddisp.pl?gene=ILK | 1.380558593 | 0.007274318 |
| *ITGA3* | Integrin alpha-3 | http://www.genecards.org/cgi-bin/carddisp.pl?gene=ITGA3 | 1.845787259 | 0.00631471 |
| *ITGA5* | Integrin alpha-5 | http://www.genecards.org/cgi-bin/carddisp.pl?gene=ITGA5 | 1.245344455 | 0.005075457 |
| *ITGAV* | Integrin alpha-V | http://www.genecards.org/cgi-bin/carddisp.pl?gene=ITGAV | 1.554123838 | 0.025624024 |
| *ITGB1* | Integrin beta-1 | http://www.genecards.org/cgi-bin/carddisp.pl?gene=ITGB1 | 1.153431105 | 0.008372463 |
| *ITGB5* | Integrin beta-5 | http://www.genecards.org/cgi-bin/carddisp.pl?gene=ITGB5 | 1.37473289 | 0.046641574 |
| *KANK2* | KN motif and ankyrin repeat domain-containing protein 2 | http://www.genecards.org/cgi-bin/carddisp.pl?gene=KANK2 | 1.507192549 | 0.018678527 |
| *KLHL17* | Kelch-like protein 17 | http://www.genecards.org/cgi-bin/carddisp.pl?gene=KLHL17 | 2.586243171 | 0.000818025 |
| *KPNA2* | Importin subunit alpha-1 | http://www.genecards.org/cgi-bin/carddisp.pl?gene=KPNA2 | 1.808298895 | 0.000907248 |
| *KRT18* | Keratin, type I cytoskeletal 18 | http://www.genecards.org/cgi-bin/carddisp.pl?gene=KRT18 | 2.875615798 | 0.004945234 |
| *KRT3* | Keratin, type II cytoskeletal 3 | http://www.genecards.org/cgi-bin/carddisp.pl?gene=KRT3 | 1.71521472 | 0.024214933 |
| *KRT71* | Keratin, type II cytoskeletal 71 | http://www.genecards.org/cgi-bin/carddisp.pl?gene=KRT71 | 1.897423775 | 0.040216601 |
| *KRT8* | Keratin, type II cytoskeletal 8 | http://www.genecards.org/cgi-bin/carddisp.pl?gene=KRT8 | 2.853640808 | 0.000726777 |
| *KRT80* | Keratin, type II cytoskeletal 80 | http://www.genecards.org/cgi-bin/carddisp.pl?gene=KRT80 | 2.547636697 | 0.026911355 |
| *LAMB1* | Laminin subunit beta-1 | http://www.genecards.org/cgi-bin/carddisp.pl?gene=LAMB1 | 1.712029471 | 0.017549359 |
| *LANCL1* | LanC-like protein 1 | http://www.genecards.org/cgi-bin/carddisp.pl?gene=LANCL1 | 1.78602898 | 0.010723641 |
| *LASP1* | LIM and SH3 domain protein 1 | http://www.genecards.org/cgi-bin/carddisp.pl?gene=LASP1 | 1.642970594 | 3.71379E-05 |
| *LATS1* | Serine/threonine-protein kinase LATS1 | http://www.genecards.org/cgi-bin/carddisp.pl?gene=LATS1 | 3.095900142 | 0.036960212 |
| *LGI1* | Leucine-rich glioma-inactivated protein 1 | http://www.genecards.org/cgi-bin/carddisp.pl?gene=LGI1 | 2.662553717 | 0.012252359 |
| *LIMA1* | LIM domain and actin-binding protein 1 | http://www.genecards.org/cgi-bin/carddisp.pl?gene=LIMA1 | 2.132722803 | 0.018903292 |
| *LIMCH1* | LIM and calponin homology domains-containing protein 1 | http://www.genecards.org/cgi-bin/carddisp.pl?gene=LIMCH1 | 7.439789638 | 0.000275452 |
| *LIN7C* | Protein lin-7 homolog C | http://www.genecards.org/cgi-bin/carddisp.pl?gene=LIN7C | 1.561598594 | 0.023439894 |
| *LMO7* | LIM domain only protein 7 | http://www.genecards.org/cgi-bin/carddisp.pl?gene=LMO7 | 2.028062995 | 0.007418388 |
| *LPP* | Lipoma-preferred partner | http://www.genecards.org/cgi-bin/carddisp.pl?gene=LPP | 1.942388338 | 0.037197305 |
| *LRRC8A* | Volume-regulated anion channel subunit LRRC8A | http://www.genecards.org/cgi-bin/carddisp.pl?gene=LRRC8A | 3.45584086 | 0.005610096 |
| *LTBP4* | Latent-transforming growth factor beta-binding protein 4 | http://www.genecards.org/cgi-bin/carddisp.pl?gene=LTBP4 | 1.339735085 | 0.00210046 |
| *MAGI3* | Membrane-associated guanylate kinase, WW and PDZ domain-containing protein 3 | http://www.genecards.org/cgi-bin/carddisp.pl?gene=MAGI3 | 3.365837057 | 0.029804142 |
| *MET* | Hepatocyte growth factor receptor | http://www.genecards.org/cgi-bin/carddisp.pl?gene=MET | 1.581891595 | 0.010114226 |
| *MME* | Neprilysin | http://www.genecards.org/cgi-bin/carddisp.pl?gene=MME | 2.373640271 | 0.012607067 |
| *MPP6* | MAGUK p55 subfamily member 6 | http://www.genecards.org/cgi-bin/carddisp.pl?gene=MPP6 | 2.816260016 | 2.37836E-05 |
| *MPZL1* | Myelin protein zero-like protein 1 | http://www.genecards.org/cgi-bin/carddisp.pl?gene=MPZL1 | 2.487195737 | 0.008135279 |
| *MRC2* | C-type mannose receptor 2 | http://www.genecards.org/cgi-bin/carddisp.pl?gene=MRC2 | 1.808109092 | 0.006909091 |
| *MUC20* | Mucin-20 | http://www.genecards.org/cgi-bin/carddisp.pl?gene=MUC20 | 3.760348365 | 0.046866327 |
| *MXRA8* | Matrix-remodeling-associated protein 8 | http://www.genecards.org/cgi-bin/carddisp.pl?gene=MXRA8 | 2.093839961 | 0.019678657 |
| *MYH10* | Myosin-10 | http://www.genecards.org/cgi-bin/carddisp.pl?gene=MYH10 | 4.229368119 | 0.001335927 |
| *MYH9* | Myosin-9 | http://www.genecards.org/cgi-bin/carddisp.pl?gene=MYH9 | 2.118937083 | 0.001692486 |
| *MYL12A* | Myosin regulatory light chain 12A | http://www.genecards.org/cgi-bin/carddisp.pl?gene=MYL12A | 2.60274976 | 1.22698E-05 |
| *MYL6* | Myosin light polypeptide 6 | http://www.genecards.org/cgi-bin/carddisp.pl?gene=MYL6 | 1.959901656 | 0.000262886 |
| *MYL9* | Myosin regulatory light polypeptide 9 | http://www.genecards.org/cgi-bin/carddisp.pl?gene=MYL9 | 1.552492245 | 0.004673703 |
| *MYLK* | Myosin light chain kinase, smooth muscle | http://www.genecards.org/cgi-bin/carddisp.pl?gene=MYLK | 2.202063097 | 0.008685169 |
| *MYO1C* | Unconventional myosin-Ic | http://www.genecards.org/cgi-bin/carddisp.pl?gene=MYO1C | 2.674853241 | 0.003146741 |
| *MYOF* | Myoferlin | http://www.genecards.org/cgi-bin/carddisp.pl?gene=MYOF | 1.997677103 | 9.02945E-05 |
| *NCEH1* | Neutral cholesterol ester hydrolase 1 | http://www.genecards.org/cgi-bin/carddisp.pl?gene=NCEH1 | 1.570317512 | 0.047080526 |
| *NCK1* | Cytoplasmic protein NCK1 | http://www.genecards.org/cgi-bin/carddisp.pl?gene=NCK1 | 4.771667361 | 0.02034128 |
| *NECTIN2* | Nectin-2 | http://www.genecards.org/cgi-bin/carddisp.pl?gene=NECTIN2 | 1.572929258 | 0.027866309 |
| *NFRKB* | Nuclear factor related to kappa-B-binding protein | http://www.genecards.org/cgi-bin/carddisp.pl?gene=NFRKB | 2.457134673 | 0.049923368 |
| *NGF* | Beta-nerve growth factor | http://www.genecards.org/cgi-bin/carddisp.pl?gene=NGF | 2.684156642 | 0.001864151 |
| *NLN* | Neurolysin, mitochondrial | http://www.genecards.org/cgi-bin/carddisp.pl?gene=NLN | 2.519769851 | 0.000442969 |
| *NME1* | Nucleoside diphosphate kinase A | http://www.genecards.org/cgi-bin/carddisp.pl?gene=NME1 | 2.367837138 | 0.030200654 |
| *NPTN* | Neuroplastin | http://www.genecards.org/cgi-bin/carddisp.pl?gene=NPTN | 1.861846232 | 0.007850759 |
| *NUMBL* | Numb-like protein | http://www.genecards.org/cgi-bin/carddisp.pl?gene=NUMBL | 1.250196229 | 0.017634035 |
| *OSBPL8* | Oxysterol-binding protein-related protein 8 | http://www.genecards.org/cgi-bin/carddisp.pl?gene=OSBPL8 | 2.612924383 | 0.000128056 |
| *PABPC1* | Polyadenylate-binding protein 1 | http://www.genecards.org/cgi-bin/carddisp.pl?gene=PABPC1 | 2.271304615 | 0.034495082 |
| *PABPC4* | Polyadenylate-binding protein 4 | http://www.genecards.org/cgi-bin/carddisp.pl?gene=PABPC4 | 3.672779391 | 0.033819185 |
| *PALLD* | Palladin | http://www.genecards.org/cgi-bin/carddisp.pl?gene=PALLD | 2.71016339 | 0.000521177 |
| *PARN* | Poly(A)-specific ribonuclease PARN | http://www.genecards.org/cgi-bin/carddisp.pl?gene=PARN | 2.457468768 | 0.028884379 |
| *PAWR* | PRKC apoptosis WT1 regulator protein | http://www.genecards.org/cgi-bin/carddisp.pl?gene=PAWR | 2.755365031 | 0.003248487 |
| *PCBP2* | Poly(rC)-binding protein 2 | http://www.genecards.org/cgi-bin/carddisp.pl?gene=PCBP2 | 1.767720623 | 0.005974914 |
| *PCDH15* | Protocadherin-15 | http://www.genecards.org/cgi-bin/carddisp.pl?gene=PCDH15 | 2.596755943 | 0.012707476 |
| *PCSK9* | Proprotein convertase subtilisin/kexin type 9 | http://www.genecards.org/cgi-bin/carddisp.pl?gene=PCSK9 | 1.419532303 | 0.007790704 |
| *PDIA6* | Protein disulfide-isomerase A6 | http://www.genecards.org/cgi-bin/carddisp.pl?gene=PDIA6 | 1.909944985 | 0.0027342 |
| *PDLIM2* | PDZ and LIM domain protein 2 | http://www.genecards.org/cgi-bin/carddisp.pl?gene=PDLIM2 | 7.134297996 | 0.000125984 |
| *PDLIM4* | PDZ and LIM domain protein 4 | http://www.genecards.org/cgi-bin/carddisp.pl?gene=PDLIM4 | 2.556282015 | 0.000389777 |
| *PDLIM5* | PDZ and LIM domain protein 5 | http://www.genecards.org/cgi-bin/carddisp.pl?gene=PDLIM5 | 2.666275053 | 0.004730218 |
| *PDLIM7* | PDZ and LIM domain protein 7 | http://www.genecards.org/cgi-bin/carddisp.pl?gene=PDLIM7 | 2.525301159 | 0.001130396 |
| *PDZRN3* | E3 ubiquitin-protein ligase PDZRN3 | http://www.genecards.org/cgi-bin/carddisp.pl?gene=PDZRN3 | 1.904159444 | 0.013631097 |
| *PFN1* | Profilin-1 | http://www.genecards.org/cgi-bin/carddisp.pl?gene=PFN1 | 1.454312304 | 0.039438826 |
| *PGRMC1* | Membrane-associated progesterone receptor component 1 | http://www.genecards.org/cgi-bin/carddisp.pl?gene=PGRMC1 | 7.137656229 | 0.009200315 |
| *PHLDA1* | Pleckstrin homology-like domain family A member 1 | http://www.genecards.org/cgi-bin/carddisp.pl?gene=PHLDA1 | 1.837188834 | 0.026372012 |
| *PLAG1* | Zinc finger protein PLAG1 | http://www.genecards.org/cgi-bin/carddisp.pl?gene=PLAG1 | 2.874307684 | 0.006508816 |
| *PLEC* | Plectin | http://www.genecards.org/cgi-bin/carddisp.pl?gene=PLEC | 1.958972745 | 0.007587025 |
| *PLS3* | Plastin-3 | http://www.genecards.org/cgi-bin/carddisp.pl?gene=PLS3 | 1.862172766 | 0.00163616 |
| *PLXNA3* | Plexin-A3 | http://www.genecards.org/cgi-bin/carddisp.pl?gene=PLXNA3 | 2.25573468 | 0.007502109 |
| *PLXNB2* | Plexin-B2 | http://www.genecards.org/cgi-bin/carddisp.pl?gene=PLXNB2 | 2.465175674 | 3.77578E-05 |
| *PPFIA1* | Liprin-alpha-1 | http://www.genecards.org/cgi-bin/carddisp.pl?gene=PPFIA1 | 2.083028056 | 0.008087461 |
| *PPFIA3* | Liprin-alpha-3 | http://www.genecards.org/cgi-bin/carddisp.pl?gene=PPFIA3 | 1.215999619 | 0.025632705 |
| *PPIB* | Peptidyl-prolyl cis-trans isomerase B | http://www.genecards.org/cgi-bin/carddisp.pl?gene=PPIB | 2.11604795 | 0.03034735 |
| *PPP1CC* | Serine/threonine-protein phosphatase PP1-gamma catalytic subunit | http://www.genecards.org/cgi-bin/carddisp.pl?gene=PPP1CC | 1.784214332 | 0.014507873 |
| *PPP1R12A* | Protein phosphatase 1 regulatory subunit 12A | http://www.genecards.org/cgi-bin/carddisp.pl?gene=PPP1R12A | 1.656427175 | 0.007289139 |
| *PPP1R18* | Phostensin | http://www.genecards.org/cgi-bin/carddisp.pl?gene=PPP1R18 | 1.645748462 | 0.000198248 |
| *PPP2R3A* | Serine/threonine-protein phosphatase 2A regulatory subunit B'' subunit alpha | http://www.genecards.org/cgi-bin/carddisp.pl?gene=PPP2R3A | 2.552167477 | 0.003398361 |
| *PRDX1* | Peroxiredoxin-1 | http://www.genecards.org/cgi-bin/carddisp.pl?gene=PRDX1 | 2.31552862 | 1.64737E-05 |
| *PRDX6* | Peroxiredoxin-6 | http://www.genecards.org/cgi-bin/carddisp.pl?gene=PRDX6 | 1.497828402 | 0.02986343 |
| *PRNP* | Major prion protein | http://www.genecards.org/cgi-bin/carddisp.pl?gene=PRNP | 1.431893622 | 0.006474152 |
| *PROCR* | Endothelial protein C receptor | http://www.genecards.org/cgi-bin/carddisp.pl?gene=PROCR | 4.318874174 | 0.003625085 |
| *PSMA3* | Proteasome subunit alpha type-3 | http://www.genecards.org/cgi-bin/carddisp.pl?gene=PSMA3 | 20.66497501 | 0.041972464 |
| *PTK2* | Focal adhesion kinase 1 | http://www.genecards.org/cgi-bin/carddisp.pl?gene=PTK2 | 2.782895636 | 0.001051542 |
| *PTK7* | Inactive tyrosine-protein kinase 7 | http://www.genecards.org/cgi-bin/carddisp.pl?gene=PTK7 | 3.23424987 | 0.002717152 |
| *PTRF* | Polymerase I and transcript release factor | http://www.genecards.org/cgi-bin/carddisp.pl?gene=PTRF | 2.642121942 | 0.03385149 |
| *PXN* | Paxillin | http://www.genecards.org/cgi-bin/carddisp.pl?gene=PXN | 2.187278939 | 0.006349484 |
| *RAB10* | Ras-related protein Rab-10 | http://www.genecards.org/cgi-bin/carddisp.pl?gene=RAB10 | 2.159309647 | 0.005049351 |
| *RAB11A* | Ras-related protein Rab-11A | http://www.genecards.org/cgi-bin/carddisp.pl?gene=RAB11A | 2.029679327 | 0.018352832 |
| *RAB34* | Ras-related protein Rab-34 | http://www.genecards.org/cgi-bin/carddisp.pl?gene=RAB34 | 2.380645729 | 0.001531135 |
| *RAB7A* | Ras-related protein Rab-7a | http://www.genecards.org/cgi-bin/carddisp.pl?gene=RAB7A | 1.583076996 | 0.010117935 |
| *RAC1* | Ras-related C3 botulinum toxin substrate 1 | http://www.genecards.org/cgi-bin/carddisp.pl?gene=RAC1 | 1.570802368 | 0.029483803 |
| *RAP1A* | Ras-related protein Rap-1A | http://www.genecards.org/cgi-bin/carddisp.pl?gene=RAP1A | 2.113475821 | 0.001034782 |
| *RAP1B* | Ras-related protein Rap-1b | http://www.genecards.org/cgi-bin/carddisp.pl?gene=RAP1B | 1.490968335 | 0.043283148 |
| *RASA3* | Ras GTPase-activating protein 3 | http://www.genecards.org/cgi-bin/carddisp.pl?gene=RASA3 | 2.523397857 | 0.006636709 |
| *RFLNB* | Refilin-B | http://www.genecards.org/cgi-bin/carddisp.pl?gene=RFLNB | 9.853734056 | 0.000372208 |
| *RNH1* | Ribonuclease inhibitor | http://www.genecards.org/cgi-bin/carddisp.pl?gene=RNH1 | 2.055515685 | 0.00444143 |
| *RPL10* | 60S ribosomal protein L10 | http://www.genecards.org/cgi-bin/carddisp.pl?gene=RPL10 | 1.689979092 | 0.017634035 |
| *RPL11* | 60S ribosomal protein L11 | http://www.genecards.org/cgi-bin/carddisp.pl?gene=RPL11 | 1.772226122 | 0.012359534 |
| *RPL12* | 60S ribosomal protein L12 | http://www.genecards.org/cgi-bin/carddisp.pl?gene=RPL12 | 1.662646467 | 0.000696725 |
| *RPL13* | 60S ribosomal protein L13 | http://www.genecards.org/cgi-bin/carddisp.pl?gene=RPL13 | 1.342498023 | 0.020688438 |
| *RPL14* | 60S ribosomal protein L14 | http://www.genecards.org/cgi-bin/carddisp.pl?gene=RPL14 | 1.692742096 | 0.001286069 |
| *RPL18* | 60S ribosomal protein L18 | http://www.genecards.org/cgi-bin/carddisp.pl?gene=RPL18 | 1.962139518 | 0.005468053 |
| *RPL19* | 60S ribosomal protein L19 | http://www.genecards.org/cgi-bin/carddisp.pl?gene=RPL19 | 1.695627053 | 0.008986871 |
| *RPL21* | 60S ribosomal protein L21 | http://www.genecards.org/cgi-bin/carddisp.pl?gene=RPL21 | 2.344506791 | 0.017401778 |
| *RPL22* | 60S ribosomal protein L22 | http://www.genecards.org/cgi-bin/carddisp.pl?gene=RPL22 | 1.834460704 | 0.002124936 |
| *RPL23* | 60S ribosomal protein L23 | http://www.genecards.org/cgi-bin/carddisp.pl?gene=RPL23 | 2.5031528 | 0.010891132 |
| *RPL26* | 60S ribosomal protein L26 | http://www.genecards.org/cgi-bin/carddisp.pl?gene=RPL26 | 2.023180997 | 0.000915254 |
| *RPL29* | 60S ribosomal protein L29 | http://www.genecards.org/cgi-bin/carddisp.pl?gene=RPL29 | 1.730122226 | 0.006638381 |
| *RPL30* | 60S ribosomal protein L30 | http://www.genecards.org/cgi-bin/carddisp.pl?gene=RPL30 | 1.495503616 | 0.034403589 |
| *RPL37A* | 60S ribosomal protein L37a | http://www.genecards.org/cgi-bin/carddisp.pl?gene=RPL37A | 3.924287768 | 0.008428622 |
| *RPL38* | 60S ribosomal protein L38 | http://www.genecards.org/cgi-bin/carddisp.pl?gene=RPL38 | 3.221075662 | 0.014224959 |
| *RPL4* | 60S ribosomal protein L4 | http://www.genecards.org/cgi-bin/carddisp.pl?gene=RPL4 | 1.298080672 | 0.015717063 |
| *RPL5* | 60S ribosomal protein L5 | http://www.genecards.org/cgi-bin/carddisp.pl?gene=RPL5 | 1.617150809 | 0.000543485 |
| *RPL6* | 60S ribosomal protein L6 | http://www.genecards.org/cgi-bin/carddisp.pl?gene=RPL6 | 1.868808922 | 0.005261739 |
| *RPL7* | 60S ribosomal protein L7 | http://www.genecards.org/cgi-bin/carddisp.pl?gene=RPL7 | 2.195923096 | 0.018564359 |
| *RPL7A* | 60S ribosomal protein L7a | http://www.genecards.org/cgi-bin/carddisp.pl?gene=RPL7A | 1.434959201 | 0.008827324 |
| *RPL8* | 60S ribosomal protein L8 | http://www.genecards.org/cgi-bin/carddisp.pl?gene=RPL8 | 1.355845734 | 0.00048051 |
| *RPLP0* | 60S acidic ribosomal protein P0 | http://www.genecards.org/cgi-bin/carddisp.pl?gene=RPLP0 | 2.018016237 | 0.004166 |
| *RPN1* | Dolichyl-diphosphooligosaccharide--protein glycosyltransferase subunit 1 | http://www.genecards.org/cgi-bin/carddisp.pl?gene=RPN1 | 1.662004278 | 0.008476224 |
| *RPS10* | 40S ribosomal protein S10 | http://www.genecards.org/cgi-bin/carddisp.pl?gene=RPS10 | 2.32917446 | 0.000125627 |
| *RPS11* | 40S ribosomal protein S11 | http://www.genecards.org/cgi-bin/carddisp.pl?gene=RPS11 | 1.394036138 | 0.000315037 |
| *RPS12* | 40S ribosomal protein S12 | http://www.genecards.org/cgi-bin/carddisp.pl?gene=RPS12 | 1.854003505 | 0.00157634 |
| *RPS13* | 40S ribosomal protein S13 | http://www.genecards.org/cgi-bin/carddisp.pl?gene=RPS13 | 1.50837622 | 0.001418314 |
| *RPS14* | 40S ribosomal protein S14 | http://www.genecards.org/cgi-bin/carddisp.pl?gene=RPS14 | 2.426507399 | 0.003054072 |
| *RPS15A* | 40S ribosomal protein S15a | http://www.genecards.org/cgi-bin/carddisp.pl?gene=RPS15A | 4.984472333 | 0.049948777 |
| *RPS16* | 40S ribosomal protein S16 | http://www.genecards.org/cgi-bin/carddisp.pl?gene=RPS16 | 1.899878573 | 0.0188466 |
| *RPS18* | 40S ribosomal protein S18 | http://www.genecards.org/cgi-bin/carddisp.pl?gene=RPS18 | 1.152956135 | 0.016660025 |
| *RPS19* | 40S ribosomal protein S19 | http://www.genecards.org/cgi-bin/carddisp.pl?gene=RPS19 | 2.283493228 | 0.001044002 |
| *RPS20* | 40S ribosomal protein S20 | http://www.genecards.org/cgi-bin/carddisp.pl?gene=RPS20 | 1.301774973 | 0.023201468 |
| *RPS23* | 40S ribosomal protein S23 | http://www.genecards.org/cgi-bin/carddisp.pl?gene=RPS23 | 2.421056305 | 0.040248217 |
| *RPS27L* | 40S ribosomal protein S27-like | http://www.genecards.org/cgi-bin/carddisp.pl?gene=RPS27L | 1.447348256 | 0.043441187 |
| *RPS29* | 40S ribosomal protein S29 | http://www.genecards.org/cgi-bin/carddisp.pl?gene=RPS29 | 2.713034451 | 0.020107906 |
| *RPS5* | 40S ribosomal protein S5 | http://www.genecards.org/cgi-bin/carddisp.pl?gene=RPS5 | 3.820210598 | 0.037226866 |
| *RPS6* | 40S ribosomal protein S6 | http://www.genecards.org/cgi-bin/carddisp.pl?gene=RPS6 | 1.516902528 | 0.016159846 |
| *RPS7* | 40S ribosomal protein S7 | http://www.genecards.org/cgi-bin/carddisp.pl?gene=RPS7 | 3.040494901 | 0.009201647 |
| *RPS8* | 40S ribosomal protein S8 | http://www.genecards.org/cgi-bin/carddisp.pl?gene=RPS8 | 1.556374338 | 0.038440083 |
| *RPS9* | 40S ribosomal protein S9 | http://www.genecards.org/cgi-bin/carddisp.pl?gene=RPS9 | 1.614294143 | 0.003936973 |
| *RPSA* | 40S ribosomal protein SA | http://www.genecards.org/cgi-bin/carddisp.pl?gene=RPSA | 1.18553499 | 0.038229019 |
| *RRAS2* | Ras-related protein R-Ras2 | http://www.genecards.org/cgi-bin/carddisp.pl?gene=RRAS2 | 1.733816667 | 0.018308813 |
| *RSU1* | Ras suppressor protein 1 | http://www.genecards.org/cgi-bin/carddisp.pl?gene=RSU1 | 1.509934919 | 0.042130708 |
| *RTN4* | Reticulon-4 | http://www.genecards.org/cgi-bin/carddisp.pl?gene=RTN4 | 2.055834349 | 0.000664414 |
| *S100A10* | Protein S100-A10 | http://www.genecards.org/cgi-bin/carddisp.pl?gene=S100A10 | 1.271557688 | 0.020179596 |
| *SCAMP3* | Secretory carrier-associated membrane protein 3 | http://www.genecards.org/cgi-bin/carddisp.pl?gene=SCAMP3 | 3.3422972 | 0.010059549 |
| *SDC1* | Syndecan-1 | http://www.genecards.org/cgi-bin/carddisp.pl?gene=SDC1 | 3.256378677 | 0.004955468 |
| *SDPR* | Serum deprivation-response protein | http://www.genecards.org/cgi-bin/carddisp.pl?gene=SDPR | 3.432479876 | 0.037290512 |
| *SEC22B* | Vesicle-trafficking protein SEC22b | http://www.genecards.org/cgi-bin/carddisp.pl?gene=SEC22B | 2.406523458 | 0.000396671 |
| *SEMA3B* | Semaphorin-3B | http://www.genecards.org/cgi-bin/carddisp.pl?gene=SEMA3B | 1.424470938 | 0.026954007 |
| *SEMA7A* | Semaphorin-7A | http://www.genecards.org/cgi-bin/carddisp.pl?gene=SEMA7A | 1.960168795 | 0.003073498 |
| *SEP11* | Septin-11 | http://www.genecards.org/cgi-bin/carddisp.pl?gene=SEP11 | 1.706045955 | 0.006025062 |
| *SEPT2* | Septin-2 | http://www.genecards.org/cgi-bin/carddisp.pl?gene=SEPT2 | 1.952119499 | 0.010698516 |
| *SEPT8* | Septin-8 | http://www.genecards.org/cgi-bin/carddisp.pl?gene=SEPT8 | 1.969926714 | 0.004544561 |
| *SERF2* | Small EDRK-rich factor 2 | http://www.genecards.org/cgi-bin/carddisp.pl?gene=SERF2 | 1.858534403 | 0.018430133 |
| *SERPINE1* | Plasminogen activator inhibitor 1 | http://www.genecards.org/cgi-bin/carddisp.pl?gene=SERPINE1 | 1.19952934 | 0.044711949 |
| *SH3BP4* | SH3 domain-binding protein 4 | http://www.genecards.org/cgi-bin/carddisp.pl?gene=SH3BP4 | 2.883603484 | 0.017604593 |
| *SKP1* | S-phase kinase-associated protein 1 | http://www.genecards.org/cgi-bin/carddisp.pl?gene=SKP1 | 1.962218819 | 0.001691172 |
| *SLC16A1* | Monocarboxylate transporter 1 | http://www.genecards.org/cgi-bin/carddisp.pl?gene=SLC16A1 | 2.293264414 | 0.000195507 |
| *SLC16A3* | Monocarboxylate transporter 4 | http://www.genecards.org/cgi-bin/carddisp.pl?gene=SLC16A3 | 1.729579503 | 0.026523297 |
| *SLC1A5* | Neutral amino acid transporter B(0) | http://www.genecards.org/cgi-bin/carddisp.pl?gene=SLC1A5 | 1.721460353 | 0.000218935 |
| *SLC2A1* | Solute carrier family 2, facilitated glucose transporter member 1 | http://www.genecards.org/cgi-bin/carddisp.pl?gene=SLC2A1 | 2.036697168 | 5.50811E-05 |
| *SLC38A2* | Sodium-coupled neutral amino acid transporter 2 | http://www.genecards.org/cgi-bin/carddisp.pl?gene=SLC38A2 | 1.917451739 | 0.034246785 |
| *SLC39A14* | Zinc transporter ZIP14 | http://www.genecards.org/cgi-bin/carddisp.pl?gene=SLC39A14 | 2.366647909 | 4.95057E-05 |
| *SLC3A2* | 4F2 cell-surface antigen heavy chain | http://www.genecards.org/cgi-bin/carddisp.pl?gene=SLC3A2 | 2.766242271 | 0.002868909 |
| *SLC44A1* | Choline transporter-like protein 1 | http://www.genecards.org/cgi-bin/carddisp.pl?gene=SLC44A1 | 2.755141122 | 0.001401569 |
| *SLC4A7* | Sodium bicarbonate cotransporter 3 | http://www.genecards.org/cgi-bin/carddisp.pl?gene=SLC4A7 | 5.344612689 | 0.007218589 |
| *SLC7A1* | High affinity cationic amino acid transporter 1 | http://www.genecards.org/cgi-bin/carddisp.pl?gene=SLC7A1 | 4.700500037 | 0.011187272 |
| *SLC7A5* | Large neutral amino acids transporter small subunit 1 | http://www.genecards.org/cgi-bin/carddisp.pl?gene=SLC7A5 | 1.801978236 | 0.008118386 |
| *SNAP23* | Synaptosomal-associated protein 23 | http://www.genecards.org/cgi-bin/carddisp.pl?gene=SNAP23 | 1.499301365 | 0.002815261 |
| *SORT1* | Sortilin | http://www.genecards.org/cgi-bin/carddisp.pl?gene=SORT1 | 4.891188647 | 0.001870707 |
| *SPTBN1* | Spectrin beta chain, non-erythrocytic 1 | http://www.genecards.org/cgi-bin/carddisp.pl?gene=SPTBN1 | 1.71299934 | 0.003733714 |
| *STEAP3* | Metalloreductase STEAP3 | http://www.genecards.org/cgi-bin/carddisp.pl?gene=STEAP3 | 2.728969617 | 9.23863E-06 |
| *STX4* | Syntaxin-4 | http://www.genecards.org/cgi-bin/carddisp.pl?gene=STX4 | 1.637363962 | 0.006769852 |
| *STX7* | Syntaxin-7 | http://www.genecards.org/cgi-bin/carddisp.pl?gene=STX7 | 1.320612574 | 0.009254618 |
| *TAGLN3* | Transgelin-3 | http://www.genecards.org/cgi-bin/carddisp.pl?gene=TAGLN3 | 3.945778166 | 0.03323048 |
| *TFRC* | Transferrin receptor protein 1 | http://www.genecards.org/cgi-bin/carddisp.pl?gene=TFRC | 1.905635989 | 0.000965719 |
| *TGFB1I1* | Transforming growth factor beta-1-induced transcript 1 protein | http://www.genecards.org/cgi-bin/carddisp.pl?gene=TGFB1I1 | 2.450365091 | 0.001687619 |
| *TGFB2* | Transforming growth factor beta-2 | http://www.genecards.org/cgi-bin/carddisp.pl?gene=TGFB2 | 2.532948253 | 0.009639392 |
| *THBS1* | Thrombospondin-1 | http://www.genecards.org/cgi-bin/carddisp.pl?gene=THBS1 | 1.160824915 | 0.028792649 |
| *THY1* | Thy-1 membrane glycoprotein | http://www.genecards.org/cgi-bin/carddisp.pl?gene=THY1 | 1.646920901 | 0.04292451 |
| *TLN1* | Talin-1 | http://www.genecards.org/cgi-bin/carddisp.pl?gene=TLN1 | 1.544202702 | 0.019187625 |
| *TMEM30A* | Cell cycle control protein 50A | http://www.genecards.org/cgi-bin/carddisp.pl?gene=TMEM30A | 5.940652936 | 0.011501732 |
| *TMEM33* | Transmembrane protein 33 | http://www.genecards.org/cgi-bin/carddisp.pl?gene=TMEM33 | 1.700093037 | 0.009185112 |
| *TMOD3* | Tropomodulin-3 | http://www.genecards.org/cgi-bin/carddisp.pl?gene=TMOD3 | 2.453209565 | 0.001046581 |
| *TMPRSS4* | Transmembrane protease serine 4 | http://www.genecards.org/cgi-bin/carddisp.pl?gene=TMPRSS4 | 4.77357803 | 0.03334266 |
| *TNC* | Tenascin | http://www.genecards.org/cgi-bin/carddisp.pl?gene=TNC | 1.72007073 | 0.003739917 |
| *TNKS1BP1* | 182 kDa tankyrase-1-binding protein | http://www.genecards.org/cgi-bin/carddisp.pl?gene=TNKS1BP1 | 1.791564081 | 0.004817236 |
| *TNS1* | Tensin-1 | http://www.genecards.org/cgi-bin/carddisp.pl?gene=TNS1 | 1.915348221 | 0.004909468 |
| *TNS2* | Tensin-2 | http://www.genecards.org/cgi-bin/carddisp.pl?gene=TNS2 | 1.71727248 | 0.032585019 |
| *TNS3* | Tensin-3 | http://www.genecards.org/cgi-bin/carddisp.pl?gene=TNS3 | 1.627765723 | 0.000180686 |
| *TPBG* | Trophoblast glycoprotein | http://www.genecards.org/cgi-bin/carddisp.pl?gene=TPBG | 4.387902127 | 0.000153578 |
| *TPM1* | Tropomyosin alpha-1 chain | http://www.genecards.org/cgi-bin/carddisp.pl?gene=TPM1 | 6.077240469 | 1.91306E-05 |
| *TPM2* | Tropomyosin beta chain | http://www.genecards.org/cgi-bin/carddisp.pl?gene=TPM2 | 2.484152966 | 0.039739385 |
| *TPM3* | Tropomyosin alpha-3 chain | http://www.genecards.org/cgi-bin/carddisp.pl?gene=TPM3 | 2.488606122 | 0.000278415 |
| *TPM4* | Tropomyosin alpha-4 chain | http://www.genecards.org/cgi-bin/carddisp.pl?gene=TPM4 | 1.443794833 | 0.00015599 |
| *TRIM72* | Tripartite motif-containing protein 72 | http://www.genecards.org/cgi-bin/carddisp.pl?gene=TRIM72 | 2.940622167 | 0.003239106 |
| *TRIO* | Triple functional domain protein | http://www.genecards.org/cgi-bin/carddisp.pl?gene=TRIO | 1.39533572 | 0.042530249 |
| *TSPAN14* | Tetraspanin-14 | http://www.genecards.org/cgi-bin/carddisp.pl?gene=TSPAN14 | 2.68570828 | 0.000168136 |
| *TUBAL3* | Tubulin alpha chain-like 3 | http://www.genecards.org/cgi-bin/carddisp.pl?gene=TUBAL3 | 1.396217598 | 0.020073292 |
| *TUBB1* | Tubulin beta-1 chain | http://www.genecards.org/cgi-bin/carddisp.pl?gene=TUBB1 | 2.45331882 | 0.024299431 |
| *TXNRD1* | Thioredoxin reductase 1, cytoplasmic | http://www.genecards.org/cgi-bin/carddisp.pl?gene=TXNRD1 | 6.890288104 | 0.042599842 |
| *TYK2* | Non-receptor tyrosine-protein kinase TYK2 | http://www.genecards.org/cgi-bin/carddisp.pl?gene=TYK2 | 1.703786097 | 0.009621928 |
| *UBAP2L* | Ubiquitin-associated protein 2-like | http://www.genecards.org/cgi-bin/carddisp.pl?gene=UBAP2L | 2.019632205 | 0.001704992 |
| *UBTD2* | Ubiquitin domain-containing protein 2 | http://www.genecards.org/cgi-bin/carddisp.pl?gene=UBTD2 | 3.285150613 | 0.004464412 |
| *UCHL1* | Ubiquitin carboxyl-terminal hydrolase isozyme L1 | http://www.genecards.org/cgi-bin/carddisp.pl?gene=UCHL1 | 2.053877081 | 0.018846574 |
| *USH2A* | Usherin | http://www.genecards.org/cgi-bin/carddisp.pl?gene=USH2A | 3.119245172 | 0.006358837 |
| *VAMP3* | Vesicle-associated membrane protein 3 | http://www.genecards.org/cgi-bin/carddisp.pl?gene=VAMP3 | 1.880660541 | 0.043726437 |
| *VASN* | Vasorin | http://www.genecards.org/cgi-bin/carddisp.pl?gene=VASN | 2.869969302 | 0.001638119 |
| *VASP* | Vasodilator-stimulated phosphoprotein | http://www.genecards.org/cgi-bin/carddisp.pl?gene=VASP | 1.535225108 | 0.027230624 |
| *VCL* | Vinculin | http://www.genecards.org/cgi-bin/carddisp.pl?gene=VCL | 1.68973645 | 0.000433981 |
| *VCP* | Transitional endoplasmic reticulum ATPase | http://www.genecards.org/cgi-bin/carddisp.pl?gene=VCP | 1.520366888 | 0.004467385 |
| *VDAC2* | Voltage-dependent anion-selective channel protein 2 | http://www.genecards.org/cgi-bin/carddisp.pl?gene=VDAC2 | 3.150545032 | 0.015700078 |
| *VIM* | Vimentin | http://www.genecards.org/cgi-bin/carddisp.pl?gene=VIM | 1.677995469 | 0.010939054 |
| *VIPR1* | Vasoactive intestinal polypeptide receptor 1 | http://www.genecards.org/cgi-bin/carddisp.pl?gene=VIPR1 | 2.993848037 | 0.004046131 |
| *WNT7A* | Protein Wnt-7a | http://www.genecards.org/cgi-bin/carddisp.pl?gene=WNT7A | 7.490447444 | 0.005406256 |
| *XAF1* | XIAP-associated factor 1 | http://www.genecards.org/cgi-bin/carddisp.pl?gene=XAF1 | 22.1913163 | 0.004596264 |
| *YBX1* | Nuclease-sensitive element-binding protein 1 | http://www.genecards.org/cgi-bin/carddisp.pl?gene=YBX1 | 2.409599503 | 0.00076126 |
| *YWHAB* | 14-3-3 protein beta/alpha | http://www.genecards.org/cgi-bin/carddisp.pl?gene=YWHAB | 1.920312011 | 0.000412692 |
| *YWHAE* | 14-3-3 protein epsilon | http://www.genecards.org/cgi-bin/carddisp.pl?gene=YWHAE | 3.858299879 | 0.011201227 |
| *YWHAH* | 14-3-3 protein eta | http://www.genecards.org/cgi-bin/carddisp.pl?gene=YWHAH | 2.088788046 | 0.000182302 |
| *YWHAQ* | 14-3-3 protein theta | http://www.genecards.org/cgi-bin/carddisp.pl?gene=YWHAQ | 2.035740142 | 0.024820023 |
| *YWHAZ* | 14-3-3 protein zeta/delta | http://www.genecards.org/cgi-bin/carddisp.pl?gene=YWHAZ | 1.520324626 | 0.00612295 |
| *ZC2HC1B* | Zinc finger C2HC domain-containing protein 1B | http://www.genecards.org/cgi-bin/carddisp.pl?gene=ZC2HC1B | 2.195201896 | 0.042503274 |
| *ZNF248* | Zinc finger protein 248 | http://www.genecards.org/cgi-bin/carddisp.pl?gene=ZNF248 | 13.18346844 | 0.029521368 |
| *ZYX* | Zyxin | http://www.genecards.org/cgi-bin/carddisp.pl?gene=ZYX | 1.806909807 | 0.014679594 |

**Increased abundance in FA fraction isolated from RPEp53^-/-^SAS6^-/-^ cells**

| **Gene name** | **Description** | [**Gene information**](https://l.facebook.com/l.php?u=http%3A%2F%2Fwww.genecards.org%2Fcgi-bin%2Fcarddisp.pl%3Fgene%3D%2522%252CA1&h=ATP6yswR14bvN0ocja1Ey1G_azEX6hPvaoaG6wnlhO80hCNibRxOBC7mpa7DI2EWkCMhK9LIGIVnWVtcmb2ldic7GGL5x8e6YusaY-ljmcwOhdMPSq3PhaYfIKBlTYELWIWn) | **Centrosome Dependence Ratio** | **p-value**  **(Student's t-test)** |
| --- | --- | --- | --- | --- |
| *ABCA13* | ATP-binding cassette sub-family A member 13 | http://www.genecards.org/cgi-bin/carddisp.pl?gene=ABCA13 | 0.691671741 | 0.006763255 |
| *ABCA6* | ATP-binding cassette sub-family A member 6 | http://www.genecards.org/cgi-bin/carddisp.pl?gene=ABCA6 | 0.5911659 | 0.007096932 |
| *ACO1* | Cytoplasmic aconitate hydratase | http://www.genecards.org/cgi-bin/carddisp.pl?gene=ACO1 | 0.535280521 | 0.020784061 |
| *ADAMTS1* | A disintegrin and metalloproteinase with thrombospondin motifs 1 | http://www.genecards.org/cgi-bin/carddisp.pl?gene=ADAMTS1 | 0.392893844 | 0.000375544 |
| *ADAMTSL1* | ADAMTS-like protein 1 | http://www.genecards.org/cgi-bin/carddisp.pl?gene=ADAMTSL1 | 0.722013995 | 0.000284506 |
| *ADGRL1* | Adhesion G protein-coupled receptor L1 | http://www.genecards.org/cgi-bin/carddisp.pl?gene=ADGRL1 | 0.265787975 | 0.043621178 |
| *AGRN* | Agrin | http://www.genecards.org/cgi-bin/carddisp.pl?gene=AGRN | 0.63541558 | 0.022774452 |
| *AHSG* | Alpha-2-HS-glycoprotein | http://www.genecards.org/cgi-bin/carddisp.pl?gene=AHSG | 0.636803328 | 0.000246997 |
| *ANGPT1* | Angiopoietin-1 | http://www.genecards.org/cgi-bin/carddisp.pl?gene=ANGPT1 | 0.762893808 | 0.042560137 |
| *ANK3* | Ankyrin-3 | http://www.genecards.org/cgi-bin/carddisp.pl?gene=ANK3 | 0.563839467 | 0.006502703 |
| *ANKRD18A* | Ankyrin repeat domain-containing protein 18A | http://www.genecards.org/cgi-bin/carddisp.pl?gene=ANKRD18A | 0.119089354 | 0.034714408 |
| *ANOS1* | Anosmin-1 | http://www.genecards.org/cgi-bin/carddisp.pl?gene=ANOS1 | 0.112740501 | 0.000891018 |
| *ANXA1* | Annexin A1 | http://www.genecards.org/cgi-bin/carddisp.pl?gene=ANXA1 | 0.639186667 | 0.000830813 |
| *ANXA4* | Annexin A4 | http://www.genecards.org/cgi-bin/carddisp.pl?gene=ANXA4 | 0.664578657 | 0.044560818 |
| *AP2A2* | AP-2 complex subunit alpha-2 | http://www.genecards.org/cgi-bin/carddisp.pl?gene=AP2A2 | 0.729800634 | 0.028093661 |
| *APOC3* | Apolipoprotein C-III | http://www.genecards.org/cgi-bin/carddisp.pl?gene=APOC3 | 0.700648203 | 0.023073216 |
| *APOE* | Apolipoprotein E | http://www.genecards.org/cgi-bin/carddisp.pl?gene=APOE | 0.753780286 | 0.045123528 |
| *APOH* | Beta-2-glycoprotein 1 | http://www.genecards.org/cgi-bin/carddisp.pl?gene=APOH | 0.529754095 | 0.010572995 |
| *APOM* | Apolipoprotein M | http://www.genecards.org/cgi-bin/carddisp.pl?gene=APOM | 0.428822671 | 0.001402808 |
| *ASAP2* | Arf-GAP with SH3 domain, ANK repeat and PH domain-containing protein 2 | http://www.genecards.org/cgi-bin/carddisp.pl?gene=ASAP2 | 0.477126885 | 0.033671832 |
| *ATIC* | Bifunctional purine biosynthesis protein PURH | http://www.genecards.org/cgi-bin/carddisp.pl?gene=ATIC | 0.237695566 | 0.008869771 |
| *ATP2B2* | Plasma membrane calcium-transporting ATPase 2 | http://www.genecards.org/cgi-bin/carddisp.pl?gene=ATP2B2 | 0.614334105 | 0.006131196 |
| *ATP5H* | ATP synthase subunit d, mitochondrial | http://www.genecards.org/cgi-bin/carddisp.pl?gene=ATP5H | 0.841811765 | 0.029291288 |
| *ATP6V1D* | V-type proton ATPase subunit D | http://www.genecards.org/cgi-bin/carddisp.pl?gene=ATP6V1D | 0.490426573 | 0.040099927 |
| *BAIAP2* | Brain-specific angiogenesis inhibitor 1-associated protein 2 | http://www.genecards.org/cgi-bin/carddisp.pl?gene=BAIAP2 | 0.210244796 | 0.023009684 |
| *BHMG1* | Basic helix-loop-helix and HMG box domain-containing protein 1 | http://www.genecards.org/cgi-bin/carddisp.pl?gene=BHMG1 | 0.530486247 | 0.00105466 |
| *BMP1* | Bone morphogenetic protein 1 | http://www.genecards.org/cgi-bin/carddisp.pl?gene=BMP1 | 0.586803708 | 0.010931045 |
| *BRD9* | Bromodomain-containing protein 9 | http://www.genecards.org/cgi-bin/carddisp.pl?gene=BRD9 | 0.520254366 | 0.002402463 |
| *C6orf10* | Uncharacterized protein C6orf10 | http://www.genecards.org/cgi-bin/carddisp.pl?gene=C6orf10 | 0.349566283 | 0.032298119 |
| *CACTIN-AS1* | Putative uncharacterized protein encoded by CACTIN-AS1 | http://www.genecards.org/cgi-bin/carddisp.pl?gene=CACTIN-AS1 | 0.240447377 | 0.002708044 |
| *CAMK1D* | Calcium/calmodulin-dependent protein kinase type 1D | http://www.genecards.org/cgi-bin/carddisp.pl?gene=CAMK1D | 0.633092908 | 0.024998631 |
| *CAPN1* | Calpain-1 catalytic subunit | http://www.genecards.org/cgi-bin/carddisp.pl?gene=CAPN1 | 0.802309922 | 0.031449864 |
| *CAPNS1* | Calpain small subunit 1 | http://www.genecards.org/cgi-bin/carddisp.pl?gene=CAPNS1 | 0.739444841 | 0.001259998 |
| *CASP12* | Inactive caspase-12 | http://www.genecards.org/cgi-bin/carddisp.pl?gene=CASP12 | 0.491519839 | 0.001939951 |
| *CAT* | Catalase | http://www.genecards.org/cgi-bin/carddisp.pl?gene=CAT | 0.340513445 | 0.015583977 |
| *CCDC124* | Coiled-coil domain-containing protein 124 | http://www.genecards.org/cgi-bin/carddisp.pl?gene=CCDC124 | 0.688392202 | 0.015523407 |
| *CCDC158* | Coiled-coil domain-containing protein 158 | http://www.genecards.org/cgi-bin/carddisp.pl?gene=CCDC158 | 0.53834432 | 0.008664129 |
| *CCDC47* | Coiled-coil domain-containing protein 47 | http://www.genecards.org/cgi-bin/carddisp.pl?gene=CCDC47 | 0.548615557 | 0.001276095 |
| *CCL18* | C-C motif chemokine 18 | http://www.genecards.org/cgi-bin/carddisp.pl?gene=CCL18 | 0.235630049 | 0.043976183 |
| *CCL26* | C-C motif chemokine 26 | http://www.genecards.org/cgi-bin/carddisp.pl?gene=CCL26 | 0.439606925 | 0.002426542 |
| *CD55* | Complement decay-accelerating factor | http://www.genecards.org/cgi-bin/carddisp.pl?gene=CD55 | 0.547903369 | 8.5027E-05 |
| *CD59* | CD59 glycoprotein | http://www.genecards.org/cgi-bin/carddisp.pl?gene=CD59 | 0.577093057 | 2.82698E-05 |
| *CEP131* | Centrosomal protein of 131 kDa | http://www.genecards.org/cgi-bin/carddisp.pl?gene=CEP131 | 0.556190237 | 0.000537556 |
| *CEP295NL* | CEP295 N-terminal-like protein | http://www.genecards.org/cgi-bin/carddisp.pl?gene=CEP295NL | 0.442310499 | 0.000541358 |
| *CETN3* | Centrin-3 | http://www.genecards.org/cgi-bin/carddisp.pl?gene=CETN3 | 0.544578826 | 0.019924252 |
| *CFAP43* | Cilia- and flagella-associated protein 43 | http://www.genecards.org/cgi-bin/carddisp.pl?gene=CFAP43 | 0.261983677 | 0.001021217 |
| *CFH* | Complement factor H | http://www.genecards.org/cgi-bin/carddisp.pl?gene=CFH | 0.443039563 | 0.000101701 |
| *CHRDL1* | Chordin-like protein 1 | http://www.genecards.org/cgi-bin/carddisp.pl?gene=CHRDL1 | 0.71767731 | 0.00403781 |
| *CHST2* | Carbohydrate sulfotransferase 2 | http://www.genecards.org/cgi-bin/carddisp.pl?gene=CHST2 | 0.341325111 | 7.60775E-05 |
| *CIRBP* | Cold-inducible RNA-binding protein | http://www.genecards.org/cgi-bin/carddisp.pl?gene=CIRBP | 0.476815987 | 0.00139897 |
| *CLEC3B* | Tetranectin | http://www.genecards.org/cgi-bin/carddisp.pl?gene=CLEC3B | 0.446309957 | 0.003480558 |
| *CLTB* | Clathrin light chain B | http://www.genecards.org/cgi-bin/carddisp.pl?gene=CLTB | 0.595371541 | 0.000540254 |
| *COL1A1* | Collagen alpha-1(I) chain | http://www.genecards.org/cgi-bin/carddisp.pl?gene=COL1A1 | 0.547339359 | 0.016110694 |
| *COL7A1* | Collagen alpha-1(VII) chain | http://www.genecards.org/cgi-bin/carddisp.pl?gene=COL7A1 | 0.715113471 | 0.007439913 |
| *CPZ* | Carboxypeptidase Z | http://www.genecards.org/cgi-bin/carddisp.pl?gene=CPZ | 0.565612445 | 0.003134383 |
| *CRYBG3* | Very large A-kinase anchor protein | http://www.genecards.org/cgi-bin/carddisp.pl?gene=CRYBG3 | 0.627234903 | 0.015644644 |
| *CTAGE1* | cTAGE family member 2 | http://www.genecards.org/cgi-bin/carddisp.pl?gene=CTAGE1 | 0.27060581 | 0.000486386 |
| *CTGF* | Connective tissue growth factor | http://www.genecards.org/cgi-bin/carddisp.pl?gene=CTGF | 0.165138118 | 0.000868018 |
| *DCD* | Dermcidin | http://www.genecards.org/cgi-bin/carddisp.pl?gene=DCD | 0.473847198 | 0.029227642 |
| *DHX33* | Putative ATP-dependent RNA helicase DHX33 | http://www.genecards.org/cgi-bin/carddisp.pl?gene=DHX33 | 0.435111782 | 0.032642799 |
| *DLC1* | Rho GTPase-activating protein 7 | http://www.genecards.org/cgi-bin/carddisp.pl?gene=DLC1 | 0.559979262 | 0.02126435 |
| *DNAI1* | Dynein intermediate chain 1, axonemal | http://www.genecards.org/cgi-bin/carddisp.pl?gene=DNAI1 | 0.513345575 | 0.000552273 |
| *DOCK7* | Dedicator of cytokinesis protein 7 | http://www.genecards.org/cgi-bin/carddisp.pl?gene=DOCK7 | 0.19942179 | 0.005629154 |
| *DSC3* | Desmocollin-3 | http://www.genecards.org/cgi-bin/carddisp.pl?gene=DSC3 | 0.713837484 | 0.024413847 |
| *DSP* | Desmoplakin | http://www.genecards.org/cgi-bin/carddisp.pl?gene=DSP | 0.637212347 | 0.033355104 |
| *EIF3CL* | Eukaryotic translation initiation factor 3 subunit C-like protein | http://www.genecards.org/cgi-bin/carddisp.pl?gene=EIF3CL | 0.58554187 | 0.005349174 |
| *EIF4A3* | Eukaryotic initiation factor 4A-III | http://www.genecards.org/cgi-bin/carddisp.pl?gene=EIF4A3 | 0.393965843 | 0.00770883 |
| *EIF4H* | Eukaryotic translation initiation factor 4H | http://www.genecards.org/cgi-bin/carddisp.pl?gene=EIF4H | 0.535009745 | 0.010635296 |
| *ELL2* | RNA polymerase II elongation factor ELL2 | http://www.genecards.org/cgi-bin/carddisp.pl?gene=ELL2 | 0.510337541 | 0.00033453 |
| *EPB41* | Protein 4.1 | http://www.genecards.org/cgi-bin/carddisp.pl?gene=EPB41 | 0.5869099 | 0.049264303 |
| *EPHA4* | Ephrin type-A receptor 4 | http://www.genecards.org/cgi-bin/carddisp.pl?gene=EPHA4 | 0.449142582 | 0.005131541 |
| *EPS15L1* | Epidermal growth factor receptor substrate 15-like 1 | http://www.genecards.org/cgi-bin/carddisp.pl?gene=EPS15L1 | 0.542974445 | 0.003369073 |
| *ESCO1* | N-acetyltransferase ESCO1 | http://www.genecards.org/cgi-bin/carddisp.pl?gene=ESCO1 | 0.477333684 | 0.002944598 |
| *ESM1* | Endothelial cell-specific molecule 1 | http://www.genecards.org/cgi-bin/carddisp.pl?gene=ESM1 | 0.338591421 | 5.223E-05 |
| *ETF1* | Eukaryotic peptide chain release factor subunit 1 | http://www.genecards.org/cgi-bin/carddisp.pl?gene=ETF1 | 0.652747537 | 0.047076715 |
| *F13B* | Coagulation factor XIII B chain | http://www.genecards.org/cgi-bin/carddisp.pl?gene=F13B | 0.562166555 | 0.019381565 |
| *F2* | Prothrombin | http://www.genecards.org/cgi-bin/carddisp.pl?gene=F2 | 0.498350085 | 7.81812E-05 |
| *F5* | Coagulation factor V | http://www.genecards.org/cgi-bin/carddisp.pl?gene=F5 | 0.652493702 | 0.002623083 |
| *FAT2* | Protocadherin Fat 2 | http://www.genecards.org/cgi-bin/carddisp.pl?gene=FAT2 | 0.733677872 | 0.003620985 |
| *FBLN1* | Fibulin-1 | http://www.genecards.org/cgi-bin/carddisp.pl?gene=FBLN1 | 0.681014828 | 0.001982927 |
| *FERMT3* | Fermitin family homolog 3 | http://www.genecards.org/cgi-bin/carddisp.pl?gene=FERMT3 | 0.640644526 | 0.001395361 |
| *FGG* | Fibrinogen gamma chain | http://www.genecards.org/cgi-bin/carddisp.pl?gene=FGG | 0.467306104 | 0.000927655 |
| *FLII* | Protein flightless-1 homolog | http://www.genecards.org/cgi-bin/carddisp.pl?gene=FLII | 0.560661463 | 0.015974586 |
| *GC* | Vitamin D-binding protein | http://www.genecards.org/cgi-bin/carddisp.pl?gene=GC | 0.785938935 | 0.020388623 |
| *GDF6* | Growth/differentiation factor 6 | http://www.genecards.org/cgi-bin/carddisp.pl?gene=GDF6 | 0.325613864 | 0.000323013 |
| *GDI2* | Rab GDP dissociation inhibitor beta | http://www.genecards.org/cgi-bin/carddisp.pl?gene=GDI2 | 0.524245997 | 0.006804809 |
| *GLIPR2* | Golgi-associated plant pathogenesis-related protein 1 | http://www.genecards.org/cgi-bin/carddisp.pl?gene=GLIPR2 | 0.784508234 | 0.027231848 |
| *GPRIN1* | G protein-regulated inducer of neurite outgrowth 1 | http://www.genecards.org/cgi-bin/carddisp.pl?gene=GPRIN1 | 0.433745655 | 0.043482708 |
| *GREM1* | Gremlin-1 | http://www.genecards.org/cgi-bin/carddisp.pl?gene=GREM1 | 0.642347404 | 0.017054325 |
| *GSN* | Gelsolin | http://www.genecards.org/cgi-bin/carddisp.pl?gene=GSN | 0.631182639 | 0.006520176 |
| *GTF2E1* | General transcription factor IIE subunit 1 | http://www.genecards.org/cgi-bin/carddisp.pl?gene=GTF2E1 | 0.415091329 | 0.006414757 |
| *HABP2* | Hyaluronan-binding protein 2 | http://www.genecards.org/cgi-bin/carddisp.pl?gene=HABP2 | 0.457455555 | 0.001038 |
| *HDAC7* | Histone deacetylase 7 | http://www.genecards.org/cgi-bin/carddisp.pl?gene=HDAC7 | 0.392425913 | 0.029129807 |
| *HERC3* | Probable E3 ubiquitin-protein ligase HERC3 | http://www.genecards.org/cgi-bin/carddisp.pl?gene=HERC3 | 0.474902816 | 0.017158955 |
| *HHIP* | Hedgehog-interacting protein | http://www.genecards.org/cgi-bin/carddisp.pl?gene=HHIP | 0.334187213 | 6.10304E-05 |
| *HIST1H1C* | Histone H1.2 | http://www.genecards.org/cgi-bin/carddisp.pl?gene=HIST1H1C | 0.716887384 | 0.025066663 |
| *HIST1H4A* | Histone H4 | http://www.genecards.org/cgi-bin/carddisp.pl?gene=HIST1H4A | 0.518554014 | 0.006787288 |
| *HLA-DRB1* | HLA class II histocompatibility antigen, DRB1-12 beta chain | http://www.genecards.org/cgi-bin/carddisp.pl?gene=HLA-DRB1 | 0.442629317 | 0.001009751 |
| *HMGA1* | High mobility group protein HMG-I/HMG-Y | http://www.genecards.org/cgi-bin/carddisp.pl?gene=HMGA1 | 0.519908997 | 2.23383E-05 |
| *HMGB1* | High mobility group protein B1 | http://www.genecards.org/cgi-bin/carddisp.pl?gene=HMGB1 | 0.323425558 | 0.007111642 |
| *HPCAL1* | Hippocalcin-like protein 1 | http://www.genecards.org/cgi-bin/carddisp.pl?gene=HPCAL1 | 0.434708968 | 0.001121947 |
| *HSP90AA1* | Heat shock protein HSP 90-alpha | http://www.genecards.org/cgi-bin/carddisp.pl?gene=HSP90AA1 | 0.813480611 | 0.026190756 |
| *HSP90AB2P* | Putative heat shock protein HSP 90-beta 2 | http://www.genecards.org/cgi-bin/carddisp.pl?gene=HSP90AB2P | 0.481371917 | 0.000622958 |
| *HSP90AB4P* | Putative heat shock protein HSP 90-beta 4 | http://www.genecards.org/cgi-bin/carddisp.pl?gene=HSP90AB4P | 0.451799528 | 0.012459804 |
| *HSP90B2P* | Putative endoplasmin-like protein | http://www.genecards.org/cgi-bin/carddisp.pl?gene=HSP90B2P | 0.103735049 | 0.045485383 |
| *HSPA1L* | Heat shock 70 kDa protein 1-like | http://www.genecards.org/cgi-bin/carddisp.pl?gene=HSPA1L | 0.511267108 | 0.004742976 |
| *HSPA2* | Heat shock-related 70 kDa protein 2 | http://www.genecards.org/cgi-bin/carddisp.pl?gene=HSPA2 | 0.557306286 | 0.002626396 |
| *IGF2* | Insulin-like growth factor II | http://www.genecards.org/cgi-bin/carddisp.pl?gene=IGF2 | 0.444139592 | 0.002186377 |
| *IGFBP2* | Insulin-like growth factor-binding protein 2 | http://www.genecards.org/cgi-bin/carddisp.pl?gene=IGFBP2 | 0.369047024 | 0.000865976 |
| *IGFBP4* | Insulin-like growth factor-binding protein 4 | http://www.genecards.org/cgi-bin/carddisp.pl?gene=IGFBP4 | 0.501482673 | 0.006122798 |
| *IGFBP7* | Insulin-like growth factor-binding protein 7 | http://www.genecards.org/cgi-bin/carddisp.pl?gene=IGFBP7 | 0.499170061 | 0.000677706 |
| *ING2* | Inhibitor of growth protein 2 | http://www.genecards.org/cgi-bin/carddisp.pl?gene=ING2 | 0.466769737 | 0.016425863 |
| *ISOC1* | Isochorismatase domain-containing protein 1 | http://www.genecards.org/cgi-bin/carddisp.pl?gene=ISOC1 | 0.737197724 | 0.004408465 |
| *ITGA2* | Integrin alpha-2 | http://www.genecards.org/cgi-bin/carddisp.pl?gene=ITGA2 | 0.425820186 | 0.000177382 |
| *ITIH2* | Inter-alpha-trypsin inhibitor heavy chain H2 | http://www.genecards.org/cgi-bin/carddisp.pl?gene=ITIH2 | 0.734713348 | 0.00431963 |
| *ITIH3* | Inter-alpha-trypsin inhibitor heavy chain H3 | http://www.genecards.org/cgi-bin/carddisp.pl?gene=ITIH3 | 0.692882737 | 0.039793533 |
| *ITIH4* | Inter-alpha-trypsin inhibitor heavy chain H4 | http://www.genecards.org/cgi-bin/carddisp.pl?gene=ITIH4 | 0.623743127 | 0.000170818 |
| *KAT6B* | Histone acetyltransferase KAT6B | http://www.genecards.org/cgi-bin/carddisp.pl?gene=KAT6B | 0.59481592 | 0.04420134 |
| *KATNAL2* | Katanin p60 ATPase-containing subunit A-like 2 | http://www.genecards.org/cgi-bin/carddisp.pl?gene=KATNAL2 | 0.073946976 | 0.006075842 |
| *KIAA1462* | Junctional protein associated with coronary artery disease | http://www.genecards.org/cgi-bin/carddisp.pl?gene=KIAA1462 | 0.455214091 | 0.008268709 |
| *KIF5B* | Kinesin-1 heavy chain | http://www.genecards.org/cgi-bin/carddisp.pl?gene=KIF5B | 0.563916738 | 0.017804212 |
| *KRT15* | Keratin, type I cytoskeletal 15 | http://www.genecards.org/cgi-bin/carddisp.pl?gene=KRT15 | 0.352106698 | 0.005866543 |
| *KRT25* | Keratin, type I cytoskeletal 25 | http://www.genecards.org/cgi-bin/carddisp.pl?gene=KRT25 | 0.588310507 | 0.009782341 |
| *KRT31* | Keratin, type I cuticular Ha1 | http://www.genecards.org/cgi-bin/carddisp.pl?gene=KRT31 | 0.561846378 | 0.011445449 |
| *KRT4* | Keratin, type II cytoskeletal 4 | http://www.genecards.org/cgi-bin/carddisp.pl?gene=KRT4 | 0.690274488 | 0.009455494 |
| *KSR2* | Kinase suppressor of Ras 2 | http://www.genecards.org/cgi-bin/carddisp.pl?gene=KSR2 | 0.577870479 | 0.000317871 |
| *LAMA1* | Laminin subunit alpha-1 | http://www.genecards.org/cgi-bin/carddisp.pl?gene=LAMA1 | 0.503268821 | 0.01545395 |
| *LARP7* | La-related protein 7 | http://www.genecards.org/cgi-bin/carddisp.pl?gene=LARP7 | 0.32280077 | 0.0028252 |
| *LBP* | Lipopolysaccharide-binding protein | http://www.genecards.org/cgi-bin/carddisp.pl?gene=LBP | 0.659778682 | 0.001785731 |
| *LIMD1* | LIM domain-containing protein 1 | http://www.genecards.org/cgi-bin/carddisp.pl?gene=LIMD1 | 0.452069353 | 9.47302E-05 |
| *LOXL2* | Lysyl oxidase homolog 2 | http://www.genecards.org/cgi-bin/carddisp.pl?gene=LOXL2 | 0.360078786 | 0.000172348 |
| *LRMP* | Lymphoid-restricted membrane protein | http://www.genecards.org/cgi-bin/carddisp.pl?gene=LRMP | 0.35086872 | 0.004110714 |
| *LSM14A* | Protein LSM14 homolog A | http://www.genecards.org/cgi-bin/carddisp.pl?gene=LSM14A | 0.684211492 | 0.034398145 |
| *LTBP1* | Latent-transforming growth factor beta-binding protein 1 | http://www.genecards.org/cgi-bin/carddisp.pl?gene=LTBP1 | 0.168517335 | 0.000620849 |
| *MANF* | Mesencephalic astrocyte-derived neurotrophic factor | http://www.genecards.org/cgi-bin/carddisp.pl?gene=MANF | 0.434662783 | 0.003827133 |
| *MAP4* | Microtubule-associated protein 4 | http://www.genecards.org/cgi-bin/carddisp.pl?gene=MAP4 | 0.846382424 | 0.033227311 |
| *MAP7D2* | MAP7 domain-containing protein 2 | http://www.genecards.org/cgi-bin/carddisp.pl?gene=MAP7D2 | 0.469619584 | 0.000276058 |
| *MASP1* | Mannan-binding lectin serine protease 1 | http://www.genecards.org/cgi-bin/carddisp.pl?gene=MASP1 | 0.533103551 | 0.01653574 |
| *MDK* | Midkine | http://www.genecards.org/cgi-bin/carddisp.pl?gene=MDK | 0.14727367 | 0.000890521 |
| *MIF* | Macrophage migration inhibitory factor | http://www.genecards.org/cgi-bin/carddisp.pl?gene=MIF | 0.491481063 | 0.019666057 |
| *MTAP* | S-methyl-5'-thioadenosine phosphorylase | http://www.genecards.org/cgi-bin/carddisp.pl?gene=MTAP | 0.448249923 | 0.003128542 |
| *MYCBP* | C-Myc-binding protein | http://www.genecards.org/cgi-bin/carddisp.pl?gene=MYCBP | 0.834526657 | 0.045252956 |
| *NACA* | Nascent polypeptide-associated complex subunit alpha, muscle-specific form | http://www.genecards.org/cgi-bin/carddisp.pl?gene=NACA | 0.757050514 | 0.001247506 |
| *NCOR1* | Nuclear receptor corepressor 1 | http://www.genecards.org/cgi-bin/carddisp.pl?gene=NCOR1 | 0.688587129 | 0.000455522 |
| *NTN4* | Netrin-4 | http://www.genecards.org/cgi-bin/carddisp.pl?gene=NTN4 | 0.111428768 | 7.00524E-05 |
| *NUMB* | Protein numb homolog | http://www.genecards.org/cgi-bin/carddisp.pl?gene=NUMB | 0.574970424 | 0.001513538 |
| *OR6Y1* | Olfactory receptor 6Y1 | http://www.genecards.org/cgi-bin/carddisp.pl?gene=OR6Y1 | 0.204090354 | 0.004702702 |
| *PGK1* | Phosphoglycerate kinase 1 | http://www.genecards.org/cgi-bin/carddisp.pl?gene=PGK1 | 0.596002333 | 0.007487517 |
| *PHF10* | PHD finger protein 10 | http://www.genecards.org/cgi-bin/carddisp.pl?gene=PHF10 | 0.55685255 | 0.018978346 |
| *PIP4K2A* | Phosphatidylinositol 5-phosphate 4-kinase type-2 alpha | http://www.genecards.org/cgi-bin/carddisp.pl?gene=PIP4K2A | 0.639862059 | 0.026679672 |
| *PLA1A* | Phospholipase A1 member A | http://www.genecards.org/cgi-bin/carddisp.pl?gene=PLA1A | 0.484375721 | 3.91272E-05 |
| *PLAT* | Tissue-type plasminogen activator | http://www.genecards.org/cgi-bin/carddisp.pl?gene=PLAT | 0.403747698 | 7.69345E-06 |
| *PLEK* | Pleckstrin | http://www.genecards.org/cgi-bin/carddisp.pl?gene=PLEK | 0.46079465 | 0.003721022 |
| *POLK* | DNA polymerase kappa | http://www.genecards.org/cgi-bin/carddisp.pl?gene=POLK | 0.692569396 | 0.022617936 |
| *POSTN* | Periostin | http://www.genecards.org/cgi-bin/carddisp.pl?gene=POSTN | 0.708407785 | 0.004695083 |
| *PPIE* | Peptidyl-prolyl cis-trans isomerase E | http://www.genecards.org/cgi-bin/carddisp.pl?gene=PPIE | 0.548602663 | 0.000195508 |
| *PRDX2* | Peroxiredoxin-2 | http://www.genecards.org/cgi-bin/carddisp.pl?gene=PRDX2 | 0.829753679 | 0.029364366 |
| *PRLR* | Prolactin receptor | http://www.genecards.org/cgi-bin/carddisp.pl?gene=PRLR | 0.622151157 | 0.023399486 |
| *PRSS12* | Neurotrypsin | http://www.genecards.org/cgi-bin/carddisp.pl?gene=PRSS12 | 0.304301111 | 0.008220464 |
| *PRSS23* | Serine protease 23 | http://www.genecards.org/cgi-bin/carddisp.pl?gene=PRSS23 | 0.571840242 | 0.008380656 |
| *PTPRK* | Receptor-type tyrosine-protein phosphatase kappa | http://www.genecards.org/cgi-bin/carddisp.pl?gene=PTPRK | 0.658576599 | 0.018797098 |
| *PXDN* | Peroxidasin homolog | http://www.genecards.org/cgi-bin/carddisp.pl?gene=PXDN | 0.515800367 | 0.004584552 |
| *QSOX1* | Sulfhydryl oxidase 1 | http://www.genecards.org/cgi-bin/carddisp.pl?gene=QSOX1 | 0.508335703 | 4.9699E-05 |
| *RAI14* | Ankycorbin | http://www.genecards.org/cgi-bin/carddisp.pl?gene=RAI14 | 0.359504664 | 0.01056379 |
| *RAN* | GTP-binding nuclear protein Ran | http://www.genecards.org/cgi-bin/carddisp.pl?gene=RAN | 0.717735343 | 0.002585482 |
| *RCC1* | Regulator of chromosome condensation | http://www.genecards.org/cgi-bin/carddisp.pl?gene=RCC1 | 0.430950425 | 0.000500494 |
| *RDX* | Radixin | http://www.genecards.org/cgi-bin/carddisp.pl?gene=RDX | 0.507125537 | 0.007234659 |
| *RIMBP3* | RIMS-binding protein 3A | http://www.genecards.org/cgi-bin/carddisp.pl?gene=RIMBP3 | 0.641507764 | 0.02390306 |
| *RNASE4* | Ribonuclease 4 | http://www.genecards.org/cgi-bin/carddisp.pl?gene=RNASE4 | 0.603026007 | 0.012139718 |
| *RNF40* | E3 ubiquitin-protein ligase BRE1B | http://www.genecards.org/cgi-bin/carddisp.pl?gene=RNF40 | 0.263086782 | 0.000128103 |
| *RPS10P5* | Putative 40S ribosomal protein S10-like | http://www.genecards.org/cgi-bin/carddisp.pl?gene=RPS10P5 | 0.436782015 | 0.03779388 |
| *RSF1* | Remodeling and spacing factor 1 | http://www.genecards.org/cgi-bin/carddisp.pl?gene=RSF1 | 0.591417794 | 0.002827626 |
| *S100A6* | Protein S100-A6 | http://www.genecards.org/cgi-bin/carddisp.pl?gene=S100A6 | 0.767872878 | 0.023035415 |
| *S100A7* | Protein S100-A7 | http://www.genecards.org/cgi-bin/carddisp.pl?gene=S100A7 | 0.473957048 | 0.036848715 |
| *SBDS* | Ribosome maturation protein SBDS | http://www.genecards.org/cgi-bin/carddisp.pl?gene=SBDS | 0.391516586 | 0.01495421 |
| *SEMA3A* | Semaphorin-3A | http://www.genecards.org/cgi-bin/carddisp.pl?gene=SEMA3A | 0.285359039 | 0.031312679 |
| *SERPINC1* | Antithrombin-III | http://www.genecards.org/cgi-bin/carddisp.pl?gene=SERPINC1 | 0.598147567 | 0.001750472 |
| *SERPIND1* | Heparin cofactor 2 | http://www.genecards.org/cgi-bin/carddisp.pl?gene=SERPIND1 | 0.628155198 | 0.003872036 |
| *SF3B2* | Splicing factor 3B subunit 2 | http://www.genecards.org/cgi-bin/carddisp.pl?gene=SF3B2 | 0.463593632 | 0.049301132 |
| *SFRP1* | Secreted frizzled-related protein 1 | http://www.genecards.org/cgi-bin/carddisp.pl?gene=SFRP1 | 0.387851699 | 2.8809E-05 |
| *SHROOM3* | Protein Shroom3 | http://www.genecards.org/cgi-bin/carddisp.pl?gene=SHROOM3 | 0.637011898 | 0.034628714 |
| *SIM1* | Single-minded homolog 1 | http://www.genecards.org/cgi-bin/carddisp.pl?gene=SIM1 | 0.526317059 | 0.033926839 |
| *SIX6OS1* | Protein SIX6OS1 | http://www.genecards.org/cgi-bin/carddisp.pl?gene=SIX6OS1 | 0.568730567 | 0.016173912 |
| *SLC13A3* | Solute carrier family 13 member 3 | http://www.genecards.org/cgi-bin/carddisp.pl?gene=SLC13A3 | 0.436508642 | 0.000148537 |
| *SLFN14* | Protein SLFN14 | http://www.genecards.org/cgi-bin/carddisp.pl?gene=SLFN14 | 0.775275822 | 0.046286661 |
| *SMC2* | Structural maintenance of chromosomes protein 2 | http://www.genecards.org/cgi-bin/carddisp.pl?gene=SMC2 | 0.687600715 | 0.004169331 |
| *SNRPD3* | Small nuclear ribonucleoprotein Sm D3 | http://www.genecards.org/cgi-bin/carddisp.pl?gene=SNRPD3 | 0.530976864 | 0.01965663 |
| *SPEF2* | Sperm flagellar protein 2 | http://www.genecards.org/cgi-bin/carddisp.pl?gene=SPEF2 | 0.571861245 | 0.006951713 |
| *SREK1* | Splicing regulatory glutamine/lysine-rich protein 1 | http://www.genecards.org/cgi-bin/carddisp.pl?gene=SREK1 | 0.475918645 | 0.002104204 |
| *SRPX* | Sushi repeat-containing protein SRPX | http://www.genecards.org/cgi-bin/carddisp.pl?gene=SRPX | 0.777374159 | 0.049193456 |
| *SSFA2* | Sperm-specific antigen 2 | http://www.genecards.org/cgi-bin/carddisp.pl?gene=SSFA2 | 0.364958034 | 0.021552078 |
| *STC1* | Stanniocalcin-1 | http://www.genecards.org/cgi-bin/carddisp.pl?gene=STC1 | 0.224088462 | 0.003999103 |
| *STIM2* | Stromal interaction molecule 2 | http://www.genecards.org/cgi-bin/carddisp.pl?gene=STIM2 | 0.373043458 | 0.011165257 |
| *STMN1* | Stathmin | http://www.genecards.org/cgi-bin/carddisp.pl?gene=STMN1 | 0.739765717 | 0.004134771 |
| *STXBP3* | Syntaxin-binding protein 3 | http://www.genecards.org/cgi-bin/carddisp.pl?gene=STXBP3 | 0.502068995 | 0.006925364 |
| *TAGLN* | Transgelin | http://www.genecards.org/cgi-bin/carddisp.pl?gene=TAGLN | 0.916698078 | 0.044575827 |
| *TALDO1* | Transaldolase | http://www.genecards.org/cgi-bin/carddisp.pl?gene=TALDO1 | 0.449757466 | 0.038586538 |
| *TAX1BP1* | Tax1-binding protein 1 | http://www.genecards.org/cgi-bin/carddisp.pl?gene=TAX1BP1 | 0.482965853 | 0.002813831 |
| *TBC1D16* | TBC1 domain family member 16 | http://www.genecards.org/cgi-bin/carddisp.pl?gene=TBC1D16 | 0.535089988 | 0.006793085 |
| *TBC1D8* | TBC1 domain family member 8 | http://www.genecards.org/cgi-bin/carddisp.pl?gene=TBC1D8 | 0.737939519 | 0.009327808 |
| *TDRD15* | Tudor domain-containing protein 15 | http://www.genecards.org/cgi-bin/carddisp.pl?gene=TDRD15 | 0.619958599 | 5.47857E-05 |
| *TERT* | Telomerase reverse transcriptase | http://www.genecards.org/cgi-bin/carddisp.pl?gene=TERT | 0.487979473 | 0.018030414 |
| *TFPI* | Tissue factor pathway inhibitor | http://www.genecards.org/cgi-bin/carddisp.pl?gene=TFPI | 0.557187387 | 0.002035988 |
| *TFPI2* | Tissue factor pathway inhibitor 2 | http://www.genecards.org/cgi-bin/carddisp.pl?gene=TFPI2 | 0.379046739 | 0.001009677 |
| *TGFB1* | Transforming growth factor beta-1 | http://www.genecards.org/cgi-bin/carddisp.pl?gene=TGFB1 | 0.604086591 | 0.015976508 |
| *TIMM8A* | Mitochondrial import inner membrane translocase subunit Tim8 A | http://www.genecards.org/cgi-bin/carddisp.pl?gene=TIMM8A | 0.398384439 | 0.02180464 |
| *TIMP3* | Metalloproteinase inhibitor 3 | http://www.genecards.org/cgi-bin/carddisp.pl?gene=TIMP3 | 0.237859367 | 0.001047056 |
| *TJAP1* | Tight junction-associated protein 1 | http://www.genecards.org/cgi-bin/carddisp.pl?gene=TJAP1 | 0.358150496 | 0.000145314 |
| *TLL1* | Tolloid-like protein 1 | http://www.genecards.org/cgi-bin/carddisp.pl?gene=TLL1 | 0.699027189 | 0.028677681 |
| *TRAP1* | Heat shock protein 75 kDa, mitochondrial | http://www.genecards.org/cgi-bin/carddisp.pl?gene=TRAP1 | 0.713556462 | 0.048210965 |
| *TRHDE* | Thyrotropin-releasing hormone-degrading ectoenzyme | http://www.genecards.org/cgi-bin/carddisp.pl?gene=TRHDE | 0.761395277 | 0.007193485 |
| *UBE2N* | Ubiquitin-conjugating enzyme E2 N | http://www.genecards.org/cgi-bin/carddisp.pl?gene=UBE2N | 0.761616969 | 0.020353478 |
| *UBE2V1* | Ubiquitin-conjugating enzyme E2 variant 1 | http://www.genecards.org/cgi-bin/carddisp.pl?gene=UBE2V1 | 0.689362518 | 0.032028432 |
| *UBR4* | E3 ubiquitin-protein ligase UBR4 | http://www.genecards.org/cgi-bin/carddisp.pl?gene=UBR4 | 0.668616434 | 0.021147183 |
| *UBTFL1* | Upstream-binding factor 1-like protein 1 | http://www.genecards.org/cgi-bin/carddisp.pl?gene=UBTFL1 | 0.419904122 | 0.020177664 |
| *UGDH* | UDP-glucose 6-dehydrogenase | http://www.genecards.org/cgi-bin/carddisp.pl?gene=UGDH | 0.646442664 | 0.01314117 |
| *UTP20* | Small subunit processome component 20 homolog | http://www.genecards.org/cgi-bin/carddisp.pl?gene=UTP20 | 0.395562579 | 0.000591557 |
| *UTRN* | Utrophin | http://www.genecards.org/cgi-bin/carddisp.pl?gene=UTRN | 0.562167447 | 0.008700582 |
| *VCAN* | Versican core protein | http://www.genecards.org/cgi-bin/carddisp.pl?gene=VCAN | 0.628502465 | 0.001737935 |
| *VTN* | Vitronectin | http://www.genecards.org/cgi-bin/carddisp.pl?gene=VTN | 0.646148315 | 0.0330943 |
| *VWA7* | von Willebrand factor A domain-containing protein 7 | http://www.genecards.org/cgi-bin/carddisp.pl?gene=VWA7 | 0.259052002 | 0.017858941 |
| *VWF* | von Willebrand factor | http://www.genecards.org/cgi-bin/carddisp.pl?gene=VWF | 0.355607853 | 0.000197432 |
| *WNT5B* | Protein Wnt-5b | http://www.genecards.org/cgi-bin/carddisp.pl?gene=WNT5B | 0.590891103 | 0.000451024 |
| *ZC3H7A* | Zinc finger CCCH domain-containing protein 7A | http://www.genecards.org/cgi-bin/carddisp.pl?gene=ZC3H7A | 0.127478727 | 5.77929E-05 |
| *ZNF140* | Zinc finger protein 140 | http://www.genecards.org/cgi-bin/carddisp.pl?gene=ZNF140 | 0.387993272 | 9.78625E-05 |
| *ZNF561* | Zinc finger protein 561 | http://www.genecards.org/cgi-bin/carddisp.pl?gene=ZNF561 | 0.690584829 | 0.02818253 |

**The same abundance in RPEp53^-/-^ and RPEp53^-/-^SAS6^-/-^ FAs**

| **Gene name** | **Description** | [**Gene information**](https://l.facebook.com/l.php?u=http%3A%2F%2Fwww.genecards.org%2Fcgi-bin%2Fcarddisp.pl%3Fgene%3D%2522%252CA1&h=ATP6yswR14bvN0ocja1Ey1G_azEX6hPvaoaG6wnlhO80hCNibRxOBC7mpa7DI2EWkCMhK9LIGIVnWVtcmb2ldic7GGL5x8e6YusaY-ljmcwOhdMPSq3PhaYfIKBlTYELWIWn) | **Centrosome Dependence Ratio** | **p-value**  **(Student's t-test)** |
| --- | --- | --- | --- | --- |
| *ABCB4* | Phosphatidylcholine translocator ABCB4 | http://www.genecards.org/cgi-bin/carddisp.pl?gene=ABCB4 | 0.639380758 | 0.117078344 |
| *ABCF1* | ATP-binding cassette sub-family F member 1 | http://www.genecards.org/cgi-bin/carddisp.pl?gene=ABCF1 | 0.516329356 | 0.268022423 |
| *ACOX1* | Peroxisomal acyl-coenzyme A oxidase 1 | http://www.genecards.org/cgi-bin/carddisp.pl?gene=ACOX1 | 7.402839535 | 0.126772806 |
| *ACSL4* | Long-chain-fatty-acid--CoA ligase 4 | http://www.genecards.org/cgi-bin/carddisp.pl?gene=ACSL4 | 1.152483141 | 0.274653124 |
| *ACTBL2* | Beta-actin-like protein 2 | http://www.genecards.org/cgi-bin/carddisp.pl?gene=ACTBL2 | 1.007142559 | 0.49443728 |
| *ACTC1* | Actin, alpha cardiac muscle 1 | http://www.genecards.org/cgi-bin/carddisp.pl?gene=ACTC1 | 2.043878104 | 0.231264674 |
| *ACTR2* | Actin-related protein 2 | http://www.genecards.org/cgi-bin/carddisp.pl?gene=ACTR2 | 1.247065485 | 0.279558724 |
| *ADAM19* | Disintegrin and metalloproteinase domain-containing protein 19 | http://www.genecards.org/cgi-bin/carddisp.pl?gene=ADAM19 | 0.741312629 | 0.243974857 |
| *ADAM8* | Disintegrin and metalloproteinase domain-containing protein 8 | http://www.genecards.org/cgi-bin/carddisp.pl?gene=ADAM8 | 0.930027276 | 0.341609977 |
| *ADAMTS15* | A disintegrin and metalloproteinase with thrombospondin motifs 15 | http://www.genecards.org/cgi-bin/carddisp.pl?gene=ADAMTS15 | 1.419194713 | 0.320199815 |
| *ADAMTS5* | A disintegrin and metalloproteinase with thrombospondin motifs 5 | http://www.genecards.org/cgi-bin/carddisp.pl?gene=ADAMTS5 | 0.918353677 | 0.359682345 |
| *AFMID* | Kynurenine formamidase | http://www.genecards.org/cgi-bin/carddisp.pl?gene=AFMID | 0.595906915 | 0.072362989 |
| *AGBL5* | Cytosolic carboxypeptidase-like protein 5 | http://www.genecards.org/cgi-bin/carddisp.pl?gene=AGBL5 | 0.899235917 | 0.255832072 |
| *AGFG1* | Arf-GAP domain and FG repeat-containing protein 1 | http://www.genecards.org/cgi-bin/carddisp.pl?gene=AGFG1 | 0.845454703 | 0.238579049 |
| *AGPAT1* | 1-acyl-sn-glycerol-3-phosphate acyltransferase alpha | http://www.genecards.org/cgi-bin/carddisp.pl?gene=AGPAT1 | 2.049559586 | 0.254932574 |
| *AKAP13* | A-kinase anchor protein 13 | http://www.genecards.org/cgi-bin/carddisp.pl?gene=AKAP13 | 1.204013291 | 0.262123896 |
| *AKAP17A* | A-kinase anchor protein 17A | http://www.genecards.org/cgi-bin/carddisp.pl?gene=AKAP17A | 1.191522624 | 0.398502437 |
| *AKAP2* | A-kinase anchor protein 2 | http://www.genecards.org/cgi-bin/carddisp.pl?gene=AKAP2 | 1.776977474 | 0.103536475 |
| *ALB* | Serum albumin | http://www.genecards.org/cgi-bin/carddisp.pl?gene=ALB | 1.157819794 | 0.062847265 |
| *ALDH18A1* | Delta-1-pyrroline-5-carboxylate synthase | http://www.genecards.org/cgi-bin/carddisp.pl?gene=ALDH18A1 | 1.637642593 | 0.100697369 |
| *ALDOA* | Fructose-bisphosphate aldolase A | http://www.genecards.org/cgi-bin/carddisp.pl?gene=ALDOA | 1.162854123 | 0.056457975 |
| *AMMECR1L* | AMMECR1-like protein | http://www.genecards.org/cgi-bin/carddisp.pl?gene=AMMECR1L | 0.382826326 | 0.089064364 |
| *ANKRD50* | Ankyrin repeat domain-containing protein 50 | http://www.genecards.org/cgi-bin/carddisp.pl?gene=ANKRD50 | 3.427040836 | 0.079196017 |
| *ANO2* | Anoctamin-2 | http://www.genecards.org/cgi-bin/carddisp.pl?gene=ANO2 | 0.245054158 | 0.057355469 |
| *ANXA2* | Annexin A2 | http://www.genecards.org/cgi-bin/carddisp.pl?gene=ANXA2 | 0.850707431 | 0.053282854 |
| *ANXA5* | Annexin A5 | http://www.genecards.org/cgi-bin/carddisp.pl?gene=ANXA5 | 1.674360351 | 0.14914375 |
| *AP2A1* | AP-2 complex subunit alpha-1 | http://www.genecards.org/cgi-bin/carddisp.pl?gene=AP2A1 | 1.06144425 | 0.284070453 |
| *AP2B1* | AP-2 complex subunit beta | http://www.genecards.org/cgi-bin/carddisp.pl?gene=AP2B1 | 0.877800454 | 0.096851496 |
| *AP2M1* | AP-2 complex subunit mu | http://www.genecards.org/cgi-bin/carddisp.pl?gene=AP2M1 | 1.002223387 | 0.494362398 |
| *AP2S1* | AP-2 complex subunit sigma | http://www.genecards.org/cgi-bin/carddisp.pl?gene=AP2S1 | 0.753237131 | 0.05910732 |
| *AP3B1* | AP-3 complex subunit beta-1 | http://www.genecards.org/cgi-bin/carddisp.pl?gene=AP3B1 | 10.80243631 | 0.177140895 |
| *AP5M1* | AP-5 complex subunit mu-1 | http://www.genecards.org/cgi-bin/carddisp.pl?gene=AP5M1 | 0.659716245 | 0.05770151 |
| *APC2* | Adenomatous polyposis coli protein 2 | http://www.genecards.org/cgi-bin/carddisp.pl?gene=APC2 | 0.718238168 | 0.05282131 |
| *APOA1* | Apolipoprotein A-I | http://www.genecards.org/cgi-bin/carddisp.pl?gene=APOA1 | 0.839580876 | 0.333236791 |
| *APOA4* | Apolipoprotein A-IV | http://www.genecards.org/cgi-bin/carddisp.pl?gene=APOA4 | 0.536184532 | 0.312978568 |
| *ARF4* | ADP-ribosylation factor 4 | http://www.genecards.org/cgi-bin/carddisp.pl?gene=ARF4 | 0.414385589 | 0.150540341 |
| *ARFIP1* | Arfaptin-1 | http://www.genecards.org/cgi-bin/carddisp.pl?gene=ARFIP1 | 1.406171879 | 0.347796372 |
| *ARG1* | Arginase-1 | http://www.genecards.org/cgi-bin/carddisp.pl?gene=ARG1 | 0.093030493 | 0.140024911 |
| *ARHGDIA* | Rho GDP-dissociation inhibitor 1 | http://www.genecards.org/cgi-bin/carddisp.pl?gene=ARHGDIA | 0.715633881 | 0.087013076 |
| *ARL6IP1* | ADP-ribosylation factor-like protein 6-interacting protein 1 | http://www.genecards.org/cgi-bin/carddisp.pl?gene=ARL6IP1 | 5.76543234 | 0.059002101 |
| *ARL6IP5* | PRA1 family protein 3 | http://www.genecards.org/cgi-bin/carddisp.pl?gene=ARL6IP5 | 0.662291055 | 0.149039812 |
| *ATP1A2* | Sodium/potassium-transporting ATPase subunit alpha-2 | http://www.genecards.org/cgi-bin/carddisp.pl?gene=ATP1A2 | 0.835007913 | 0.199413167 |
| *ATP1A3* | Sodium/potassium-transporting ATPase subunit alpha-3 | http://www.genecards.org/cgi-bin/carddisp.pl?gene=ATP1A3 | 1.575848025 | 0.058796766 |
| *ATP1B1* | Sodium/potassium-transporting ATPase subunit beta-1 | http://www.genecards.org/cgi-bin/carddisp.pl?gene=ATP1B1 | 1.122192437 | 0.338364879 |
| *ATP2A1* | Sarcoplasmic/endoplasmic reticulum calcium ATPase 1 | http://www.genecards.org/cgi-bin/carddisp.pl?gene=ATP2A1 | 1.197790685 | 0.156693653 |
| *ATP2B1* | Plasma membrane calcium-transporting ATPase 1 | http://www.genecards.org/cgi-bin/carddisp.pl?gene=ATP2B1 | 0.726780876 | 0.057466299 |
| *ATP2B4* | Plasma membrane calcium-transporting ATPase 4 | http://www.genecards.org/cgi-bin/carddisp.pl?gene=ATP2B4 | 1.18044718 | 0.121833133 |
| *ATP4A* | Potassium-transporting ATPase alpha chain 1 | http://www.genecards.org/cgi-bin/carddisp.pl?gene=ATP4A | 1.067311416 | 0.314915803 |
| *ATP6V0A4* | V-type proton ATPase 116 kDa subunit a isoform 4 | http://www.genecards.org/cgi-bin/carddisp.pl?gene=ATP6V0A4 | 1.128431521 | 0.44381957 |
| *ATP7A* | Copper-transporting ATPase 1 | http://www.genecards.org/cgi-bin/carddisp.pl?gene=ATP7A | 0.682377664 | 0.126022403 |
| *ATRX* | Transcriptional regulator ATRX | http://www.genecards.org/cgi-bin/carddisp.pl?gene=ATRX | 0.946630297 | 0.319167569 |
| *AXL* | Tyrosine-protein kinase receptor UFO | http://www.genecards.org/cgi-bin/carddisp.pl?gene=AXL | 0.938293299 | 0.407977197 |
| *AZGP1* | Zinc-alpha-2-glycoprotein | http://www.genecards.org/cgi-bin/carddisp.pl?gene=AZGP1 | 0.349626424 | 0.065155088 |
| *BAZ1A* | Bromodomain adjacent to zinc finger domain protein 1A | http://www.genecards.org/cgi-bin/carddisp.pl?gene=BAZ1A | 0.448878678 | 0.052323205 |
| *BAZ2B* | Bromodomain adjacent to zinc finger domain protein 2B | http://www.genecards.org/cgi-bin/carddisp.pl?gene=BAZ2B | 0.545869322 | 0.063647938 |
| *BCAP31* | B-cell receptor-associated protein 31 | http://www.genecards.org/cgi-bin/carddisp.pl?gene=BCAP31 | 1.242167439 | 0.312539653 |
| *BDNF* | Brain-derived neurotrophic factor | http://www.genecards.org/cgi-bin/carddisp.pl?gene=BDNF | 0.416618285 | 0.077119767 |
| *BDP1* | Transcription factor TFIIIB component B'' homolog | http://www.genecards.org/cgi-bin/carddisp.pl?gene=BDP1 | 1.147739252 | 0.183869594 |
| *BEND7* | BEN domain-containing protein 7 | http://www.genecards.org/cgi-bin/carddisp.pl?gene=BEND7 | 0.179853343 | 0.147730373 |
| *BLK* | Tyrosine-protein kinase Blk | http://www.genecards.org/cgi-bin/carddisp.pl?gene=BLK | 0.398250237 | 0.143373997 |
| *BLVRB* | Flavin reductase (NADPH) | http://www.genecards.org/cgi-bin/carddisp.pl?gene=BLVRB | 1.441750157 | 0.229731805 |
| *BRWD3* | Bromodomain and WD repeat-containing protein 3 | http://www.genecards.org/cgi-bin/carddisp.pl?gene=BRWD3 | 1.169406091 | 0.41203739 |
| *BTBD10* | BTB/POZ domain-containing protein 10 | http://www.genecards.org/cgi-bin/carddisp.pl?gene=BTBD10 | 0.407704091 | 0.056579854 |
| *BTF3* | Transcription factor BTF3 | http://www.genecards.org/cgi-bin/carddisp.pl?gene=BTF3 | 1.045288829 | 0.445126084 |
| *C1QBP* | Complement component 1 Q subcomponent-binding protein, mitochondrial | http://www.genecards.org/cgi-bin/carddisp.pl?gene=C1QBP | 0.564760274 | 0.186001436 |
| *C1QTNF3* | Complement C1q tumor necrosis factor-related protein 3 | http://www.genecards.org/cgi-bin/carddisp.pl?gene=C1QTNF3 | 1.329299641 | 0.277578889 |
| *C20orf27* | UPF0687 protein C20orf27 | http://www.genecards.org/cgi-bin/carddisp.pl?gene=C20orf27 | 0.707625533 | 0.223385986 |
| *C2orf42* | Uncharacterized protein C2orf42 | http://www.genecards.org/cgi-bin/carddisp.pl?gene=C2orf42 | 1.145324814 | 0.347239539 |
| *C3* | Complement C3 | http://www.genecards.org/cgi-bin/carddisp.pl?gene=C3 | 0.962276784 | 0.424868433 |
| *C4A* | Complement C4-A | http://www.genecards.org/cgi-bin/carddisp.pl?gene=C4A | 0.784408969 | 0.240669577 |
| *C9* | Complement component C9 | http://www.genecards.org/cgi-bin/carddisp.pl?gene=C9 | 1.074208081 | 0.446015958 |
| *CACNA2D1* | Voltage-dependent calcium channel subunit alpha-2/delta-1 | http://www.genecards.org/cgi-bin/carddisp.pl?gene=CACNA2D1 | 2.763714381 | 0.078956502 |
| *CALML5* | Calmodulin-like protein 5 | http://www.genecards.org/cgi-bin/carddisp.pl?gene=CALML5 | 0.445239882 | 0.112668163 |
| *CALU* | Calumenin | http://www.genecards.org/cgi-bin/carddisp.pl?gene=CALU | 0.834592719 | 0.217607438 |
| *CAMTA1* | Calmodulin-binding transcription activator 1 | http://www.genecards.org/cgi-bin/carddisp.pl?gene=CAMTA1 | 0.069538857 | 0.171746011 |
| *CAP1* | Adenylyl cyclase-associated protein 1 | http://www.genecards.org/cgi-bin/carddisp.pl?gene=CAP1 | 0.853036717 | 0.354361347 |
| *CAPRIN1* | Caprin-1 | http://www.genecards.org/cgi-bin/carddisp.pl?gene=CAPRIN1 | 0.795523087 | 0.050681669 |
| *CAPZA1* | F-actin-capping protein subunit alpha-1 | http://www.genecards.org/cgi-bin/carddisp.pl?gene=CAPZA1 | 1.054097916 | 0.445623628 |
| *CASK* | Peripheral plasma membrane protein CASK | http://www.genecards.org/cgi-bin/carddisp.pl?gene=CASK | 1.509845016 | 0.055757558 |
| *CASP14* | Caspase-14 | http://www.genecards.org/cgi-bin/carddisp.pl?gene=CASP14 | 0.487766862 | 0.089677934 |
| *CC2D1A* | Coiled-coil and C2 domain-containing protein 1A | http://www.genecards.org/cgi-bin/carddisp.pl?gene=CC2D1A | 0.990441392 | 0.488812198 |
| *CCDC14* | Coiled-coil domain-containing protein 14 | http://www.genecards.org/cgi-bin/carddisp.pl?gene=CCDC14 | 0.801371921 | 0.182452473 |
| *CCDC50* | Coiled-coil domain-containing protein 50 | http://www.genecards.org/cgi-bin/carddisp.pl?gene=CCDC50 | 1.068630215 | 0.279590083 |
| *CCDC62* | Coiled-coil domain-containing protein 62 | http://www.genecards.org/cgi-bin/carddisp.pl?gene=CCDC62 | 0.324191016 | 0.13341855 |
| *CCDC96* | Coiled-coil domain-containing protein 96 | http://www.genecards.org/cgi-bin/carddisp.pl?gene=CCDC96 | 0.2326365 | 0.183947024 |
| *CCIN* | Calicin | http://www.genecards.org/cgi-bin/carddisp.pl?gene=CCIN | 1.170562795 | 0.211694096 |
| *CD101* | Immunoglobulin superfamily member 2 | http://www.genecards.org/cgi-bin/carddisp.pl?gene=CD101 | 1.42662414 | 0.237078619 |
| *CD109* | CD109 antigen | http://www.genecards.org/cgi-bin/carddisp.pl?gene=CD109 | 2.551947849 | 0.120064203 |
| *CD151* | CD151 antigen | http://www.genecards.org/cgi-bin/carddisp.pl?gene=CD151 | 1.929684478 | 0.196513267 |
| *CD247* | T-cell surface glycoprotein CD3 zeta chain | http://www.genecards.org/cgi-bin/carddisp.pl?gene=CD247 | 0.78833787 | 0.183178683 |
| *CD44* | CD44 antigen | http://www.genecards.org/cgi-bin/carddisp.pl?gene=CD44 | 1.113115076 | 0.136652247 |
| *CD82* | CD82 antigen | http://www.genecards.org/cgi-bin/carddisp.pl?gene=CD82 | 1.229702181 | 0.369956416 |
| *CD9* | CD9 antigen | http://www.genecards.org/cgi-bin/carddisp.pl?gene=CD9 | 1.00951871 | 0.453317416 |
| *CD97* | CD97 antigen | http://www.genecards.org/cgi-bin/carddisp.pl?gene=CD97 | 3.367969218 | 0.084833094 |
| *CD99* | CD99 antigen | http://www.genecards.org/cgi-bin/carddisp.pl?gene=CD99 | 1.022067595 | 0.452266376 |
| *CD99L2* | CD99 antigen-like protein 2 | http://www.genecards.org/cgi-bin/carddisp.pl?gene=CD99L2 | 1.628401943 | 0.141007595 |
| *CDC42* | Cell division control protein 42 homolog | http://www.genecards.org/cgi-bin/carddisp.pl?gene=CDC42 | 2.256596417 | 0.15347705 |
| *CDC42EP1* | Cdc42 effector protein 1 | http://www.genecards.org/cgi-bin/carddisp.pl?gene=CDC42EP1 | 2.826868272 | 0.101843016 |
| *CDC42EP4* | Cdc42 effector protein 4 | http://www.genecards.org/cgi-bin/carddisp.pl?gene=CDC42EP4 | 1.495492304 | 0.263446814 |
| *CDH6* | Cadherin-6 | http://www.genecards.org/cgi-bin/carddisp.pl?gene=CDH6 | 0.797324527 | 0.235206371 |
| *CDK8* | Cyclin-dependent kinase 8 | http://www.genecards.org/cgi-bin/carddisp.pl?gene=CDK8 | 0.525183316 | 0.060145617 |
| *CDT1* | DNA replication factor Cdt1 | http://www.genecards.org/cgi-bin/carddisp.pl?gene=CDT1 | 0.815086908 | 0.357244166 |
| *CELSR1* | Cadherin EGF LAG seven-pass G-type receptor 1 | http://www.genecards.org/cgi-bin/carddisp.pl?gene=CELSR1 | 5.650599272 | 0.086658581 |
| *CEP192* | Centrosomal protein of 192 kDa | http://www.genecards.org/cgi-bin/carddisp.pl?gene=CEP192 | 0.8450129 | 0.271032716 |
| *CFL1* | Cofilin-1 | http://www.genecards.org/cgi-bin/carddisp.pl?gene=CFL1 | 1.610261551 | 0.178227919 |
| *CFL2* | Cofilin-2 | http://www.genecards.org/cgi-bin/carddisp.pl?gene=CFL2 | 1.112208684 | 0.373894901 |
| *CHMP4B* | Charged multivesicular body protein 4b | http://www.genecards.org/cgi-bin/carddisp.pl?gene=CHMP4B | 1.165156224 | 0.213122267 |
| *CKAP5* | Cytoskeleton-associated protein 5 | http://www.genecards.org/cgi-bin/carddisp.pl?gene=CKAP5 | 0.686804299 | 0.158210251 |
| *CLASP1* | CLIP-associating protein 1 | http://www.genecards.org/cgi-bin/carddisp.pl?gene=CLASP1 | 0.779509808 | 0.226587445 |
| *CLIC1* | Chloride intracellular channel protein 1 | http://www.genecards.org/cgi-bin/carddisp.pl?gene=CLIC1 | 0.80174167 | 0.159840581 |
| *CLIC4* | Chloride intracellular channel protein 4 | http://www.genecards.org/cgi-bin/carddisp.pl?gene=CLIC4 | 0.939859132 | 0.382680696 |
| *CLMP* | CXADR-like membrane protein | http://www.genecards.org/cgi-bin/carddisp.pl?gene=CLMP | 1.768282054 | 0.061303012 |
| *CLTA* | Clathrin light chain A | http://www.genecards.org/cgi-bin/carddisp.pl?gene=CLTA | 0.84585009 | 0.147371511 |
| *CMYA5* | Cardiomyopathy-associated protein 5 | http://www.genecards.org/cgi-bin/carddisp.pl?gene=CMYA5 | 0.488428994 | 0.164237387 |
| *CNGB1* | Cyclic nucleotide-gated cation channel beta-1 | http://www.genecards.org/cgi-bin/carddisp.pl?gene=CNGB1 | 0.333205665 | 0.062000299 |
| *CNP* | 2',3'-cyclic-nucleotide 3'-phosphodiesterase | http://www.genecards.org/cgi-bin/carddisp.pl?gene=CNP | 1.625090752 | 0.1278392 |
| *CNTN3* | Contactin-3 | http://www.genecards.org/cgi-bin/carddisp.pl?gene=CNTN3 | 0.897407836 | 0.423467109 |
| *COBL* | Protein cordon-bleu | http://www.genecards.org/cgi-bin/carddisp.pl?gene=COBL | 1.112006974 | 0.41000028 |
| *COL12A1* | Collagen alpha-1(XII) chain | http://www.genecards.org/cgi-bin/carddisp.pl?gene=COL12A1 | 0.513281561 | 0.204553808 |
| *COL17A1* | Collagen alpha-1(XVII) chain | http://www.genecards.org/cgi-bin/carddisp.pl?gene=COL17A1 | 0.292260279 | 0.217614385 |
| *COL4A1* | Collagen alpha-1(IV) chain | http://www.genecards.org/cgi-bin/carddisp.pl?gene=COL4A1 | 4.772717652 | 0.101820159 |
| *COL4A2* | Collagen alpha-2(IV) chain | http://www.genecards.org/cgi-bin/carddisp.pl?gene=COL4A2 | 0.866819354 | 0.31986542 |
| *COL6A1* | Collagen alpha-1(VI) chain | http://www.genecards.org/cgi-bin/carddisp.pl?gene=COL6A1 | 0.524940644 | 0.13267065 |
| *COL6A2* | Collagen alpha-2(VI) chain | http://www.genecards.org/cgi-bin/carddisp.pl?gene=COL6A2 | 2.159652335 | 0.063949246 |
| *COTL1* | Coactosin-like protein | http://www.genecards.org/cgi-bin/carddisp.pl?gene=COTL1 | 1.499018878 | 0.085925628 |
| *CPLX1* | Complexin-1 | http://www.genecards.org/cgi-bin/carddisp.pl?gene=CPLX1 | 1.060736182 | 0.266517052 |
| *CPNE2* | Copine-2 | http://www.genecards.org/cgi-bin/carddisp.pl?gene=CPNE2 | 0.87231913 | 0.388651208 |
| *CPNE3* | Copine-3 | http://www.genecards.org/cgi-bin/carddisp.pl?gene=CPNE3 | 0.488860605 | 0.132820503 |
| *CPSF1* | Cleavage and polyadenylation specificity factor subunit 1 | http://www.genecards.org/cgi-bin/carddisp.pl?gene=CPSF1 | 3.267328568 | 0.166540796 |
| *CR1* | Complement receptor type 1 | http://www.genecards.org/cgi-bin/carddisp.pl?gene=CR1 | 0.993165579 | 0.483730454 |
| *CRACR2A* | EF-hand calcium-binding domain-containing protein 4B | http://www.genecards.org/cgi-bin/carddisp.pl?gene=CRACR2A | 0.380715555 | 0.256703454 |
| *CREB5* | Cyclic AMP-responsive element-binding protein 5 | http://www.genecards.org/cgi-bin/carddisp.pl?gene=CREB5 | 1.018840066 | 0.479122509 |
| *CRLF3* | Cytokine receptor-like factor 3 | http://www.genecards.org/cgi-bin/carddisp.pl?gene=CRLF3 | 1.21650571 | 0.260740735 |
| *CROCC* | Rootletin | http://www.genecards.org/cgi-bin/carddisp.pl?gene=CROCC | 0.81453803 | 0.131080263 |
| *CSF3R* | Granulocyte colony-stimulating factor receptor | http://www.genecards.org/cgi-bin/carddisp.pl?gene=CSF3R | 1.088460219 | 0.411767605 |
| *CSRP1* | Cysteine and glycine-rich protein 1 | http://www.genecards.org/cgi-bin/carddisp.pl?gene=CSRP1 | 1.086119487 | 0.305481319 |
| *CSRP2* | Cysteine and glycine-rich protein 2 | http://www.genecards.org/cgi-bin/carddisp.pl?gene=CSRP2 | 1.389831647 | 0.074836394 |
| *CSTA* | Cystatin-A | http://www.genecards.org/cgi-bin/carddisp.pl?gene=CSTA | 0.35875744 | 0.09427734 |
| *CTNNB1* | Catenin beta-1 | http://www.genecards.org/cgi-bin/carddisp.pl?gene=CTNNB1 | 0.817738583 | 0.146555264 |
| *CTTNBP2* | Cortactin-binding protein 2 | http://www.genecards.org/cgi-bin/carddisp.pl?gene=CTTNBP2 | 0.9590645 | 0.448657749 |
| *CTTNBP2NL* | CTTNBP2 N-terminal-like protein | http://www.genecards.org/cgi-bin/carddisp.pl?gene=CTTNBP2NL | 0.634394249 | 0.301154777 |
| *CUTA* | Protein CutA | http://www.genecards.org/cgi-bin/carddisp.pl?gene=CUTA | 1.250744161 | 0.222534892 |
| *CWC22* | Pre-mRNA-splicing factor CWC22 homolog | http://www.genecards.org/cgi-bin/carddisp.pl?gene=CWC22 | 0.669649019 | 0.207118655 |
| *CYFIP1* | Cytoplasmic FMR1-interacting protein 1 | http://www.genecards.org/cgi-bin/carddisp.pl?gene=CYFIP1 | 1.296248928 | 0.138706943 |
| *CYR61* | Protein CYR61 | http://www.genecards.org/cgi-bin/carddisp.pl?gene=CYR61 | 1.047373364 | 0.36577325 |
| *DAB2* | Disabled homolog 2 | http://www.genecards.org/cgi-bin/carddisp.pl?gene=DAB2 | 1.052865635 | 0.431034897 |
| *DCAF12L2* | DDB1- and CUL4-associated factor 12-like protein 2 | http://www.genecards.org/cgi-bin/carddisp.pl?gene=DCAF12L2 | 0.852152625 | 0.417178587 |
| *DCST2* | DC-STAMP domain-containing protein 2 | http://www.genecards.org/cgi-bin/carddisp.pl?gene=DCST2 | 1.303250774 | 0.272522852 |
| *DDR2* | Discoidin domain-containing receptor 2 | http://www.genecards.org/cgi-bin/carddisp.pl?gene=DDR2 | 1.411460334 | 0.124171812 |
| *DDX46* | Probable ATP-dependent RNA helicase DDX46 | http://www.genecards.org/cgi-bin/carddisp.pl?gene=DDX46 | 0.718495212 | 0.162661124 |
| *DENND6A* | Protein DENND6A | http://www.genecards.org/cgi-bin/carddisp.pl?gene=DENND6A | 0.806712955 | 0.154720753 |
| *DERL2* | Derlin-2 | http://www.genecards.org/cgi-bin/carddisp.pl?gene=DERL2 | 3.109520587 | 0.156546342 |
| *DES* | Desmin | http://www.genecards.org/cgi-bin/carddisp.pl?gene=DES | 0.82354958 | 0.275481067 |
| *DIAPH1* | Protein diaphanous homolog 1 | http://www.genecards.org/cgi-bin/carddisp.pl?gene=DIAPH1 | 0.111808171 | 0.163465883 |
| *DNAH2* | Dynein heavy chain 2, axonemal | http://www.genecards.org/cgi-bin/carddisp.pl?gene=DNAH2 | 1.224154482 | 0.307074328 |
| *DNAH8* | Dynein heavy chain 8, axonemal | http://www.genecards.org/cgi-bin/carddisp.pl?gene=DNAH8 | 1.370347019 | 0.19674605 |
| *DNAJA2* | DnaJ homolog subfamily A member 2 | http://www.genecards.org/cgi-bin/carddisp.pl?gene=DNAJA2 | 0.839138393 | 0.318009665 |
| *DNER* | Delta and Notch-like epidermal growth factor-related receptor | http://www.genecards.org/cgi-bin/carddisp.pl?gene=DNER | 0.748742369 | 0.054449803 |
| *DNHD1* | Dynein heavy chain domain-containing protein 1 | http://www.genecards.org/cgi-bin/carddisp.pl?gene=DNHD1 | 0.492205006 | 0.205323405 |
| *DNM2* | Dynamin-2 | http://www.genecards.org/cgi-bin/carddisp.pl?gene=DNM2 | 0.981028349 | 0.469875496 |
| *DNMT1* | DNA (cytosine-5)-methyltransferase 1 | http://www.genecards.org/cgi-bin/carddisp.pl?gene=DNMT1 | 0.556121214 | 0.182091641 |
| *DOPEY1* | Protein dopey-1 | http://www.genecards.org/cgi-bin/carddisp.pl?gene=DOPEY1 | 0.278519479 | 0.084538149 |
| *DPYSL2* | Dihydropyrimidinase-related protein 2 | http://www.genecards.org/cgi-bin/carddisp.pl?gene=DPYSL2 | 1.284016689 | 0.241718225 |
| *DRG1* | Developmentally-regulated GTP-binding protein 1 | http://www.genecards.org/cgi-bin/carddisp.pl?gene=DRG1 | 0.793360329 | 0.068192955 |
| *DSC1* | Desmocollin-1 | http://www.genecards.org/cgi-bin/carddisp.pl?gene=DSC1 | 0.542477798 | 0.191293817 |
| *DSC2* | Desmocollin-2 | http://www.genecards.org/cgi-bin/carddisp.pl?gene=DSC2 | 0.634132492 | 0.098173308 |
| *DSG1* | Desmoglein-1 | http://www.genecards.org/cgi-bin/carddisp.pl?gene=DSG1 | 0.392786464 | 0.109162358 |
| *DUOX2* | Dual oxidase 2 | http://www.genecards.org/cgi-bin/carddisp.pl?gene=DUOX2 | 0.200381477 | 0.143065607 |
| *DYNC1H1* | Cytoplasmic dynein 1 heavy chain 1 | http://www.genecards.org/cgi-bin/carddisp.pl?gene=DYNC1H1 | 1.106880073 | 0.387302357 |
| *DYSF* | Dysferlin | http://www.genecards.org/cgi-bin/carddisp.pl?gene=DYSF | 1.126698721 | 0.350462436 |
| *E2F7* | Transcription factor E2F7 | http://www.genecards.org/cgi-bin/carddisp.pl?gene=E2F7 | 1.160033789 | 0.45042522 |
| *EDIL3* | EGF-like repeat and discoidin I-like domain-containing protein 3 | http://www.genecards.org/cgi-bin/carddisp.pl?gene=EDIL3 | 0.586842028 | 0.110496239 |
| *EEF1A1* | Elongation factor 1-alpha 1 | http://www.genecards.org/cgi-bin/carddisp.pl?gene=EEF1A1 | 0.939422468 | 0.413505173 |
| *EEF1B2* | Elongation factor 1-beta | http://www.genecards.org/cgi-bin/carddisp.pl?gene=EEF1B2 | 1.11499496 | 0.338438574 |
| *EEF1D* | Elongation factor 1-delta | http://www.genecards.org/cgi-bin/carddisp.pl?gene=EEF1D | 1.215665503 | 0.113901304 |
| *EEF2* | Elongation factor 2 | http://www.genecards.org/cgi-bin/carddisp.pl?gene=EEF2 | 1.042965924 | 0.33252047 |
| *EFHD1* | EF-hand domain-containing protein D1 | http://www.genecards.org/cgi-bin/carddisp.pl?gene=EFHD1 | 0.575737704 | 0.082996753 |
| *EFNB1* | Ephrin-B1 | http://www.genecards.org/cgi-bin/carddisp.pl?gene=EFNB1 | 64.22777469 | 0.084824878 |
| *EHBP1L1* | EH domain-binding protein 1-like protein 1 | http://www.genecards.org/cgi-bin/carddisp.pl?gene=EHBP1L1 | 1.327766125 | 0.21698997 |
| *EHD1* | EH domain-containing protein 1 | http://www.genecards.org/cgi-bin/carddisp.pl?gene=EHD1 | 0.920431558 | 0.336802396 |
| *EHD2* | EH domain-containing protein 2 | http://www.genecards.org/cgi-bin/carddisp.pl?gene=EHD2 | 1.047624925 | 0.33644774 |
| *EHD3* | EH domain-containing protein 3 | http://www.genecards.org/cgi-bin/carddisp.pl?gene=EHD3 | 1.599548544 | 0.20873499 |
| *EIF1AX* | Eukaryotic translation initiation factor 1A, X-chromosomal | http://www.genecards.org/cgi-bin/carddisp.pl?gene=EIF1AX | 1.541239839 | 0.181228555 |
| *EIF2S2* | Eukaryotic translation initiation factor 2 subunit 2 | http://www.genecards.org/cgi-bin/carddisp.pl?gene=EIF2S2 | 1.137285649 | 0.209342574 |
| *EIF3G* | Eukaryotic translation initiation factor 3 subunit G | http://www.genecards.org/cgi-bin/carddisp.pl?gene=EIF3G | 0.805677453 | 0.290535782 |
| *EIF4G1* | Eukaryotic translation initiation factor 4 gamma 1 | http://www.genecards.org/cgi-bin/carddisp.pl?gene=EIF4G1 | 0.999814899 | 0.499528005 |
| *EML6* | Echinoderm microtubule-associated protein-like 6 | http://www.genecards.org/cgi-bin/carddisp.pl?gene=EML6 | 0.536058407 | 0.188545467 |
| *EPB41L3* | Band 4.1-like protein 3 | http://www.genecards.org/cgi-bin/carddisp.pl?gene=EPB41L3 | 0.978479119 | 0.437350373 |
| *EPHA3* | Ephrin type-A receptor 3 | http://www.genecards.org/cgi-bin/carddisp.pl?gene=EPHA3 | 0.806738651 | 0.105800218 |
| *EPHA6* | Ephrin type-A receptor 6 | http://www.genecards.org/cgi-bin/carddisp.pl?gene=EPHA6 | 0.894835899 | 0.296812272 |
| *EPS8* | Epidermal growth factor receptor kinase substrate 8 | http://www.genecards.org/cgi-bin/carddisp.pl?gene=EPS8 | 0.853060016 | 0.205968504 |
| *ERBB2* | Receptor tyrosine-protein kinase erbB-2 | http://www.genecards.org/cgi-bin/carddisp.pl?gene=ERBB2 | 1.180075448 | 0.224977397 |
| *ESR2* | Estrogen receptor beta | http://www.genecards.org/cgi-bin/carddisp.pl?gene=ESR2 | 0.957286232 | 0.479382213 |
| *ESYT2* | Extended synaptotagmin-2 | http://www.genecards.org/cgi-bin/carddisp.pl?gene=ESYT2 | 1.284115414 | 0.135273167 |
| *EVA1B* | Protein eva-1 homolog B | http://www.genecards.org/cgi-bin/carddisp.pl?gene=EVA1B | 2.616030061 | 0.132323791 |
| *EXOC8* | Exocyst complex component 8 | http://www.genecards.org/cgi-bin/carddisp.pl?gene=EXOC8 | 0.615230227 | 0.188304386 |
| *EXTL1* | Exostosin-like 1 | http://www.genecards.org/cgi-bin/carddisp.pl?gene=EXTL1 | 1.027898412 | 0.474479044 |
| *EZR* | Ezrin | http://www.genecards.org/cgi-bin/carddisp.pl?gene=EZR | 1.144340198 | 0.087557122 |
| *F10* | Coagulation factor X | http://www.genecards.org/cgi-bin/carddisp.pl?gene=F10 | 1.117065303 | 0.221044179 |
| *F13A1* | Coagulation factor XIII A chain | http://www.genecards.org/cgi-bin/carddisp.pl?gene=F13A1 | 1.070745049 | 0.407108261 |
| *F8* | Coagulation factor VIII | http://www.genecards.org/cgi-bin/carddisp.pl?gene=F8 | 0.84181007 | 0.122256637 |
| *FABP5* | Fatty acid-binding protein, epidermal | http://www.genecards.org/cgi-bin/carddisp.pl?gene=FABP5 | 0.316639374 | 0.122237051 |
| *FAM117A* | Protein FAM117A | http://www.genecards.org/cgi-bin/carddisp.pl?gene=FAM117A | 0.925856908 | 0.261972995 |
| *FAM120C* | Constitutive coactivator of PPAR-gamma-like protein 2 | http://www.genecards.org/cgi-bin/carddisp.pl?gene=FAM120C | 0.989489777 | 0.49063828 |
| *FAM129B* | Niban-like protein 1 | http://www.genecards.org/cgi-bin/carddisp.pl?gene=FAM129B | 2.349603742 | 0.075272696 |
| *FAM160A1* | Protein FAM160A1 | http://www.genecards.org/cgi-bin/carddisp.pl?gene=FAM160A1 | 1.458807799 | 0.188675697 |
| *FAM160B2* | Protein FAM160B2 | http://www.genecards.org/cgi-bin/carddisp.pl?gene=FAM160B2 | 0.760711363 | 0.106947851 |
| *FAM186A* | Protein FAM186A | http://www.genecards.org/cgi-bin/carddisp.pl?gene=FAM186A | 1.637875078 | 0.121192627 |
| *FAM49B* | Protein FAM49B | http://www.genecards.org/cgi-bin/carddisp.pl?gene=FAM49B | 1.076218817 | 0.433596105 |
| *FANK1* | Fibronectin type 3 and ankyrin repeat domains protein 1 | http://www.genecards.org/cgi-bin/carddisp.pl?gene=FANK1 | 1.047139317 | 0.468221368 |
| *FAS* | Tumor necrosis factor receptor superfamily member 6 | http://www.genecards.org/cgi-bin/carddisp.pl?gene=FAS | 1.265031647 | 0.189670495 |
| *FBN1* | Fibrillin-1 | http://www.genecards.org/cgi-bin/carddisp.pl?gene=FBN1 | 0.801428339 | 0.182698634 |
| *FBN2* | Fibrillin-2 | http://www.genecards.org/cgi-bin/carddisp.pl?gene=FBN2 | 1.820233533 | 0.058351917 |
| *FCHO1* | F-BAR domain only protein 1 | http://www.genecards.org/cgi-bin/carddisp.pl?gene=FCHO1 | 1.338264865 | 0.156560155 |
| *FCHSD2* | F-BAR and double SH3 domains protein 2 | http://www.genecards.org/cgi-bin/carddisp.pl?gene=FCHSD2 | 0.814049202 | 0.354484475 |
| *FGD4* | FYVE, RhoGEF and PH domain-containing protein 4 | http://www.genecards.org/cgi-bin/carddisp.pl?gene=FGD4 | 0.737295706 | 0.142279224 |
| *FGFRL1* | Fibroblast growth factor receptor-like 1 | http://www.genecards.org/cgi-bin/carddisp.pl?gene=FGFRL1 | 0.384952622 | 0.083427047 |
| *FH* | Fumarate hydratase, mitochondrial | http://www.genecards.org/cgi-bin/carddisp.pl?gene=FH | 1.774952199 | 0.168399541 |
| *FHL1* | Four and a half LIM domains protein 1 | http://www.genecards.org/cgi-bin/carddisp.pl?gene=FHL1 | 1.107866478 | 0.383804624 |
| *FHOD1* | FH1/FH2 domain-containing protein 1 | http://www.genecards.org/cgi-bin/carddisp.pl?gene=FHOD1 | 2.047409039 | 0.09550554 |
| *FKBP1A* | Peptidyl-prolyl cis-trans isomerase FKBP1A | http://www.genecards.org/cgi-bin/carddisp.pl?gene=FKBP1A | 0.64075169 | 0.052566198 |
| *FKBP3* | Peptidyl-prolyl cis-trans isomerase FKBP3 | http://www.genecards.org/cgi-bin/carddisp.pl?gene=FKBP3 | 0.756554023 | 0.182531106 |
| *FLG2* | Filaggrin-2 | http://www.genecards.org/cgi-bin/carddisp.pl?gene=FLG2 | 0.910300002 | 0.332124265 |
| *FMN2* | Formin-2 | http://www.genecards.org/cgi-bin/carddisp.pl?gene=FMN2 | 2.956565297 | 0.174948356 |
| *FNDC1* | Fibronectin type III domain-containing protein 1 | http://www.genecards.org/cgi-bin/carddisp.pl?gene=FNDC1 | 0.366402759 | 0.076661956 |
| *FSIP2* | Fibrous sheath-interacting protein 2 | http://www.genecards.org/cgi-bin/carddisp.pl?gene=FSIP2 | 1.083983447 | 0.431329305 |
| *FST* | Follistatin | http://www.genecards.org/cgi-bin/carddisp.pl?gene=FST | 0.885936797 | 0.436800161 |
| *G3BP1* | Ras GTPase-activating protein-binding protein 1 | http://www.genecards.org/cgi-bin/carddisp.pl?gene=G3BP1 | 1.536215763 | 0.108903504 |
| *G6PD* | Glucose-6-phosphate 1-dehydrogenase | http://www.genecards.org/cgi-bin/carddisp.pl?gene=G6PD | 2.234641849 | 0.186593412 |
| *GALNT12* | Polypeptide N-acetylgalactosaminyltransferase 12 | http://www.genecards.org/cgi-bin/carddisp.pl?gene=GALNT12 | 3.313656072 | 0.094676225 |
| *GAPDH* | Glyceraldehyde-3-phosphate dehydrogenase | http://www.genecards.org/cgi-bin/carddisp.pl?gene=GAPDH | 1.125699658 | 0.240386103 |
| *GDF5* | Growth/differentiation factor 5 | http://www.genecards.org/cgi-bin/carddisp.pl?gene=GDF5 | 1.199429196 | 0.282924851 |
| *GGT2* | Inactive gamma-glutamyltranspeptidase 2 | http://www.genecards.org/cgi-bin/carddisp.pl?gene=GGT2 | 0.735381073 | 0.083079925 |
| *GIT1* | ARF GTPase-activating protein GIT1 | http://www.genecards.org/cgi-bin/carddisp.pl?gene=GIT1 | 1.552439902 | 0.074508849 |
| *GLIPR1* | Glioma pathogenesis-related protein 1 | http://www.genecards.org/cgi-bin/carddisp.pl?gene=GLIPR1 | 0.786110448 | 0.370530882 |
| *GMFB* | Glia maturation factor beta | http://www.genecards.org/cgi-bin/carddisp.pl?gene=GMFB | 1.048695159 | 0.435503647 |
| *GNA11* | Guanine nucleotide-binding protein subunit alpha-11 | http://www.genecards.org/cgi-bin/carddisp.pl?gene=GNA11 | 1.380787235 | 0.094194649 |
| *GNA12* | Guanine nucleotide-binding protein subunit alpha-12 | http://www.genecards.org/cgi-bin/carddisp.pl?gene=GNA12 | 0.47696012 | 0.067794012 |
| *GNAO1* | Guanine nucleotide-binding protein G(o) subunit alpha | http://www.genecards.org/cgi-bin/carddisp.pl?gene=GNAO1 | 0.700983761 | 0.122950622 |
| *GNAS* | Guanine nucleotide-binding protein G(s) subunit alpha isoforms XLas | http://www.genecards.org/cgi-bin/carddisp.pl?gene=GNAS | 0.926638042 | 0.38434911 |
| *GNLY* | Granulysin | http://www.genecards.org/cgi-bin/carddisp.pl?gene=GNLY | 0.62782465 | 0.144501277 |
| *GOLGA4* | Golgin subfamily A member 4 | http://www.genecards.org/cgi-bin/carddisp.pl?gene=GOLGA4 | 0.830689381 | 0.38883503 |
| *GPI* | Glucose-6-phosphate isomerase | http://www.genecards.org/cgi-bin/carddisp.pl?gene=GPI | 5.852267439 | 0.058921902 |
| *GPRC5A* | Retinoic acid-induced protein 3 | http://www.genecards.org/cgi-bin/carddisp.pl?gene=GPRC5A | 1.412902644 | 0.133239421 |
| *GRB2* | Growth factor receptor-bound protein 2 | http://www.genecards.org/cgi-bin/carddisp.pl?gene=GRB2 | 1.385337685 | 0.06306716 |
| *GRIN2D* | Glutamate receptor ionotropic, NMDA 2D | http://www.genecards.org/cgi-bin/carddisp.pl?gene=GRIN2D | 1.475322925 | 0.217819411 |
| *GSTA3* | Glutathione S-transferase A3 | http://www.genecards.org/cgi-bin/carddisp.pl?gene=GSTA3 | 0.84550903 | 0.232606907 |
| *GSTP1* | Glutathione S-transferase P | http://www.genecards.org/cgi-bin/carddisp.pl?gene=GSTP1 | 1.547756152 | 0.066218047 |
| *GTF2IRD1* | General transcription factor II-I repeat domain-containing protein 1 | http://www.genecards.org/cgi-bin/carddisp.pl?gene=GTF2IRD1 | 0.56832465 | 0.092851796 |
| *GTF3C1* | General transcription factor 3C polypeptide 1 | http://www.genecards.org/cgi-bin/carddisp.pl?gene=GTF3C1 | 0.764271861 | 0.173779699 |
| *GULP1* | PTB domain-containing engulfment adapter protein 1 | http://www.genecards.org/cgi-bin/carddisp.pl?gene=GULP1 | 0.59467976 | 0.133298493 |
| *H1FX* | Histone H1x | http://www.genecards.org/cgi-bin/carddisp.pl?gene=H1FX | 0.971596766 | 0.448939642 |
| *HAL* | Histidine ammonia-lyase | http://www.genecards.org/cgi-bin/carddisp.pl?gene=HAL | 0.863715114 | 0.242475305 |
| *HBA1* | Hemoglobin subunit alpha | http://www.genecards.org/cgi-bin/carddisp.pl?gene=HBA1 | 0.918146191 | 0.196330981 |
| *HBB* | Hemoglobin subunit beta | http://www.genecards.org/cgi-bin/carddisp.pl?gene=HBB | 0.972893726 | 0.463507851 |
| *HBE1* | Hemoglobin subunit epsilon | http://www.genecards.org/cgi-bin/carddisp.pl?gene=HBE1 | 0.55417348 | 0.06440382 |
| *HBEGF* | Proheparin-binding EGF-like growth factor | http://www.genecards.org/cgi-bin/carddisp.pl?gene=HBEGF | 0.60582241 | 0.249843485 |
| *HERC5* | E3 ISG15--protein ligase HERC5 | http://www.genecards.org/cgi-bin/carddisp.pl?gene=HERC5 | 0.283767283 | 0.118660135 |
| *HHLA1* | HERV-H LTR-associating protein 1 | http://www.genecards.org/cgi-bin/carddisp.pl?gene=HHLA1 | 0.799690994 | 0.271439777 |
| *HIST1H2AJ* | Histone H2A type 1-J | http://www.genecards.org/cgi-bin/carddisp.pl?gene=HIST1H2AJ | 1.192653476 | 0.367788213 |
| *HIST1H2BA* | Histone H2B type 1-A | http://www.genecards.org/cgi-bin/carddisp.pl?gene=HIST1H2BA | 0.396247082 | 0.097225884 |
| *HIST2H2BF* | Histone H2B type 2-F | http://www.genecards.org/cgi-bin/carddisp.pl?gene=HIST2H2BF | 0.872331459 | 0.272155554 |
| *HK1* | Hexokinase-1 | http://www.genecards.org/cgi-bin/carddisp.pl?gene=HK1 | 1.405126939 | 0.095641523 |
| *HLA-C* | HLA class I histocompatibility antigen, Cw-17 alpha chain | http://www.genecards.org/cgi-bin/carddisp.pl?gene=HLA-C | 2.206183341 | 0.06364961 |
| *HMGB2* | High mobility group protein B2 | http://www.genecards.org/cgi-bin/carddisp.pl?gene=HMGB2 | 0.636137593 | 0.05907032 |
| *HPCA* | Neuron-specific calcium-binding protein hippocalcin | http://www.genecards.org/cgi-bin/carddisp.pl?gene=HPCA | 0.650867349 | 0.194415846 |
| *HRNR* | Hornerin | http://www.genecards.org/cgi-bin/carddisp.pl?gene=HRNR | 0.483686834 | 0.20471451 |
| *HSP90AA2P* | Heat shock protein HSP 90-alpha A2 | http://www.genecards.org/cgi-bin/carddisp.pl?gene=HSP90AA2P | 1.553408242 | 0.072097083 |
| *HSP90AB1* | Heat shock protein HSP 90-beta | http://www.genecards.org/cgi-bin/carddisp.pl?gene=HSP90AB1 | 0.926235806 | 0.153170973 |
| *HSP90AB3P* | Putative heat shock protein HSP 90-beta-3 | http://www.genecards.org/cgi-bin/carddisp.pl?gene=HSP90AB3P | 0.413558765 | 0.112906564 |
| *HSPA6* | Heat shock 70 kDa protein 6 | http://www.genecards.org/cgi-bin/carddisp.pl?gene=HSPA6 | 0.015017068 | 0.181436199 |
| *HSPA8* | Heat shock cognate 71 kDa protein | http://www.genecards.org/cgi-bin/carddisp.pl?gene=HSPA8 | 1.082362764 | 0.295810247 |
| *HSPA9* | Stress-70 protein, mitochondrial | http://www.genecards.org/cgi-bin/carddisp.pl?gene=HSPA9 | 1.14714777 | 0.320505794 |
| *HSPB1* | Heat shock protein beta-1 | http://www.genecards.org/cgi-bin/carddisp.pl?gene=HSPB1 | 0.936440916 | 0.315643924 |
| *HSPD1* | 60 kDa heat shock protein, mitochondrial | http://www.genecards.org/cgi-bin/carddisp.pl?gene=HSPD1 | 2.4301545 | 0.054705876 |
| *HSPE1* | 10 kDa heat shock protein, mitochondrial | http://www.genecards.org/cgi-bin/carddisp.pl?gene=HSPE1 | 1.847472678 | 0.061194559 |
| *HTRA1* | Serine protease HTRA1 | http://www.genecards.org/cgi-bin/carddisp.pl?gene=HTRA1 | 1.58642439 | 0.053978613 |
| *ICAM3* | Intercellular adhesion molecule 3 | http://www.genecards.org/cgi-bin/carddisp.pl?gene=ICAM3 | 1.925505135 | 0.184922896 |
| *IGFN1* | Immunoglobulin-like and fibronectin type III domain-containing protein 1 | http://www.genecards.org/cgi-bin/carddisp.pl?gene=IGFN1 | 1.236154447 | 0.266844153 |
| *IGSF8* | Immunoglobulin superfamily member 8 | http://www.genecards.org/cgi-bin/carddisp.pl?gene=IGSF8 | 0.397246186 | 0.191617876 |
| *IMPDH2* | Inosine-5'-monophosphate dehydrogenase 2 | http://www.genecards.org/cgi-bin/carddisp.pl?gene=IMPDH2 | 0.480686272 | 0.27012108 |
| *INA* | Alpha-internexin | http://www.genecards.org/cgi-bin/carddisp.pl?gene=INA | 0.765242421 | 0.07571856 |
| *IQGAP1* | Ras GTPase-activating-like protein IQGAP1 | http://www.genecards.org/cgi-bin/carddisp.pl?gene=IQGAP1 | 0.748978766 | 0.110134607 |
| *IQGAP2* | Ras GTPase-activating-like protein IQGAP2 | http://www.genecards.org/cgi-bin/carddisp.pl?gene=IQGAP2 | 0.93201123 | 0.416470955 |
| *ITGA2B* | Integrin alpha-IIb | http://www.genecards.org/cgi-bin/carddisp.pl?gene=ITGA2B | 0.732100214 | 0.059659629 |
| *ITGB2* | Integrin beta-2 | http://www.genecards.org/cgi-bin/carddisp.pl?gene=ITGB2 | 0.897257806 | 0.286076138 |
| *ITSN1* | Intersectin-1 | http://www.genecards.org/cgi-bin/carddisp.pl?gene=ITSN1 | 1.264708117 | 0.224187531 |
| *ITSN2* | Intersectin-2 | http://www.genecards.org/cgi-bin/carddisp.pl?gene=ITSN2 | 2.420268835 | 0.055149717 |
| *JUP* | Junction plakoglobin | http://www.genecards.org/cgi-bin/carddisp.pl?gene=JUP | 0.498463342 | 0.175435127 |
| *KAT6A* | Histone acetyltransferase KAT6A | http://www.genecards.org/cgi-bin/carddisp.pl?gene=KAT6A | 0.535425674 | 0.071153565 |
| *KCNK10* | Potassium channel subfamily K member 10 | http://www.genecards.org/cgi-bin/carddisp.pl?gene=KCNK10 | 0.889127826 | 0.232113126 |
| *KIAA0556* | Protein KIAA0556 | http://www.genecards.org/cgi-bin/carddisp.pl?gene=KIAA0556 | 0.652355373 | 0.174567923 |
| *KIF17* | Kinesin-like protein KIF17 | http://www.genecards.org/cgi-bin/carddisp.pl?gene=KIF17 | 0.471660146 | 0.214116658 |
| *KIF18A* | Kinesin-like protein KIF18A | http://www.genecards.org/cgi-bin/carddisp.pl?gene=KIF18A | 4.210039404 | 0.16643595 |
| *KIF20B* | Kinesin-like protein KIF20B | http://www.genecards.org/cgi-bin/carddisp.pl?gene=KIF20B | 0.526598624 | 0.140794133 |
| *KIF26B* | Kinesin-like protein KIF26B | http://www.genecards.org/cgi-bin/carddisp.pl?gene=KIF26B | 1.202488363 | 0.369634246 |
| *KIF5A* | Kinesin heavy chain isoform 5A | http://www.genecards.org/cgi-bin/carddisp.pl?gene=KIF5A | 1.079230485 | 0.445272522 |
| *KRT1* | Keratin, type II cytoskeletal 1 | http://www.genecards.org/cgi-bin/carddisp.pl?gene=KRT1 | 0.44020671 | 0.15357688 |
| *KRT10* | Keratin, type I cytoskeletal 10 | http://www.genecards.org/cgi-bin/carddisp.pl?gene=KRT10 | 0.557018572 | 0.061081424 |
| *KRT14* | Keratin, type I cytoskeletal 14 | http://www.genecards.org/cgi-bin/carddisp.pl?gene=KRT14 | 0.254230654 | 0.112014693 |
| *KRT16* | Keratin, type I cytoskeletal 16 | http://www.genecards.org/cgi-bin/carddisp.pl?gene=KRT16 | 0.174140436 | 0.11511984 |
| *KRT17* | Keratin, type I cytoskeletal 17 | http://www.genecards.org/cgi-bin/carddisp.pl?gene=KRT17 | 0.234944079 | 0.132551493 |
| *KRT2* | Keratin, type II cytoskeletal 2 epidermal | http://www.genecards.org/cgi-bin/carddisp.pl?gene=KRT2 | 0.815287246 | 0.170977531 |
| *KRT5* | Keratin, type II cytoskeletal 5 | http://www.genecards.org/cgi-bin/carddisp.pl?gene=KRT5 | 0.343478007 | 0.119530702 |
| *KRT6A* | Keratin, type II cytoskeletal 6A | http://www.genecards.org/cgi-bin/carddisp.pl?gene=KRT6A | 0.204565088 | 0.143640075 |
| *KRT6B* | Keratin, type II cytoskeletal 6B | http://www.genecards.org/cgi-bin/carddisp.pl?gene=KRT6B | 0.256583377 | 0.256951151 |
| *KRT75* | Keratin, type II cytoskeletal 75 | http://www.genecards.org/cgi-bin/carddisp.pl?gene=KRT75 | 1.115901769 | 0.452193238 |
| *KRT78* | Keratin, type II cytoskeletal 78 | http://www.genecards.org/cgi-bin/carddisp.pl?gene=KRT78 | 0.367393427 | 0.131694782 |
| *KRT85* | Keratin, type II cuticular Hb5 | http://www.genecards.org/cgi-bin/carddisp.pl?gene=KRT85 | 1.095407549 | 0.303040855 |
| *KRT9* | Keratin, type I cytoskeletal 9 | http://www.genecards.org/cgi-bin/carddisp.pl?gene=KRT9 | 0.406321266 | 0.173802695 |
| *LAMC1* | Laminin subunit gamma-1 | http://www.genecards.org/cgi-bin/carddisp.pl?gene=LAMC1 | 0.917806268 | 0.304588618 |
| *LAMP2* | Lysosome-associated membrane glycoprotein 2 | http://www.genecards.org/cgi-bin/carddisp.pl?gene=LAMP2 | 1.219066274 | 0.10804371 |
| *LCN1* | Lipocalin-1 | http://www.genecards.org/cgi-bin/carddisp.pl?gene=LCN1 | 0.422715348 | 0.097272445 |
| *LDHA* | L-lactate dehydrogenase A chain | http://www.genecards.org/cgi-bin/carddisp.pl?gene=LDHA | 1.099727907 | 0.126211621 |
| *LDHB* | L-lactate dehydrogenase B chain | http://www.genecards.org/cgi-bin/carddisp.pl?gene=LDHB | 1.479031033 | 0.137589313 |
| *LGALS1* | Galectin-1 | http://www.genecards.org/cgi-bin/carddisp.pl?gene=LGALS1 | 0.761895638 | 0.213868622 |
| *LHFPL2* | Lipoma HMGIC fusion partner-like 2 protein | http://www.genecards.org/cgi-bin/carddisp.pl?gene=LHFPL2 | 1.398113215 | 0.094562197 |
| *LIMS1* | LIM and senescent cell antigen-like-containing domain protein 1 | http://www.genecards.org/cgi-bin/carddisp.pl?gene=LIMS1 | 1.015316834 | 0.407388156 |
| *LLGL1* | Lethal(2) giant larvae protein homolog 1 | http://www.genecards.org/cgi-bin/carddisp.pl?gene=LLGL1 | 7.323612357 | 0.070501927 |
| *LNPEP* | Leucyl-cystinyl aminopeptidase | http://www.genecards.org/cgi-bin/carddisp.pl?gene=LNPEP | 1.796858404 | 0.160970777 |
| *LPXN* | Leupaxin | http://www.genecards.org/cgi-bin/carddisp.pl?gene=LPXN | 0.781476472 | 0.123331235 |
| *LRP1* | Prolow-density lipoprotein receptor-related protein 1 | http://www.genecards.org/cgi-bin/carddisp.pl?gene=LRP1 | 2.608432163 | 0.073681258 |
| *LRPPRC* | Leucine-rich PPR motif-containing protein, mitochondrial | http://www.genecards.org/cgi-bin/carddisp.pl?gene=LRPPRC | 1.611954975 | 0.076707955 |
| *LRRC17* | Leucine-rich repeat-containing protein 17 | http://www.genecards.org/cgi-bin/carddisp.pl?gene=LRRC17 | 0.828421378 | 0.125233276 |
| *LRRIQ3* | Leucine-rich repeat and IQ domain-containing protein 3 | http://www.genecards.org/cgi-bin/carddisp.pl?gene=LRRIQ3 | 0.503092513 | 0.057813646 |
| *LRRK2* | Leucine-rich repeat serine/threonine-protein kinase 2 | http://www.genecards.org/cgi-bin/carddisp.pl?gene=LRRK2 | 0.628735018 | 0.069373017 |
| *LTF* | Lactotransferrin | http://www.genecards.org/cgi-bin/carddisp.pl?gene=LTF | 1.267424762 | 0.077740019 |
| *LYZ* | Lysozyme C | http://www.genecards.org/cgi-bin/carddisp.pl?gene=LYZ | 1.171061504 | 0.319548203 |
| *MACF1* | Microtubule-actin cross-linking factor 1, isoforms 1/2/3/5 | http://www.genecards.org/cgi-bin/carddisp.pl?gene=MACF1 | 0.763549606 | 0.265954613 |
| *MAD1L1* | Mitotic spindle assembly checkpoint protein MAD1 | http://www.genecards.org/cgi-bin/carddisp.pl?gene=MAD1L1 | 0.833926189 | 0.235380429 |
| *MAN2C1* | Alpha-mannosidase 2C1 | http://www.genecards.org/cgi-bin/carddisp.pl?gene=MAN2C1 | 0.523492717 | 0.057134124 |
| *MAP1A* | Microtubule-associated protein 1A | http://www.genecards.org/cgi-bin/carddisp.pl?gene=MAP1A | 1.851001653 | 0.05573325 |
| *MAP2* | Microtubule-associated protein 2 | http://www.genecards.org/cgi-bin/carddisp.pl?gene=MAP2 | 6.943640126 | 0.075253231 |
| *MAP6* | Microtubule-associated protein 6 | http://www.genecards.org/cgi-bin/carddisp.pl?gene=MAP6 | 1.080003995 | 0.41869124 |
| *MARCKS* | Myristoylated alanine-rich C-kinase substrate | http://www.genecards.org/cgi-bin/carddisp.pl?gene=MARCKS | 1.367243586 | 0.099373759 |
| *MARCKSL1* | MARCKS-related protein | http://www.genecards.org/cgi-bin/carddisp.pl?gene=MARCKSL1 | 1.373573591 | 0.201969064 |
| *MARK2* | Serine/threonine-protein kinase MARK2 | http://www.genecards.org/cgi-bin/carddisp.pl?gene=MARK2 | 0.836503136 | 0.171927689 |
| *MATN2* | Matrilin-2 | http://www.genecards.org/cgi-bin/carddisp.pl?gene=MATN2 | 1.046746466 | 0.331597059 |
| *MCM9* | DNA helicase MCM9 | http://www.genecards.org/cgi-bin/carddisp.pl?gene=MCM9 | 0.532678007 | 0.133136095 |
| *MDN1* | Midasin | http://www.genecards.org/cgi-bin/carddisp.pl?gene=MDN1 | 1.067943984 | 0.400330529 |
| *METTL7B* | Methyltransferase-like protein 7B | http://www.genecards.org/cgi-bin/carddisp.pl?gene=METTL7B | 0.648289806 | 0.099992813 |
| *MFGE8* | Lactadherin | http://www.genecards.org/cgi-bin/carddisp.pl?gene=MFGE8 | 1.565565805 | 0.103026516 |
| *MGAM2* | Probable maltase-glucoamylase 2 | http://www.genecards.org/cgi-bin/carddisp.pl?gene=MGAM2 | 1.094075991 | 0.334977988 |
| *MICAL2* | [F-actin]-methionine sulfoxide oxidase MICAL2 | http://www.genecards.org/cgi-bin/carddisp.pl?gene=MICAL2 | 0.763503361 | 0.05351481 |
| *MLLT6* | Protein AF-17 | http://www.genecards.org/cgi-bin/carddisp.pl?gene=MLLT6 | 0.652671968 | 0.35637276 |
| *MMP14* | Matrix metalloproteinase-14 | http://www.genecards.org/cgi-bin/carddisp.pl?gene=MMP14 | 0.9038572 | 0.133451925 |
| *MMP15* | Matrix metalloproteinase-15 | http://www.genecards.org/cgi-bin/carddisp.pl?gene=MMP15 | 0.490010891 | 0.148439812 |
| *MMRN1* | Multimerin-1 | http://www.genecards.org/cgi-bin/carddisp.pl?gene=MMRN1 | 1.180340238 | 0.23463749 |
| *MMS22L* | Protein MMS22-like | http://www.genecards.org/cgi-bin/carddisp.pl?gene=MMS22L | 0.707114676 | 0.066549075 |
| *MOB1A* | MOB kinase activator 1A | http://www.genecards.org/cgi-bin/carddisp.pl?gene=MOB1A | 1.128540477 | 0.274209515 |
| *MPP1* | 55 kDa erythrocyte membrane protein | http://www.genecards.org/cgi-bin/carddisp.pl?gene=MPP1 | 0.748865124 | 0.111827071 |
| *MPRIP* | Myosin phosphatase Rho-interacting protein | http://www.genecards.org/cgi-bin/carddisp.pl?gene=MPRIP | 0.32103936 | 0.062089326 |
| *MSN* | Moesin | http://www.genecards.org/cgi-bin/carddisp.pl?gene=MSN | 1.027840244 | 0.411672707 |
| *MTHFD2* | Bifunctional methylenetetrahydrofolate dehydrogenase/cyclohydrolase, mitochondrial | http://www.genecards.org/cgi-bin/carddisp.pl?gene=MTHFD2 | 0.939370222 | 0.409779321 |
| *MTOR* | Serine/threonine-protein kinase mTOR | http://www.genecards.org/cgi-bin/carddisp.pl?gene=MTOR | 0.973907583 | 0.35197606 |
| *MTPN* | Myotrophin | http://www.genecards.org/cgi-bin/carddisp.pl?gene=MTPN | 0.507735128 | 0.083646994 |
| *MUC16* | Mucin-16 | http://www.genecards.org/cgi-bin/carddisp.pl?gene=MUC16 | 0.933225999 | 0.437175197 |
| *MYADM* | Myeloid-associated differentiation marker | http://www.genecards.org/cgi-bin/carddisp.pl?gene=MYADM | 1.188974706 | 0.282145035 |
| *MYH11* | Myosin-11 | http://www.genecards.org/cgi-bin/carddisp.pl?gene=MYH11 | 0.595035426 | 0.097682931 |
| *MYH14* | Myosin-14 | http://www.genecards.org/cgi-bin/carddisp.pl?gene=MYH14 | 0.839417869 | 0.370871044 |
| *MYO18B* | Unconventional myosin-XVIIIb | http://www.genecards.org/cgi-bin/carddisp.pl?gene=MYO18B | 1.266853906 | 0.329660436 |
| *MYO19* | Unconventional myosin-XIX | http://www.genecards.org/cgi-bin/carddisp.pl?gene=MYO19 | 1.689923422 | 0.201004196 |
| *MYO1A* | Unconventional myosin-Ia | http://www.genecards.org/cgi-bin/carddisp.pl?gene=MYO1A | 0.614242075 | 0.116273801 |
| *MYO1B* | Unconventional myosin-Ib | http://www.genecards.org/cgi-bin/carddisp.pl?gene=MYO1B | 0.846117842 | 0.321298962 |
| *MYO5A* | Unconventional myosin-Va | http://www.genecards.org/cgi-bin/carddisp.pl?gene=MYO5A | 0.40469182 | 0.100645274 |
| *MYO5B* | Unconventional myosin-Vb | http://www.genecards.org/cgi-bin/carddisp.pl?gene=MYO5B | 1.559194177 | 0.05718103 |
| *MYO9A* | Unconventional myosin-IXa | http://www.genecards.org/cgi-bin/carddisp.pl?gene=MYO9A | 0.513226784 | 0.118796298 |
| *MYPN* | Myopalladin | http://www.genecards.org/cgi-bin/carddisp.pl?gene=MYPN | 0.581191227 | 0.072207312 |
| *MYT1L* | Myelin transcription factor 1-like protein | http://www.genecards.org/cgi-bin/carddisp.pl?gene=MYT1L | 1.343693512 | 0.111578239 |
| *NAB1* | NGFI-A-binding protein 1 | http://www.genecards.org/cgi-bin/carddisp.pl?gene=NAB1 | 0.7049712 | 0.227763121 |
| *NAPSA* | Napsin-A | http://www.genecards.org/cgi-bin/carddisp.pl?gene=NAPSA | 0.768238422 | 0.194741862 |
| *NCL* | Nucleolin | http://www.genecards.org/cgi-bin/carddisp.pl?gene=NCL | 1.012339492 | 0.456602763 |
| *NCOR2* | Nuclear receptor corepressor 2 | http://www.genecards.org/cgi-bin/carddisp.pl?gene=NCOR2 | 0.890178941 | 0.440278323 |
| *NDUFS8* | NADH dehydrogenase [ubiquinone] iron-sulfur protein 8, mitochondrial | http://www.genecards.org/cgi-bin/carddisp.pl?gene=NDUFS8 | 0.556323074 | 0.240364247 |
| *NEBL* | Nebulette | http://www.genecards.org/cgi-bin/carddisp.pl?gene=NEBL | 0.767938465 | 0.118106012 |
| *NECAP2* | Adaptin ear-binding coat-associated protein 2 | http://www.genecards.org/cgi-bin/carddisp.pl?gene=NECAP2 | 2.363113511 | 0.065760331 |
| *NEUROG3* | Neurogenin-3 | http://www.genecards.org/cgi-bin/carddisp.pl?gene=NEUROG3 | 0.969114067 | 0.449125615 |
| *NEXN* | Nexilin | http://www.genecards.org/cgi-bin/carddisp.pl?gene=NEXN | 1.03919948 | 0.27471705 |
| *NOTCH2* | Neurogenic locus notch homolog protein 2 | http://www.genecards.org/cgi-bin/carddisp.pl?gene=NOTCH2 | 0.918299049 | 0.437021077 |
| *NOV* | Protein NOV homolog | http://www.genecards.org/cgi-bin/carddisp.pl?gene=NOV | only present in RPEp53-/-SAS6-/- FA | 0.117427406 |
| *NPR2* | Atrial natriuretic peptide receptor 2 | http://www.genecards.org/cgi-bin/carddisp.pl?gene=NPR2 | 0.893815108 | 0.435716723 |
| *NRIP1* | Nuclear receptor-interacting protein 1 | http://www.genecards.org/cgi-bin/carddisp.pl?gene=NRIP1 | 11.76241858 | 0.130618784 |
| *NRK* | Nik-related protein kinase | http://www.genecards.org/cgi-bin/carddisp.pl?gene=NRK | 1.400213322 | 0.243085203 |
| *NRP1* | Neuropilin-1 | http://www.genecards.org/cgi-bin/carddisp.pl?gene=NRP1 | 1.181144863 | 0.065729425 |
| *NSDHL* | Sterol-4-alpha-carboxylate 3-dehydrogenase, decarboxylating | http://www.genecards.org/cgi-bin/carddisp.pl?gene=NSDHL | 1.60333309 | 0.050694492 |
| *NT5C1B* | Cytosolic 5'-nucleotidase 1B | http://www.genecards.org/cgi-bin/carddisp.pl?gene=NT5C1B | 2.778310294 | 0.154172983 |
| *NT5E* | 5'-nucleotidase | http://www.genecards.org/cgi-bin/carddisp.pl?gene=NT5E | 1.137886861 | 0.138417385 |
| *OCC1* | Overexpressed in colon carcinoma 1 protein | http://www.genecards.org/cgi-bin/carddisp.pl?gene=OCC1 | 0.573176471 | 0.106888945 |
| *ODF2* | Outer dense fiber protein 2 | http://www.genecards.org/cgi-bin/carddisp.pl?gene=ODF2 | 1.296004666 | 0.102527339 |
| *OR1B1* | Olfactory receptor 1B1 | http://www.genecards.org/cgi-bin/carddisp.pl?gene=OR1B1 | 1.743223078 | 0.055898428 |
| *P2RX5* | P2X purinoceptor 5 | http://www.genecards.org/cgi-bin/carddisp.pl?gene=P2RX5 | 1.248114641 | 0.329302279 |
| *P4HB* | Protein disulfide-isomerase | http://www.genecards.org/cgi-bin/carddisp.pl?gene=P4HB | 1.043051591 | 0.416569487 |
| *PA2G4* | Proliferation-associated protein 2G4 | http://www.genecards.org/cgi-bin/carddisp.pl?gene=PA2G4 | 1.200416796 | 0.068877475 |
| *PACSIN2* | Protein kinase C and casein kinase substrate in neurons protein 2 | http://www.genecards.org/cgi-bin/carddisp.pl?gene=PACSIN2 | 0.699252901 | 0.096938827 |
| *PACSIN3* | Protein kinase C and casein kinase substrate in neurons protein 3 | http://www.genecards.org/cgi-bin/carddisp.pl?gene=PACSIN3 | 2.002548292 | 0.128317487 |
| *PAK2* | Serine/threonine-protein kinase PAK 2 | http://www.genecards.org/cgi-bin/carddisp.pl?gene=PAK2 | 5.252349268 | 0.077179126 |
| *PALD1* | Paladin | http://www.genecards.org/cgi-bin/carddisp.pl?gene=PALD1 | 0.955284675 | 0.424535072 |
| *PAN2* | PAB-dependent poly(A)-specific ribonuclease subunit PAN2 | http://www.genecards.org/cgi-bin/carddisp.pl?gene=PAN2 | 2.107741363 | 0.298178102 |
| *PARK7* | Protein DJ-1 | http://www.genecards.org/cgi-bin/carddisp.pl?gene=PARK7 | 1.352719975 | 0.31770467 |
| *PARVA* | Alpha-parvin | http://www.genecards.org/cgi-bin/carddisp.pl?gene=PARVA | 1.233157941 | 0.173875428 |
| *PCBP1* | Poly(rC)-binding protein 1 | http://www.genecards.org/cgi-bin/carddisp.pl?gene=PCBP1 | 1.068470942 | 0.296339512 |
| *PCDH11X* | Protocadherin-11 X-linked | http://www.genecards.org/cgi-bin/carddisp.pl?gene=PCDH11X | 0.572387512 | 0.083550408 |
| *PCSK5* | Proprotein convertase subtilisin/kexin type 5 | http://www.genecards.org/cgi-bin/carddisp.pl?gene=PCSK5 | 0.575452756 | 0.169566247 |
| *PDAP1* | 28 kDa heat- and acid-stable phosphoprotein | http://www.genecards.org/cgi-bin/carddisp.pl?gene=PDAP1 | 0.92621512 | 0.420287596 |
| *PDCD6IP* | Programmed cell death 6-interacting protein | http://www.genecards.org/cgi-bin/carddisp.pl?gene=PDCD6IP | 0.765052444 | 0.084634416 |
| *PDE1C* | Calcium/calmodulin-dependent 3',5'-cyclic nucleotide phosphodiesterase 1C | http://www.genecards.org/cgi-bin/carddisp.pl?gene=PDE1C | 1.193926127 | 0.301800775 |
| *PDGFC* | Platelet-derived growth factor C | http://www.genecards.org/cgi-bin/carddisp.pl?gene=PDGFC | 1.544802244 | 0.132747216 |
| *PDIA3* | Protein disulfide-isomerase A3 | http://www.genecards.org/cgi-bin/carddisp.pl?gene=PDIA3 | 0.993514299 | 0.467321098 |
| *PDLIM1* | PDZ and LIM domain protein 1 | http://www.genecards.org/cgi-bin/carddisp.pl?gene=PDLIM1 | 1.682584289 | 0.101029011 |
| *PEBP1* | Phosphatidylethanolamine-binding protein 1 | http://www.genecards.org/cgi-bin/carddisp.pl?gene=PEBP1 | 0.839358601 | 0.260493052 |
| *PFDN2* | Prefoldin subunit 2 | http://www.genecards.org/cgi-bin/carddisp.pl?gene=PFDN2 | 1.105372531 | 0.318062401 |
| *PFN2* | Profilin-2 | http://www.genecards.org/cgi-bin/carddisp.pl?gene=PFN2 | 0.892465229 | 0.271540985 |
| *PGAM1* | Phosphoglycerate mutase 1 | http://www.genecards.org/cgi-bin/carddisp.pl?gene=PGAM1 | 2.112865873 | 0.050508244 |
| *PGM1* | Phosphoglucomutase-1 | http://www.genecards.org/cgi-bin/carddisp.pl?gene=PGM1 | 1.418206217 | 0.145650422 |
| *PHLDB1* | Pleckstrin homology-like domain family B member 1 | http://www.genecards.org/cgi-bin/carddisp.pl?gene=PHLDB1 | 0.911153945 | 0.194290447 |
| *PHLDB2* | Pleckstrin homology-like domain family B member 2 | http://www.genecards.org/cgi-bin/carddisp.pl?gene=PHLDB2 | 0.729022823 | 0.142322905 |
| *PICALM* | Phosphatidylinositol-binding clathrin assembly protein | http://www.genecards.org/cgi-bin/carddisp.pl?gene=PICALM | 0.9579564 | 0.433337047 |
| *PIP* | Prolactin-inducible protein | http://www.genecards.org/cgi-bin/carddisp.pl?gene=PIP | 0.383120749 | 0.115803862 |
| *PKM* | Pyruvate kinase PKM | http://www.genecards.org/cgi-bin/carddisp.pl?gene=PKM | 0.828194389 | 0.097195418 |
| *PKP2* | Plakophilin-2 | http://www.genecards.org/cgi-bin/carddisp.pl?gene=PKP2 | 1.229106846 | 0.204707914 |
| *PLAU* | Urokinase-type plasminogen activator | http://www.genecards.org/cgi-bin/carddisp.pl?gene=PLAU | 0.96199346 | 0.347430271 |
| *PLAUR* | Urokinase plasminogen activator surface receptor | http://www.genecards.org/cgi-bin/carddisp.pl?gene=PLAUR | 0.837593372 | 0.092256732 |
| *PLG* | Plasminogen | http://www.genecards.org/cgi-bin/carddisp.pl?gene=PLG | 0.507370776 | 0.132073431 |
| *PLP2* | Proteolipid protein 2 | http://www.genecards.org/cgi-bin/carddisp.pl?gene=PLP2 | 1.171016035 | 0.134075707 |
| *PLS1* | Plastin-1 | http://www.genecards.org/cgi-bin/carddisp.pl?gene=PLS1 | 0.865797322 | 0.269317935 |
| *PLSCR1* | Phospholipid scramblase 1 | http://www.genecards.org/cgi-bin/carddisp.pl?gene=PLSCR1 | 1.097456897 | 0.268644759 |
| *PODXL* | Podocalyxin | http://www.genecards.org/cgi-bin/carddisp.pl?gene=PODXL | 0.724516465 | 0.068797555 |
| *POLR2H* | DNA-directed RNA polymerases I, II, and III subunit RPABC3 | http://www.genecards.org/cgi-bin/carddisp.pl?gene=POLR2H | 4.932587046 | 0.196384077 |
| *POLR3A* | DNA-directed RNA polymerase III subunit RPC1 | http://www.genecards.org/cgi-bin/carddisp.pl?gene=POLR3A | 6.112705052 | 0.185337794 |
| *POTEF* | POTE ankyrin domain family member F | http://www.genecards.org/cgi-bin/carddisp.pl?gene=POTEF | 0.71355522 | 0.10539252 |
| *PPFIBP1* | Liprin-beta-1 | http://www.genecards.org/cgi-bin/carddisp.pl?gene=PPFIBP1 | 0.904138724 | 0.235347861 |
| *PPIA* | Peptidyl-prolyl cis-trans isomerase A | http://www.genecards.org/cgi-bin/carddisp.pl?gene=PPIA | 0.861827103 | 0.090441734 |
| *PPIAL4A* | Peptidyl-prolyl cis-trans isomerase A-like 4A | http://www.genecards.org/cgi-bin/carddisp.pl?gene=PPIAL4A | 1.22120419 | 0.290736568 |
| *PPIP5K2* | Inositol hexakisphosphate and diphosphoinositol-pentakisphosphate kinase 2 | http://www.genecards.org/cgi-bin/carddisp.pl?gene=PPIP5K2 | 0.796703573 | 0.224873072 |
| *PPP1R16A* | Protein phosphatase 1 regulatory subunit 16A | http://www.genecards.org/cgi-bin/carddisp.pl?gene=PPP1R16A | 1.306138528 | 0.349095334 |
| *PPP2R1A* | Serine/threonine-protein phosphatase 2A 65 kDa regulatory subunit A alpha isoform | http://www.genecards.org/cgi-bin/carddisp.pl?gene=PPP2R1A | 1.419977321 | 0.14290206 |
| *PRAMEF7* | PRAME family member 7 | http://www.genecards.org/cgi-bin/carddisp.pl?gene=PRAMEF7 | 4.513489131 | 0.134881489 |
| *PRDM1* | PR domain zinc finger protein 1 | http://www.genecards.org/cgi-bin/carddisp.pl?gene=PRDM1 | 0.615647033 | 0.089742142 |
| *PRG4* | Proteoglycan 4 | http://www.genecards.org/cgi-bin/carddisp.pl?gene=PRG4 | 1.148513307 | 0.377546413 |
| *PRKCSH* | Glucosidase 2 subunit beta | http://www.genecards.org/cgi-bin/carddisp.pl?gene=PRKCSH | 1.264075841 | 0.077304603 |
| *PRSS3* | Trypsin-3 | http://www.genecards.org/cgi-bin/carddisp.pl?gene=PRSS3 | 1.085440733 | 0.462022938 |
| *PSEN1* | Presenilin-1 | http://www.genecards.org/cgi-bin/carddisp.pl?gene=PSEN1 | 2.440729624 | 0.061994695 |
| *PSMD7* | 26S proteasome non-ATPase regulatory subunit 7 | http://www.genecards.org/cgi-bin/carddisp.pl?gene=PSMD7 | 1.054664542 | 0.440071925 |
| *PTPN13* | Tyrosine-protein phosphatase non-receptor type 13 | http://www.genecards.org/cgi-bin/carddisp.pl?gene=PTPN13 | 2.277004666 | 0.165637666 |
| *PTPRA* | Receptor-type tyrosine-protein phosphatase alpha | http://www.genecards.org/cgi-bin/carddisp.pl?gene=PTPRA | 0.12212447 | 0.152012315 |
| *PTPRJ* | Receptor-type tyrosine-protein phosphatase eta | http://www.genecards.org/cgi-bin/carddisp.pl?gene=PTPRJ | 0.78043805 | 0.299343545 |
| *PTTG1IP* | Pituitary tumor-transforming gene 1 protein-interacting protein | http://www.genecards.org/cgi-bin/carddisp.pl?gene=PTTG1IP | 3.340981737 | 0.123552252 |
| *PTX3* | Pentraxin-related protein PTX3 | http://www.genecards.org/cgi-bin/carddisp.pl?gene=PTX3 | 1.14648559 | 0.357097362 |
| *PVR* | Poliovirus receptor | http://www.genecards.org/cgi-bin/carddisp.pl?gene=PVR | 1.070187299 | 0.28638072 |
| *QSOX2* | Sulfhydryl oxidase 2 | http://www.genecards.org/cgi-bin/carddisp.pl?gene=QSOX2 | 0.920883575 | 0.284631554 |
| *QTRT2* | Queuine tRNA-ribosyltransferase accessory subunit 2 | http://www.genecards.org/cgi-bin/carddisp.pl?gene=QTRT2 | 1.336393373 | 0.304249501 |
| *RAB13* | Ras-related protein Rab-13 | http://www.genecards.org/cgi-bin/carddisp.pl?gene=RAB13 | 1.269842235 | 0.294737887 |
| *RAB1A* | Ras-related protein Rab-1A | http://www.genecards.org/cgi-bin/carddisp.pl?gene=RAB1A | 1.191858749 | 0.33118209 |
| *RAB21* | Ras-related protein Rab-21 | http://www.genecards.org/cgi-bin/carddisp.pl?gene=RAB21 | 1.114560151 | 0.13489742 |
| *RAB23* | Ras-related protein Rab-23 | http://www.genecards.org/cgi-bin/carddisp.pl?gene=RAB23 | 0.787630621 | 0.084463101 |
| *RAB2B* | Ras-related protein Rab-2B | http://www.genecards.org/cgi-bin/carddisp.pl?gene=RAB2B | 1.467744098 | 0.055289783 |
| *RAB40C* | Ras-related protein Rab-40C | http://www.genecards.org/cgi-bin/carddisp.pl?gene=RAB40C | 0.909714314 | 0.192570802 |
| *RAB5C* | Ras-related protein Rab-5C | http://www.genecards.org/cgi-bin/carddisp.pl?gene=RAB5C | 2.49568792 | 0.087232505 |
| *RABGAP1* | Rab GTPase-activating protein 1 | http://www.genecards.org/cgi-bin/carddisp.pl?gene=RABGAP1 | 0.922408963 | 0.318122129 |
| *RACK1* | Receptor of activated protein C kinase 1 | http://www.genecards.org/cgi-bin/carddisp.pl?gene=RACK1 | 1.092557657 | 0.219654549 |
| *RALA* | Ras-related protein Ral-A | http://www.genecards.org/cgi-bin/carddisp.pl?gene=RALA | 4.510022093 | 0.094592509 |
| *RALB* | Ras-related protein Ral-B | http://www.genecards.org/cgi-bin/carddisp.pl?gene=RALB | 3.189181782 | 0.105665364 |
| *RAP2B* | Ras-related protein Rap-2b | http://www.genecards.org/cgi-bin/carddisp.pl?gene=RAP2B | 1.00416275 | 0.486823908 |
| *RAPH1* | Ras-associated and pleckstrin homology domains-containing protein 1 | http://www.genecards.org/cgi-bin/carddisp.pl?gene=RAPH1 | 1.013411636 | 0.466752376 |
| *RASA2* | Ras GTPase-activating protein 2 | http://www.genecards.org/cgi-bin/carddisp.pl?gene=RASA2 | 0.67189112 | 0.063588249 |
| *RCN1* | Reticulocalbin-1 | http://www.genecards.org/cgi-bin/carddisp.pl?gene=RCN1 | 1.115132743 | 0.371872202 |
| *RFNG* | Beta-1,3-N-acetylglucosaminyltransferase radical fringe | http://www.genecards.org/cgi-bin/carddisp.pl?gene=RFNG | 1.12076521 | 0.36251103 |
| *RHOA* | Transforming protein RhoA | http://www.genecards.org/cgi-bin/carddisp.pl?gene=RHOA | 1.289939243 | 0.109147211 |
| *RHOG* | Rho-related GTP-binding protein RhoG | http://www.genecards.org/cgi-bin/carddisp.pl?gene=RHOG | 1.278176682 | 0.155555576 |
| *RNPEPL1* | Aminopeptidase RNPEPL1 | http://www.genecards.org/cgi-bin/carddisp.pl?gene=RNPEPL1 | 2.147922089 | 0.150143093 |
| *RORB* | Nuclear receptor ROR-beta | http://www.genecards.org/cgi-bin/carddisp.pl?gene=RORB | 0.38190356 | 0.108735358 |
| *RP2* | Protein XRP2 | http://www.genecards.org/cgi-bin/carddisp.pl?gene=RP2 | 0.998617306 | 0.491511924 |
| *RPL24* | 60S ribosomal protein L24 | http://www.genecards.org/cgi-bin/carddisp.pl?gene=RPL24 | 0.893360596 | 0.279133998 |
| *RPL27* | 60S ribosomal protein L27 | http://www.genecards.org/cgi-bin/carddisp.pl?gene=RPL27 | 0.994313407 | 0.47298949 |
| *RPL28* | 60S ribosomal protein L28 | http://www.genecards.org/cgi-bin/carddisp.pl?gene=RPL28 | 1.347858273 | 0.318403608 |
| *RPL3* | 60S ribosomal protein L3 | http://www.genecards.org/cgi-bin/carddisp.pl?gene=RPL3 | 1.529808169 | 0.051970199 |
| *RPL9* | 60S ribosomal protein L9 | http://www.genecards.org/cgi-bin/carddisp.pl?gene=RPL9 | 4.543855664 | 0.116096437 |
| *RPLP1* | 60S acidic ribosomal protein P1 | http://www.genecards.org/cgi-bin/carddisp.pl?gene=RPLP1 | 2.394186938 | 0.106248876 |
| *RPLP2* | 60S acidic ribosomal protein P2 | http://www.genecards.org/cgi-bin/carddisp.pl?gene=RPLP2 | 1.28263832 | 0.057680205 |
| *RPS17* | 40S ribosomal protein S17 | http://www.genecards.org/cgi-bin/carddisp.pl?gene=RPS17 | 3.263742989 | 0.148476885 |
| *RPS2* | 40S ribosomal protein S2 | http://www.genecards.org/cgi-bin/carddisp.pl?gene=RPS2 | 0.926045725 | 0.222391667 |
| *RPS21* | 40S ribosomal protein S21 | http://www.genecards.org/cgi-bin/carddisp.pl?gene=RPS21 | 1.090705664 | 0.417903355 |
| *RPS25* | 40S ribosomal protein S25 | http://www.genecards.org/cgi-bin/carddisp.pl?gene=RPS25 | 0.591533492 | 0.103204345 |
| *RPS27A* | Ubiquitin-40S ribosomal protein S27a | http://www.genecards.org/cgi-bin/carddisp.pl?gene=RPS27A | 0.889830333 | 0.170948028 |
| *RPS28* | 40S ribosomal protein S28 | http://www.genecards.org/cgi-bin/carddisp.pl?gene=RPS28 | 1.318523043 | 0.187195925 |
| *RPS3* | 40S ribosomal protein S3 | http://www.genecards.org/cgi-bin/carddisp.pl?gene=RPS3 | 1.140078118 | 0.209043691 |
| *RPS3A* | 40S ribosomal protein S3a | http://www.genecards.org/cgi-bin/carddisp.pl?gene=RPS3A | 2.493330889 | 0.099050448 |
| *RPS4X* | 40S ribosomal protein S4, X isoform | http://www.genecards.org/cgi-bin/carddisp.pl?gene=RPS4X | 0.562132647 | 0.170885562 |
| *RRAS* | Ras-related protein R-Ras | http://www.genecards.org/cgi-bin/carddisp.pl?gene=RRAS | 4.039646597 | 0.078238552 |
| *S100A13* | Protein S100-A13 | http://www.genecards.org/cgi-bin/carddisp.pl?gene=S100A13 | 0.948302983 | 0.363910008 |
| *S100A9* | Protein S100-A9 | http://www.genecards.org/cgi-bin/carddisp.pl?gene=S100A9 | 0.373939421 | 0.091164819 |
| *SACS* | Sacsin | http://www.genecards.org/cgi-bin/carddisp.pl?gene=SACS | 1.06687486 | 0.270577131 |
| *SBNO2* | Protein strawberry notch homolog 2 | http://www.genecards.org/cgi-bin/carddisp.pl?gene=SBNO2 | 1.369649633 | 0.165147016 |
| *SCAMP1* | Secretory carrier-associated membrane protein 1 | http://www.genecards.org/cgi-bin/carddisp.pl?gene=SCAMP1 | 2.098646283 | 0.070458093 |
| *SCAMP4* | Secretory carrier-associated membrane protein 4 | http://www.genecards.org/cgi-bin/carddisp.pl?gene=SCAMP4 | 1.381397037 | 0.05703192 |
| *SCN11A* | Sodium channel protein type 11 subunit alpha | http://www.genecards.org/cgi-bin/carddisp.pl?gene=SCN11A | 0.694305847 | 0.078685011 |
| *SCRIB* | Protein scribble homolog | http://www.genecards.org/cgi-bin/carddisp.pl?gene=SCRIB | 0.774363104 | 0.245845957 |
| *SDC2* | Syndecan-2 | http://www.genecards.org/cgi-bin/carddisp.pl?gene=SDC2 | 0.911867023 | 0.293330809 |
| *SEMA3C* | Semaphorin-3C | http://www.genecards.org/cgi-bin/carddisp.pl?gene=SEMA3C | 0.750323748 | 0.058402959 |
| *SEP10* | Septin-10 | http://www.genecards.org/cgi-bin/carddisp.pl?gene=SEP10 | 1.873000172 | 0.130018422 |
| *SEPT1* | Septin-1 | http://www.genecards.org/cgi-bin/carddisp.pl?gene=SEPT1 | 1.278936386 | 0.211410847 |
| *SEPT6* | Septin-6 | http://www.genecards.org/cgi-bin/carddisp.pl?gene=SEPT6 | 1.223665709 | 0.110526247 |
| *SEPT7* | Septin-7 | http://www.genecards.org/cgi-bin/carddisp.pl?gene=SEPT7 | 1.234021564 | 0.171590518 |
| *SEPT9* | Septin-9 | http://www.genecards.org/cgi-bin/carddisp.pl?gene=SEPT9 | 1.137307479 | 0.326982082 |
| *SERBP1* | Plasminogen activator inhibitor 1 RNA-binding protein | http://www.genecards.org/cgi-bin/carddisp.pl?gene=SERBP1 | 1.012543289 | 0.468954885 |
| *SERPINA1* | Alpha-1-antitrypsin | http://www.genecards.org/cgi-bin/carddisp.pl?gene=SERPINA1 | 1.501550129 | 0.050161304 |
| *SERPINF1* | Pigment epithelium-derived factor | http://www.genecards.org/cgi-bin/carddisp.pl?gene=SERPINF1 | 0.805117316 | 0.185202361 |
| *SERPINF2* | Alpha-2-antiplasmin | http://www.genecards.org/cgi-bin/carddisp.pl?gene=SERPINF2 | 0.977315318 | 0.456500527 |
| *SERPINH1* | Serpin H1 | http://www.genecards.org/cgi-bin/carddisp.pl?gene=SERPINH1 | 1.07576041 | 0.35443408 |
| *SETD3* | Histone-lysine N-methyltransferase setd3 | http://www.genecards.org/cgi-bin/carddisp.pl?gene=SETD3 | 0.627460987 | 0.355067556 |
| *SH3GL2* | Endophilin-A1 | http://www.genecards.org/cgi-bin/carddisp.pl?gene=SH3GL2 | 1.283746749 | 0.35970333 |
| *SIRPA* | Tyrosine-protein phosphatase non-receptor type substrate 1 | http://www.genecards.org/cgi-bin/carddisp.pl?gene=SIRPA | 0.592649584 | 0.12738742 |
| *SLC12A1* | Solute carrier family 12 member 1 | http://www.genecards.org/cgi-bin/carddisp.pl?gene=SLC12A1 | 2.220274626 | 0.162814669 |
| *SLC25A3* | Phosphate carrier protein, mitochondrial | http://www.genecards.org/cgi-bin/carddisp.pl?gene=SLC25A3 | 0.836693484 | 0.145839869 |
| *SLC25A5* | ADP/ATP translocase 2 | http://www.genecards.org/cgi-bin/carddisp.pl?gene=SLC25A5 | 1.426824113 | 0.050349971 |
| *SLC29A1* | Equilibrative nucleoside transporter 1 | http://www.genecards.org/cgi-bin/carddisp.pl?gene=SLC29A1 | 0.648891904 | 0.172710397 |
| *SLC39A10* | Zinc transporter ZIP10 | http://www.genecards.org/cgi-bin/carddisp.pl?gene=SLC39A10 | 0.851602355 | 0.283192031 |
| *SLC39A12* | Zinc transporter ZIP12 | http://www.genecards.org/cgi-bin/carddisp.pl?gene=SLC39A12 | 0.86027664 | 0.212684363 |
| *SLC9A2* | Sodium/hydrogen exchanger 2 | http://www.genecards.org/cgi-bin/carddisp.pl?gene=SLC9A2 | 0.996281201 | 0.491004053 |
| *SLC9A3R1* | Na(+)/H(+) exchange regulatory cofactor NHE-RF1 | http://www.genecards.org/cgi-bin/carddisp.pl?gene=SLC9A3R1 | 1.111915985 | 0.153592908 |
| *SLIT1* | Slit homolog 1 protein | http://www.genecards.org/cgi-bin/carddisp.pl?gene=SLIT1 | 0.453356588 | 0.205279392 |
| *SMARCA2* | Probable global transcription activator SNF2L2 | http://www.genecards.org/cgi-bin/carddisp.pl?gene=SMARCA2 | 0.725823931 | 0.158704289 |
| *SMARCA5* | SWI/SNF-related matrix-associated actin-dependent regulator of chromatin subfamily A member 5 | http://www.genecards.org/cgi-bin/carddisp.pl?gene=SMARCA5 | 1.789001168 | 0.116722296 |
| *SNRPD2* | Small nuclear ribonucleoprotein Sm D2 | http://www.genecards.org/cgi-bin/carddisp.pl?gene=SNRPD2 | 2.419688017 | 0.160329692 |
| *SNRPE* | Small nuclear ribonucleoprotein E | http://www.genecards.org/cgi-bin/carddisp.pl?gene=SNRPE | 0.99216124 | 0.494017313 |
| *SNRPF* | Small nuclear ribonucleoprotein F | http://www.genecards.org/cgi-bin/carddisp.pl?gene=SNRPF | 1.083042692 | 0.286042919 |
| *SNTB2* | Beta-2-syntrophin | http://www.genecards.org/cgi-bin/carddisp.pl?gene=SNTB2 | 1.205451501 | 0.076925177 |
| *SORBS2* | Sorbin and SH3 domain-containing protein 2 | http://www.genecards.org/cgi-bin/carddisp.pl?gene=SORBS2 | 0.563668925 | 0.146316045 |
| *SORBS3* | Vinexin | http://www.genecards.org/cgi-bin/carddisp.pl?gene=SORBS3 | 1.294079832 | 0.088804147 |
| *SPECC1* | Cytospin-B | http://www.genecards.org/cgi-bin/carddisp.pl?gene=SPECC1 | 9.246626868 | 0.171552147 |
| *SPTAN1* | Spectrin alpha chain, non-erythrocytic 1 | http://www.genecards.org/cgi-bin/carddisp.pl?gene=SPTAN1 | 2.269657673 | 0.076630896 |
| *SPTB* | Spectrin beta chain, erythrocytic | http://www.genecards.org/cgi-bin/carddisp.pl?gene=SPTB | 2.109406598 | 0.218432049 |
| *SRP9* | Signal recognition particle 9 kDa protein | http://www.genecards.org/cgi-bin/carddisp.pl?gene=SRP9 | 1.070261723 | 0.364588703 |
| *SRSF3* | Serine/arginine-rich splicing factor 3 | http://www.genecards.org/cgi-bin/carddisp.pl?gene=SRSF3 | 0.574356634 | 0.062444463 |
| *SSC4D* | Scavenger receptor cysteine-rich domain-containing group B protein | http://www.genecards.org/cgi-bin/carddisp.pl?gene=SSC4D | 0.509444094 | 0.065748783 |
| *ST13P5* | Putative protein FAM10A5 | http://www.genecards.org/cgi-bin/carddisp.pl?gene=ST13P5 | 1.025098829 | 0.466767644 |
| *ST7* | Suppressor of tumorigenicity 7 protein | http://www.genecards.org/cgi-bin/carddisp.pl?gene=ST7 | 0.487470122 | 0.186564064 |
| *STRAP* | Serine-threonine kinase receptor-associated protein | http://www.genecards.org/cgi-bin/carddisp.pl?gene=STRAP | 0.786246597 | 0.362444084 |
| *STX12* | Syntaxin-12 | http://www.genecards.org/cgi-bin/carddisp.pl?gene=STX12 | 0.398556645 | 0.095345132 |
| *STXBP1* | Syntaxin-binding protein 1 | http://www.genecards.org/cgi-bin/carddisp.pl?gene=STXBP1 | 1.194236689 | 0.176007812 |
| *SVIP* | Small VCP/p97-interacting protein | http://www.genecards.org/cgi-bin/carddisp.pl?gene=SVIP | 0.811842642 | 0.074795175 |
| *SYCP1* | Synaptonemal complex protein 1 | http://www.genecards.org/cgi-bin/carddisp.pl?gene=SYCP1 | 1.000137149 | 0.49974863 |
| *SYK* | Tyrosine-protein kinase SYK | http://www.genecards.org/cgi-bin/carddisp.pl?gene=SYK | 0.69399535 | 0.121749588 |
| *SYNCRIP* | Heterogeneous nuclear ribonucleoprotein Q | http://www.genecards.org/cgi-bin/carddisp.pl?gene=SYNCRIP | 21.69017224 | 0.140908037 |
| *SYNE1* | Nesprin-1 | http://www.genecards.org/cgi-bin/carddisp.pl?gene=SYNE1 | 0.585372624 | 0.135731001 |
| *SYNE2* | Nesprin-2 | http://www.genecards.org/cgi-bin/carddisp.pl?gene=SYNE2 | 1.008497351 | 0.484065481 |
| *SYNJ2* | Synaptojanin-2 | http://www.genecards.org/cgi-bin/carddisp.pl?gene=SYNJ2 | 2.123442425 | 0.061744193 |
| *SYNM* | Synemin | http://www.genecards.org/cgi-bin/carddisp.pl?gene=SYNM | 0.644436441 | 0.221247 |
| *SYTL2* | Synaptotagmin-like protein 2 | http://www.genecards.org/cgi-bin/carddisp.pl?gene=SYTL2 | 0.63782708 | 0.110552659 |
| *TAF4B* | Transcription initiation factor TFIID subunit 4B | http://www.genecards.org/cgi-bin/carddisp.pl?gene=TAF4B | 1.437588763 | 0.057815957 |
| *TAF7* | Transcription initiation factor TFIID subunit 7 | http://www.genecards.org/cgi-bin/carddisp.pl?gene=TAF7 | 0.705228521 | 0.174019569 |
| *TAGLN2* | Transgelin-2 | http://www.genecards.org/cgi-bin/carddisp.pl?gene=TAGLN2 | 1.534118305 | 0.069794928 |
| *TAOK2* | Serine/threonine-protein kinase TAO2 | http://www.genecards.org/cgi-bin/carddisp.pl?gene=TAOK2 | 1.000895515 | 0.497950397 |
| *TBC1D24* | TBC1 domain family member 24 | http://www.genecards.org/cgi-bin/carddisp.pl?gene=TBC1D24 | 1.349941494 | 0.17675888 |
| *TBC1D8B* | TBC1 domain family member 8B | http://www.genecards.org/cgi-bin/carddisp.pl?gene=TBC1D8B | 1.807860224 | 0.076609163 |
| *TBL3* | Transducin beta-like protein 3 | http://www.genecards.org/cgi-bin/carddisp.pl?gene=TBL3 | 0.427081125 | 0.071820948 |
| *TEAD1* | Transcriptional enhancer factor TEF-1 | http://www.genecards.org/cgi-bin/carddisp.pl?gene=TEAD1 | only present in RPEp53-/- FA | 0.195501109 |
| *TF* | Serotransferrin | http://www.genecards.org/cgi-bin/carddisp.pl?gene=TF | 1.40292809 | 0.075857643 |
| *TGFBI* | Transforming growth factor-beta-induced protein ig-h3 | http://www.genecards.org/cgi-bin/carddisp.pl?gene=TGFBI | 1.05678114 | 0.188523075 |
| *TGOLN2* | Trans-Golgi network integral membrane protein 2 | http://www.genecards.org/cgi-bin/carddisp.pl?gene=TGOLN2 | 1.152018749 | 0.125371702 |
| *THADA* | Thyroid adenoma-associated protein | http://www.genecards.org/cgi-bin/carddisp.pl?gene=THADA | 0.787690784 | 0.419632833 |
| *THBS2* | Thrombospondin-2 | http://www.genecards.org/cgi-bin/carddisp.pl?gene=THBS2 | 0.681643988 | 0.215256847 |
| *THBS4* | Thrombospondin-4 | http://www.genecards.org/cgi-bin/carddisp.pl?gene=THBS4 | 1.446845001 | 0.201335447 |
| *THRAP3* | Thyroid hormone receptor-associated protein 3 | http://www.genecards.org/cgi-bin/carddisp.pl?gene=THRAP3 | 1.074846406 | 0.221772393 |
| *THSD4* | Thrombospondin type-1 domain-containing protein 4 | http://www.genecards.org/cgi-bin/carddisp.pl?gene=THSD4 | 0.962461774 | 0.302083844 |
| *TIPARP* | TCDD-inducible poly [ADP-ribose] polymerase | http://www.genecards.org/cgi-bin/carddisp.pl?gene=TIPARP | 0.803836274 | 0.275945216 |
| *TKFC* | Triokinase/FMN cyclase | http://www.genecards.org/cgi-bin/carddisp.pl?gene=TKFC | 0.461169699 | 0.102725189 |
| *TKT* | Transketolase | http://www.genecards.org/cgi-bin/carddisp.pl?gene=TKT | 0.942640783 | 0.375885876 |
| *TLDC1* | TLD domain-containing protein 1 | http://www.genecards.org/cgi-bin/carddisp.pl?gene=TLDC1 | 46.1609087 | 0.193823933 |
| *TLL2* | Tolloid-like protein 2 | http://www.genecards.org/cgi-bin/carddisp.pl?gene=TLL2 | 1.906091151 | 0.082480503 |
| *TLN2* | Talin-2 | http://www.genecards.org/cgi-bin/carddisp.pl?gene=TLN2 | 1.263740457 | 0.191193659 |
| *TLR5* | Toll-like receptor 5 | http://www.genecards.org/cgi-bin/carddisp.pl?gene=TLR5 | 1.071589778 | 0.414002527 |
| *TMBIM1* | Protein lifeguard 3 | http://www.genecards.org/cgi-bin/carddisp.pl?gene=TMBIM1 | 0.557579769 | 0.051063677 |
| *TMC3* | Transmembrane channel-like protein 3 | http://www.genecards.org/cgi-bin/carddisp.pl?gene=TMC3 | 1.170449857 | 0.443668915 |
| *TMEFF1* | Tomoregulin-1 | http://www.genecards.org/cgi-bin/carddisp.pl?gene=TMEFF1 | 0.55595172 | 0.256056792 |
| *TMEM109* | Transmembrane protein 109 | http://www.genecards.org/cgi-bin/carddisp.pl?gene=TMEM109 | 1.112072095 | 0.356066636 |
| *TMEM123* | Porimin | http://www.genecards.org/cgi-bin/carddisp.pl?gene=TMEM123 | 1.142804886 | 0.320640728 |
| *TMEM2* | Transmembrane protein 2 | http://www.genecards.org/cgi-bin/carddisp.pl?gene=TMEM2 | 0.750359278 | 0.084169722 |
| *TNFRSF12A* | Tumor necrosis factor receptor superfamily member 12A | http://www.genecards.org/cgi-bin/carddisp.pl?gene=TNFRSF12A | 1.636830111 | 0.137592681 |
| *TNS4* | Tensin-4 | http://www.genecards.org/cgi-bin/carddisp.pl?gene=TNS4 | 1.01579294 | 0.484017124 |
| *TPD52L2* | Tumor protein D54 | http://www.genecards.org/cgi-bin/carddisp.pl?gene=TPD52L2 | 1.447983467 | 0.277930456 |
| *TPI1* | Triosephosphate isomerase | http://www.genecards.org/cgi-bin/carddisp.pl?gene=TPI1 | 1.00661178 | 0.480853799 |
| *TPT1* | Translationally-controlled tumor protein | http://www.genecards.org/cgi-bin/carddisp.pl?gene=TPT1 | 1.147065029 | 0.295880999 |
| *TRAK1* | Trafficking kinesin-binding protein 1 | http://www.genecards.org/cgi-bin/carddisp.pl?gene=TRAK1 | 3.223840836 | 0.198795395 |
| *TRDN* | Triadin | http://www.genecards.org/cgi-bin/carddisp.pl?gene=TRDN | 1.672699057 | 0.200953846 |
| *TRIM4* | E3 ubiquitin-protein ligase TRIM4 | http://www.genecards.org/cgi-bin/carddisp.pl?gene=TRIM4 | 0.240779179 | 0.059505418 |
| *TRIM58* | E3 ubiquitin-protein ligase TRIM58 | http://www.genecards.org/cgi-bin/carddisp.pl?gene=TRIM58 | 21.14931474 | 0.108095169 |
| *TRIOBP* | TRIO and F-actin-binding protein | http://www.genecards.org/cgi-bin/carddisp.pl?gene=TRIOBP | 1.09797521 | 0.288900337 |
| *TRMT10B* | tRNA methyltransferase 10 homolog B | http://www.genecards.org/cgi-bin/carddisp.pl?gene=TRMT10B | 0.952773438 | 0.471134722 |
| *TRPC7* | Short transient receptor potential channel 7 | http://www.genecards.org/cgi-bin/carddisp.pl?gene=TRPC7 | 2.289887042 | 0.107471243 |
| *TRPM2* | Transient receptor potential cation channel subfamily M member 2 | http://www.genecards.org/cgi-bin/carddisp.pl?gene=TRPM2 | 1.936245504 | 0.111487502 |
| *TSNAXIP1* | Translin-associated factor X-interacting protein 1 | http://www.genecards.org/cgi-bin/carddisp.pl?gene=TSNAXIP1 | 0.419535679 | 0.11718517 |
| *TSPOAP1* | Peripheral-type benzodiazepine receptor-associated protein 1 | http://www.genecards.org/cgi-bin/carddisp.pl?gene=TSPOAP1 | 0.370662796 | 0.235561129 |
| *TTBK1* | Tau-tubulin kinase 1 | http://www.genecards.org/cgi-bin/carddisp.pl?gene=TTBK1 | 0.410344526 | 0.092756122 |
| *TTC17* | Tetratricopeptide repeat protein 17 | http://www.genecards.org/cgi-bin/carddisp.pl?gene=TTC17 | 0.781920455 | 0.332185107 |
| *TTC3* | E3 ubiquitin-protein ligase TTC3 | http://www.genecards.org/cgi-bin/carddisp.pl?gene=TTC3 | 1.233522867 | 0.256864659 |
| *TUBA1A* | Tubulin alpha-1A chain | http://www.genecards.org/cgi-bin/carddisp.pl?gene=TUBA1A | 1.117661032 | 0.425169229 |
| *TUBA4A* | Tubulin alpha-4A chain | http://www.genecards.org/cgi-bin/carddisp.pl?gene=TUBA4A | 0.940446796 | 0.434311346 |
| *TUBB* | Tubulin beta chain | http://www.genecards.org/cgi-bin/carddisp.pl?gene=TUBB | 0.864462892 | 0.24125643 |
| *TUBB4B* | Tubulin beta-4B chain | http://www.genecards.org/cgi-bin/carddisp.pl?gene=TUBB4B | 0.881345474 | 0.205779612 |
| *TXN* | Thioredoxin | http://www.genecards.org/cgi-bin/carddisp.pl?gene=TXN | 0.862614275 | 0.185046513 |
| *TXNDC5* | Thioredoxin domain-containing protein 5 | http://www.genecards.org/cgi-bin/carddisp.pl?gene=TXNDC5 | 2.000088809 | 0.120355048 |
| *TYMSOS* | TYMS opposite strand protein | http://www.genecards.org/cgi-bin/carddisp.pl?gene=TYMSOS | 0.534364749 | 0.053425846 |
| *UACA* | Uveal autoantigen with coiled-coil domains and ankyrin repeats | http://www.genecards.org/cgi-bin/carddisp.pl?gene=UACA | 0.967305461 | 0.400746186 |
| *UBA1* | Ubiquitin-like modifier-activating enzyme 1 | http://www.genecards.org/cgi-bin/carddisp.pl?gene=UBA1 | 0.70367639 | 0.18024458 |
| *UBAP2* | Ubiquitin-associated protein 2 | http://www.genecards.org/cgi-bin/carddisp.pl?gene=UBAP2 | 0.922702263 | 0.395236824 |
| *UHRF1* | E3 ubiquitin-protein ligase UHRF1 | http://www.genecards.org/cgi-bin/carddisp.pl?gene=UHRF1 | 0.47080621 | 0.089312563 |
| *UNC80* | Protein unc-80 homolog | http://www.genecards.org/cgi-bin/carddisp.pl?gene=UNC80 | 0.961497813 | 0.360715873 |
| *VAT1* | Synaptic vesicle membrane protein VAT-1 homolog | http://www.genecards.org/cgi-bin/carddisp.pl?gene=VAT1 | 2.580674234 | 0.25407346 |
| *VAV1* | Proto-oncogene vav | http://www.genecards.org/cgi-bin/carddisp.pl?gene=VAV1 | 0.651135139 | 0.158868646 |
| *VTA1* | Vacuolar protein sorting-associated protein VTA1 homolog | http://www.genecards.org/cgi-bin/carddisp.pl?gene=VTA1 | 0.544558442 | 0.209366632 |
| *WASF2* | Wiskott-Aldrich syndrome protein family member 2 | http://www.genecards.org/cgi-bin/carddisp.pl?gene=WASF2 | 1.352728673 | 0.11383666 |
| *WDR1* | WD repeat-containing protein 1 | http://www.genecards.org/cgi-bin/carddisp.pl?gene=WDR1 | 1.35797847 | 0.084161015 |
| *WNT5A* | Protein Wnt-5a | http://www.genecards.org/cgi-bin/carddisp.pl?gene=WNT5A | 1.227750062 | 0.071788041 |
| *WRN* | Werner syndrome ATP-dependent helicase | http://www.genecards.org/cgi-bin/carddisp.pl?gene=WRN | 0.941719868 | 0.288334972 |
| *XAGE5* | X antigen family member 5 | http://www.genecards.org/cgi-bin/carddisp.pl?gene=XAGE5 | 0.514524152 | 0.110542503 |
| *YES1* | Tyrosine-protein kinase Yes | http://www.genecards.org/cgi-bin/carddisp.pl?gene=YES1 | 4.017535806 | 0.091589719 |
| *YWHAG* | 14-3-3 protein gamma | http://www.genecards.org/cgi-bin/carddisp.pl?gene=YWHAG | 7.326561854 | 0.050529995 |
| *ZBED9* | SCAN domain-containing protein 3 | http://www.genecards.org/cgi-bin/carddisp.pl?gene=ZBED9 | 0.230443115 | 0.200101895 |
| *ZC3H15* | Zinc finger CCCH domain-containing protein 15 | http://www.genecards.org/cgi-bin/carddisp.pl?gene=ZC3H15 | 0.860736753 | 0.237303611 |
| *ZMYND15* | Zinc finger MYND domain-containing protein 15 | http://www.genecards.org/cgi-bin/carddisp.pl?gene=ZMYND15 | 0.759894892 | 0.088955583 |
| *ZNF211* | Zinc finger protein 211 | http://www.genecards.org/cgi-bin/carddisp.pl?gene=ZNF211 | 0.668922803 | 0.168616528 |
| *ZNF529* | Zinc finger protein 529 | http://www.genecards.org/cgi-bin/carddisp.pl?gene=ZNF529 | 0.524486207 | 0.091439195 |
| *ZNF562* | Zinc finger protein 562 | http://www.genecards.org/cgi-bin/carddisp.pl?gene=ZNF562 | 1.172753874 | 0.311866556 |
| *ZNF648* | Zinc finger protein 648 | http://www.genecards.org/cgi-bin/carddisp.pl?gene=ZNF648 | 0.835154018 | 0.197242947 |
| *ZYG11B* | Protein zyg-11 homolog B | http://www.genecards.org/cgi-bin/carddisp.pl?gene=ZYG11B | 2.540911313 | 0.178681701 |
