# Supplementary material for: Centrosome guides spatial activation of Rac to control cell polarization and directed cell migration
Source: Life Sci Alliance. 2019 Feb 8;2(1):e201800135. doi: 10.26508/lsa.201800135 (PMC6369537; doi:10.26508/lsa.201800135)
Supplement: Supplementary file 8 [file LSA-2018-00135_TableS5.docx]

**Table S5. The list of proteins reproducibly identified in FAs of either RPEp53^-/-^ and RPEp53^-/-^STIL^-/-^ cells and their centrosome dependence ratios**

**Increased abundance in FA fraction isolated from RPEp53^-/-^ cells**

| **Gene name** | **Description** | **Gene information** | **Centrosome Dependence Ratio** | **p-value**  **(Student's t-test)** |
| --- | --- | --- | --- | --- |
| *A1BG* | Alpha-1B-glycoprotein | [http://www.genecards.org/cgi-bin/carddisp.pl?gene=A1BG](http://www.genecards.org/cgi-bin/carddisp.pl?gene=ACTR5) | only present in RPEp53-/- FA | 0.008915974 |
| *ACTBL2* | Beta-actin-like protein 2 | http://www.genecards.org/cgi-bin/carddisp.pl?gene=ACTBL2 | 1.858694388 | 0.016959873 |
| *ACTG1* | Actin, cytoplasmic 2 | http://www.genecards.org/cgi-bin/carddisp.pl?gene=ACTG1 | 1.679321752 | 0.001500982 |
| *ACTN1* | Alpha-actinin-1 | http://www.genecards.org/cgi-bin/carddisp.pl?gene=ACTN1 | 1.688951972 | 0.00471769 |
| *ACTN4* | Alpha-actinin-4 | http://www.genecards.org/cgi-bin/carddisp.pl?gene=ACTN4 | 2.008679887 | 0.000132959 |
| *ACTR3* | Actin-related protein 3 | http://www.genecards.org/cgi-bin/carddisp.pl?gene=ACTR3 | 1.945420155 | 0.009032623 |
| *ADAM10* | Disintegrin and metalloproteinase domain-containing protein 10 | http://www.genecards.org/cgi-bin/carddisp.pl?gene=ADAM10 | 1.957566962 | 0.004471167 |
| *ADAM8* | Disintegrin and metalloproteinase domain-containing protein 8 | http://www.genecards.org/cgi-bin/carddisp.pl?gene=ADAM8 | 2.628982739 | 0.003856478 |
| *ADAM9* | Disintegrin and metalloproteinase domain-containing protein 9 | http://www.genecards.org/cgi-bin/carddisp.pl?gene=ADAM9 | 3.39294566 | 0.005107484 |
| *ADAMTS5* | A disintegrin and metalloproteinase with thrombospondin motifs 5 | http://www.genecards.org/cgi-bin/carddisp.pl?gene=ADAMTS5 | 5.970027152 | 0.00283234 |
| *ADD1* | Alpha-adducin | http://www.genecards.org/cgi-bin/carddisp.pl?gene=ADD1 | 5.77071722 | 0.009221051 |
| *ADD3* | Gamma-adducin | http://www.genecards.org/cgi-bin/carddisp.pl?gene=ADD3 | 11.29244863 | 0.001250735 |
| *AHNAK* | Neuroblast differentiation-associated protein AHNAK | http://www.genecards.org/cgi-bin/carddisp.pl?gene=AHNAK | 1.657152415 | 0.029403899 |
| *AHNAK2* | Protein AHNAK2 | http://www.genecards.org/cgi-bin/carddisp.pl?gene=AHNAK2 | 1.725414068 | 0.010658993 |
| *AKAP12* | A-kinase anchor protein 12 | http://www.genecards.org/cgi-bin/carddisp.pl?gene=AKAP12 | 2.52116242 | 0.000432966 |
| *AKAP13* | A-kinase anchor protein 13 | http://www.genecards.org/cgi-bin/carddisp.pl?gene=AKAP13 | 1.74208827 | 0.022004512 |
| *ALCAM* | CD166 antigen | http://www.genecards.org/cgi-bin/carddisp.pl?gene=ALCAM | 2.415559255 | 0.016332937 |
| *ANKRD50* | Ankyrin repeat domain-containing protein 50 | http://www.genecards.org/cgi-bin/carddisp.pl?gene=ANKRD50 | 11.68646226 | 0.047513213 |
| *ANPEP* | Aminopeptidase N | http://www.genecards.org/cgi-bin/carddisp.pl?gene=ANPEP | 2.285640313 | 0.000197245 |
| *ANXA4* | Annexin A4 | http://www.genecards.org/cgi-bin/carddisp.pl?gene=ANXA4 | 2.84252671 | 0.009083155 |
| *AP2M1* | AP-2 complex subunit mu | http://www.genecards.org/cgi-bin/carddisp.pl?gene=AP2M1 | 1.64153663 | 0.038147186 |
| *APOB* | Apolipoprotein B-100 | http://www.genecards.org/cgi-bin/carddisp.pl?gene=APOB | 5.154605296 | 0.013628228 |
| *APOM* | Apolipoprotein M | http://www.genecards.org/cgi-bin/carddisp.pl?gene=APOM | 5.770082473 | 0.005456968 |
| *APP* | Amyloid beta A4 protein | http://www.genecards.org/cgi-bin/carddisp.pl?gene=APP | 2.515845402 | 0.006371482 |
| *ARHGEF6* | Rho guanine nucleotide exchange factor 6 | http://www.genecards.org/cgi-bin/carddisp.pl?gene=ARHGEF6 | 4.416802686 | 0.001108355 |
| *ARHGEF7* | Rho guanine nucleotide exchange factor 7 | http://www.genecards.org/cgi-bin/carddisp.pl?gene=ARHGEF7 | 13.57659877 | 0.002184008 |
| *ARPC1A* | Actin-related protein 2/3 complex subunit 1A | http://www.genecards.org/cgi-bin/carddisp.pl?gene=ARPC1A | 1.702778957 | 0.000377839 |
| *ARPC1B* | Actin-related protein 2/3 complex subunit 1B | http://www.genecards.org/cgi-bin/carddisp.pl?gene=ARPC1B | 3.376681998 | 0.000745878 |
| *ARPC2* | Actin-related protein 2/3 complex subunit 2 | http://www.genecards.org/cgi-bin/carddisp.pl?gene=ARPC2 | 2.762341613 | 0.002997517 |
| *ARPC3* | Actin-related protein 2/3 complex subunit 3 | http://www.genecards.org/cgi-bin/carddisp.pl?gene=ARPC3 | 2.201765941 | 0.006342606 |
| *ARPC4* | Actin-related protein 2/3 complex subunit 4 | http://www.genecards.org/cgi-bin/carddisp.pl?gene=ARPC4 | 4.577466296 | 1.78328E-05 |
| *ARPC5* | Actin-related protein 2/3 complex subunit 5 | http://www.genecards.org/cgi-bin/carddisp.pl?gene=ARPC5 | 2.796727012 | 0.004557944 |
| *ARPC5L* | Actin-related protein 2/3 complex subunit 5-like protein | http://www.genecards.org/cgi-bin/carddisp.pl?gene=ARPC5L | 1.967992628 | 0.019085246 |
| *ARSJ* | Arylsulfatase J | http://www.genecards.org/cgi-bin/carddisp.pl?gene=ARSJ | 1.789555774 | 0.012112189 |
| *ASPH* | Aspartyl/asparaginyl beta-hydroxylase | http://www.genecards.org/cgi-bin/carddisp.pl?gene=ASPH | 2.459956938 | 1.35966E-05 |
| *ATL3* | Atlastin-3 | http://www.genecards.org/cgi-bin/carddisp.pl?gene=ATL3 | 3.730306047 | 0.013057661 |
| *ATP1A1* | Sodium/potassium-transporting ATPase subunit alpha-1 | http://www.genecards.org/cgi-bin/carddisp.pl?gene=ATP1A1 | 1.37182138 | 0.008928209 |
| *ATP1A3* | Sodium/potassium-transporting ATPase subunit alpha-3 | http://www.genecards.org/cgi-bin/carddisp.pl?gene=ATP1A3 | 3.323137057 | 0.011209225 |
| *ATP1B3* | Sodium/potassium-transporting ATPase subunit beta-3 | http://www.genecards.org/cgi-bin/carddisp.pl?gene=ATP1B3 | 1.754724328 | 0.003377131 |
| *ATP5A1* | ATP synthase subunit alpha, mitochondrial | http://www.genecards.org/cgi-bin/carddisp.pl?gene=ATP5A1 | 2.43453897 | 0.047946963 |
| *ATP5B* | ATP synthase subunit beta, mitochondrial | http://www.genecards.org/cgi-bin/carddisp.pl?gene=ATP5B | 6.064686856 | 0.001134496 |
| *AXL* | Tyrosine-protein kinase receptor UFO | http://www.genecards.org/cgi-bin/carddisp.pl?gene=AXL | 1.557915479 | 0.02825913 |
| *BLK* | Tyrosine-protein kinase Blk | http://www.genecards.org/cgi-bin/carddisp.pl?gene=BLK | 3.835493787 | 0.045887075 |
| *BRD9* | Bromodomain-containing protein 9 | http://www.genecards.org/cgi-bin/carddisp.pl?gene=BRD9 | 3.229014233 | 0.008936096 |
| *BSG* | Basigin | http://www.genecards.org/cgi-bin/carddisp.pl?gene=BSG | 2.685593891 | 0.002593121 |
| *CA9* | Carbonic anhydrase 9 | http://www.genecards.org/cgi-bin/carddisp.pl?gene=CA9 | 8.460575922 | 0.001393252 |
| *CALD1* | Caldesmon | http://www.genecards.org/cgi-bin/carddisp.pl?gene=CALD1 | 1.388554637 | 0.001121261 |
| *CALM1* | Calmodulin | http://www.genecards.org/cgi-bin/carddisp.pl?gene=CALM1 | 1.674261343 | 0.020123546 |
| *CALR* | Calreticulin | http://www.genecards.org/cgi-bin/carddisp.pl?gene=CALR | 2.436669199 | 0.002367056 |
| *CALU* | Calumenin | http://www.genecards.org/cgi-bin/carddisp.pl?gene=CALU | 2.104807559 | 0.039011734 |
| *CAMK4* | Calcium/calmodulin-dependent protein kinase type IV | http://www.genecards.org/cgi-bin/carddisp.pl?gene=CAMK4 | 1.416754735 | 0.026880971 |
| *CANX* | Calnexin | http://www.genecards.org/cgi-bin/carddisp.pl?gene=CANX | 7.102265341 | 0.001918756 |
| *CAPZB* | F-actin-capping protein subunit beta | http://www.genecards.org/cgi-bin/carddisp.pl?gene=CAPZB | 3.39641707 | 3.53071E-05 |
| *CASK* | Peripheral plasma membrane protein CASK | http://www.genecards.org/cgi-bin/carddisp.pl?gene=CASK | 1.877025946 | 0.004090925 |
| *CASP12* | Inactive caspase-12 | http://www.genecards.org/cgi-bin/carddisp.pl?gene=CASP12 | 2.656361947 | 0.030494265 |
| *CAV1* | Caveolin-1 | http://www.genecards.org/cgi-bin/carddisp.pl?gene=CAV1 | 3.632133015 | 0.000562155 |
| *CAV2* | Caveolin-2 | http://www.genecards.org/cgi-bin/carddisp.pl?gene=CAV2 | 2.775193529 | 0.011801207 |
| *CCDC80* | Coiled-coil domain-containing protein 80 | http://www.genecards.org/cgi-bin/carddisp.pl?gene=CCDC80 | 2.191681121 | 0.000362312 |
| *CCNY* | Cyclin-Y | http://www.genecards.org/cgi-bin/carddisp.pl?gene=CCNY | 19.36333634 | 0.005855736 |
| *CD44* | CD44 antigen | http://www.genecards.org/cgi-bin/carddisp.pl?gene=CD44 | 1.594055999 | 0.004124344 |
| *CD58* | Lymphocyte function-associated antigen 3 | http://www.genecards.org/cgi-bin/carddisp.pl?gene=CD58 | 2.685343032 | 0.000574908 |
| *CD59* | CD59 glycoprotein | http://www.genecards.org/cgi-bin/carddisp.pl?gene=CD59 | 1.530564065 | 0.008743061 |
| *CD81* | CD81 antigen | http://www.genecards.org/cgi-bin/carddisp.pl?gene=CD81 | 1.288171206 | 0.013952757 |
| *CD99* | CD99 antigen | http://www.genecards.org/cgi-bin/carddisp.pl?gene=CD99 | 1.477506719 | 0.047888475 |
| *CDC42EP3* | Cdc42 effector protein 3 | http://www.genecards.org/cgi-bin/carddisp.pl?gene=CDC42EP3 | 6.003447613 | 0.010059272 |
| *CDH6* | Cadherin-6 | http://www.genecards.org/cgi-bin/carddisp.pl?gene=CDH6 | 2.677302681 | 0.042722803 |
| *CEP89* | Centrosomal protein of 89 kDa | http://www.genecards.org/cgi-bin/carddisp.pl?gene=CEP89 | 5.205298718 | 0.00414893 |
| *CFL2* | Cofilin-2 | http://www.genecards.org/cgi-bin/carddisp.pl?gene=CFL2 | 2.943782328 | 0.039774361 |
| *CHD7* | Chromodomain-helicase-DNA-binding protein 7 | http://www.genecards.org/cgi-bin/carddisp.pl?gene=CHD7 | 3.868758202 | 0.003556487 |
| *CHMP6* | Charged multivesicular body protein 6 | http://www.genecards.org/cgi-bin/carddisp.pl?gene=CHMP6 | 3.582317555 | 0.006562979 |
| *CLDND1* | Claudin domain-containing protein 1 | http://www.genecards.org/cgi-bin/carddisp.pl?gene=CLDND1 | 2.051677153 | 0.027380411 |
| *CLIP1* | CAP-Gly domain-containing linker protein 1 | http://www.genecards.org/cgi-bin/carddisp.pl?gene=CLIP1 | 2.703427162 | 0.002695064 |
| *CNN2* | Calponin-2 | http://www.genecards.org/cgi-bin/carddisp.pl?gene=CNN2 | 1.868049352 | 0.009014161 |
| *CNN3* | Calponin-3 | http://www.genecards.org/cgi-bin/carddisp.pl?gene=CNN3 | 3.780917683 | 0.033388441 |
| *COL4A5* | Collagen alpha-5(IV) chain | http://www.genecards.org/cgi-bin/carddisp.pl?gene=COL4A5 | 1.291238768 | 0.009100322 |
| *COL6A2* | Collagen alpha-2(VI) chain | http://www.genecards.org/cgi-bin/carddisp.pl?gene=COL6A2 | 2.819185512 | 0.043194112 |
| *COQ6* | Ubiquinone biosynthesis monooxygenase COQ6, mitochondrial | http://www.genecards.org/cgi-bin/carddisp.pl?gene=COQ6 | 3.690909799 | 0.000573579 |
| *CORO1B* | Coronin-1B | http://www.genecards.org/cgi-bin/carddisp.pl?gene=CORO1B | 1.709897906 | 0.002113966 |
| *CORO1C* | Coronin-1C | http://www.genecards.org/cgi-bin/carddisp.pl?gene=CORO1C | 3.34050247 | 0.006442529 |
| *CR1* | Complement receptor type 1 | http://www.genecards.org/cgi-bin/carddisp.pl?gene=CR1 | 1.700975555 | 0.016009249 |
| *CRK* | Adapter molecule crk | http://www.genecards.org/cgi-bin/carddisp.pl?gene=CRK | 4.793911844 | 0.00135081 |
| *CRLF3* | Cytokine receptor-like factor 3 | http://www.genecards.org/cgi-bin/carddisp.pl?gene=CRLF3 | 6.717079809 | 0.012925097 |
| *CROCC* | Rootletin | http://www.genecards.org/cgi-bin/carddisp.pl?gene=CROCC | 1.756265815 | 0.038345801 |
| *CSK* | Tyrosine-protein kinase CSK | http://www.genecards.org/cgi-bin/carddisp.pl?gene=CSK | 1.654722986 | 0.001184357 |
| *CSRP2* | Cysteine and glycine-rich protein 2 | http://www.genecards.org/cgi-bin/carddisp.pl?gene=CSRP2 | 4.622586267 | 0.00318514 |
| *CTNNA1* | Catenin alpha-1 | http://www.genecards.org/cgi-bin/carddisp.pl?gene=CTNNA1 | 2.19292729 | 0.000794174 |
| *CTNND1* | Catenin delta-1 | http://www.genecards.org/cgi-bin/carddisp.pl?gene=CTNND1 | 2.734198523 | 0.0005396 |
| *CTTN* | Src substrate cortactin | http://www.genecards.org/cgi-bin/carddisp.pl?gene=CTTN | 1.928499666 | 0.000137117 |
| *CTTNBP2NL* | CTTNBP2 N-terminal-like protein | http://www.genecards.org/cgi-bin/carddisp.pl?gene=CTTNBP2NL | 3.152897229 | 0.030845865 |
| *DAB2* | Disabled homolog 2 | http://www.genecards.org/cgi-bin/carddisp.pl?gene=DAB2 | 1.621175905 | 0.015228332 |
| *DBN1* | Drebrin | http://www.genecards.org/cgi-bin/carddisp.pl?gene=DBN1 | 2.097664943 | 0.002515928 |
| *DBNL* | Drebrin-like protein | http://www.genecards.org/cgi-bin/carddisp.pl?gene=DBNL | 3.007916817 | 0.007578994 |
| *DCBLD2* | Discoidin, CUB and LCCL domain-containing protein 2 | http://www.genecards.org/cgi-bin/carddisp.pl?gene=DCBLD2 | 4.779440065 | 0.001004382 |
| *DCST2* | DC-STAMP domain-containing protein 2 | http://www.genecards.org/cgi-bin/carddisp.pl?gene=DCST2 | 4.215007045 | 0.027740157 |
| *DHRS7C* | Dehydrogenase/reductase SDR family member 7C | http://www.genecards.org/cgi-bin/carddisp.pl?gene=DHRS7C | 2.966983655 | 0.008486467 |
| *DLG1* | Disks large homolog 1 | http://www.genecards.org/cgi-bin/carddisp.pl?gene=DLG1 | 2.488769491 | 0.000748376 |
| *DNAH2* | Dynein heavy chain 2, axonemal | http://www.genecards.org/cgi-bin/carddisp.pl?gene=DNAH2 | 5.700091267 | 0.033280005 |
| *DNAH9* | Dynein heavy chain 9, axonemal | http://www.genecards.org/cgi-bin/carddisp.pl?gene=DNAH9 | 1.594011115 | 0.043008436 |
| *DNAI1* | Dynein intermediate chain 1, axonemal | http://www.genecards.org/cgi-bin/carddisp.pl?gene=DNAI1 | 2.787707883 | 0.007370797 |
| *DOCK6* | Dedicator of cytokinesis protein 6 | http://www.genecards.org/cgi-bin/carddisp.pl?gene=DOCK6 | 5.199262569 | 0.022912112 |
| *DSG2* | Desmoglein-2 | http://www.genecards.org/cgi-bin/carddisp.pl?gene=DSG2 | 6.392876645 | 0.001328343 |
| *DYNLL1* | Dynein light chain 1, cytoplasmic | http://www.genecards.org/cgi-bin/carddisp.pl?gene=DYNLL1 | 2.072808763 | 0.018785596 |
| *ECE1* | Endothelin-converting enzyme 1 | http://www.genecards.org/cgi-bin/carddisp.pl?gene=ECE1 | 3.875748078 | 0.012617578 |
| *EDEM3* | ER degradation-enhancing alpha-mannosidase-like protein 3 | http://www.genecards.org/cgi-bin/carddisp.pl?gene=EDEM3 | 10.92259609 | 0.000165078 |
| *EEF1B2* | Elongation factor 1-beta | http://www.genecards.org/cgi-bin/carddisp.pl?gene=EEF1B2 | 2.152797007 | 0.017590667 |
| *EGFR* | Epidermal growth factor receptor | http://www.genecards.org/cgi-bin/carddisp.pl?gene=EGFR | 2.072798091 | 0.002942868 |
| *EHD4* | EH domain-containing protein 4 | http://www.genecards.org/cgi-bin/carddisp.pl?gene=EHD4 | 1.617964416 | 0.000644941 |
| *EIF1AX* | Eukaryotic translation initiation factor 1A, X-chromosomal | http://www.genecards.org/cgi-bin/carddisp.pl?gene=EIF1AX | 4.984431408 | 0.042802196 |
| *EIF3CL* | Eukaryotic translation initiation factor 3 subunit C-like protein | http://www.genecards.org/cgi-bin/carddisp.pl?gene=EIF3CL | 43.06393893 | 0.001876616 |
| *ENDOD1* | Endonuclease domain-containing 1 protein | http://www.genecards.org/cgi-bin/carddisp.pl?gene=ENDOD1 | 14.89343802 | 0.000934843 |
| *EPB41L2* | Band 4.1-like protein 2 | http://www.genecards.org/cgi-bin/carddisp.pl?gene=EPB41L2 | 3.521964966 | 0.000816946 |
| *EPHA2* | Ephrin type-A receptor 2 | http://www.genecards.org/cgi-bin/carddisp.pl?gene=EPHA2 | 1.692905843 | 0.002169651 |
| *EPHA3* | Ephrin type-A receptor 3 | http://www.genecards.org/cgi-bin/carddisp.pl?gene=EPHA3 | 1.499305492 | 0.04968208 |
| *EPHB2* | Ephrin type-B receptor 2 | http://www.genecards.org/cgi-bin/carddisp.pl?gene=EPHB2 | 5.899548092 | 4.57939E-05 |
| *ERBB2* | Receptor tyrosine-protein kinase erbB-2 | http://www.genecards.org/cgi-bin/carddisp.pl?gene=ERBB2 | 2.620045631 | 0.010578633 |
| *ERLIN2* | Erlin-2 | http://www.genecards.org/cgi-bin/carddisp.pl?gene=ERLIN2 | 3.477728012 | 0.000332023 |
| *ERP29* | Endoplasmic reticulum resident protein 29 | http://www.genecards.org/cgi-bin/carddisp.pl?gene=ERP29 | 38.78539529 | 0.000650484 |
| *ESYT1* | Extended synaptotagmin-1 | http://www.genecards.org/cgi-bin/carddisp.pl?gene=ESYT1 | 9.590112031 | 0.000538601 |
| *EVA1A* | Protein eva-1 homolog A | http://www.genecards.org/cgi-bin/carddisp.pl?gene=EVA1A | 2.352989342 | 0.014316957 |
| *EZR* | Ezrin | http://www.genecards.org/cgi-bin/carddisp.pl?gene=EZR | 1.444214924 | 0.00646426 |
| *F11R* | Junctional adhesion molecule A | http://www.genecards.org/cgi-bin/carddisp.pl?gene=F11R | 13.66028659 | 0.02137313 |
| *F3* | Tissue factor | http://www.genecards.org/cgi-bin/carddisp.pl?gene=F3 | 2.208252122 | 2.01824E-05 |
| *FAM129B* | Niban-like protein 1 | http://www.genecards.org/cgi-bin/carddisp.pl?gene=FAM129B | 6.20580386 | 0.033805184 |
| *FAM160A1* | Protein FAM160A1 | http://www.genecards.org/cgi-bin/carddisp.pl?gene=FAM160A1 | 15.5516419 | 0.02472671 |
| *FAM160B2* | Protein FAM160B2 | http://www.genecards.org/cgi-bin/carddisp.pl?gene=FAM160B2 | 2.273313352 | 0.002677875 |
| *FAM186A* | Protein FAM186A | http://www.genecards.org/cgi-bin/carddisp.pl?gene=FAM186A | 5.867526086 | 0.025092129 |
| *FAS* | Tumor necrosis factor receptor superfamily member 6 | http://www.genecards.org/cgi-bin/carddisp.pl?gene=FAS | 1.820466485 | 0.031272066 |
| *FBLIM1* | Filamin-binding LIM protein 1 | http://www.genecards.org/cgi-bin/carddisp.pl?gene=FBLIM1 | 2.606422014 | 0.00280705 |
| *FERMT2* | Fermitin family homolog 2 | http://www.genecards.org/cgi-bin/carddisp.pl?gene=FERMT2 | 1.798844038 | 0.027153534 |
| *FHL2* | Four and a half LIM domains protein 2 | http://www.genecards.org/cgi-bin/carddisp.pl?gene=FHL2 | 4.668036462 | 0.000224072 |
| *FHL3* | Four and a half LIM domains protein 3 | http://www.genecards.org/cgi-bin/carddisp.pl?gene=FHL3 | 8.086028406 | 0.01929067 |
| *FLG2* | Filaggrin-2 | http://www.genecards.org/cgi-bin/carddisp.pl?gene=FLG2 | 5.024969458 | 0.001887526 |
| *FLII* | Protein flightless-1 homolog | http://www.genecards.org/cgi-bin/carddisp.pl?gene=FLII | 5.813765125 | 0.017086824 |
| *FLNA* | Filamin-A | http://www.genecards.org/cgi-bin/carddisp.pl?gene=FLNA | 2.051875868 | 0.005701202 |
| *FLNB* | Filamin-B | http://www.genecards.org/cgi-bin/carddisp.pl?gene=FLNB | 2.554017258 | 0.002471891 |
| *FLNC* | Filamin-C | http://www.genecards.org/cgi-bin/carddisp.pl?gene=FLNC | 1.972616986 | 0.000297516 |
| *FLOT1* | Flotillin-1 | http://www.genecards.org/cgi-bin/carddisp.pl?gene=FLOT1 | 1.717239058 | 0.012578457 |
| *FMNL3* | Formin-like protein 3 | http://www.genecards.org/cgi-bin/carddisp.pl?gene=FMNL3 | 4.917189539 | 0.021644044 |
| *FSCN1* | Fascin | http://www.genecards.org/cgi-bin/carddisp.pl?gene=FSCN1 | 2.431808247 | 0.000172131 |
| *FXR1* | Fragile X mental retardation syndrome-related protein 1 | http://www.genecards.org/cgi-bin/carddisp.pl?gene=FXR1 | 1.488213448 | 0.022776481 |
| *FZD7* | Frizzled-7 | http://www.genecards.org/cgi-bin/carddisp.pl?gene=FZD7 | 2.083464103 | 0.004092501 |
| *GANAB* | Neutral alpha-glucosidase AB | http://www.genecards.org/cgi-bin/carddisp.pl?gene=GANAB | only present in RPEp53-/- FA | 0.018625285 |
| *GGCT* | Gamma-glutamylcyclotransferase | http://www.genecards.org/cgi-bin/carddisp.pl?gene=GGCT | 6.718536813 | 0.001647534 |
| *GLYR1* | Putative oxidoreductase GLYR1 | http://www.genecards.org/cgi-bin/carddisp.pl?gene=GLYR1 | 14.19150259 | 0.000829567 |
| *GNA11* | Guanine nucleotide-binding protein subunit alpha-11 | http://www.genecards.org/cgi-bin/carddisp.pl?gene=GNA11 | 2.368982797 | 0.014804817 |
| *GNA13* | Guanine nucleotide-binding protein subunit alpha-13 | http://www.genecards.org/cgi-bin/carddisp.pl?gene=GNA13 | 1.917101337 | 0.008464953 |
| *GNAI2* | Guanine nucleotide-binding protein G(i) subunit alpha-2 | http://www.genecards.org/cgi-bin/carddisp.pl?gene=GNAI2 | 1.470650072 | 0.048018811 |
| *GNAI3* | Guanine nucleotide-binding protein G(k) subunit alpha | http://www.genecards.org/cgi-bin/carddisp.pl?gene=GNAI3 | 8.933054385 | 0.004327287 |
| *GNB1* | Guanine nucleotide-binding protein G(I)/G(S)/G(T) subunit beta-1 | http://www.genecards.org/cgi-bin/carddisp.pl?gene=GNB1 | 2.683741049 | 0.003555221 |
| *GNB2* | Guanine nucleotide-binding protein G(I)/G(S)/G(T) subunit beta-2 | http://www.genecards.org/cgi-bin/carddisp.pl?gene=GNB2 | 2.042107546 | 0.015797131 |
| *GNG12* | Guanine nucleotide-binding protein G(I)/G(S)/G(O) subunit gamma-12 | http://www.genecards.org/cgi-bin/carddisp.pl?gene=GNG12 | 1.198683916 | 0.031791553 |
| *GOLGA4* | Golgin subfamily A member 4 | http://www.genecards.org/cgi-bin/carddisp.pl?gene=GOLGA4 | 8.204929328 | 0.022840564 |
| *GOLGA7* | Golgin subfamily A member 7 | http://www.genecards.org/cgi-bin/carddisp.pl?gene=GOLGA7 | 3.041703176 | 0.000389428 |
| *GPC1* | Glypican-1 | http://www.genecards.org/cgi-bin/carddisp.pl?gene=GPC1 | 7.073791604 | 0.002939333 |
| *GPR176* | Probable G-protein coupled receptor 176 | http://www.genecards.org/cgi-bin/carddisp.pl?gene=GPR176 | 3.840007794 | 8.56112E-05 |
| *GRM4* | Metabotropic glutamate receptor 4 | http://www.genecards.org/cgi-bin/carddisp.pl?gene=GRM4 | 5.690540287 | 0.019565833 |
| *GSTP1* | Glutathione S-transferase P | http://www.genecards.org/cgi-bin/carddisp.pl?gene=GSTP1 | 41.75515486 | 0.000549138 |
| *HIP1R* | Huntingtin-interacting protein 1-related protein | http://www.genecards.org/cgi-bin/carddisp.pl?gene=HIP1R | 2.222878832 | 0.004646635 |
| *HIST1H1B* | Histone H1.5 | http://www.genecards.org/cgi-bin/carddisp.pl?gene=HIST1H1B | 4.199185098 | 0.014697558 |
| *HLA-B* | HLA class I histocompatibility antigen, B-48 alpha chain | http://www.genecards.org/cgi-bin/carddisp.pl?gene=HLA-B | 21.04683987 | 0.01836974 |
| *HLA-C* | HLA class I histocompatibility antigen, Cw-17 alpha chain | http://www.genecards.org/cgi-bin/carddisp.pl?gene=HLA-C | 2.360552071 | 0.02844546 |
| *HSP90AA2P* | Heat shock protein HSP 90-alpha A2 | http://www.genecards.org/cgi-bin/carddisp.pl?gene=HSP90AA2P | 2.219403301 | 0.025295891 |
| *HSP90B1* | Endoplasmin | http://www.genecards.org/cgi-bin/carddisp.pl?gene=HSP90B1 | 6.028680421 | 0.002307536 |
| *HSPA5* | 78 kDa glucose-regulated protein | http://www.genecards.org/cgi-bin/carddisp.pl?gene=HSPA5 | 3.706551503 | 0.000446286 |
| *HSPA9* | Stress-70 protein, mitochondrial | http://www.genecards.org/cgi-bin/carddisp.pl?gene=HSPA9 | 3.880820382 | 0.026599893 |
| *HSPE1* | 10 kDa heat shock protein, mitochondrial | http://www.genecards.org/cgi-bin/carddisp.pl?gene=HSPE1 | 2.247948679 | 0.038373334 |
| *HTRA1* | Serine protease HTRA1 | http://www.genecards.org/cgi-bin/carddisp.pl?gene=HTRA1 | 6.163654022 | 0.007289322 |
| *ICAM3* | Intercellular adhesion molecule 3 | http://www.genecards.org/cgi-bin/carddisp.pl?gene=ICAM3 | 13.34380822 | 0.03806318 |
| *IGFBP3* | Insulin-like growth factor-binding protein 3 | http://www.genecards.org/cgi-bin/carddisp.pl?gene=IGFBP3 | 1.647826119 | 0.00342898 |
| *IGSF8* | Immunoglobulin superfamily member 8 | http://www.genecards.org/cgi-bin/carddisp.pl?gene=IGSF8 | 5.049260805 | 0.009593285 |
| *ILK* | Integrin-linked protein kinase | http://www.genecards.org/cgi-bin/carddisp.pl?gene=ILK | 1.488138031 | 0.00433295 |
| *ITGA3* | Integrin alpha-3 | http://www.genecards.org/cgi-bin/carddisp.pl?gene=ITGA3 | 3.685566539 | 0.001388494 |
| *ITGA5* | Integrin alpha-5 | http://www.genecards.org/cgi-bin/carddisp.pl?gene=ITGA5 | 1.626404136 | 0.000221843 |
| *ITGAV* | Integrin alpha-V | http://www.genecards.org/cgi-bin/carddisp.pl?gene=ITGAV | 1.846999938 | 0.010469949 |
| *ITGB1* | Integrin beta-1 | http://www.genecards.org/cgi-bin/carddisp.pl?gene=ITGB1 | 1.631113598 | 2.9224E-05 |
| *ITGB2* | Integrin beta-2 | http://www.genecards.org/cgi-bin/carddisp.pl?gene=ITGB2 | 3.390256848 | 0.0034284 |
| *ITGB5* | Integrin beta-5 | http://www.genecards.org/cgi-bin/carddisp.pl?gene=ITGB5 | 1.409088867 | 0.038362779 |
| *ITSN2* | Intersectin-2 | http://www.genecards.org/cgi-bin/carddisp.pl?gene=ITSN2 | 4.021349674 | 0.029570702 |
| *KANK2* | KN motif and ankyrin repeat domain-containing protein 2 | http://www.genecards.org/cgi-bin/carddisp.pl?gene=KANK2 | 1.592776846 | 0.013341032 |
| *KIF17* | Kinesin-like protein KIF17 | http://www.genecards.org/cgi-bin/carddisp.pl?gene=KIF17 | 5.160622399 | 0.010241562 |
| *KLHL17* | Kelch-like protein 17 | http://www.genecards.org/cgi-bin/carddisp.pl?gene=KLHL17 | 4.487675971 | 0.00025616 |
| *KRT18* | Keratin, type I cytoskeletal 18 | http://www.genecards.org/cgi-bin/carddisp.pl?gene=KRT18 | 3.436072327 | 0.004329707 |
| *KRT3* | Keratin, type II cytoskeletal 3 | http://www.genecards.org/cgi-bin/carddisp.pl?gene=KRT3 | 2.471212775 | 0.008215758 |
| *KRT8* | Keratin, type II cytoskeletal 8 | http://www.genecards.org/cgi-bin/carddisp.pl?gene=KRT8 | 2.808434204 | 0.000989528 |
| *KRT80* | Keratin, type II cytoskeletal 80 | http://www.genecards.org/cgi-bin/carddisp.pl?gene=KRT80 | 1.858577531 | 0.013297912 |
| *KRT85* | Keratin, type II cuticular Hb5 | http://www.genecards.org/cgi-bin/carddisp.pl?gene=KRT85 | 1.722039879 | 0.038989504 |
| *LAMB1* | Laminin subunit beta-1 | http://www.genecards.org/cgi-bin/carddisp.pl?gene=LAMB1 | 2.424432885 | 0.004949399 |
| *LAMP2* | Lysosome-associated membrane glycoprotein 2 | http://www.genecards.org/cgi-bin/carddisp.pl?gene=LAMP2 | 4.760262197 | 0.000693363 |
| *LANCL1* | LanC-like protein 1 | http://www.genecards.org/cgi-bin/carddisp.pl?gene=LANCL1 | 2.688088677 | 0.002155141 |
| *LASP1* | LIM and SH3 domain protein 1 | http://www.genecards.org/cgi-bin/carddisp.pl?gene=LASP1 | 1.647243076 | 4.25269E-05 |
| *LGI1* | Leucine-rich glioma-inactivated protein 1 | http://www.genecards.org/cgi-bin/carddisp.pl?gene=LGI1 | 4.164388776 | 0.010790459 |
| *LIMA1* | LIM domain and actin-binding protein 1 | http://www.genecards.org/cgi-bin/carddisp.pl?gene=LIMA1 | 2.700698679 | 0.011313099 |
| *LIMCH1* | LIM and calponin homology domains-containing protein 1 | http://www.genecards.org/cgi-bin/carddisp.pl?gene=LIMCH1 | 9.495272145 | 0.000538814 |
| *LIN7C* | Protein lin-7 homolog C | http://www.genecards.org/cgi-bin/carddisp.pl?gene=LIN7C | 3.181217602 | 0.002864607 |
| *LMO7* | LIM domain only protein 7 | http://www.genecards.org/cgi-bin/carddisp.pl?gene=LMO7 | 1.935871847 | 0.007716774 |
| *LPP* | Lipoma-preferred partner | http://www.genecards.org/cgi-bin/carddisp.pl?gene=LPP | 2.067513424 | 0.031042351 |
| *LRRC17* | Leucine-rich repeat-containing protein 17 | http://www.genecards.org/cgi-bin/carddisp.pl?gene=LRRC17 | 1.346482659 | 0.005565898 |
| *LTBP4* | Latent-transforming growth factor beta-binding protein 4 | http://www.genecards.org/cgi-bin/carddisp.pl?gene=LTBP4 | 1.621180727 | 0.000275201 |
| *MAGI3* | Membrane-associated guanylate kinase, WW and PDZ domain-containing protein 3 | http://www.genecards.org/cgi-bin/carddisp.pl?gene=MAGI3 | 2.39425879 | 0.045390979 |
| *MAP1A* | Microtubule-associated protein 1A | http://www.genecards.org/cgi-bin/carddisp.pl?gene=MAP1A | 4.787744244 | 0.015648947 |
| *MDN1* | Midasin | http://www.genecards.org/cgi-bin/carddisp.pl?gene=MDN1 | 4.520208682 | 0.011269564 |
| *MET* | Hepatocyte growth factor receptor | http://www.genecards.org/cgi-bin/carddisp.pl?gene=MET | 1.918634438 | 0.004835112 |
| *METTL7B* | Methyltransferase-like protein 7B | http://www.genecards.org/cgi-bin/carddisp.pl?gene=METTL7B | 5.372417245 | 0.035724079 |
| *MFGE8* | Lactadherin | http://www.genecards.org/cgi-bin/carddisp.pl?gene=MFGE8 | 4.388351535 | 0.018900923 |
| *MME* | Neprilysin | http://www.genecards.org/cgi-bin/carddisp.pl?gene=MME | 1.664783902 | 0.032816192 |
| *MMP15* | Matrix metalloproteinase-15 | http://www.genecards.org/cgi-bin/carddisp.pl?gene=MMP15 | 10.27686231 | 0.044002447 |
| *MOB1A* | MOB kinase activator 1A | http://www.genecards.org/cgi-bin/carddisp.pl?gene=MOB1A | 1.450089083 | 0.012104662 |
| *MPP6* | MAGUK p55 subfamily member 6 | http://www.genecards.org/cgi-bin/carddisp.pl?gene=MPP6 | 3.837100602 | 1.42304E-05 |
| *MPZL1* | Myelin protein zero-like protein 1 | http://www.genecards.org/cgi-bin/carddisp.pl?gene=MPZL1 | 1.511068203 | 0.045162799 |
| *MRC2* | C-type mannose receptor 2 | http://www.genecards.org/cgi-bin/carddisp.pl?gene=MRC2 | 2.337251667 | 0.002477085 |
| *MTOR* | Serine/threonine-protein kinase mTOR | http://www.genecards.org/cgi-bin/carddisp.pl?gene=MTOR | 1.808265864 | 0.013198004 |
| *MXRA8* | Matrix-remodeling-associated protein 8 | http://www.genecards.org/cgi-bin/carddisp.pl?gene=MXRA8 | 10.06023992 | 0.003344922 |
| *MYADM* | Myeloid-associated differentiation marker | http://www.genecards.org/cgi-bin/carddisp.pl?gene=MYADM | 4.3160817 | 0.021053777 |
| *MYH10* | Myosin-10 | http://www.genecards.org/cgi-bin/carddisp.pl?gene=MYH10 | 4.636537909 | 0.000901891 |
| *MYH9* | Myosin-9 | http://www.genecards.org/cgi-bin/carddisp.pl?gene=MYH9 | 1.68976624 | 0.003364832 |
| *MYL12A* | Myosin regulatory light chain 12A | http://www.genecards.org/cgi-bin/carddisp.pl?gene=MYL12A | 2.7417023 | 1.37692E-05 |
| *MYL6* | Myosin light polypeptide 6 | http://www.genecards.org/cgi-bin/carddisp.pl?gene=MYL6 | 2.848302282 | 4.16651E-05 |
| *MYL9* | Myosin regulatory light polypeptide 9 | http://www.genecards.org/cgi-bin/carddisp.pl?gene=MYL9 | 2.167987795 | 0.001152552 |
| *MYOF* | Myoferlin | http://www.genecards.org/cgi-bin/carddisp.pl?gene=MYOF | 2.219751165 | 0.00032508 |
| *NCEH1* | Neutral cholesterol ester hydrolase 1 | http://www.genecards.org/cgi-bin/carddisp.pl?gene=NCEH1 | 4.316887223 | 0.006312478 |
| *NCK1* | Cytoplasmic protein NCK1 | http://www.genecards.org/cgi-bin/carddisp.pl?gene=NCK1 | 52.84277016 | 0.012382486 |
| *NCOR2* | Nuclear receptor corepressor 2 | http://www.genecards.org/cgi-bin/carddisp.pl?gene=NCOR2 | 3.340968658 | 0.012932967 |
| *NECAP2* | Adaptin ear-binding coat-associated protein 2 | http://www.genecards.org/cgi-bin/carddisp.pl?gene=NECAP2 | 6.637649607 | 0.027858417 |
| *NEXN* | Nexilin | http://www.genecards.org/cgi-bin/carddisp.pl?gene=NEXN | 1.190805008 | 0.018073933 |
| *NPTN* | Neuroplastin | http://www.genecards.org/cgi-bin/carddisp.pl?gene=NPTN | 1.662828564 | 0.010592932 |
| *NRP1* | Neuropilin-1 | http://www.genecards.org/cgi-bin/carddisp.pl?gene=NRP1 | 1.410853909 | 0.00364931 |
| *NSDHL* | Sterol-4-alpha-carboxylate 3-dehydrogenase, decarboxylating | http://www.genecards.org/cgi-bin/carddisp.pl?gene=NSDHL | 6.2333757 | 0.00367783 |
| *NT5E* | 5'-nucleotidase | http://www.genecards.org/cgi-bin/carddisp.pl?gene=NT5E | 1.934722296 | 0.003428459 |
| *ODF2* | Outer dense fiber protein 2 | http://www.genecards.org/cgi-bin/carddisp.pl?gene=ODF2 | 2.469075805 | 0.006853101 |
| *OR1B1* | Olfactory receptor 1B1 | http://www.genecards.org/cgi-bin/carddisp.pl?gene=OR1B1 | 20.33958933 | 0.00778558 |
| *OSBPL8* | Oxysterol-binding protein-related protein 8 | http://www.genecards.org/cgi-bin/carddisp.pl?gene=OSBPL8 | 3.436764011 | 6.09212E-05 |
| *PA2G4* | Proliferation-associated protein 2G4 | http://www.genecards.org/cgi-bin/carddisp.pl?gene=PA2G4 | 1.774087906 | 0.001286629 |
| *PABPC1* | Polyadenylate-binding protein 1 | http://www.genecards.org/cgi-bin/carddisp.pl?gene=PABPC1 | 4.221751396 | 0.014452896 |
| *PABPC4* | Polyadenylate-binding protein 4 | http://www.genecards.org/cgi-bin/carddisp.pl?gene=PABPC4 | 36.20929797 | 0.017907893 |
| *PALLD* | Palladin | http://www.genecards.org/cgi-bin/carddisp.pl?gene=PALLD | 2.195022801 | 0.001625217 |
| *PAWR* | PRKC apoptosis WT1 regulator protein | http://www.genecards.org/cgi-bin/carddisp.pl?gene=PAWR | 2.689668812 | 0.002274642 |
| *PCBP2* | Poly(rC)-binding protein 2 | http://www.genecards.org/cgi-bin/carddisp.pl?gene=PCBP2 | 59.22286448 | 0.000563692 |
| *PCSK9* | Proprotein convertase subtilisin/kexin type 9 | http://www.genecards.org/cgi-bin/carddisp.pl?gene=PCSK9 | 2.175236192 | 0.000549713 |
| *PDIA6* | Protein disulfide-isomerase A6 | http://www.genecards.org/cgi-bin/carddisp.pl?gene=PDIA6 | 3.19286588 | 0.001259826 |
| *PDLIM1* | PDZ and LIM domain protein 1 | http://www.genecards.org/cgi-bin/carddisp.pl?gene=PDLIM1 | 4.285692049 | 0.001466718 |
| *PDLIM2* | PDZ and LIM domain protein 2 | http://www.genecards.org/cgi-bin/carddisp.pl?gene=PDLIM2 | 30.3696112 | 0.00035844 |
| *PDLIM4* | PDZ and LIM domain protein 4 | http://www.genecards.org/cgi-bin/carddisp.pl?gene=PDLIM4 | 3.980777601 | 0.000179427 |
| *PDLIM5* | PDZ and LIM domain protein 5 | http://www.genecards.org/cgi-bin/carddisp.pl?gene=PDLIM5 | 2.238311917 | 0.005101557 |
| *PDLIM7* | PDZ and LIM domain protein 7 | http://www.genecards.org/cgi-bin/carddisp.pl?gene=PDLIM7 | 2.534104611 | 0.001334949 |
| *PFDN2* | Prefoldin subunit 2 | http://www.genecards.org/cgi-bin/carddisp.pl?gene=PFDN2 | 14.31720603 | 0.003583805 |
| *PGRMC1* | Membrane-associated progesterone receptor component 1 | http://www.genecards.org/cgi-bin/carddisp.pl?gene=PGRMC1 | 41.75202981 | 0.007139922 |
| *PHLDA1* | Pleckstrin homology-like domain family A member 1 | http://www.genecards.org/cgi-bin/carddisp.pl?gene=PHLDA1 | 2.339286638 | 0.014957166 |
| *PLAG1* | Zinc finger protein PLAG1 | http://www.genecards.org/cgi-bin/carddisp.pl?gene=PLAG1 | 10.22534172 | 0.002738822 |
| *PLAU* | Urokinase-type plasminogen activator | http://www.genecards.org/cgi-bin/carddisp.pl?gene=PLAU | 1.569868729 | 0.006075542 |
| *PLEC* | Plectin | http://www.genecards.org/cgi-bin/carddisp.pl?gene=PLEC | 1.464921594 | 0.023171089 |
| *PLP2* | Proteolipid protein 2 | http://www.genecards.org/cgi-bin/carddisp.pl?gene=PLP2 | 1.96763762 | 0.004672068 |
| *PLSCR1* | Phospholipid scramblase 1 | http://www.genecards.org/cgi-bin/carddisp.pl?gene=PLSCR1 | 1.527574367 | 0.037544598 |
| *PLXNA3* | Plexin-A3 | http://www.genecards.org/cgi-bin/carddisp.pl?gene=PLXNA3 | 3.864345296 | 0.001368652 |
| *PLXNB2* | Plexin-B2 | http://www.genecards.org/cgi-bin/carddisp.pl?gene=PLXNB2 | 2.9800584 | 3.25448E-05 |
| *PODXL* | Podocalyxin | http://www.genecards.org/cgi-bin/carddisp.pl?gene=PODXL | 1.954054577 | 0.010817204 |
| *PPFIA1* | Liprin-alpha-1 | http://www.genecards.org/cgi-bin/carddisp.pl?gene=PPFIA1 | 2.371143133 | 0.006084276 |
| *PPIB* | Peptidyl-prolyl cis-trans isomerase B | http://www.genecards.org/cgi-bin/carddisp.pl?gene=PPIB | 40.3932121 | 0.006670207 |
| *PPIP5K2* | Inositol hexakisphosphate and diphosphoinositol-pentakisphosphate kinase 2 | http://www.genecards.org/cgi-bin/carddisp.pl?gene=PPIP5K2 | 5.913717744 | 0.00300664 |
| *PPP1CC* | Serine/threonine-protein phosphatase PP1-gamma catalytic subunit | http://www.genecards.org/cgi-bin/carddisp.pl?gene=PPP1CC | 1.918214651 | 0.018423953 |
| *PPP1R12A* | Protein phosphatase 1 regulatory subunit 12A | http://www.genecards.org/cgi-bin/carddisp.pl?gene=PPP1R12A | 2.704772215 | 5.36644E-06 |
| *PPP1R18* | Phostensin | http://www.genecards.org/cgi-bin/carddisp.pl?gene=PPP1R18 | 2.145285115 | 0.000111987 |
| *PRDX1* | Peroxiredoxin-1 | http://www.genecards.org/cgi-bin/carddisp.pl?gene=PRDX1 | 2.082458024 | 9.29339E-05 |
| *PRDX6* | Peroxiredoxin-6 | http://www.genecards.org/cgi-bin/carddisp.pl?gene=PRDX6 | 1.858184694 | 0.009266235 |
| *PRKCSH* | Glucosidase 2 subunit beta | http://www.genecards.org/cgi-bin/carddisp.pl?gene=PRKCSH | 2.189188254 | 0.004317949 |
| *PRNP* | Major prion protein | http://www.genecards.org/cgi-bin/carddisp.pl?gene=PRNP | 3.21842496 | 0.000120971 |
| *PROCR* | Endothelial protein C receptor | http://www.genecards.org/cgi-bin/carddisp.pl?gene=PROCR | 7.621610172 | 0.002175209 |
| *PSEN1* | Presenilin-1 | http://www.genecards.org/cgi-bin/carddisp.pl?gene=PSEN1 | 3.841167597 | 0.035970478 |
| *PSMA3* | Proteasome subunit alpha type-3 | http://www.genecards.org/cgi-bin/carddisp.pl?gene=PSMA3 | 21.24960346 | 0.041769437 |
| *PSMD7* | 26S proteasome non-ATPase regulatory subunit 7 | http://www.genecards.org/cgi-bin/carddisp.pl?gene=PSMD7 | 2.839833497 | 0.007675468 |
| *PTK2* | Focal adhesion kinase 1 | http://www.genecards.org/cgi-bin/carddisp.pl?gene=PTK2 | 3.980735274 | 0.001257661 |
| *PTK7* | Inactive tyrosine-protein kinase 7 | http://www.genecards.org/cgi-bin/carddisp.pl?gene=PTK7 | 2.285662456 | 0.003075415 |
| *PTPRJ* | Receptor-type tyrosine-protein phosphatase eta | http://www.genecards.org/cgi-bin/carddisp.pl?gene=PTPRJ | 5.034174937 | 0.024327649 |
| *PTRF* | Polymerase I and transcript release factor | http://www.genecards.org/cgi-bin/carddisp.pl?gene=PTRF | 3.192212137 | 0.026824115 |
| *PVR* | Poliovirus receptor | http://www.genecards.org/cgi-bin/carddisp.pl?gene=PVR | 1.374605068 | 0.030247023 |
| *PXN* | Paxillin | http://www.genecards.org/cgi-bin/carddisp.pl?gene=PXN | 4.452045597 | 0.002854497 |
| *QSOX2* | Sulfhydryl oxidase 2 | http://www.genecards.org/cgi-bin/carddisp.pl?gene=QSOX2 | 3.919476499 | 0.000580881 |
| *RAB10* | Ras-related protein Rab-10 | http://www.genecards.org/cgi-bin/carddisp.pl?gene=RAB10 | 1.78681178 | 0.009145376 |
| *RAB11A* | Ras-related protein Rab-11A | http://www.genecards.org/cgi-bin/carddisp.pl?gene=RAB11A | 1.72991339 | 0.027861468 |
| *RAB2B* | Ras-related protein Rab-2B | http://www.genecards.org/cgi-bin/carddisp.pl?gene=RAB2B | 1.892319255 | 0.032120221 |
| *RAB34* | Ras-related protein Rab-34 | http://www.genecards.org/cgi-bin/carddisp.pl?gene=RAB34 | 2.644299917 | 0.003104441 |
| *RAB40C* | Ras-related protein Rab-40C | http://www.genecards.org/cgi-bin/carddisp.pl?gene=RAB40C | 9.687896324 | 0.000193001 |
| *RAC1* | Ras-related C3 botulinum toxin substrate 1 | http://www.genecards.org/cgi-bin/carddisp.pl?gene=RAC1 | 2.293788914 | 0.007502384 |
| *RAP1A* | Ras-related protein Rap-1A | http://www.genecards.org/cgi-bin/carddisp.pl?gene=RAP1A | 2.172900298 | 0.001504838 |
| *RAP1B* | Ras-related protein Rap-1b | http://www.genecards.org/cgi-bin/carddisp.pl?gene=RAP1B | 1.59464686 | 0.041767988 |
| *RAPH1* | Ras-associated and pleckstrin homology domains-containing protein 1 | http://www.genecards.org/cgi-bin/carddisp.pl?gene=RAPH1 | 2.261912562 | 0.005403856 |
| *RASA3* | Ras GTPase-activating protein 3 | http://www.genecards.org/cgi-bin/carddisp.pl?gene=RASA3 | 7.980037335 | 0.002065938 |
| *RCN1* | Reticulocalbin-1 | http://www.genecards.org/cgi-bin/carddisp.pl?gene=RCN1 | 8.680407852 | 0.025230019 |
| *RFLNB* | Refilin-B | http://www.genecards.org/cgi-bin/carddisp.pl?gene=RFLNB | 21.67211164 | 0.000428693 |
| *RHOA* | Transforming protein RhoA | http://www.genecards.org/cgi-bin/carddisp.pl?gene=RHOA | 2.732793383 | 0.013462041 |
| *RP2* | Protein XRP2 | http://www.genecards.org/cgi-bin/carddisp.pl?gene=RP2 | 4.746542823 | 0.000509152 |
| *RPL10* | 60S ribosomal protein L10 | http://www.genecards.org/cgi-bin/carddisp.pl?gene=RPL10 | 1.49307554 | 0.037117195 |
| *RPL11* | 60S ribosomal protein L11 | http://www.genecards.org/cgi-bin/carddisp.pl?gene=RPL11 | 3.626137444 | 0.00106086 |
| *RPL12* | 60S ribosomal protein L12 | http://www.genecards.org/cgi-bin/carddisp.pl?gene=RPL12 | 1.403860943 | 0.002837515 |
| *RPL13* | 60S ribosomal protein L13 | http://www.genecards.org/cgi-bin/carddisp.pl?gene=RPL13 | 2.149295157 | 0.000104074 |
| *RPL14* | 60S ribosomal protein L14 | http://www.genecards.org/cgi-bin/carddisp.pl?gene=RPL14 | 5.349923548 | 0.00045106 |
| *RPL18* | 60S ribosomal protein L18 | http://www.genecards.org/cgi-bin/carddisp.pl?gene=RPL18 | 3.15605134 | 0.002157468 |
| *RPL19* | 60S ribosomal protein L19 | http://www.genecards.org/cgi-bin/carddisp.pl?gene=RPL19 | 1.542689517 | 0.015547642 |
| *RPL21* | 60S ribosomal protein L21 | http://www.genecards.org/cgi-bin/carddisp.pl?gene=RPL21 | 9.48009707 | 0.005456307 |
| *RPL22* | 60S ribosomal protein L22 | http://www.genecards.org/cgi-bin/carddisp.pl?gene=RPL22 | 1.394616468 | 0.009924346 |
| *RPL23* | 60S ribosomal protein L23 | http://www.genecards.org/cgi-bin/carddisp.pl?gene=RPL23 | 1.839284861 | 0.020183661 |
| *RPL29* | 60S ribosomal protein L29 | http://www.genecards.org/cgi-bin/carddisp.pl?gene=RPL29 | 3.681820427 | 0.000206338 |
| *RPL30* | 60S ribosomal protein L30 | http://www.genecards.org/cgi-bin/carddisp.pl?gene=RPL30 | 3.069031918 | 0.003884593 |
| *RPL37A* | 60S ribosomal protein L37a | http://www.genecards.org/cgi-bin/carddisp.pl?gene=RPL37A | 5.550966916 | 0.006020914 |
| *RPL38* | 60S ribosomal protein L38 | http://www.genecards.org/cgi-bin/carddisp.pl?gene=RPL38 | 5.9702873 | 0.007151675 |
| *RPL4* | 60S ribosomal protein L4 | http://www.genecards.org/cgi-bin/carddisp.pl?gene=RPL4 | 1.66721921 | 0.001295107 |
| *RPL5* | 60S ribosomal protein L5 | http://www.genecards.org/cgi-bin/carddisp.pl?gene=RPL5 | 11.6723245 | 5.73886E-06 |
| *RPL6* | 60S ribosomal protein L6 | http://www.genecards.org/cgi-bin/carddisp.pl?gene=RPL6 | 2.795723368 | 0.000970425 |
| *RPL7* | 60S ribosomal protein L7 | http://www.genecards.org/cgi-bin/carddisp.pl?gene=RPL7 | 2.632439742 | 0.012967361 |
| *RPL7A* | 60S ribosomal protein L7a | http://www.genecards.org/cgi-bin/carddisp.pl?gene=RPL7A | 1.652440449 | 0.005822064 |
| *RPL8* | 60S ribosomal protein L8 | http://www.genecards.org/cgi-bin/carddisp.pl?gene=RPL8 | 2.030809469 | 6.49289E-05 |
| *RPLP0* | 60S acidic ribosomal protein P0 | http://www.genecards.org/cgi-bin/carddisp.pl?gene=RPLP0 | 2.455463988 | 0.00246016 |
| *RPS10* | 40S ribosomal protein S10 | http://www.genecards.org/cgi-bin/carddisp.pl?gene=RPS10 | 3.010820792 | 0.000225712 |
| *RPS12* | 40S ribosomal protein S12 | http://www.genecards.org/cgi-bin/carddisp.pl?gene=RPS12 | 2.074594028 | 0.000994048 |
| *RPS13* | 40S ribosomal protein S13 | http://www.genecards.org/cgi-bin/carddisp.pl?gene=RPS13 | 4.521035845 | 0.000489167 |
| *RPS14* | 40S ribosomal protein S14 | http://www.genecards.org/cgi-bin/carddisp.pl?gene=RPS14 | 1.522544588 | 0.027247598 |
| *RPS16* | 40S ribosomal protein S16 | http://www.genecards.org/cgi-bin/carddisp.pl?gene=RPS16 | 8.094221539 | 0.002405712 |
| *RPS18* | 40S ribosomal protein S18 | http://www.genecards.org/cgi-bin/carddisp.pl?gene=RPS18 | 2.079659635 | 2.85705E-05 |
| *RPS19* | 40S ribosomal protein S19 | http://www.genecards.org/cgi-bin/carddisp.pl?gene=RPS19 | 1.518848241 | 0.009293668 |
| *RPS23* | 40S ribosomal protein S23 | http://www.genecards.org/cgi-bin/carddisp.pl?gene=RPS23 | only present in RPEp53-/- FA | 0.011062425 |
| *RPS27L* | 40S ribosomal protein S27-like | http://www.genecards.org/cgi-bin/carddisp.pl?gene=RPS27L | 2.093918786 | 0.008988858 |
| *RPS29* | 40S ribosomal protein S29 | http://www.genecards.org/cgi-bin/carddisp.pl?gene=RPS29 | 11.355501 | 0.008071091 |
| *RPS4X* | 40S ribosomal protein S4, X isoform | http://www.genecards.org/cgi-bin/carddisp.pl?gene=RPS4X | 5.092234228 | 0.048050556 |
| *RPS5* | 40S ribosomal protein S5 | http://www.genecards.org/cgi-bin/carddisp.pl?gene=RPS5 | 14.59468607 | 0.021493745 |
| *RPS6* | 40S ribosomal protein S6 | http://www.genecards.org/cgi-bin/carddisp.pl?gene=RPS6 | 1.546968953 | 0.008392994 |
| *RPS7* | 40S ribosomal protein S7 | http://www.genecards.org/cgi-bin/carddisp.pl?gene=RPS7 | 43.23376602 | 0.003626599 |
| *RPS9* | 40S ribosomal protein S9 | http://www.genecards.org/cgi-bin/carddisp.pl?gene=RPS9 | 2.856936603 | 6.35231E-05 |
| *RTN4* | Reticulon-4 | http://www.genecards.org/cgi-bin/carddisp.pl?gene=RTN4 | 2.53502453 | 0.000369899 |
| *SCAMP3* | Secretory carrier-associated membrane protein 3 | http://www.genecards.org/cgi-bin/carddisp.pl?gene=SCAMP3 | 4.655548411 | 0.007544265 |
| *SCAMP4* | Secretory carrier-associated membrane protein 4 | http://www.genecards.org/cgi-bin/carddisp.pl?gene=SCAMP4 | 4.122513715 | 0.000598172 |
| *SDC1* | Syndecan-1 | http://www.genecards.org/cgi-bin/carddisp.pl?gene=SDC1 | 2.250767758 | 0.006813102 |
| *SEC22B* | Vesicle-trafficking protein SEC22b | http://www.genecards.org/cgi-bin/carddisp.pl?gene=SEC22B | 4.065387641 | 3.35194E-05 |
| *SEMA7A* | Semaphorin-7A | http://www.genecards.org/cgi-bin/carddisp.pl?gene=SEMA7A | 3.202623239 | 0.000687792 |
| *SEP11* | Septin-11 | http://www.genecards.org/cgi-bin/carddisp.pl?gene=SEP11 | 2.101886987 | 0.00347163 |
| *SEPT2* | Septin-2 | http://www.genecards.org/cgi-bin/carddisp.pl?gene=SEPT2 | 2.655408476 | 0.005526081 |
| *SEPT8* | Septin-8 | http://www.genecards.org/cgi-bin/carddisp.pl?gene=SEPT8 | 2.420176285 | 0.002471624 |
| *SEPT9* | Septin-9 | http://www.genecards.org/cgi-bin/carddisp.pl?gene=SEPT9 | 2.196014063 | 0.000156817 |
| *SERPIND1* | Heparin cofactor 2 | http://www.genecards.org/cgi-bin/carddisp.pl?gene=SERPIND1 | 1.23548342 | 0.034283558 |
| *SERPINE1* | Plasminogen activator inhibitor 1 | http://www.genecards.org/cgi-bin/carddisp.pl?gene=SERPINE1 | 1.721632763 | 0.001808691 |
| *SKP1* | S-phase kinase-associated protein 1 | http://www.genecards.org/cgi-bin/carddisp.pl?gene=SKP1 | 2.535506863 | 0.000550702 |
| *SLC16A1* | Monocarboxylate transporter 1 | http://www.genecards.org/cgi-bin/carddisp.pl?gene=SLC16A1 | 2.460010127 | 1.9613E-05 |
| *SLC25A5* | ADP/ATP translocase 2 | http://www.genecards.org/cgi-bin/carddisp.pl?gene=SLC25A5 | 2.088594703 | 0.006963431 |
| *SLC2A1* | Solute carrier family 2, facilitated glucose transporter member 1 | http://www.genecards.org/cgi-bin/carddisp.pl?gene=SLC2A1 | 2.429981797 | 9.71624E-05 |
| *SLC39A12* | Zinc transporter ZIP12 | http://www.genecards.org/cgi-bin/carddisp.pl?gene=SLC39A12 | 2.290260838 | 0.019015803 |
| *SLC39A14* | Zinc transporter ZIP14 | http://www.genecards.org/cgi-bin/carddisp.pl?gene=SLC39A14 | 2.416620792 | 0.000154401 |
| *SLC3A2* | 4F2 cell-surface antigen heavy chain | http://www.genecards.org/cgi-bin/carddisp.pl?gene=SLC3A2 | 2.736883729 | 0.002691976 |
| *SLC44A1* | Choline transporter-like protein 1 | http://www.genecards.org/cgi-bin/carddisp.pl?gene=SLC44A1 | 9.645852924 | 0.00112506 |
| *SLC4A7* | Sodium bicarbonate cotransporter 3 | http://www.genecards.org/cgi-bin/carddisp.pl?gene=SLC4A7 | 2.019004309 | 0.023959468 |
| *SLC7A1* | High affinity cationic amino acid transporter 1 | http://www.genecards.org/cgi-bin/carddisp.pl?gene=SLC7A1 | 105.3247043 | 0.006700935 |
| *SLC9A3R1* | Na(+)/H(+) exchange regulatory cofactor NHE-RF1 | http://www.genecards.org/cgi-bin/carddisp.pl?gene=SLC9A3R1 | 1.631799299 | 0.005113656 |
| *SLFN14* | Protein SLFN14 | http://www.genecards.org/cgi-bin/carddisp.pl?gene=SLFN14 | 365.2457977 | 0.000340157 |
| *SMARCA2* | Probable global transcription activator SNF2L2 | http://www.genecards.org/cgi-bin/carddisp.pl?gene=SMARCA2 | 1.387760398 | 0.015490752 |
| *SNAP23* | Synaptosomal-associated protein 23 | http://www.genecards.org/cgi-bin/carddisp.pl?gene=SNAP23 | 2.389874226 | 0.000185959 |
| *SNRPE* | Small nuclear ribonucleoprotein E | http://www.genecards.org/cgi-bin/carddisp.pl?gene=SNRPE | 14.65784736 | 0.032801006 |
| *SNRPF* | Small nuclear ribonucleoprotein F | http://www.genecards.org/cgi-bin/carddisp.pl?gene=SNRPF | 8.382205592 | 0.000664376 |
| *SORBS3* | Vinexin | http://www.genecards.org/cgi-bin/carddisp.pl?gene=SORBS3 | 1.759769501 | 0.014400807 |
| *SORT1* | Sortilin | http://www.genecards.org/cgi-bin/carddisp.pl?gene=SORT1 | 5.156684662 | 0.001225554 |
| *SPTBN1* | Spectrin beta chain, non-erythrocytic 1 | http://www.genecards.org/cgi-bin/carddisp.pl?gene=SPTBN1 | 1.362481514 | 0.019285641 |
| *SSC4D* | Scavenger receptor cysteine-rich domain-containing group B protein | http://www.genecards.org/cgi-bin/carddisp.pl?gene=SSC4D | 8.703573131 | 0.001815673 |
| *STEAP3* | Metalloreductase STEAP3 | http://www.genecards.org/cgi-bin/carddisp.pl?gene=STEAP3 | 7.798856272 | 4.25591E-07 |
| *STX4* | Syntaxin-4 | http://www.genecards.org/cgi-bin/carddisp.pl?gene=STX4 | 2.130081913 | 0.001549444 |
| *STX7* | Syntaxin-7 | http://www.genecards.org/cgi-bin/carddisp.pl?gene=STX7 | 1.886314029 | 0.000955858 |
| *STXBP1* | Syntaxin-binding protein 1 | http://www.genecards.org/cgi-bin/carddisp.pl?gene=STXBP1 | 4.858301555 | 5.04342E-05 |
| *SYNJ2* | Synaptojanin-2 | http://www.genecards.org/cgi-bin/carddisp.pl?gene=SYNJ2 | 3.946113686 | 0.028596198 |
| *SYNM* | Synemin | http://www.genecards.org/cgi-bin/carddisp.pl?gene=SYNM | 3.256934674 | 0.021141032 |
| *TAF7* | Transcription initiation factor TFIID subunit 7 | http://www.genecards.org/cgi-bin/carddisp.pl?gene=TAF7 | only present in RPEp53-/- FA | 0.035696755 |
| *TAGLN2* | Transgelin-2 | http://www.genecards.org/cgi-bin/carddisp.pl?gene=TAGLN2 | 2.738372564 | 0.011668059 |
| *TAGLN3* | Transgelin-3 | http://www.genecards.org/cgi-bin/carddisp.pl?gene=TAGLN3 | 11.4556716 | 0.020475516 |
| *TFRC* | Transferrin receptor protein 1 | http://www.genecards.org/cgi-bin/carddisp.pl?gene=TFRC | 2.691334539 | 0.000294757 |
| *TGFB1I1* | Transforming growth factor beta-1-induced transcript 1 protein | http://www.genecards.org/cgi-bin/carddisp.pl?gene=TGFB1I1 | 2.984560683 | 0.001281093 |
| *THY1* | Thy-1 membrane glycoprotein | http://www.genecards.org/cgi-bin/carddisp.pl?gene=THY1 | 4.122287248 | 0.004060897 |
| *TIMM8A* | Mitochondrial import inner membrane translocase subunit Tim8 A | http://www.genecards.org/cgi-bin/carddisp.pl?gene=TIMM8A | 7.77234802 | 0.010965335 |
| *TLN1* | Talin-1 | http://www.genecards.org/cgi-bin/carddisp.pl?gene=TLN1 | 1.380857075 | 0.038708979 |
| *TLN2* | Talin-2 | http://www.genecards.org/cgi-bin/carddisp.pl?gene=TLN2 | 2.284901621 | 0.024651118 |
| *TMEM123* | Porimin | http://www.genecards.org/cgi-bin/carddisp.pl?gene=TMEM123 | 3.952565292 | 0.01299108 |
| *TMEM33* | Transmembrane protein 33 | http://www.genecards.org/cgi-bin/carddisp.pl?gene=TMEM33 | 6.917570745 | 0.001271173 |
| *TMOD3* | Tropomodulin-3 | http://www.genecards.org/cgi-bin/carddisp.pl?gene=TMOD3 | 1.390040205 | 0.042029679 |
| *TNS1* | Tensin-1 | http://www.genecards.org/cgi-bin/carddisp.pl?gene=TNS1 | 2.194780219 | 0.003541308 |
| *TNS2* | Tensin-2 | http://www.genecards.org/cgi-bin/carddisp.pl?gene=TNS2 | 1.610817328 | 0.042803042 |
| *TNS3* | Tensin-3 | http://www.genecards.org/cgi-bin/carddisp.pl?gene=TNS3 | 2.112473502 | 2.65493E-05 |
| *TPBG* | Trophoblast glycoprotein | http://www.genecards.org/cgi-bin/carddisp.pl?gene=TPBG | 8.459279729 | 0.000167405 |
| *TPM1* | Tropomyosin alpha-1 chain | http://www.genecards.org/cgi-bin/carddisp.pl?gene=TPM1 | 4.992190556 | 1.1325E-05 |
| *TPM3* | Tropomyosin alpha-3 chain | http://www.genecards.org/cgi-bin/carddisp.pl?gene=TPM3 | 1.833750233 | 0.000710176 |
| *TPM4* | Tropomyosin alpha-4 chain | http://www.genecards.org/cgi-bin/carddisp.pl?gene=TPM4 | 1.966351049 | 0.000681003 |
| *TRIM72* | Tripartite motif-containing protein 72 | http://www.genecards.org/cgi-bin/carddisp.pl?gene=TRIM72 | 6.764227541 | 0.002721394 |
| *TRIO* | Triple functional domain protein | http://www.genecards.org/cgi-bin/carddisp.pl?gene=TRIO | 1.931810306 | 0.007257637 |
| *TRPM2* | Transient receptor potential cation channel subfamily M member 2 | http://www.genecards.org/cgi-bin/carddisp.pl?gene=TRPM2 | 4.32881599 | 0.045847474 |
| *TSPAN14* | Tetraspanin-14 | http://www.genecards.org/cgi-bin/carddisp.pl?gene=TSPAN14 | 2.23263773 | 0.000122204 |
| *TTC17* | Tetratricopeptide repeat protein 17 | http://www.genecards.org/cgi-bin/carddisp.pl?gene=TTC17 | 10.180861 | 0.000653818 |
| *TTC3* | E3 ubiquitin-protein ligase TTC3 | http://www.genecards.org/cgi-bin/carddisp.pl?gene=TTC3 | 12.02714689 | 0.013293489 |
| *TUBAL3* | Tubulin alpha chain-like 3 | http://www.genecards.org/cgi-bin/carddisp.pl?gene=TUBAL3 | 1.771254452 | 0.022212198 |
| *TUBB1* | Tubulin beta-1 chain | http://www.genecards.org/cgi-bin/carddisp.pl?gene=TUBB1 | 10.22294517 | 0.007919936 |
| *TXNDC5* | Thioredoxin domain-containing protein 5 | http://www.genecards.org/cgi-bin/carddisp.pl?gene=TXNDC5 | only present in RPEp53-/- FA | 0.027054675 |
| *UBTD2* | Ubiquitin domain-containing protein 2 | http://www.genecards.org/cgi-bin/carddisp.pl?gene=UBTD2 | 17.20096795 | 0.00193495 |
| *UCHL1* | Ubiquitin carboxyl-terminal hydrolase isozyme L1 | http://www.genecards.org/cgi-bin/carddisp.pl?gene=UCHL1 | 4.016494429 | 0.005851158 |
| *USH2A* | Usherin | http://www.genecards.org/cgi-bin/carddisp.pl?gene=USH2A | 2.05718607 | 0.018128883 |
| *VASN* | Vasorin | http://www.genecards.org/cgi-bin/carddisp.pl?gene=VASN | 7.553508932 | 0.000339177 |
| *VASP* | Vasodilator-stimulated phosphoprotein | http://www.genecards.org/cgi-bin/carddisp.pl?gene=VASP | 1.757865103 | 0.014789834 |
| *VCL* | Vinculin | http://www.genecards.org/cgi-bin/carddisp.pl?gene=VCL | 1.539375412 | 0.000910655 |
| *VIM* | Vimentin | http://www.genecards.org/cgi-bin/carddisp.pl?gene=VIM | 1.71477959 | 0.010307244 |
| *VIPR1* | Vasoactive intestinal polypeptide receptor 1 | http://www.genecards.org/cgi-bin/carddisp.pl?gene=VIPR1 | only present in RPEp53-/- FA | 0.001820361 |
| *WDR1* | WD repeat-containing protein 1 | http://www.genecards.org/cgi-bin/carddisp.pl?gene=WDR1 | 1.789043402 | 0.024818599 |
| *WNT7A* | Protein Wnt-7a | http://www.genecards.org/cgi-bin/carddisp.pl?gene=WNT7A | 60.95365479 | 0.004170321 |
| *XAF1* | XIAP-associated factor 1 | http://www.genecards.org/cgi-bin/carddisp.pl?gene=XAF1 | 10.16745767 | 0.004701161 |
| *YBX1* | Nuclease-sensitive element-binding protein 1 | http://www.genecards.org/cgi-bin/carddisp.pl?gene=YBX1 | 3.124757604 | 0.000687518 |
| *YWHAB* | 14-3-3 protein beta/alpha | http://www.genecards.org/cgi-bin/carddisp.pl?gene=YWHAB | 1.402237739 | 0.001591598 |
| *YWHAE* | 14-3-3 protein epsilon | http://www.genecards.org/cgi-bin/carddisp.pl?gene=YWHAE | 3.034104795 | 0.014669022 |
| *YWHAQ* | 14-3-3 protein theta | http://www.genecards.org/cgi-bin/carddisp.pl?gene=YWHAQ | 1.848715334 | 0.032951746 |
| *ZC2HC1B* | Zinc finger C2HC domain-containing protein 1B | http://www.genecards.org/cgi-bin/carddisp.pl?gene=ZC2HC1B | 5.050418991 | 0.016800254 |
| *ZNF562* | Zinc finger protein 562 | http://www.genecards.org/cgi-bin/carddisp.pl?gene=ZNF562 | 3.089863396 | 0.040200542 |
| *ZYX* | Zyxin | http://www.genecards.org/cgi-bin/carddisp.pl?gene=ZYX | 2.064102696 | 0.007614368 |

**Increased abundance in FA fraction isolated from RPEp53^-/-^STIL^-/-^ cells**

| **Gene name** | **Description** | [**Gene information**](https://l.facebook.com/l.php?u=http%3A%2F%2Fwww.genecards.org%2Fcgi-bin%2Fcarddisp.pl%3Fgene%3D%2522%252CA1&h=ATP6yswR14bvN0ocja1Ey1G_azEX6hPvaoaG6wnlhO80hCNibRxOBC7mpa7DI2EWkCMhK9LIGIVnWVtcmb2ldic7GGL5x8e6YusaY-ljmcwOhdMPSq3PhaYfIKBlTYELWIWn) | **Centrosome Dependence Ratio** | **p-value**  **(Student's t-test)** |
| --- | --- | --- | --- | --- |
| *A2M* | Alpha-2-macroglobulin | http://www.genecards.org/cgi-bin/carddisp.pl?gene=A2M | 0.669354483 | 0.006169066 |
| *ABCA13* | ATP-binding cassette sub-family A member 13 | http://www.genecards.org/cgi-bin/carddisp.pl?gene=ABCA13 | 0.374938102 | 0.006287998 |
| *ABCB4* | Phosphatidylcholine translocator ABCB4 | http://www.genecards.org/cgi-bin/carddisp.pl?gene=ABCB4 | 0.152771969 | 0.02882878 |
| *ADAM19* | Disintegrin and metalloproteinase domain-containing protein 19 | http://www.genecards.org/cgi-bin/carddisp.pl?gene=ADAM19 | 0.047089588 | 0.015019215 |
| *ADAMTS1* | A disintegrin and metalloproteinase with thrombospondin motifs 1 | http://www.genecards.org/cgi-bin/carddisp.pl?gene=ADAMTS1 | 0.129375733 | 2.50264E-05 |
| *ADAMTSL1* | ADAMTS-like protein 1 | http://www.genecards.org/cgi-bin/carddisp.pl?gene=ADAMTSL1 | 0.805826679 | 0.003227055 |
| *ADGRL1* | Adhesion G protein-coupled receptor L1 | http://www.genecards.org/cgi-bin/carddisp.pl?gene=ADGRL1 | 0.194419679 | 0.002634084 |
| *ADM* | ADM | http://www.genecards.org/cgi-bin/carddisp.pl?gene=ADM | 0.201969258 | 0.007734353 |
| *AFMID* | Kynurenine formamidase | http://www.genecards.org/cgi-bin/carddisp.pl?gene=AFMID | 0.400737773 | 0.046148086 |
| *AGBL5* | Cytosolic carboxypeptidase-like protein 5 | http://www.genecards.org/cgi-bin/carddisp.pl?gene=AGBL5 | 0.515946097 | 0.018849832 |
| *AGFG1* | Arf-GAP domain and FG repeat-containing protein 1 | http://www.genecards.org/cgi-bin/carddisp.pl?gene=AGFG1 | 0.48511992 | 0.007444082 |
| *AHSG* | Alpha-2-HS-glycoprotein | http://www.genecards.org/cgi-bin/carddisp.pl?gene=AHSG | 0.355521562 | 0.000567091 |
| *AKAP17A* | A-kinase anchor protein 17A | http://www.genecards.org/cgi-bin/carddisp.pl?gene=AKAP17A | 0.007614519 | 0.046654605 |
| *ALB* | Serum albumin | http://www.genecards.org/cgi-bin/carddisp.pl?gene=ALB | 0.47573582 | 1.28551E-05 |
| *ALDOA* | Fructose-bisphosphate aldolase A | http://www.genecards.org/cgi-bin/carddisp.pl?gene=ALDOA | 0.768596839 | 0.010963149 |
| *AMMECR1L* | AMMECR1-like protein | http://www.genecards.org/cgi-bin/carddisp.pl?gene=AMMECR1L | 0.025774843 | 0.00159542 |
| *ANGPT1* | Angiopoietin-1 | http://www.genecards.org/cgi-bin/carddisp.pl?gene=ANGPT1 | 0.287893192 | 9.0177E-05 |
| *ANKRD18A* | Ankyrin repeat domain-containing protein 18A | http://www.genecards.org/cgi-bin/carddisp.pl?gene=ANKRD18A | 0.006409662 | 0.009018229 |
| *ANOS1* | Anosmin-1 | http://www.genecards.org/cgi-bin/carddisp.pl?gene=ANOS1 | 0.039290279 | 0.000476708 |
| *ANXA1* | Annexin A1 | http://www.genecards.org/cgi-bin/carddisp.pl?gene=ANXA1 | 0.573233911 | 0.002180537 |
| *AP5M1* | AP-5 complex subunit mu-1 | http://www.genecards.org/cgi-bin/carddisp.pl?gene=AP5M1 | 0.334236014 | 0.009124933 |
| *APC2* | Adenomatous polyposis coli protein 2 | http://www.genecards.org/cgi-bin/carddisp.pl?gene=APC2 | 0.516519265 | 0.006791926 |
| *APOC3* | Apolipoprotein C-III | http://www.genecards.org/cgi-bin/carddisp.pl?gene=APOC3 | 0.432581444 | 0.000173576 |
| *APOE* | Apolipoprotein E | http://www.genecards.org/cgi-bin/carddisp.pl?gene=APOE | 0.645999359 | 0.034877961 |
| *APOH* | Beta-2-glycoprotein 1 | http://www.genecards.org/cgi-bin/carddisp.pl?gene=APOH | 0.36365124 | 0.001207148 |
| *ARG1* | Arginase-1 | http://www.genecards.org/cgi-bin/carddisp.pl?gene=ARG1 | 0.051298673 | 0.017977119 |
| *ARHGDIA* | Rho GDP-dissociation inhibitor 1 | http://www.genecards.org/cgi-bin/carddisp.pl?gene=ARHGDIA | 0.592561492 | 0.019393211 |
| *ATIC* | Bifunctional purine biosynthesis protein PURH | http://www.genecards.org/cgi-bin/carddisp.pl?gene=ATIC | 0.385867389 | 0.019191215 |
| *ATP1A2* | Sodium/potassium-transporting ATPase subunit alpha-2 | http://www.genecards.org/cgi-bin/carddisp.pl?gene=ATP1A2 | 0.613336996 | 0.014161944 |
| *ATP2B2* | Plasma membrane calcium-transporting ATPase 2 | http://www.genecards.org/cgi-bin/carddisp.pl?gene=ATP2B2 | 0.307916461 | 0.007227809 |
| *ATP2B4* | Plasma membrane calcium-transporting ATPase 4 | http://www.genecards.org/cgi-bin/carddisp.pl?gene=ATP2B4 | 0.731842808 | 0.043634643 |
| *ATP6V1D* | V-type proton ATPase subunit D | http://www.genecards.org/cgi-bin/carddisp.pl?gene=ATP6V1D | 0.45895882 | 0.016121841 |
| *AZGP1* | Zinc-alpha-2-glycoprotein | http://www.genecards.org/cgi-bin/carddisp.pl?gene=AZGP1 | 0.282916146 | 0.010627932 |
| *BAIAP2* | Brain-specific angiogenesis inhibitor 1-associated protein 2 | http://www.genecards.org/cgi-bin/carddisp.pl?gene=BAIAP2 | 0.097161571 | 0.006853066 |
| *BHMG1* | Basic helix-loop-helix and HMG box domain-containing protein 1 | http://www.genecards.org/cgi-bin/carddisp.pl?gene=BHMG1 | 0.432514245 | 0.027925789 |
| *BTBD10* | BTB/POZ domain-containing protein 10 | http://www.genecards.org/cgi-bin/carddisp.pl?gene=BTBD10 | 0.045050331 | 0.00055786 |
| *C1QTNF3* | Complement C1q tumor necrosis factor-related protein 3 | http://www.genecards.org/cgi-bin/carddisp.pl?gene=C1QTNF3 | 0.41917958 | 0.024272467 |
| *C2orf42* | Uncharacterized protein C2orf42 | http://www.genecards.org/cgi-bin/carddisp.pl?gene=C2orf42 | 0.28181775 | 0.000222032 |
| *C3* | Complement C3 | http://www.genecards.org/cgi-bin/carddisp.pl?gene=C3 | 0.603077466 | 0.040220162 |
| *C4A* | Complement C4-A | http://www.genecards.org/cgi-bin/carddisp.pl?gene=C4A | 0.314297368 | 0.013885266 |
| *C6orf10* | Uncharacterized protein C6orf10 | http://www.genecards.org/cgi-bin/carddisp.pl?gene=C6orf10 | 0.021658879 | 0.03698109 |
| *CAMK1D* | Calcium/calmodulin-dependent protein kinase type 1D | http://www.genecards.org/cgi-bin/carddisp.pl?gene=CAMK1D | 0.153847115 | 0.030327666 |
| *CAPN1* | Calpain-1 catalytic subunit | http://www.genecards.org/cgi-bin/carddisp.pl?gene=CAPN1 | 0.771906335 | 0.025871182 |
| *CAPNS1* | Calpain small subunit 1 | http://www.genecards.org/cgi-bin/carddisp.pl?gene=CAPNS1 | 0.639829592 | 0.000105872 |
| *CAPZA1* | F-actin-capping protein subunit alpha-1 | http://www.genecards.org/cgi-bin/carddisp.pl?gene=CAPZA1 | 0.115998417 | 1.02998E-06 |
| *CASP14* | Caspase-14 | http://www.genecards.org/cgi-bin/carddisp.pl?gene=CASP14 | 0.212548726 | 0.008612789 |
| *CAT* | Catalase | http://www.genecards.org/cgi-bin/carddisp.pl?gene=CAT | 0.055719014 | 0.038552101 |
| *CCDC124* | Coiled-coil domain-containing protein 124 | http://www.genecards.org/cgi-bin/carddisp.pl?gene=CCDC124 | 0.61602309 | 0.003747491 |
| *CCDC14* | Coiled-coil domain-containing protein 14 | http://www.genecards.org/cgi-bin/carddisp.pl?gene=CCDC14 | 0.392655629 | 0.011229117 |
| *CCDC47* | Coiled-coil domain-containing protein 47 | http://www.genecards.org/cgi-bin/carddisp.pl?gene=CCDC47 | 0.330390494 | 0.004915938 |
| *CD247* | T-cell surface glycoprotein CD3 zeta chain | http://www.genecards.org/cgi-bin/carddisp.pl?gene=CD247 | 0.271559118 | 0.024341696 |
| *CD55* | Complement decay-accelerating factor | http://www.genecards.org/cgi-bin/carddisp.pl?gene=CD55 | 0.798324811 | 0.022296168 |
| *CD82* | CD82 antigen | http://www.genecards.org/cgi-bin/carddisp.pl?gene=CD82 | 0.349741239 | 0.004348294 |
| *CD9* | CD9 antigen | http://www.genecards.org/cgi-bin/carddisp.pl?gene=CD9 | 0.737134062 | 0.040201305 |
| *CEP131* | Centrosomal protein of 131 kDa | http://www.genecards.org/cgi-bin/carddisp.pl?gene=CEP131 | 0.399697465 | 0.012709998 |
| *CEP295NL* | CEP295 N-terminal-like protein | http://www.genecards.org/cgi-bin/carddisp.pl?gene=CEP295NL | 0.418199183 | 0.041794667 |
| *CFAP43* | Cilia- and flagella-associated protein 43 | http://www.genecards.org/cgi-bin/carddisp.pl?gene=CFAP43 | 0.00973246 | 0.018717257 |
| *CFH* | Complement factor H | http://www.genecards.org/cgi-bin/carddisp.pl?gene=CFH | 0.342570019 | 7.43564E-05 |
| *CHMP4B* | Charged multivesicular body protein 4b | http://www.genecards.org/cgi-bin/carddisp.pl?gene=CHMP4B | 0.595683315 | 0.006571339 |
| *CHRDL1* | Chordin-like protein 1 | http://www.genecards.org/cgi-bin/carddisp.pl?gene=CHRDL1 | 0.436571097 | 0.001403584 |
| *CHST2* | Carbohydrate sulfotransferase 2 | http://www.genecards.org/cgi-bin/carddisp.pl?gene=CHST2 | 0.19044386 | 0.00277362 |
| *CIRBP* | Cold-inducible RNA-binding protein | http://www.genecards.org/cgi-bin/carddisp.pl?gene=CIRBP | 0.357330322 | 0.000405452 |
| *CLEC3B* | Tetranectin | http://www.genecards.org/cgi-bin/carddisp.pl?gene=CLEC3B | 0.225211567 | 3.63377E-05 |
| *CLIC1* | Chloride intracellular channel protein 1 | http://www.genecards.org/cgi-bin/carddisp.pl?gene=CLIC1 | 0.549965378 | 0.016152102 |
| *CLTA* | Clathrin light chain A | http://www.genecards.org/cgi-bin/carddisp.pl?gene=CLTA | 0.760518309 | 0.045390408 |
| *CLTB* | Clathrin light chain B | http://www.genecards.org/cgi-bin/carddisp.pl?gene=CLTB | 0.603405189 | 0.002321351 |
| *CMYA5* | Cardiomyopathy-associated protein 5 | http://www.genecards.org/cgi-bin/carddisp.pl?gene=CMYA5 | 0.417225846 | 0.000621331 |
| *CNGB1* | Cyclic nucleotide-gated cation channel beta-1 | http://www.genecards.org/cgi-bin/carddisp.pl?gene=CNGB1 | 0.070150912 | 0.007569925 |
| *COL6A1* | Collagen alpha-1(VI) chain | http://www.genecards.org/cgi-bin/carddisp.pl?gene=COL6A1 | 0.339987913 | 0.030021514 |
| *COL7A1* | Collagen alpha-1(VII) chain | http://www.genecards.org/cgi-bin/carddisp.pl?gene=COL7A1 | 0.550968044 | 0.002651337 |
| *CPLX1* | Complexin-1 | http://www.genecards.org/cgi-bin/carddisp.pl?gene=CPLX1 | 0.622703917 | 0.002867576 |
| *CREB5* | Cyclic AMP-responsive element-binding protein 5 | http://www.genecards.org/cgi-bin/carddisp.pl?gene=CREB5 | 0.443005537 | 0.011602172 |
| *CRYBG3* | Very large A-kinase anchor protein | http://www.genecards.org/cgi-bin/carddisp.pl?gene=CRYBG3 | 0.605933476 | 0.009716337 |
| *CSRP1* | Cysteine and glycine-rich protein 1 | http://www.genecards.org/cgi-bin/carddisp.pl?gene=CSRP1 | 0.717623035 | 0.027996299 |
| *CSTA* | Cystatin-A | http://www.genecards.org/cgi-bin/carddisp.pl?gene=CSTA | 0.064571042 | 0.009203988 |
| *CTGF* | Connective tissue growth factor | http://www.genecards.org/cgi-bin/carddisp.pl?gene=CTGF | 0.184092626 | 0.001217282 |
| *DCD* | Dermcidin | http://www.genecards.org/cgi-bin/carddisp.pl?gene=DCD | 0.163598249 | 0.004982064 |
| *DDX46* | Probable ATP-dependent RNA helicase DDX46 | http://www.genecards.org/cgi-bin/carddisp.pl?gene=DDX46 | 0.399046144 | 0.012960168 |
| *DHX33* | Putative ATP-dependent RNA helicase DHX33 | http://www.genecards.org/cgi-bin/carddisp.pl?gene=DHX33 | 0.189504163 | 0.026918767 |
| *DKK1* | Dickkopf-related protein 1 | http://www.genecards.org/cgi-bin/carddisp.pl?gene=DKK1 | 0.609972379 | 0.000566168 |
| *DLC1* | Rho GTPase-activating protein 7 | http://www.genecards.org/cgi-bin/carddisp.pl?gene=DLC1 | 0.084792391 | 0.002938277 |
| *DNAJA2* | DnaJ homolog subfamily A member 2 | http://www.genecards.org/cgi-bin/carddisp.pl?gene=DNAJA2 | 0.307394652 | 0.019870099 |
| *DNER* | Delta and Notch-like epidermal growth factor-related receptor | http://www.genecards.org/cgi-bin/carddisp.pl?gene=DNER | 0.416635649 | 0.007316808 |
| *DNM2* | Dynamin-2 | http://www.genecards.org/cgi-bin/carddisp.pl?gene=DNM2 | 0.077809535 | 0.019431209 |
| *DNMT1* | DNA (cytosine-5)-methyltransferase 1 | http://www.genecards.org/cgi-bin/carddisp.pl?gene=DNMT1 | 0.15350788 | 3.67754E-05 |
| *DOCK7* | Dedicator of cytokinesis protein 7 | http://www.genecards.org/cgi-bin/carddisp.pl?gene=DOCK7 | 0.127704025 | 0.002022434 |
| *DRG1* | Developmentally-regulated GTP-binding protein 1 | http://www.genecards.org/cgi-bin/carddisp.pl?gene=DRG1 | 0.732436403 | 0.030579291 |
| *DSC2* | Desmocollin-2 | http://www.genecards.org/cgi-bin/carddisp.pl?gene=DSC2 | 0.498615539 | 0.010808607 |
| *DSC3* | Desmocollin-3 | http://www.genecards.org/cgi-bin/carddisp.pl?gene=DSC3 | 0.408118666 | 0.000286329 |
| *DSG1* | Desmoglein-1 | http://www.genecards.org/cgi-bin/carddisp.pl?gene=DSG1 | 0.527785545 | 0.025608671 |
| *EFHD1* | EF-hand domain-containing protein D1 | http://www.genecards.org/cgi-bin/carddisp.pl?gene=EFHD1 | 0.122345164 | 0.002621248 |
| *EIF4H* | Eukaryotic translation initiation factor 4H | http://www.genecards.org/cgi-bin/carddisp.pl?gene=EIF4H | 0.426480616 | 0.022806407 |
| *ELL2* | RNA polymerase II elongation factor ELL2 | http://www.genecards.org/cgi-bin/carddisp.pl?gene=ELL2 | 0.361574573 | 0.03242951 |
| *EML6* | Echinoderm microtubule-associated protein-like 6 | http://www.genecards.org/cgi-bin/carddisp.pl?gene=EML6 | 0.115388896 | 0.002636407 |
| *EPB41* | Protein 4.1 | http://www.genecards.org/cgi-bin/carddisp.pl?gene=EPB41 | 0.233205912 | 0.000833328 |
| *EPB41L3* | Band 4.1-like protein 3 | http://www.genecards.org/cgi-bin/carddisp.pl?gene=EPB41L3 | 0.500769894 | 7.59149E-05 |
| *EPHA4* | Ephrin type-A receptor 4 | http://www.genecards.org/cgi-bin/carddisp.pl?gene=EPHA4 | 0.193155232 | 0.005983773 |
| *EPHA6* | Ephrin type-A receptor 6 | http://www.genecards.org/cgi-bin/carddisp.pl?gene=EPHA6 | 0.079467233 | 0.029928336 |
| *EPS15L1* | Epidermal growth factor receptor substrate 15-like 1 | http://www.genecards.org/cgi-bin/carddisp.pl?gene=EPS15L1 | 0.47458537 | 0.002703713 |
| *EPS8* | Epidermal growth factor receptor kinase substrate 8 | http://www.genecards.org/cgi-bin/carddisp.pl?gene=EPS8 | 0.40580208 | 0.028447863 |
| *ESCO1* | N-acetyltransferase ESCO1 | http://www.genecards.org/cgi-bin/carddisp.pl?gene=ESCO1 | 0.292452414 | 0.026299599 |
| *ESM1* | Endothelial cell-specific molecule 1 | http://www.genecards.org/cgi-bin/carddisp.pl?gene=ESM1 | 0.308234126 | 0.011455929 |
| *EXOC8* | Exocyst complex component 8 | http://www.genecards.org/cgi-bin/carddisp.pl?gene=EXOC8 | 0.072711489 | 0.001419484 |
| *F2* | Prothrombin | http://www.genecards.org/cgi-bin/carddisp.pl?gene=F2 | 0.34103894 | 0.00089925 |
| *F5* | Coagulation factor V | http://www.genecards.org/cgi-bin/carddisp.pl?gene=F5 | 0.465385328 | 0.004369762 |
| *FAM117A* | Protein FAM117A | http://www.genecards.org/cgi-bin/carddisp.pl?gene=FAM117A | 0.418495948 | 0.009375776 |
| *FBN1* | Fibrillin-1 | http://www.genecards.org/cgi-bin/carddisp.pl?gene=FBN1 | 0.348299112 | 0.001235782 |
| *FCHO1* | F-BAR domain only protein 1 | http://www.genecards.org/cgi-bin/carddisp.pl?gene=FCHO1 | 0.025206107 | 0.000220507 |
| *FERMT3* | Fermitin family homolog 3 | http://www.genecards.org/cgi-bin/carddisp.pl?gene=FERMT3 | 0.349238299 | 0.000216485 |
| *FGD4* | FYVE, RhoGEF and PH domain-containing protein 4 | http://www.genecards.org/cgi-bin/carddisp.pl?gene=FGD4 | 0.085915983 | 0.014389269 |
| *FGFRL1* | Fibroblast growth factor receptor-like 1 | http://www.genecards.org/cgi-bin/carddisp.pl?gene=FGFRL1 | 0.129428606 | 0.044598222 |
| *FGG* | Fibrinogen gamma chain | http://www.genecards.org/cgi-bin/carddisp.pl?gene=FGG | 0.217642642 | 0.004767848 |
| *FN1* | Fibronectin | http://www.genecards.org/cgi-bin/carddisp.pl?gene=FN1 | 0.611783436 | 0.000547449 |
| *FNDC1* | Fibronectin type III domain-containing protein 1 | http://www.genecards.org/cgi-bin/carddisp.pl?gene=FNDC1 | 0.050601424 | 0.009053031 |
| *G6PD* | Glucose-6-phosphate 1-dehydrogenase | http://www.genecards.org/cgi-bin/carddisp.pl?gene=G6PD | 0.004839382 | 0.035576846 |
| *GC* | Vitamin D-binding protein | http://www.genecards.org/cgi-bin/carddisp.pl?gene=GC | 0.313928841 | 0.000491116 |
| *GDF6* | Growth/differentiation factor 6 | http://www.genecards.org/cgi-bin/carddisp.pl?gene=GDF6 | 0.325665345 | 1.07666E-07 |
| *GLIPR2* | Golgi-associated plant pathogenesis-related protein 1 | http://www.genecards.org/cgi-bin/carddisp.pl?gene=GLIPR2 | 0.684122573 | 0.004365912 |
| *GNA12* | Guanine nucleotide-binding protein subunit alpha-12 | http://www.genecards.org/cgi-bin/carddisp.pl?gene=GNA12 | 0.201024566 | 0.036159703 |
| *GNAO1* | Guanine nucleotide-binding protein G(o) subunit alpha | http://www.genecards.org/cgi-bin/carddisp.pl?gene=GNAO1 | 0.448577314 | 0.006005388 |
| *GNLY* | Granulysin | http://www.genecards.org/cgi-bin/carddisp.pl?gene=GNLY | 0.179891256 | 0.03350802 |
| *GPRIN1* | G protein-regulated inducer of neurite outgrowth 1 | http://www.genecards.org/cgi-bin/carddisp.pl?gene=GPRIN1 | 0.061827025 | 0.000402731 |
| *GREM1* | Gremlin-1 | http://www.genecards.org/cgi-bin/carddisp.pl?gene=GREM1 | 0.353885661 | 0.000135239 |
| *GSN* | Gelsolin | http://www.genecards.org/cgi-bin/carddisp.pl?gene=GSN | 0.281553077 | 0.00010601 |
| *GTF2IRD1* | General transcription factor II-I repeat domain-containing protein 1 | http://www.genecards.org/cgi-bin/carddisp.pl?gene=GTF2IRD1 | 0.190505819 | 0.038685759 |
| *GTF3C1* | General transcription factor 3C polypeptide 1 | http://www.genecards.org/cgi-bin/carddisp.pl?gene=GTF3C1 | 0.25592409 | 0.040162805 |
| *H1FX* | Histone H1x | http://www.genecards.org/cgi-bin/carddisp.pl?gene=H1FX | 0.048432012 | 0.022060961 |
| *HABP2* | Hyaluronan-binding protein 2 | http://www.genecards.org/cgi-bin/carddisp.pl?gene=HABP2 | 0.539489838 | 0.00023383 |
| *HAL* | Histidine ammonia-lyase | http://www.genecards.org/cgi-bin/carddisp.pl?gene=HAL | 0.470009843 | 0.021930378 |
| *HBA1* | Hemoglobin subunit alpha | http://www.genecards.org/cgi-bin/carddisp.pl?gene=HBA1 | 0.417543585 | 0.000529177 |
| *HBE1* | Hemoglobin subunit epsilon | http://www.genecards.org/cgi-bin/carddisp.pl?gene=HBE1 | 0.374184401 | 0.003448707 |
| *HBEGF* | Proheparin-binding EGF-like growth factor | http://www.genecards.org/cgi-bin/carddisp.pl?gene=HBEGF | 0.096178189 | 0.000510727 |
| *HERC3* | Probable E3 ubiquitin-protein ligase HERC3 | http://www.genecards.org/cgi-bin/carddisp.pl?gene=HERC3 | 0.255747855 | 0.014495864 |
| *HHIP* | Hedgehog-interacting protein | http://www.genecards.org/cgi-bin/carddisp.pl?gene=HHIP | 0.45897813 | 0.001668125 |
| *HHLA1* | HERV-H LTR-associating protein 1 | http://www.genecards.org/cgi-bin/carddisp.pl?gene=HHLA1 | 0.211538415 | 0.036195655 |
| *HIST1H2BA* | Histone H2B type 1-A | http://www.genecards.org/cgi-bin/carddisp.pl?gene=HIST1H2BA | 0.111395722 | 0.036891349 |
| *HIST1H4A* | Histone H4 | http://www.genecards.org/cgi-bin/carddisp.pl?gene=HIST1H4A | 0.465353513 | 0.000493783 |
| *HLA-DRB1* | HLA class II histocompatibility antigen, DRB1-12 beta chain | http://www.genecards.org/cgi-bin/carddisp.pl?gene=HLA-DRB1 | 0.264909446 | 9.78614E-05 |
| *HMGA1* | High mobility group protein HMG-I/HMG-Y | http://www.genecards.org/cgi-bin/carddisp.pl?gene=HMGA1 | 0.484448687 | 0.004266871 |
| *HMGB1* | High mobility group protein B1 | http://www.genecards.org/cgi-bin/carddisp.pl?gene=HMGB1 | 0.258009048 | 0.012007226 |
| *HMGB2* | High mobility group protein B2 | http://www.genecards.org/cgi-bin/carddisp.pl?gene=HMGB2 | 0.272756816 | 0.026603978 |
| *HRG* | Histidine-rich glycoprotein | http://www.genecards.org/cgi-bin/carddisp.pl?gene=HRG | 0.471682904 | 0.003142969 |
| *HSP90AA1* | Heat shock protein HSP 90-alpha | http://www.genecards.org/cgi-bin/carddisp.pl?gene=HSP90AA1 | 0.739165562 | 0.028457826 |
| *HSP90B2P* | Putative endoplasmin-like protein | http://www.genecards.org/cgi-bin/carddisp.pl?gene=HSP90B2P | 0.170570842 | 0.005445978 |
| *HSPA1L* | Heat shock 70 kDa protein 1-like | http://www.genecards.org/cgi-bin/carddisp.pl?gene=HSPA1L | 0.473045745 | 0.020897051 |
| *HSPA6* | Heat shock 70 kDa protein 6 | http://www.genecards.org/cgi-bin/carddisp.pl?gene=HSPA6 | 0.129235118 | 0.020570364 |
| *IGF2* | Insulin-like growth factor II | http://www.genecards.org/cgi-bin/carddisp.pl?gene=IGF2 | 0.28051043 | 0.00111549 |
| *IGF2R* | Cation-independent mannose-6-phosphate receptor | http://www.genecards.org/cgi-bin/carddisp.pl?gene=IGF2R | 0.547129427 | 0.023912709 |
| *IGFBP2* | Insulin-like growth factor-binding protein 2 | http://www.genecards.org/cgi-bin/carddisp.pl?gene=IGFBP2 | 0.167231978 | 0.00741237 |
| *IGFBP4* | Insulin-like growth factor-binding protein 4 | http://www.genecards.org/cgi-bin/carddisp.pl?gene=IGFBP4 | 0.310943576 | 5.78821E-07 |
| *IGFBP7* | Insulin-like growth factor-binding protein 7 | http://www.genecards.org/cgi-bin/carddisp.pl?gene=IGFBP7 | 0.520324057 | 0.000132518 |
| *ING2* | Inhibitor of growth protein 2 | http://www.genecards.org/cgi-bin/carddisp.pl?gene=ING2 | 0.130458757 | 0.001648992 |
| *IQGAP1* | Ras GTPase-activating-like protein IQGAP1 | http://www.genecards.org/cgi-bin/carddisp.pl?gene=IQGAP1 | 0.568958415 | 0.000959473 |
| *ISOC1* | Isochorismatase domain-containing protein 1 | http://www.genecards.org/cgi-bin/carddisp.pl?gene=ISOC1 | 0.492176921 | 0.009694598 |
| *ITGA2* | Integrin alpha-2 | http://www.genecards.org/cgi-bin/carddisp.pl?gene=ITGA2 | 0.566775145 | 0.0153426 |
| *ITGA2B* | Integrin alpha-IIb | http://www.genecards.org/cgi-bin/carddisp.pl?gene=ITGA2B | 0.762311432 | 0.008792137 |
| *ITIH2* | Inter-alpha-trypsin inhibitor heavy chain H2 | http://www.genecards.org/cgi-bin/carddisp.pl?gene=ITIH2 | 0.649315188 | 0.0254595 |
| *ITIH3* | Inter-alpha-trypsin inhibitor heavy chain H3 | http://www.genecards.org/cgi-bin/carddisp.pl?gene=ITIH3 | 0.554324669 | 0.002461969 |
| *ITIH4* | Inter-alpha-trypsin inhibitor heavy chain H4 | http://www.genecards.org/cgi-bin/carddisp.pl?gene=ITIH4 | 0.361090223 | 0.000953736 |
| *ITSN1* | Intersectin-1 | http://www.genecards.org/cgi-bin/carddisp.pl?gene=ITSN1 | 0.514240408 | 0.015106705 |
| *KAT6A* | Histone acetyltransferase KAT6A | http://www.genecards.org/cgi-bin/carddisp.pl?gene=KAT6A | 0.354682498 | 0.00023762 |
| *KAT6B* | Histone acetyltransferase KAT6B | http://www.genecards.org/cgi-bin/carddisp.pl?gene=KAT6B | 0.137796711 | 0.003373704 |
| *KATNAL2* | Katanin p60 ATPase-containing subunit A-like 2 | http://www.genecards.org/cgi-bin/carddisp.pl?gene=KATNAL2 | 0.001786081 | 0.008988322 |
| *KIAA0556* | Protein KIAA0556 | http://www.genecards.org/cgi-bin/carddisp.pl?gene=KIAA0556 | 0.24313021 | 0.014575212 |
| *KIAA1462* | Junctional protein associated with coronary artery disease | http://www.genecards.org/cgi-bin/carddisp.pl?gene=KIAA1462 | 0.330613772 | 0.001757675 |
| *KIF5B* | Kinesin-1 heavy chain | http://www.genecards.org/cgi-bin/carddisp.pl?gene=KIF5B | 0.452024066 | 0.000722189 |
| *KRT1* | Keratin, type II cytoskeletal 1 | http://www.genecards.org/cgi-bin/carddisp.pl?gene=KRT1 | 0.522856136 | 0.035115934 |
| *KRT10* | Keratin, type I cytoskeletal 10 | http://www.genecards.org/cgi-bin/carddisp.pl?gene=KRT10 | 0.438593368 | 0.014333856 |
| *KRT14* | Keratin, type I cytoskeletal 14 | http://www.genecards.org/cgi-bin/carddisp.pl?gene=KRT14 | 0.251737583 | 0.010185692 |
| *KRT15* | Keratin, type I cytoskeletal 15 | http://www.genecards.org/cgi-bin/carddisp.pl?gene=KRT15 | 0.072641636 | 0.001469846 |
| *KRT16* | Keratin, type I cytoskeletal 16 | http://www.genecards.org/cgi-bin/carddisp.pl?gene=KRT16 | 0.302031644 | 0.015790811 |
| *KRT17* | Keratin, type I cytoskeletal 17 | http://www.genecards.org/cgi-bin/carddisp.pl?gene=KRT17 | 0.339003027 | 0.032513415 |
| *KRT2* | Keratin, type II cytoskeletal 2 epidermal | http://www.genecards.org/cgi-bin/carddisp.pl?gene=KRT2 | 0.582881115 | 0.045674667 |
| *KRT25* | Keratin, type I cytoskeletal 25 | http://www.genecards.org/cgi-bin/carddisp.pl?gene=KRT25 | 0.396547141 | 0.000187956 |
| *KRT31* | Keratin, type I cuticular Ha1 | http://www.genecards.org/cgi-bin/carddisp.pl?gene=KRT31 | 0.233504239 | 0.02378831 |
| *KRT4* | Keratin, type II cytoskeletal 4 | http://www.genecards.org/cgi-bin/carddisp.pl?gene=KRT4 | 0.783800349 | 0.03701111 |
| *KRT5* | Keratin, type II cytoskeletal 5 | http://www.genecards.org/cgi-bin/carddisp.pl?gene=KRT5 | 0.431076415 | 0.007845464 |
| *KRT71* | Keratin, type II cytoskeletal 71 | http://www.genecards.org/cgi-bin/carddisp.pl?gene=KRT71 | 0.0224634 | 0.048785814 |
| *KRT75* | Keratin, type II cytoskeletal 75 | http://www.genecards.org/cgi-bin/carddisp.pl?gene=KRT75 | 0.209211635 | 0.006670218 |
| *KSR2* | Kinase suppressor of Ras 2 | http://www.genecards.org/cgi-bin/carddisp.pl?gene=KSR2 | 0.413453952 | 6.0882E-05 |
| *LAMA1* | Laminin subunit alpha-1 | http://www.genecards.org/cgi-bin/carddisp.pl?gene=LAMA1 | 0.14715491 | 0.000157488 |
| *LARP7* | La-related protein 7 | http://www.genecards.org/cgi-bin/carddisp.pl?gene=LARP7 | 0.111846808 | 0.009449477 |
| *LCN1* | Lipocalin-1 | http://www.genecards.org/cgi-bin/carddisp.pl?gene=LCN1 | 0.494342419 | 0.032052639 |
| *LOXL2* | Lysyl oxidase homolog 2 | http://www.genecards.org/cgi-bin/carddisp.pl?gene=LOXL2 | 0.27841825 | 0.001206009 |
| *LPXN* | Leupaxin | http://www.genecards.org/cgi-bin/carddisp.pl?gene=LPXN | 0.469726259 | 0.002531528 |
| *LRMP* | Lymphoid-restricted membrane protein | http://www.genecards.org/cgi-bin/carddisp.pl?gene=LRMP | 0.01544366 | 0.041190259 |
| *LRRC8A* | Volume-regulated anion channel subunit LRRC8A | http://www.genecards.org/cgi-bin/carddisp.pl?gene=LRRC8A | 0.231178961 | 0.007213044 |
| *LRRK2* | Leucine-rich repeat serine/threonine-protein kinase 2 | http://www.genecards.org/cgi-bin/carddisp.pl?gene=LRRK2 | 0.127224999 | 0.010728838 |
| *LSM14A* | Protein LSM14 homolog A | http://www.genecards.org/cgi-bin/carddisp.pl?gene=LSM14A | 0.297011318 | 1.13244E-05 |
| *LTBP1* | Latent-transforming growth factor beta-binding protein 1 | http://www.genecards.org/cgi-bin/carddisp.pl?gene=LTBP1 | 0.152827879 | 0.000101539 |
| *LTF* | Lactotransferrin | http://www.genecards.org/cgi-bin/carddisp.pl?gene=LTF | 0.396825664 | 2.91933E-05 |
| *MACF1* | Microtubule-actin cross-linking factor 1, isoforms 1/2/3/5 | http://www.genecards.org/cgi-bin/carddisp.pl?gene=MACF1 | 0.504844364 | 0.011884884 |
| *MAD1L1* | Mitotic spindle assembly checkpoint protein MAD1 | http://www.genecards.org/cgi-bin/carddisp.pl?gene=MAD1L1 | 0.412801701 | 0.008750134 |
| *MAN2C1* | Alpha-mannosidase 2C1 | http://www.genecards.org/cgi-bin/carddisp.pl?gene=MAN2C1 | 0.302671832 | 0.034852986 |
| *MANF* | Mesencephalic astrocyte-derived neurotrophic factor | http://www.genecards.org/cgi-bin/carddisp.pl?gene=MANF | 0.27381313 | 0.021375826 |
| *MAP4* | Microtubule-associated protein 4 | http://www.genecards.org/cgi-bin/carddisp.pl?gene=MAP4 | 0.407107159 | 0.000356215 |
| *MAP7D2* | MAP7 domain-containing protein 2 | http://www.genecards.org/cgi-bin/carddisp.pl?gene=MAP7D2 | 0.204986437 | 0.00042537 |
| *MARK2* | Serine/threonine-protein kinase MARK2 | http://www.genecards.org/cgi-bin/carddisp.pl?gene=MARK2 | 0.696946287 | 0.021151125 |
| *MASP1* | Mannan-binding lectin serine protease 1 | http://www.genecards.org/cgi-bin/carddisp.pl?gene=MASP1 | 0.258751866 | 0.000379624 |
| *MATN2* | Matrilin-2 | http://www.genecards.org/cgi-bin/carddisp.pl?gene=MATN2 | 0.678068605 | 0.00468002 |
| *MCM9* | DNA helicase MCM9 | http://www.genecards.org/cgi-bin/carddisp.pl?gene=MCM9 | 0.374338592 | 0.044916044 |
| *MDK* | Midkine | http://www.genecards.org/cgi-bin/carddisp.pl?gene=MDK | 0.143561751 | 3.87655E-05 |
| *MGAM2* | Probable maltase-glucoamylase 2 | http://www.genecards.org/cgi-bin/carddisp.pl?gene=MGAM2 | 0.481424487 | 0.001636138 |
| *MICAL2* | [F-actin]-methionine sulfoxide oxidase MICAL2 | http://www.genecards.org/cgi-bin/carddisp.pl?gene=MICAL2 | 0.098131635 | 0.002571914 |
| *MIF* | Macrophage migration inhibitory factor | http://www.genecards.org/cgi-bin/carddisp.pl?gene=MIF | 0.140604109 | 0.022915186 |
| *MLLT6* | Protein AF-17 | http://www.genecards.org/cgi-bin/carddisp.pl?gene=MLLT6 | 0.046170181 | 0.035542275 |
| *MPP1* | 55 kDa erythrocyte membrane protein | http://www.genecards.org/cgi-bin/carddisp.pl?gene=MPP1 | 0.493442036 | 0.005192664 |
| *MPRIP* | Myosin phosphatase Rho-interacting protein | http://www.genecards.org/cgi-bin/carddisp.pl?gene=MPRIP | 0.268614971 | 0.004450703 |
| *MTAP* | S-methyl-5'-thioadenosine phosphorylase | http://www.genecards.org/cgi-bin/carddisp.pl?gene=MTAP | 0.414538819 | 0.000692643 |
| *MTHFD2* | Bifunctional methylenetetrahydrofolate dehydrogenase/cyclohydrolase, mitochondrial | http://www.genecards.org/cgi-bin/carddisp.pl?gene=MTHFD2 | 0.471372375 | 0.012636465 |
| *MTPN* | Myotrophin | http://www.genecards.org/cgi-bin/carddisp.pl?gene=MTPN | 0.277870384 | 0.005103883 |
| *MYCBP* | C-Myc-binding protein | http://www.genecards.org/cgi-bin/carddisp.pl?gene=MYCBP | 0.587984636 | 3.9925E-05 |
| *MYO1A* | Unconventional myosin-Ia | http://www.genecards.org/cgi-bin/carddisp.pl?gene=MYO1A | 0.198065669 | 0.002325922 |
| *MYO5A* | Unconventional myosin-Va | http://www.genecards.org/cgi-bin/carddisp.pl?gene=MYO5A | 0.250272016 | 0.043302896 |
| *MYPN* | Myopalladin | http://www.genecards.org/cgi-bin/carddisp.pl?gene=MYPN | 0.14992771 | 0.018814154 |
| *NAB1* | NGFI-A-binding protein 1 | http://www.genecards.org/cgi-bin/carddisp.pl?gene=NAB1 | 0.205130685 | 0.009723101 |
| *NACA* | Nascent polypeptide-associated complex subunit alpha, muscle-specific form | http://www.genecards.org/cgi-bin/carddisp.pl?gene=NACA | 0.376051845 | 7.43768E-08 |
| *NCOR1* | Nuclear receptor corepressor 1 | http://www.genecards.org/cgi-bin/carddisp.pl?gene=NCOR1 | 0.510282549 | 0.024025608 |
| *NEBL* | Nebulette | http://www.genecards.org/cgi-bin/carddisp.pl?gene=NEBL | 0.061793196 | 0.001096142 |
| *NFRKB* | Nuclear factor related to kappa-B-binding protein | http://www.genecards.org/cgi-bin/carddisp.pl?gene=NFRKB | 0.226949077 | 0.003962815 |
| *NGF* | Beta-nerve growth factor | http://www.genecards.org/cgi-bin/carddisp.pl?gene=NGF | 0.47225 | 0.046463885 |
| *NLN* | Neurolysin, mitochondrial | http://www.genecards.org/cgi-bin/carddisp.pl?gene=NLN | 0.415193174 | 0.007365339 |
| *NME1* | Nucleoside diphosphate kinase A | http://www.genecards.org/cgi-bin/carddisp.pl?gene=NME1 | 0.286129277 | 0.0102015 |
| *NOV* | Protein NOV homolog | http://www.genecards.org/cgi-bin/carddisp.pl?gene=NOV | only present in RPEp53-/-STIL-/- FA | 0.037403003 |
| *NT5C1B* | Cytosolic 5'-nucleotidase 1B | http://www.genecards.org/cgi-bin/carddisp.pl?gene=NT5C1B | 0.375767125 | 0.029697645 |
| *NTN4* | Netrin-4 | http://www.genecards.org/cgi-bin/carddisp.pl?gene=NTN4 | 0.076036621 | 0.004719267 |
| *NUMB* | Protein numb homolog | http://www.genecards.org/cgi-bin/carddisp.pl?gene=NUMB | 0.765683798 | 0.00601946 |
| *NUMBL* | Numb-like protein | http://www.genecards.org/cgi-bin/carddisp.pl?gene=NUMBL | 0.64511696 | 5.28195E-05 |
| *OR6Y1* | Olfactory receptor 6Y1 | http://www.genecards.org/cgi-bin/carddisp.pl?gene=OR6Y1 | 0.129872269 | 0.016133669 |
| *P2RX5* | P2X purinoceptor 5 | http://www.genecards.org/cgi-bin/carddisp.pl?gene=P2RX5 | 0.461481361 | 0.032924452 |
| *PACSIN2* | Protein kinase C and casein kinase substrate in neurons protein 2 | http://www.genecards.org/cgi-bin/carddisp.pl?gene=PACSIN2 | 0.345919105 | 0.003576799 |
| *PARVA* | Alpha-parvin | http://www.genecards.org/cgi-bin/carddisp.pl?gene=PARVA | 0.39402308 | 0.000825867 |
| *PDE1C* | Calcium/calmodulin-dependent 3',5'-cyclic nucleotide phosphodiesterase 1C | http://www.genecards.org/cgi-bin/carddisp.pl?gene=PDE1C | 0.453487508 | 0.003865285 |
| *PFN2* | Profilin-2 | http://www.genecards.org/cgi-bin/carddisp.pl?gene=PFN2 | 0.511775735 | 0.01758196 |
| *PGK1* | Phosphoglycerate kinase 1 | http://www.genecards.org/cgi-bin/carddisp.pl?gene=PGK1 | 0.44684313 | 0.008290262 |
| *PHF10* | PHD finger protein 10 | http://www.genecards.org/cgi-bin/carddisp.pl?gene=PHF10 | 0.27524538 | 0.000418625 |
| *PHLDB2* | Pleckstrin homology-like domain family B member 2 | http://www.genecards.org/cgi-bin/carddisp.pl?gene=PHLDB2 | 0.319951173 | 0.000668806 |
| *PIP4K2A* | Phosphatidylinositol 5-phosphate 4-kinase type-2 alpha | http://www.genecards.org/cgi-bin/carddisp.pl?gene=PIP4K2A | 0.40032797 | 0.000231654 |
| *PKM* | Pyruvate kinase PKM | http://www.genecards.org/cgi-bin/carddisp.pl?gene=PKM | 0.525415563 | 0.002481859 |
| *PLA1A* | Phospholipase A1 member A | http://www.genecards.org/cgi-bin/carddisp.pl?gene=PLA1A | 0.346623883 | 4.67017E-05 |
| *PLAT* | Tissue-type plasminogen activator | http://www.genecards.org/cgi-bin/carddisp.pl?gene=PLAT | 0.383352749 | 0.002863371 |
| *PLAUR* | Urokinase plasminogen activator surface receptor | http://www.genecards.org/cgi-bin/carddisp.pl?gene=PLAUR | 0.597922166 | 0.001450398 |
| *PLEK* | Pleckstrin | http://www.genecards.org/cgi-bin/carddisp.pl?gene=PLEK | 0.328215172 | 0.004408199 |
| *PLS1* | Plastin-1 | http://www.genecards.org/cgi-bin/carddisp.pl?gene=PLS1 | 0.054630159 | 0.004770892 |
| *POLK* | DNA polymerase kappa | http://www.genecards.org/cgi-bin/carddisp.pl?gene=POLK | 0.321903703 | 0.005389875 |
| *POSTN* | Periostin | http://www.genecards.org/cgi-bin/carddisp.pl?gene=POSTN | 0.478661674 | 0.022251496 |
| *POTEF* | POTE ankyrin domain family member F | http://www.genecards.org/cgi-bin/carddisp.pl?gene=POTEF | 0.540794715 | 0.017561574 |
| *PPFIA3* | Liprin-alpha-3 | http://www.genecards.org/cgi-bin/carddisp.pl?gene=PPFIA3 | 0.311138888 | 0.001077174 |
| *PPIA* | Peptidyl-prolyl cis-trans isomerase A | http://www.genecards.org/cgi-bin/carddisp.pl?gene=PPIA | 0.684220158 | 0.003429177 |
| *PPIE* | Peptidyl-prolyl cis-trans isomerase E | http://www.genecards.org/cgi-bin/carddisp.pl?gene=PPIE | 0.324699727 | 0.000247233 |
| *PRDM1* | PR domain zinc finger protein 1 | http://www.genecards.org/cgi-bin/carddisp.pl?gene=PRDM1 | 0.214930721 | 0.017154948 |
| *PRLR* | Prolactin receptor | http://www.genecards.org/cgi-bin/carddisp.pl?gene=PRLR | 0.398627636 | 0.015177544 |
| *PRSS12* | Neurotrypsin | http://www.genecards.org/cgi-bin/carddisp.pl?gene=PRSS12 | 0.340201476 | 0.000434726 |
| *PRSS23* | Serine protease 23 | http://www.genecards.org/cgi-bin/carddisp.pl?gene=PRSS23 | 0.616934692 | 0.021967337 |
| *PXDN* | Peroxidasin homolog | http://www.genecards.org/cgi-bin/carddisp.pl?gene=PXDN | 0.274493744 | 2.39143E-05 |
| *QSOX1* | Sulfhydryl oxidase 1 | http://www.genecards.org/cgi-bin/carddisp.pl?gene=QSOX1 | 0.383886952 | 0.002414202 |
| *RABGAP1* | Rab GTPase-activating protein 1 | http://www.genecards.org/cgi-bin/carddisp.pl?gene=RABGAP1 | 0.460630295 | 0.000757295 |
| *RAI14* | Ankycorbin | http://www.genecards.org/cgi-bin/carddisp.pl?gene=RAI14 | 0.535038664 | 0.012217123 |
| *RAN* | GTP-binding nuclear protein Ran | http://www.genecards.org/cgi-bin/carddisp.pl?gene=RAN | 0.481757498 | 1.61796E-05 |
| *RDX* | Radixin | http://www.genecards.org/cgi-bin/carddisp.pl?gene=RDX | 0.606047641 | 0.001180763 |
| *RIMBP3* | RIMS-binding protein 3A | http://www.genecards.org/cgi-bin/carddisp.pl?gene=RIMBP3 | 0.219613807 | 0.000555172 |
| *RNASE4* | Ribonuclease 4 | http://www.genecards.org/cgi-bin/carddisp.pl?gene=RNASE4 | 0.268496493 | 0.004737149 |
| *RNF40* | E3 ubiquitin-protein ligase BRE1B | http://www.genecards.org/cgi-bin/carddisp.pl?gene=RNF40 | 0.064821527 | 0.004379248 |
| *RPL24* | 60S ribosomal protein L24 | http://www.genecards.org/cgi-bin/carddisp.pl?gene=RPL24 | 0.726029234 | 0.018968616 |
| *RPLP2* | 60S acidic ribosomal protein P2 | http://www.genecards.org/cgi-bin/carddisp.pl?gene=RPLP2 | 0.634978865 | 0.048883635 |
| *RPS10P5* | Putative 40S ribosomal protein S10-like | http://www.genecards.org/cgi-bin/carddisp.pl?gene=RPS10P5 | 0.47187186 | 0.005680055 |
| *RPS25* | 40S ribosomal protein S25 | http://www.genecards.org/cgi-bin/carddisp.pl?gene=RPS25 | 0.291035551 | 0.000944721 |
| *RPS27A* | Ubiquitin-40S ribosomal protein S27a | http://www.genecards.org/cgi-bin/carddisp.pl?gene=RPS27A | 0.705451154 | 0.008488046 |
| *RSF1* | Remodeling and spacing factor 1 | http://www.genecards.org/cgi-bin/carddisp.pl?gene=RSF1 | 0.604866732 | 0.013965476 |
| *SACS* | Sacsin | http://www.genecards.org/cgi-bin/carddisp.pl?gene=SACS | 0.415230686 | 0.026924516 |
| *SBDS* | Ribosome maturation protein SBDS | http://www.genecards.org/cgi-bin/carddisp.pl?gene=SBDS | 0.198490094 | 0.002990815 |
| *SCN11A* | Sodium channel protein type 11 subunit alpha | http://www.genecards.org/cgi-bin/carddisp.pl?gene=SCN11A | 0.574796909 | 0.024471526 |
| *SDC2* | Syndecan-2 | http://www.genecards.org/cgi-bin/carddisp.pl?gene=SDC2 | 0.604436018 | 0.022715089 |
| *SEMA3B* | Semaphorin-3B | http://www.genecards.org/cgi-bin/carddisp.pl?gene=SEMA3B | 0.539791842 | 0.005984381 |
| *SEMA3C* | Semaphorin-3C | http://www.genecards.org/cgi-bin/carddisp.pl?gene=SEMA3C | 0.473115989 | 0.00012807 |
| *SEPT1* | Septin-1 | http://www.genecards.org/cgi-bin/carddisp.pl?gene=SEPT1 | 0.282525515 | 0.042550438 |
| *SEPT7* | Septin-7 | http://www.genecards.org/cgi-bin/carddisp.pl?gene=SEPT7 | 0.415817495 | 0.000467516 |
| *SERBP1* | Plasminogen activator inhibitor 1 RNA-binding protein | http://www.genecards.org/cgi-bin/carddisp.pl?gene=SERBP1 | 0.644154479 | 0.002231696 |
| *SERF2* | Small EDRK-rich factor 2 | http://www.genecards.org/cgi-bin/carddisp.pl?gene=SERF2 | 0.431402642 | 0.00625924 |
| *SERPINC1* | Antithrombin-III | http://www.genecards.org/cgi-bin/carddisp.pl?gene=SERPINC1 | 0.556957015 | 0.008143238 |
| *SERPINF1* | Pigment epithelium-derived factor | http://www.genecards.org/cgi-bin/carddisp.pl?gene=SERPINF1 | 0.153496731 | 0.011854131 |
| *SFRP1* | Secreted frizzled-related protein 1 | http://www.genecards.org/cgi-bin/carddisp.pl?gene=SFRP1 | 0.696514506 | 0.026525279 |
| *SIM1* | Single-minded homolog 1 | http://www.genecards.org/cgi-bin/carddisp.pl?gene=SIM1 | 0.129763384 | 0.035173138 |
| *SIRPA* | Tyrosine-protein phosphatase non-receptor type substrate 1 | http://www.genecards.org/cgi-bin/carddisp.pl?gene=SIRPA | 0.225051752 | 0.009602429 |
| *SLC13A3* | Solute carrier family 13 member 3 | http://www.genecards.org/cgi-bin/carddisp.pl?gene=SLC13A3 | 0.39536138 | 0.021711646 |
| *SLC1A5* | Neutral amino acid transporter B(0) | http://www.genecards.org/cgi-bin/carddisp.pl?gene=SLC1A5 | 0.470141929 | 0.000613124 |
| *SLC25A3* | Phosphate carrier protein, mitochondrial | http://www.genecards.org/cgi-bin/carddisp.pl?gene=SLC25A3 | 0.471881059 | 0.019619043 |
| *SLC29A1* | Equilibrative nucleoside transporter 1 | http://www.genecards.org/cgi-bin/carddisp.pl?gene=SLC29A1 | 0.235129647 | 0.00762204 |
| *SMC2* | Structural maintenance of chromosomes protein 2 | http://www.genecards.org/cgi-bin/carddisp.pl?gene=SMC2 | 0.302338064 | 0.008798758 |
| *SNRPD3* | Small nuclear ribonucleoprotein Sm D3 | http://www.genecards.org/cgi-bin/carddisp.pl?gene=SNRPD3 | 0.248372745 | 0.001023608 |
| *SREK1* | Splicing regulatory glutamine/lysine-rich protein 1 | http://www.genecards.org/cgi-bin/carddisp.pl?gene=SREK1 | 0.367959214 | 0.001129883 |
| *SRSF3* | Serine/arginine-rich splicing factor 3 | http://www.genecards.org/cgi-bin/carddisp.pl?gene=SRSF3 | 0.225425661 | 0.000128154 |
| *ST13P5* | Putative protein FAM10A5 | http://www.genecards.org/cgi-bin/carddisp.pl?gene=ST13P5 | 0.679551588 | 0.036921532 |
| *ST7* | Suppressor of tumorigenicity 7 protein | http://www.genecards.org/cgi-bin/carddisp.pl?gene=ST7 | 0.320364404 | 0.026272098 |
| *STC1* | Stanniocalcin-1 | http://www.genecards.org/cgi-bin/carddisp.pl?gene=STC1 | 0.044240325 | 0.000591431 |
| *STIM2* | Stromal interaction molecule 2 | http://www.genecards.org/cgi-bin/carddisp.pl?gene=STIM2 | 0.113100465 | 0.011870808 |
| *STXBP3* | Syntaxin-binding protein 3 | http://www.genecards.org/cgi-bin/carddisp.pl?gene=STXBP3 | 0.301881411 | 0.004385452 |
| *SYNE2* | Nesprin-2 | http://www.genecards.org/cgi-bin/carddisp.pl?gene=SYNE2 | 0.616021003 | 0.009079947 |
| *TAF4B* | Transcription initiation factor TFIID subunit 4B | http://www.genecards.org/cgi-bin/carddisp.pl?gene=TAF4B | 0.638923753 | 0.017761223 |
| *TAGLN* | Transgelin | http://www.genecards.org/cgi-bin/carddisp.pl?gene=TAGLN | 0.204704674 | 0.002066813 |
| *TALDO1* | Transaldolase | http://www.genecards.org/cgi-bin/carddisp.pl?gene=TALDO1 | 0.257091355 | 0.023876073 |
| *TAOK2* | Serine/threonine-protein kinase TAO2 | http://www.genecards.org/cgi-bin/carddisp.pl?gene=TAOK2 | 0.297639617 | 0.005100885 |
| *TAX1BP1* | Tax1-binding protein 1 | http://www.genecards.org/cgi-bin/carddisp.pl?gene=TAX1BP1 | 0.246511195 | 0.000791428 |
| *TBC1D16* | TBC1 domain family member 16 | http://www.genecards.org/cgi-bin/carddisp.pl?gene=TBC1D16 | 0.257356903 | 8.03861E-05 |
| *TBL3* | Transducin beta-like protein 3 | http://www.genecards.org/cgi-bin/carddisp.pl?gene=TBL3 | 0.05666463 | 0.042785791 |
| *TDRD15* | Tudor domain-containing protein 15 | http://www.genecards.org/cgi-bin/carddisp.pl?gene=TDRD15 | 0.458702306 | 0.000767311 |
| *TEAD1* | Transcriptional enhancer factor TEF-1 | http://www.genecards.org/cgi-bin/carddisp.pl?gene=TEAD1 | 0.009301278 | 0.022457572 |
| *TF* | Serotransferrin | http://www.genecards.org/cgi-bin/carddisp.pl?gene=TF | 0.390142729 | 0.032830835 |
| *TFPI* | Tissue factor pathway inhibitor | http://www.genecards.org/cgi-bin/carddisp.pl?gene=TFPI | 0.505749103 | 0.003738265 |
| *TFPI2* | Tissue factor pathway inhibitor 2 | http://www.genecards.org/cgi-bin/carddisp.pl?gene=TFPI2 | 0.343013745 | 0.000656919 |
| *TGFB1* | Transforming growth factor beta-1 | http://www.genecards.org/cgi-bin/carddisp.pl?gene=TGFB1 | 0.236923613 | 4.44746E-05 |
| *TGFB2* | Transforming growth factor beta-2 | http://www.genecards.org/cgi-bin/carddisp.pl?gene=TGFB2 | 0.504414755 | 0.002893442 |
| *TGOLN2* | Trans-Golgi network integral membrane protein 2 | http://www.genecards.org/cgi-bin/carddisp.pl?gene=TGOLN2 | 0.647354974 | 0.012930977 |
| *THBS1* | Thrombospondin-1 | http://www.genecards.org/cgi-bin/carddisp.pl?gene=THBS1 | 0.798786839 | 0.007281067 |
| *THBS2* | Thrombospondin-2 | http://www.genecards.org/cgi-bin/carddisp.pl?gene=THBS2 | 0.651047094 | 0.036774812 |
| *THBS4* | Thrombospondin-4 | http://www.genecards.org/cgi-bin/carddisp.pl?gene=THBS4 | 0.173566884 | 0.000706891 |
| *THRAP3* | Thyroid hormone receptor-associated protein 3 | http://www.genecards.org/cgi-bin/carddisp.pl?gene=THRAP3 | 0.476731002 | 0.018402658 |
| *TIMP3* | Metalloproteinase inhibitor 3 | http://www.genecards.org/cgi-bin/carddisp.pl?gene=TIMP3 | 0.296947065 | 0.004238507 |
| *TJAP1* | Tight junction-associated protein 1 | http://www.genecards.org/cgi-bin/carddisp.pl?gene=TJAP1 | 0.396403408 | 0.000199007 |
| *TLL1* | Tolloid-like protein 1 | http://www.genecards.org/cgi-bin/carddisp.pl?gene=TLL1 | 0.337143797 | 0.000539259 |
| *TLR5* | Toll-like receptor 5 | http://www.genecards.org/cgi-bin/carddisp.pl?gene=TLR5 | 0.323983369 | 0.013256677 |
| *TMEFF1* | Tomoregulin-1 | http://www.genecards.org/cgi-bin/carddisp.pl?gene=TMEFF1 | 0.268337852 | 0.048458151 |
| *TMEM2* | Transmembrane protein 2 | http://www.genecards.org/cgi-bin/carddisp.pl?gene=TMEM2 | 0.428223853 | 0.00338974 |
| *TMPRSS4* | Transmembrane protease serine 4 | http://www.genecards.org/cgi-bin/carddisp.pl?gene=TMPRSS4 | 0.198296462 | 0.020784358 |
| *TPD52L2* | Tumor protein D54 | http://www.genecards.org/cgi-bin/carddisp.pl?gene=TPD52L2 | 0.390061309 | 0.034790984 |
| *TRDN* | Triadin | http://www.genecards.org/cgi-bin/carddisp.pl?gene=TRDN | 0.167907937 | 0.009154615 |
| *TRIM4* | E3 ubiquitin-protein ligase TRIM4 | http://www.genecards.org/cgi-bin/carddisp.pl?gene=TRIM4 | 0.114169572 | 0.011025808 |
| *TRIOBP* | TRIO and F-actin-binding protein | http://www.genecards.org/cgi-bin/carddisp.pl?gene=TRIOBP | 0.29775083 | 8.3985E-05 |
| *TUBA4A* | Tubulin alpha-4A chain | http://www.genecards.org/cgi-bin/carddisp.pl?gene=TUBA4A | 0.388745948 | 0.00285034 |
| *TUBB* | Tubulin beta chain | http://www.genecards.org/cgi-bin/carddisp.pl?gene=TUBB | 0.440663333 | 0.000367856 |
| *TXN* | Thioredoxin | http://www.genecards.org/cgi-bin/carddisp.pl?gene=TXN | 0.628243887 | 0.022643613 |
| *TXNRD1* | Thioredoxin reductase 1, cytoplasmic | http://www.genecards.org/cgi-bin/carddisp.pl?gene=TXNRD1 | 0.107353207 | 0.001938225 |
| *TYK2* | Non-receptor tyrosine-protein kinase TYK2 | http://www.genecards.org/cgi-bin/carddisp.pl?gene=TYK2 | 0.256883186 | 0.003430298 |
| *TYMSOS* | TYMS opposite strand protein | http://www.genecards.org/cgi-bin/carddisp.pl?gene=TYMSOS | 0.419525466 | 0.034462941 |
| *UACA* | Uveal autoantigen with coiled-coil domains and ankyrin repeats | http://www.genecards.org/cgi-bin/carddisp.pl?gene=UACA | 0.308123586 | 4.42776E-06 |
| *UBE2N* | Ubiquitin-conjugating enzyme E2 N | http://www.genecards.org/cgi-bin/carddisp.pl?gene=UBE2N | 0.575746845 | 0.003339382 |
| *UBE2V1* | Ubiquitin-conjugating enzyme E2 variant 1 | http://www.genecards.org/cgi-bin/carddisp.pl?gene=UBE2V1 | 0.219557996 | 0.005640187 |
| *UBR4* | E3 ubiquitin-protein ligase UBR4 | http://www.genecards.org/cgi-bin/carddisp.pl?gene=UBR4 | 0.129420091 | 0.001135026 |
| *UBTFL1* | Upstream-binding factor 1-like protein 1 | http://www.genecards.org/cgi-bin/carddisp.pl?gene=UBTFL1 | 0.155483596 | 0.008103286 |
| *UNC80* | Protein unc-80 homolog | http://www.genecards.org/cgi-bin/carddisp.pl?gene=UNC80 | 0.291527943 | 0.013033886 |
| *UTP20* | Small subunit processome component 20 homolog | http://www.genecards.org/cgi-bin/carddisp.pl?gene=UTP20 | 0.23156433 | 0.014768717 |
| *UTRN* | Utrophin | http://www.genecards.org/cgi-bin/carddisp.pl?gene=UTRN | 0.537863308 | 0.023531491 |
| *VTN* | Vitronectin | http://www.genecards.org/cgi-bin/carddisp.pl?gene=VTN | 0.424021174 | 0.009209212 |
| *VWA7* | von Willebrand factor A domain-containing protein 7 | http://www.genecards.org/cgi-bin/carddisp.pl?gene=VWA7 | 0.023048867 | 0.001024923 |
| *VWF* | von Willebrand factor | http://www.genecards.org/cgi-bin/carddisp.pl?gene=VWF | 0.271804869 | 0.007901846 |
| *WRN* | Werner syndrome ATP-dependent helicase | http://www.genecards.org/cgi-bin/carddisp.pl?gene=WRN | 0.728313155 | 0.039654381 |
| *XAGE5* | X antigen family member 5 | http://www.genecards.org/cgi-bin/carddisp.pl?gene=XAGE5 | 0.096571958 | 0.004571714 |
| *ZBED9* | SCAN domain-containing protein 3 | http://www.genecards.org/cgi-bin/carddisp.pl?gene=ZBED9 | 0.525472036 | 0.004868464 |
| *ZC3H15* | Zinc finger CCCH domain-containing protein 15 | http://www.genecards.org/cgi-bin/carddisp.pl?gene=ZC3H15 | 0.326460072 | 0.00171843 |
| *ZC3H7A* | Zinc finger CCCH domain-containing protein 7A | http://www.genecards.org/cgi-bin/carddisp.pl?gene=ZC3H7A | 0.10378839 | 2.62782E-05 |
| *ZNF140* | Zinc finger protein 140 | http://www.genecards.org/cgi-bin/carddisp.pl?gene=ZNF140 | 0.371933933 | 0.001672825 |
| *ZNF211* | Zinc finger protein 211 | http://www.genecards.org/cgi-bin/carddisp.pl?gene=ZNF211 | 0.036195365 | 0.005175069 |
| *ZNF561* | Zinc finger protein 561 | http://www.genecards.org/cgi-bin/carddisp.pl?gene=ZNF561 | 0.630878709 | 0.013041959 |
| *ZNF648* | Zinc finger protein 648 | http://www.genecards.org/cgi-bin/carddisp.pl?gene=ZNF648 | 0.437727796 | 0.032872897 |

**The same abundance in RPEp53^-/-^ and RPEp53^-/-^STIL^-/-^ FAs**

| **Gene name** | **Description** | [**Gene information**](https://l.facebook.com/l.php?u=http%3A%2F%2Fwww.genecards.org%2Fcgi-bin%2Fcarddisp.pl%3Fgene%3D%2522%252CA1&h=ATP6yswR14bvN0ocja1Ey1G_azEX6hPvaoaG6wnlhO80hCNibRxOBC7mpa7DI2EWkCMhK9LIGIVnWVtcmb2ldic7GGL5x8e6YusaY-ljmcwOhdMPSq3PhaYfIKBlTYELWIWn) | **Centrosome Dependence Ratio** | **p-value**  **(Student's t-test)** |
| --- | --- | --- | --- | --- |
| *ABCA6* | ATP-binding cassette sub-family A member 6 | http://www.genecards.org/cgi-bin/carddisp.pl?gene=ABCA6 | 1.020052807 | 0.464658483 |
| *ABCF1* | ATP-binding cassette sub-family F member 1 | http://www.genecards.org/cgi-bin/carddisp.pl?gene=ABCF1 | 2.53534503 | 0.117532366 |
| *ACO1* | Cytoplasmic aconitate hydratase | http://www.genecards.org/cgi-bin/carddisp.pl?gene=ACO1 | 1.470277322 | 0.259241525 |
| *ACOX1* | Peroxisomal acyl-coenzyme A oxidase 1 | http://www.genecards.org/cgi-bin/carddisp.pl?gene=ACOX1 | 3.18309053 | 0.17686972 |
| *ACSL4* | Long-chain-fatty-acid--CoA ligase 4 | http://www.genecards.org/cgi-bin/carddisp.pl?gene=ACSL4 | 1.275591658 | 0.166470154 |
| *ACTC1* | Actin, alpha cardiac muscle 1 | http://www.genecards.org/cgi-bin/carddisp.pl?gene=ACTC1 | 0.907561574 | 0.440977861 |
| *ACTR2* | Actin-related protein 2 | http://www.genecards.org/cgi-bin/carddisp.pl?gene=ACTR2 | 0.840920876 | 0.140116156 |
| *ADAMTS15* | A disintegrin and metalloproteinase with thrombospondin motifs 15 | http://www.genecards.org/cgi-bin/carddisp.pl?gene=ADAMTS15 | 7.546181998 | 0.101648759 |
| *AFP* | Alpha-fetoprotein | http://www.genecards.org/cgi-bin/carddisp.pl?gene=AFP | 1.558058576 | 0.102206439 |
| *AGPAT1* | 1-acyl-sn-glycerol-3-phosphate acyltransferase alpha | http://www.genecards.org/cgi-bin/carddisp.pl?gene=AGPAT1 | 214.9960346 | 0.094088354 |
| *AGRN* | Agrin | http://www.genecards.org/cgi-bin/carddisp.pl?gene=AGRN | 0.490478583 | 0.069538241 |
| *AKAP2* | A-kinase anchor protein 2 | http://www.genecards.org/cgi-bin/carddisp.pl?gene=AKAP2 | 2.422628127 | 0.057101363 |
| *ALDH18A1* | Delta-1-pyrroline-5-carboxylate synthase | http://www.genecards.org/cgi-bin/carddisp.pl?gene=ALDH18A1 | 1.472639766 | 0.142372987 |
| *ANK3* | Ankyrin-3 | http://www.genecards.org/cgi-bin/carddisp.pl?gene=ANK3 | 1.002743369 | 0.494818825 |
| *ANO2* | Anoctamin-2 | http://www.genecards.org/cgi-bin/carddisp.pl?gene=ANO2 | 0.453823775 | 0.102012041 |
| *ANXA2* | Annexin A2 | http://www.genecards.org/cgi-bin/carddisp.pl?gene=ANXA2 | 0.895065954 | 0.131024423 |
| *ANXA5* | Annexin A5 | http://www.genecards.org/cgi-bin/carddisp.pl?gene=ANXA5 | 1.284790169 | 0.274602589 |
| *ANXA6* | Annexin A6 | http://www.genecards.org/cgi-bin/carddisp.pl?gene=ANXA6 | 1.283164816 | 0.152589449 |
| *AP2A1* | AP-2 complex subunit alpha-1 | http://www.genecards.org/cgi-bin/carddisp.pl?gene=AP2A1 | 0.987445904 | 0.449254344 |
| *AP2A2* | AP-2 complex subunit alpha-2 | http://www.genecards.org/cgi-bin/carddisp.pl?gene=AP2A2 | 1.044532059 | 0.121665908 |
| *AP2B1* | AP-2 complex subunit beta | http://www.genecards.org/cgi-bin/carddisp.pl?gene=AP2B1 | 0.849925745 | 0.052074588 |
| *AP2S1* | AP-2 complex subunit sigma | http://www.genecards.org/cgi-bin/carddisp.pl?gene=AP2S1 | 1.173147881 | 0.259899439 |
| *AP3B1* | AP-3 complex subunit beta-1 | http://www.genecards.org/cgi-bin/carddisp.pl?gene=AP3B1 | 0.364526039 | 0.067363167 |
| *APOA1* | Apolipoprotein A-I | http://www.genecards.org/cgi-bin/carddisp.pl?gene=APOA1 | 0.329939017 | 0.052571202 |
| *APOA4* | Apolipoprotein A-IV | http://www.genecards.org/cgi-bin/carddisp.pl?gene=APOA4 | 1.919125954 | 0.234199132 |
| *ARF4* | ADP-ribosylation factor 4 | http://www.genecards.org/cgi-bin/carddisp.pl?gene=ARF4 | 0.320239343 | 0.066994586 |
| *ARFIP1* | Arfaptin-1 | http://www.genecards.org/cgi-bin/carddisp.pl?gene=ARFIP1 | 18.80772699 | 0.113854754 |
| *ARHGAP22* | Rho GTPase-activating protein 22 | http://www.genecards.org/cgi-bin/carddisp.pl?gene=ARHGAP22 | 1.574188907 | 0.205573624 |
| *ARHGEF2* | Rho guanine nucleotide exchange factor 2 | http://www.genecards.org/cgi-bin/carddisp.pl?gene=ARHGEF2 | 1.057003638 | 0.338828733 |
| *ARL6IP1* | ADP-ribosylation factor-like protein 6-interacting protein 1 | http://www.genecards.org/cgi-bin/carddisp.pl?gene=ARL6IP1 | 6.034195763 | 0.056867515 |
| *ARL6IP5* | PRA1 family protein 3 | http://www.genecards.org/cgi-bin/carddisp.pl?gene=ARL6IP5 | 0.794509151 | 0.293141923 |
| *ASAP2* | Arf-GAP with SH3 domain, ANK repeat and PH domain-containing protein 2 | http://www.genecards.org/cgi-bin/carddisp.pl?gene=ASAP2 | 0.826161918 | 0.341189316 |
| *ATP1B1* | Sodium/potassium-transporting ATPase subunit beta-1 | http://www.genecards.org/cgi-bin/carddisp.pl?gene=ATP1B1 | 1.106643618 | 0.397572064 |
| *ATP2A1* | Sarcoplasmic/endoplasmic reticulum calcium ATPase 1 | http://www.genecards.org/cgi-bin/carddisp.pl?gene=ATP2A1 | 1.115999369 | 0.25159548 |
| *ATP2B1* | Plasma membrane calcium-transporting ATPase 1 | http://www.genecards.org/cgi-bin/carddisp.pl?gene=ATP2B1 | 0.626044978 | 0.179659759 |
| *ATP4A* | Potassium-transporting ATPase alpha chain 1 | http://www.genecards.org/cgi-bin/carddisp.pl?gene=ATP4A | 0.735566459 | 0.054436396 |
| *ATP5E* | ATP synthase subunit epsilon, mitochondrial | http://www.genecards.org/cgi-bin/carddisp.pl?gene=ATP5E | 3.971990688 | 0.057810909 |
| *ATP5H* | ATP synthase subunit d, mitochondrial | http://www.genecards.org/cgi-bin/carddisp.pl?gene=ATP5H | 0.655514649 | 0.061388003 |
| *ATP6V0A4* | V-type proton ATPase 116 kDa subunit a isoform 4 | http://www.genecards.org/cgi-bin/carddisp.pl?gene=ATP6V0A4 | 2.10960888 | 0.248917091 |
| *ATP7A* | Copper-transporting ATPase 1 | http://www.genecards.org/cgi-bin/carddisp.pl?gene=ATP7A | 0.247706573 | 0.059216907 |
| *ATRX* | Transcriptional regulator ATRX | http://www.genecards.org/cgi-bin/carddisp.pl?gene=ATRX | 0.924677383 | 0.245937106 |
| *B2M* | Beta-2-microglobulin | http://www.genecards.org/cgi-bin/carddisp.pl?gene=B2M | 0.713179153 | 0.067030427 |
| *BASP1* | Brain acid soluble protein 1 | http://www.genecards.org/cgi-bin/carddisp.pl?gene=BASP1 | 0.932696559 | 0.336762512 |
| *BAZ1A* | Bromodomain adjacent to zinc finger domain protein 1A | http://www.genecards.org/cgi-bin/carddisp.pl?gene=BAZ1A | 1.024222355 | 0.467047461 |
| *BAZ2B* | Bromodomain adjacent to zinc finger domain protein 2B | http://www.genecards.org/cgi-bin/carddisp.pl?gene=BAZ2B | 0.601817115 | 0.097550354 |
| *BCAP31* | B-cell receptor-associated protein 31 | http://www.genecards.org/cgi-bin/carddisp.pl?gene=BCAP31 | 2.66853877 | 0.075660144 |
| *BCL10* | B-cell lymphoma/leukemia 10 | http://www.genecards.org/cgi-bin/carddisp.pl?gene=BCL10 | 1.027293956 | 0.464967744 |
| *BDNF* | Brain-derived neurotrophic factor | http://www.genecards.org/cgi-bin/carddisp.pl?gene=BDNF | 0.639640191 | 0.054477083 |
| *BDP1* | Transcription factor TFIIIB component B'' homolog | http://www.genecards.org/cgi-bin/carddisp.pl?gene=BDP1 | 0.601403847 | 0.056719541 |
| *BEND7* | BEN domain-containing protein 7 | http://www.genecards.org/cgi-bin/carddisp.pl?gene=BEND7 | 1.615129534 | 0.174435545 |
| *BLVRB* | Flavin reductase (NADPH) | http://www.genecards.org/cgi-bin/carddisp.pl?gene=BLVRB | 0.612537915 | 0.113675788 |
| *BMP1* | Bone morphogenetic protein 1 | http://www.genecards.org/cgi-bin/carddisp.pl?gene=BMP1 | 0.731321486 | 0.091013969 |
| *BRCA1* | Breast cancer type 1 susceptibility protein | http://www.genecards.org/cgi-bin/carddisp.pl?gene=BRCA1 | 1.025923 | 0.429094629 |
| *BRWD3* | Bromodomain and WD repeat-containing protein 3 | http://www.genecards.org/cgi-bin/carddisp.pl?gene=BRWD3 | 0.510368623 | 0.075791573 |
| *BTF3* | Transcription factor BTF3 | http://www.genecards.org/cgi-bin/carddisp.pl?gene=BTF3 | 0.79702653 | 0.245951316 |
| *C1QBP* | Complement component 1 Q subcomponent-binding protein, mitochondrial | http://www.genecards.org/cgi-bin/carddisp.pl?gene=C1QBP | 1.284810005 | 0.140513531 |
| *C20orf27* | UPF0687 protein C20orf27 | http://www.genecards.org/cgi-bin/carddisp.pl?gene=C20orf27 | 0.663023326 | 0.242560194 |
| *C3orf58* | Deleted in autism protein 1 | http://www.genecards.org/cgi-bin/carddisp.pl?gene=C3orf58 | 0.713616803 | 0.213250415 |
| *C9* | Complement component C9 | http://www.genecards.org/cgi-bin/carddisp.pl?gene=C9 | 1.058679086 | 0.459702923 |
| *CACNA2D1* | Voltage-dependent calcium channel subunit alpha-2/delta-1 | http://www.genecards.org/cgi-bin/carddisp.pl?gene=CACNA2D1 | 0.512765398 | 0.195343171 |
| *CACTIN-AS1* | Putative uncharacterized protein encoded by CACTIN-AS1 | http://www.genecards.org/cgi-bin/carddisp.pl?gene=CACTIN-AS1 | 1.690523113 | 0.130177127 |
| *CALML5* | Calmodulin-like protein 5 | http://www.genecards.org/cgi-bin/carddisp.pl?gene=CALML5 | 0.731757654 | 0.102827858 |
| *CAMTA1* | Calmodulin-binding transcription activator 1 | http://www.genecards.org/cgi-bin/carddisp.pl?gene=CAMTA1 | 1.58927221 | 0.125605359 |
| *CAP1* | Adenylyl cyclase-associated protein 1 | http://www.genecards.org/cgi-bin/carddisp.pl?gene=CAP1 | 0.518015951 | 0.095329295 |
| *CAPRIN1* | Caprin-1 | http://www.genecards.org/cgi-bin/carddisp.pl?gene=CAPRIN1 | 0.851509976 | 0.144956013 |
| *CBR1* | Carbonyl reductase [NADPH] 1 | http://www.genecards.org/cgi-bin/carddisp.pl?gene=CBR1 | 1.040713931 | 0.451743975 |
| *CC2D1A* | Coiled-coil and C2 domain-containing protein 1A | http://www.genecards.org/cgi-bin/carddisp.pl?gene=CC2D1A | 0.416535438 | 0.08525554 |
| *CCDC158* | Coiled-coil domain-containing protein 158 | http://www.genecards.org/cgi-bin/carddisp.pl?gene=CCDC158 | 0.82250501 | 0.26791869 |
| *CCDC50* | Coiled-coil domain-containing protein 50 | http://www.genecards.org/cgi-bin/carddisp.pl?gene=CCDC50 | 0.058887713 | 0.117179188 |
| *CCDC62* | Coiled-coil domain-containing protein 62 | http://www.genecards.org/cgi-bin/carddisp.pl?gene=CCDC62 | 0.618418667 | 0.097131118 |
| *CCDC96* | Coiled-coil domain-containing protein 96 | http://www.genecards.org/cgi-bin/carddisp.pl?gene=CCDC96 | 1.029975746 | 0.476269344 |
| *CCIN* | Calicin | http://www.genecards.org/cgi-bin/carddisp.pl?gene=CCIN | 0.67963956 | 0.05548132 |
| *CCL18* | C-C motif chemokine 18 | http://www.genecards.org/cgi-bin/carddisp.pl?gene=CCL18 | 0.101250434 | 0.07384334 |
| *CCL26* | C-C motif chemokine 26 | http://www.genecards.org/cgi-bin/carddisp.pl?gene=CCL26 | 0.633134969 | 0.192047087 |
| *CD101* | Immunoglobulin superfamily member 2 | http://www.genecards.org/cgi-bin/carddisp.pl?gene=CD101 | 1.283482465 | 0.263402402 |
| *CD109* | CD109 antigen | http://www.genecards.org/cgi-bin/carddisp.pl?gene=CD109 | 1.81752582 | 0.181836393 |
| *CD151* | CD151 antigen | http://www.genecards.org/cgi-bin/carddisp.pl?gene=CD151 | 5.993444529 | 0.08161169 |
| *CD276* | CD276 antigen | http://www.genecards.org/cgi-bin/carddisp.pl?gene=CD276 | 3.729247493 | 0.053442878 |
| *CD97* | CD97 antigen | http://www.genecards.org/cgi-bin/carddisp.pl?gene=CD97 | 4.464251317 | 0.069892282 |
| *CD99L2* | CD99 antigen-like protein 2 | http://www.genecards.org/cgi-bin/carddisp.pl?gene=CD99L2 | 1.10846959 | 0.38227973 |
| *CDC42* | Cell division control protein 42 homolog | http://www.genecards.org/cgi-bin/carddisp.pl?gene=CDC42 | 2.863405022 | 0.123788731 |
| *CDC42EP1* | Cdc42 effector protein 1 | http://www.genecards.org/cgi-bin/carddisp.pl?gene=CDC42EP1 | 1.630084501 | 0.202176528 |
| *CDC42EP4* | Cdc42 effector protein 4 | http://www.genecards.org/cgi-bin/carddisp.pl?gene=CDC42EP4 | 5.599360966 | 0.080682 |
| *CDH13* | Cadherin-13 | http://www.genecards.org/cgi-bin/carddisp.pl?gene=CDH13 | 1.033219411 | 0.383659451 |
| *CDH2* | Cadherin-2 | http://www.genecards.org/cgi-bin/carddisp.pl?gene=CDH2 | 1.423914235 | 0.062289774 |
| *CDH23* | Cadherin-23 | http://www.genecards.org/cgi-bin/carddisp.pl?gene=CDH23 | 1.100288999 | 0.393756643 |
| *CDK8* | Cyclin-dependent kinase 8 | http://www.genecards.org/cgi-bin/carddisp.pl?gene=CDK8 | 1.375026273 | 0.21695028 |
| *CDT1* | DNA replication factor Cdt1 | http://www.genecards.org/cgi-bin/carddisp.pl?gene=CDT1 | 0.924872215 | 0.42548834 |
| *CELSR1* | Cadherin EGF LAG seven-pass G-type receptor 1 | http://www.genecards.org/cgi-bin/carddisp.pl?gene=CELSR1 | 3.465628405 | 0.111975767 |
| *CEP192* | Centrosomal protein of 192 kDa | http://www.genecards.org/cgi-bin/carddisp.pl?gene=CEP192 | 0.73627463 | 0.132662877 |
| *CETN3* | Centrin-3 | http://www.genecards.org/cgi-bin/carddisp.pl?gene=CETN3 | 0.285953177 | 0.063618551 |
| *CFL1* | Cofilin-1 | http://www.genecards.org/cgi-bin/carddisp.pl?gene=CFL1 | 1.022001968 | 0.478468528 |
| *CKAP5* | Cytoskeleton-associated protein 5 | http://www.genecards.org/cgi-bin/carddisp.pl?gene=CKAP5 | 0.742978176 | 0.26146537 |
| *CLASP1* | CLIP-associating protein 1 | http://www.genecards.org/cgi-bin/carddisp.pl?gene=CLASP1 | 0.418948177 | 0.090783209 |
| *CLIC4* | Chloride intracellular channel protein 4 | http://www.genecards.org/cgi-bin/carddisp.pl?gene=CLIC4 | 0.890707755 | 0.309526925 |
| *CLMP* | CXADR-like membrane protein | http://www.genecards.org/cgi-bin/carddisp.pl?gene=CLMP | 0.847518551 | 0.300546015 |
| *CLTC* | Clathrin heavy chain 1 | http://www.genecards.org/cgi-bin/carddisp.pl?gene=CLTC | 1.129919279 | 0.049979489 |
| *CNP* | 2',3'-cyclic-nucleotide 3'-phosphodiesterase | http://www.genecards.org/cgi-bin/carddisp.pl?gene=CNP | 0.649137213 | 0.071462081 |
| *CNTN3* | Contactin-3 | http://www.genecards.org/cgi-bin/carddisp.pl?gene=CNTN3 | 1.634079305 | 0.295232455 |
| *COBL* | Protein cordon-bleu | http://www.genecards.org/cgi-bin/carddisp.pl?gene=COBL | 2.659977508 | 0.059755521 |
| *COL12A1* | Collagen alpha-1(XII) chain | http://www.genecards.org/cgi-bin/carddisp.pl?gene=COL12A1 | 1.99936279 | 0.07054505 |
| *COL17A1* | Collagen alpha-1(XVII) chain | http://www.genecards.org/cgi-bin/carddisp.pl?gene=COL17A1 | 1.552130542 | 0.199865047 |
| *COL1A1* | Collagen alpha-1(I) chain | http://www.genecards.org/cgi-bin/carddisp.pl?gene=COL1A1 | 0.105624868 | 0.196893354 |
| *COL4A1* | Collagen alpha-1(IV) chain | http://www.genecards.org/cgi-bin/carddisp.pl?gene=COL4A1 | 31.55041171 | 0.070748671 |
| *COL4A2* | Collagen alpha-2(IV) chain | http://www.genecards.org/cgi-bin/carddisp.pl?gene=COL4A2 | 0.712620947 | 0.074409174 |
| *COL8A1* | Collagen alpha-1(VIII) chain | http://www.genecards.org/cgi-bin/carddisp.pl?gene=COL8A1 | 1.011794977 | 0.409057846 |
| *COTL1* | Coactosin-like protein | http://www.genecards.org/cgi-bin/carddisp.pl?gene=COTL1 | 1.138911838 | 0.354482833 |
| *CPNE2* | Copine-2 | http://www.genecards.org/cgi-bin/carddisp.pl?gene=CPNE2 | 0.915751009 | 0.441457871 |
| *CPNE3* | Copine-3 | http://www.genecards.org/cgi-bin/carddisp.pl?gene=CPNE3 | 1.165792479 | 0.345881145 |
| *CPSF1* | Cleavage and polyadenylation specificity factor subunit 1 | http://www.genecards.org/cgi-bin/carddisp.pl?gene=CPSF1 | 4.050555508 | 0.148019931 |
| *CPZ* | Carboxypeptidase Z | http://www.genecards.org/cgi-bin/carddisp.pl?gene=CPZ | 1.113255551 | 0.262120461 |
| *CRACR2A* | EF-hand calcium-binding domain-containing protein 4B | http://www.genecards.org/cgi-bin/carddisp.pl?gene=CRACR2A | 2.935857081 | 0.252220149 |
| *CRKL* | Crk-like protein | http://www.genecards.org/cgi-bin/carddisp.pl?gene=CRKL | 1.969223911 | 0.069805275 |
| *CSF3R* | Granulocyte colony-stimulating factor receptor | http://www.genecards.org/cgi-bin/carddisp.pl?gene=CSF3R | 1.784648634 | 0.114264412 |
| *CSPG4* | Chondroitin sulfate proteoglycan 4 | http://www.genecards.org/cgi-bin/carddisp.pl?gene=CSPG4 | 1.026512554 | 0.432307156 |
| *CTAGE1* | cTAGE family member 2 | http://www.genecards.org/cgi-bin/carddisp.pl?gene=CTAGE1 | 0.988386224 | 0.473735722 |
| *CTNNB1* | Catenin beta-1 | http://www.genecards.org/cgi-bin/carddisp.pl?gene=CTNNB1 | 0.56244981 | 0.0735306 |
| *CTTNBP2* | Cortactin-binding protein 2 | http://www.genecards.org/cgi-bin/carddisp.pl?gene=CTTNBP2 | 1.144923688 | 0.38377989 |
| *CUTA* | Protein CutA | http://www.genecards.org/cgi-bin/carddisp.pl?gene=CUTA | 1.16366825 | 0.353817626 |
| *CWC22* | Pre-mRNA-splicing factor CWC22 homolog | http://www.genecards.org/cgi-bin/carddisp.pl?gene=CWC22 | 0.604385069 | 0.189870711 |
| *CYFIP1* | Cytoplasmic FMR1-interacting protein 1 | http://www.genecards.org/cgi-bin/carddisp.pl?gene=CYFIP1 | 1.200999338 | 0.177880598 |
| *CYR61* | Protein CYR61 | http://www.genecards.org/cgi-bin/carddisp.pl?gene=CYR61 | 0.793661877 | 0.057763286 |
| *DAG1* | Dystroglycan | http://www.genecards.org/cgi-bin/carddisp.pl?gene=DAG1 | 1.332654981 | 0.12088301 |
| *DCAF12L2* | DDB1- and CUL4-associated factor 12-like protein 2 | http://www.genecards.org/cgi-bin/carddisp.pl?gene=DCAF12L2 | 0.039839892 | 0.053836818 |
| *DDR2* | Discoidin domain-containing receptor 2 | http://www.genecards.org/cgi-bin/carddisp.pl?gene=DDR2 | 0.373912538 | 0.085425914 |
| *DENND6A* | Protein DENND6A | http://www.genecards.org/cgi-bin/carddisp.pl?gene=DENND6A | 1.250871434 | 0.25327426 |
| *DERL2* | Derlin-2 | http://www.genecards.org/cgi-bin/carddisp.pl?gene=DERL2 | 17.1394346 | 0.094856246 |
| *DES* | Desmin | http://www.genecards.org/cgi-bin/carddisp.pl?gene=DES | 0.277942343 | 0.129709136 |
| *DIAPH1* | Protein diaphanous homolog 1 | http://www.genecards.org/cgi-bin/carddisp.pl?gene=DIAPH1 | 0.127259889 | 0.112941023 |
| *DNAH5* | Dynein heavy chain 5, axonemal | http://www.genecards.org/cgi-bin/carddisp.pl?gene=DNAH5 | 0.834174702 | 0.06329013 |
| *DNAH8* | Dynein heavy chain 8, axonemal | http://www.genecards.org/cgi-bin/carddisp.pl?gene=DNAH8 | 1.68680207 | 0.114806552 |
| *DNHD1* | Dynein heavy chain domain-containing protein 1 | http://www.genecards.org/cgi-bin/carddisp.pl?gene=DNHD1 | 2.541218985 | 0.12013832 |
| *DOPEY1* | Protein dopey-1 | http://www.genecards.org/cgi-bin/carddisp.pl?gene=DOPEY1 | 0.003439252 | 0.195132445 |
| *DPYSL2* | Dihydropyrimidinase-related protein 2 | http://www.genecards.org/cgi-bin/carddisp.pl?gene=DPYSL2 | 0.746986012 | 0.138983763 |
| *DSC1* | Desmocollin-1 | http://www.genecards.org/cgi-bin/carddisp.pl?gene=DSC1 | 0.680602316 | 0.201411839 |
| *DSP* | Desmoplakin | http://www.genecards.org/cgi-bin/carddisp.pl?gene=DSP | 0.424080697 | 0.079761746 |
| *DSTN* | Destrin | http://www.genecards.org/cgi-bin/carddisp.pl?gene=DSTN | 1.448412062 | 0.060251764 |
| *DUOX2* | Dual oxidase 2 | http://www.genecards.org/cgi-bin/carddisp.pl?gene=DUOX2 | 0.953082054 | 0.461915853 |
| *DYNC1H1* | Cytoplasmic dynein 1 heavy chain 1 | http://www.genecards.org/cgi-bin/carddisp.pl?gene=DYNC1H1 | 2.022421083 | 0.053699149 |
| *DYSF* | Dysferlin | http://www.genecards.org/cgi-bin/carddisp.pl?gene=DYSF | 0.706958521 | 0.056325178 |
| *E2F7* | Transcription factor E2F7 | http://www.genecards.org/cgi-bin/carddisp.pl?gene=E2F7 | 6.431839457 | 0.15984333 |
| *EDIL3* | EGF-like repeat and discoidin I-like domain-containing protein 3 | http://www.genecards.org/cgi-bin/carddisp.pl?gene=EDIL3 | 1.207286309 | 0.161038457 |
| *EEF1A1* | Elongation factor 1-alpha 1 | http://www.genecards.org/cgi-bin/carddisp.pl?gene=EEF1A1 | 0.77716232 | 0.221795852 |
| *EEF1D* | Elongation factor 1-delta | http://www.genecards.org/cgi-bin/carddisp.pl?gene=EEF1D | 1.029921577 | 0.42033484 |
| *EEF1G* | Elongation factor 1-gamma | http://www.genecards.org/cgi-bin/carddisp.pl?gene=EEF1G | 1.262772668 | 0.091379335 |
| *EEF2* | Elongation factor 2 | http://www.genecards.org/cgi-bin/carddisp.pl?gene=EEF2 | 0.88931738 | 0.197274676 |
| *EFHD2* | EF-hand domain-containing protein D2 | http://www.genecards.org/cgi-bin/carddisp.pl?gene=EFHD2 | 1.420027261 | 0.12517301 |
| *EFNB1* | Ephrin-B1 | http://www.genecards.org/cgi-bin/carddisp.pl?gene=EFNB1 | 13.12019429 | 0.09486667 |
| *EHBP1L1* | EH domain-binding protein 1-like protein 1 | http://www.genecards.org/cgi-bin/carddisp.pl?gene=EHBP1L1 | 1.701975059 | 0.103228714 |
| *EHD1* | EH domain-containing protein 1 | http://www.genecards.org/cgi-bin/carddisp.pl?gene=EHD1 | 0.738917385 | 0.130699115 |
| *EHD2* | EH domain-containing protein 2 | http://www.genecards.org/cgi-bin/carddisp.pl?gene=EHD2 | 0.94165272 | 0.303824391 |
| *EHD3* | EH domain-containing protein 3 | http://www.genecards.org/cgi-bin/carddisp.pl?gene=EHD3 | 1.639016062 | 0.22420941 |
| *EIF2S2* | Eukaryotic translation initiation factor 2 subunit 2 | http://www.genecards.org/cgi-bin/carddisp.pl?gene=EIF2S2 | 0.766115191 | 0.060863702 |
| *EIF3G* | Eukaryotic translation initiation factor 3 subunit G | http://www.genecards.org/cgi-bin/carddisp.pl?gene=EIF3G | 0.557106313 | 0.085974511 |
| *EIF4A1* | Eukaryotic initiation factor 4A-I | http://www.genecards.org/cgi-bin/carddisp.pl?gene=EIF4A1 | 1.30187563 | 0.115783371 |
| *EIF4A3* | Eukaryotic initiation factor 4A-III | http://www.genecards.org/cgi-bin/carddisp.pl?gene=EIF4A3 | 0.565860312 | 0.105239861 |
| *EIF4G1* | Eukaryotic translation initiation factor 4 gamma 1 | http://www.genecards.org/cgi-bin/carddisp.pl?gene=EIF4G1 | 0.577347242 | 0.089293416 |
| *EMB* | Embigin | http://www.genecards.org/cgi-bin/carddisp.pl?gene=EMB | 0.902127228 | 0.349818443 |
| *ENAH* | Protein enabled homolog | http://www.genecards.org/cgi-bin/carddisp.pl?gene=ENAH | 0.015064305 | 0.069343924 |
| *ENO1* | Alpha-enolase | http://www.genecards.org/cgi-bin/carddisp.pl?gene=ENO1 | 1.017448219 | 0.401125429 |
| *EPN1* | Epsin-1 | http://www.genecards.org/cgi-bin/carddisp.pl?gene=EPN1 | 0.751029436 | 0.20825793 |
| *ERBIN* | Erbin | http://www.genecards.org/cgi-bin/carddisp.pl?gene=ERBIN | 0.138919077 | 0.089583076 |
| *ESR2* | Estrogen receptor beta | http://www.genecards.org/cgi-bin/carddisp.pl?gene=ESR2 | 0.557566149 | 0.195255516 |
| *ESYT2* | Extended synaptotagmin-2 | http://www.genecards.org/cgi-bin/carddisp.pl?gene=ESYT2 | 1.142942458 | 0.217162321 |
| *ETF1* | Eukaryotic peptide chain release factor subunit 1 | http://www.genecards.org/cgi-bin/carddisp.pl?gene=ETF1 | 1.952740373 | 0.163313563 |
| *EVA1B* | Protein eva-1 homolog B | http://www.genecards.org/cgi-bin/carddisp.pl?gene=EVA1B | 3.850221497 | 0.099402819 |
| *EXTL1* | Exostosin-like 1 | http://www.genecards.org/cgi-bin/carddisp.pl?gene=EXTL1 | 0.138372854 | 0.052412605 |
| *F10* | Coagulation factor X | http://www.genecards.org/cgi-bin/carddisp.pl?gene=F10 | 0.743768982 | 0.067983821 |
| *F13A1* | Coagulation factor XIII A chain | http://www.genecards.org/cgi-bin/carddisp.pl?gene=F13A1 | 0.584565933 | 0.132312135 |
| *F13B* | Coagulation factor XIII B chain | http://www.genecards.org/cgi-bin/carddisp.pl?gene=F13B | 0.498291164 | 0.064065998 |
| *F8* | Coagulation factor VIII | http://www.genecards.org/cgi-bin/carddisp.pl?gene=F8 | 0.828315097 | 0.209352671 |
| *FABP5* | Fatty acid-binding protein, epidermal | http://www.genecards.org/cgi-bin/carddisp.pl?gene=FABP5 | 0.300789715 | 0.059422773 |
| *FAM120C* | Constitutive coactivator of PPAR-gamma-like protein 2 | http://www.genecards.org/cgi-bin/carddisp.pl?gene=FAM120C | 0.44417701 | 0.15801751 |
| *FAM49B* | Protein FAM49B | http://www.genecards.org/cgi-bin/carddisp.pl?gene=FAM49B | 0.541229109 | 0.06885827 |
| *FANK1* | Fibronectin type 3 and ankyrin repeat domains protein 1 | http://www.genecards.org/cgi-bin/carddisp.pl?gene=FANK1 | 0.736880202 | 0.26333562 |
| *FAT2* | Protocadherin Fat 2 | http://www.genecards.org/cgi-bin/carddisp.pl?gene=FAT2 | 0.888671265 | 0.250461897 |
| *FBLN1* | Fibulin-1 | http://www.genecards.org/cgi-bin/carddisp.pl?gene=FBLN1 | 0.780283271 | 0.2442649 |
| *FBN2* | Fibrillin-2 | http://www.genecards.org/cgi-bin/carddisp.pl?gene=FBN2 | 1.316453594 | 0.173708487 |
| *FCHSD2* | F-BAR and double SH3 domains protein 2 | http://www.genecards.org/cgi-bin/carddisp.pl?gene=FCHSD2 | 1.210298214 | 0.328636521 |
| *FH* | Fumarate hydratase, mitochondrial | http://www.genecards.org/cgi-bin/carddisp.pl?gene=FH | 6.185241463 | 0.057243177 |
| *FHL1* | Four and a half LIM domains protein 1 | http://www.genecards.org/cgi-bin/carddisp.pl?gene=FHL1 | 1.072029775 | 0.424471787 |
| *FHOD1* | FH1/FH2 domain-containing protein 1 | http://www.genecards.org/cgi-bin/carddisp.pl?gene=FHOD1 | 2.093327045 | 0.095647642 |
| *FKBP1A* | Peptidyl-prolyl cis-trans isomerase FKBP1A | http://www.genecards.org/cgi-bin/carddisp.pl?gene=FKBP1A | 0.443349799 | 0.052632652 |
| *FKBP3* | Peptidyl-prolyl cis-trans isomerase FKBP3 | http://www.genecards.org/cgi-bin/carddisp.pl?gene=FKBP3 | 0.48247146 | 0.087978284 |
| *FMN2* | Formin-2 | http://www.genecards.org/cgi-bin/carddisp.pl?gene=FMN2 | 2.395276293 | 0.196660223 |
| *FSIP2* | Fibrous sheath-interacting protein 2 | http://www.genecards.org/cgi-bin/carddisp.pl?gene=FSIP2 | 0.621153617 | 0.156670892 |
| *FST* | Follistatin | http://www.genecards.org/cgi-bin/carddisp.pl?gene=FST | 0.820740679 | 0.382322658 |
| *G3BP1* | Ras GTPase-activating protein-binding protein 1 | http://www.genecards.org/cgi-bin/carddisp.pl?gene=G3BP1 | 1.221717502 | 0.231758481 |
| *GALNT12* | Polypeptide N-acetylgalactosaminyltransferase 12 | http://www.genecards.org/cgi-bin/carddisp.pl?gene=GALNT12 | 28.88868484 | 0.050723931 |
| *GAPDH* | Glyceraldehyde-3-phosphate dehydrogenase | http://www.genecards.org/cgi-bin/carddisp.pl?gene=GAPDH | 1.43055276 | 0.071252297 |
| *GDF5* | Growth/differentiation factor 5 | http://www.genecards.org/cgi-bin/carddisp.pl?gene=GDF5 | 0.002181285 | 0.089073535 |
| *GDI2* | Rab GDP dissociation inhibitor beta | http://www.genecards.org/cgi-bin/carddisp.pl?gene=GDI2 | 0.692108836 | 0.200487734 |
| *GGT2* | Inactive gamma-glutamyltranspeptidase 2 | http://www.genecards.org/cgi-bin/carddisp.pl?gene=GGT2 | 0.726788306 | 0.143629172 |
| *GIT1* | ARF GTPase-activating protein GIT1 | http://www.genecards.org/cgi-bin/carddisp.pl?gene=GIT1 | 0.219431771 | 0.152823306 |
| *GLIPR1* | Glioma pathogenesis-related protein 1 | http://www.genecards.org/cgi-bin/carddisp.pl?gene=GLIPR1 | 3.142587989 | 0.105170537 |
| *GLS* | Glutaminase kidney isoform, mitochondrial | http://www.genecards.org/cgi-bin/carddisp.pl?gene=GLS | 1.66603197 | 0.158418305 |
| *GMFB* | Glia maturation factor beta | http://www.genecards.org/cgi-bin/carddisp.pl?gene=GMFB | 1.431122504 | 0.160144848 |
| *GNAQ* | Guanine nucleotide-binding protein G(q) subunit alpha | http://www.genecards.org/cgi-bin/carddisp.pl?gene=GNAQ | 1.457662815 | 0.054946265 |
| *GNAS* | Guanine nucleotide-binding protein G(s) subunit alpha isoforms XLas | http://www.genecards.org/cgi-bin/carddisp.pl?gene=GNAS | 0.111572306 | 0.078049005 |
| *GNB4* | Guanine nucleotide-binding protein subunit beta-4 | http://www.genecards.org/cgi-bin/carddisp.pl?gene=GNB4 | 1.681805525 | 0.082997819 |
| *GPD2* | Glycerol-3-phosphate dehydrogenase, mitochondrial | http://www.genecards.org/cgi-bin/carddisp.pl?gene=GPD2 | 0.896700428 | 0.323028304 |
| *GPI* | Glucose-6-phosphate isomerase | http://www.genecards.org/cgi-bin/carddisp.pl?gene=GPI | 8.763938533 | 0.050541989 |
| *GPRC5A* | Retinoic acid-induced protein 3 | http://www.genecards.org/cgi-bin/carddisp.pl?gene=GPRC5A | 1.9421873 | 0.052745078 |
| *GRB2* | Growth factor receptor-bound protein 2 | http://www.genecards.org/cgi-bin/carddisp.pl?gene=GRB2 | 1.474190737 | 0.144160785 |
| *GRIN2D* | Glutamate receptor ionotropic, NMDA 2D | http://www.genecards.org/cgi-bin/carddisp.pl?gene=GRIN2D | 0.602724157 | 0.07960194 |
| *GSTA3* | Glutathione S-transferase A3 | http://www.genecards.org/cgi-bin/carddisp.pl?gene=GSTA3 | 1.005681203 | 0.491925464 |
| *GTF2E1* | General transcription factor IIE subunit 1 | http://www.genecards.org/cgi-bin/carddisp.pl?gene=GTF2E1 | 0.636549837 | 0.097685781 |
| *GULP1* | PTB domain-containing engulfment adapter protein 1 | http://www.genecards.org/cgi-bin/carddisp.pl?gene=GULP1 | 1.28748755 | 0.288474648 |
| *HBB* | Hemoglobin subunit beta | http://www.genecards.org/cgi-bin/carddisp.pl?gene=HBB | 1.096931457 | 0.354930246 |
| *HDAC7* | Histone deacetylase 7 | http://www.genecards.org/cgi-bin/carddisp.pl?gene=HDAC7 | 1.018571241 | 0.489307196 |
| *HERC5* | E3 ISG15--protein ligase HERC5 | http://www.genecards.org/cgi-bin/carddisp.pl?gene=HERC5 | 0.005071243 | 0.082822911 |
| *HIST1H1C* | Histone H1.2 | http://www.genecards.org/cgi-bin/carddisp.pl?gene=HIST1H1C | 0.981245671 | 0.463327395 |
| *HIST1H2AJ* | Histone H2A type 1-J | http://www.genecards.org/cgi-bin/carddisp.pl?gene=HIST1H2AJ | 3.004032457 | 0.098089009 |
| *HIST2H2BF* | Histone H2B type 2-F | http://www.genecards.org/cgi-bin/carddisp.pl?gene=HIST2H2BF | 1.205418335 | 0.30895484 |
| *HK1* | Hexokinase-1 | http://www.genecards.org/cgi-bin/carddisp.pl?gene=HK1 | 1.290870735 | 0.141599896 |
| *HPCA* | Neuron-specific calcium-binding protein hippocalcin | http://www.genecards.org/cgi-bin/carddisp.pl?gene=HPCA | 0.250693051 | 0.087343482 |
| *HPCAL1* | Hippocalcin-like protein 1 | http://www.genecards.org/cgi-bin/carddisp.pl?gene=HPCAL1 | 1.253749848 | 0.278487799 |
| *HRNR* | Hornerin | http://www.genecards.org/cgi-bin/carddisp.pl?gene=HRNR | 0.643541338 | 0.095453212 |
| *HSP90AB1* | Heat shock protein HSP 90-beta | http://www.genecards.org/cgi-bin/carddisp.pl?gene=HSP90AB1 | 0.833170476 | 0.104028538 |
| *HSP90AB2P* | Putative heat shock protein HSP 90-beta 2 | http://www.genecards.org/cgi-bin/carddisp.pl?gene=HSP90AB2P | 0.788287682 | 0.304848005 |
| *HSP90AB3P* | Putative heat shock protein HSP 90-beta-3 | http://www.genecards.org/cgi-bin/carddisp.pl?gene=HSP90AB3P | 0.764614847 | 0.200137277 |
| *HSP90AB4P* | Putative heat shock protein HSP 90-beta 4 | http://www.genecards.org/cgi-bin/carddisp.pl?gene=HSP90AB4P | 0.980638422 | 0.468819252 |
| *HSPA2* | Heat shock-related 70 kDa protein 2 | http://www.genecards.org/cgi-bin/carddisp.pl?gene=HSPA2 | 0.682222672 | 0.114447053 |
| *HSPA8* | Heat shock cognate 71 kDa protein | http://www.genecards.org/cgi-bin/carddisp.pl?gene=HSPA8 | 1.137502416 | 0.232053081 |
| *HSPB1* | Heat shock protein beta-1 | http://www.genecards.org/cgi-bin/carddisp.pl?gene=HSPB1 | 1.185908327 | 0.196558297 |
| *HSPD1* | 60 kDa heat shock protein, mitochondrial | http://www.genecards.org/cgi-bin/carddisp.pl?gene=HSPD1 | 1.51458944 | 0.143012224 |
| *IGFBP5* | Insulin-like growth factor-binding protein 5 | http://www.genecards.org/cgi-bin/carddisp.pl?gene=IGFBP5 | 1.145583323 | 0.141502161 |
| *IGFN1* | Immunoglobulin-like and fibronectin type III domain-containing protein 1 | http://www.genecards.org/cgi-bin/carddisp.pl?gene=IGFN1 | 1.174282074 | 0.323142222 |
| *IMPDH2* | Inosine-5'-monophosphate dehydrogenase 2 | http://www.genecards.org/cgi-bin/carddisp.pl?gene=IMPDH2 | 1.169195714 | 0.423623775 |
| *INA* | Alpha-internexin | http://www.genecards.org/cgi-bin/carddisp.pl?gene=INA | 0.95776716 | 0.406443413 |
| *IQGAP2* | Ras GTPase-activating-like protein IQGAP2 | http://www.genecards.org/cgi-bin/carddisp.pl?gene=IQGAP2 | 1.655747219 | 0.124437462 |
| *JUP* | Junction plakoglobin | http://www.genecards.org/cgi-bin/carddisp.pl?gene=JUP | 0.772765949 | 0.170747485 |
| *KCNK10* | Potassium channel subfamily K member 10 | http://www.genecards.org/cgi-bin/carddisp.pl?gene=KCNK10 | 0.78903373 | 0.096806124 |
| *KIF18A* | Kinesin-like protein KIF18A | http://www.genecards.org/cgi-bin/carddisp.pl?gene=KIF18A | 19.28938192 | 0.123450619 |
| *KIF20B* | Kinesin-like protein KIF20B | http://www.genecards.org/cgi-bin/carddisp.pl?gene=KIF20B | 0.528711032 | 0.139715344 |
| *KIF26B* | Kinesin-like protein KIF26B | http://www.genecards.org/cgi-bin/carddisp.pl?gene=KIF26B | 7.612677925 | 0.054478822 |
| *KIF5A* | Kinesin heavy chain isoform 5A | http://www.genecards.org/cgi-bin/carddisp.pl?gene=KIF5A | 0.787685667 | 0.294600179 |
| *KPNA2* | Importin subunit alpha-1 | http://www.genecards.org/cgi-bin/carddisp.pl?gene=KPNA2 | 0.078126882 | 0.078396413 |
| *KRT6A* | Keratin, type II cytoskeletal 6A | http://www.genecards.org/cgi-bin/carddisp.pl?gene=KRT6A | 0.454698454 | 0.067093852 |
| *KRT6B* | Keratin, type II cytoskeletal 6B | http://www.genecards.org/cgi-bin/carddisp.pl?gene=KRT6B | 9.22544154 | 0.217448071 |
| *KRT78* | Keratin, type II cytoskeletal 78 | http://www.genecards.org/cgi-bin/carddisp.pl?gene=KRT78 | 1.648020037 | 0.210309881 |
| *KRT9* | Keratin, type I cytoskeletal 9 | http://www.genecards.org/cgi-bin/carddisp.pl?gene=KRT9 | 0.509221218 | 0.061150726 |
| *LAMC1* | Laminin subunit gamma-1 | http://www.genecards.org/cgi-bin/carddisp.pl?gene=LAMC1 | 0.764083665 | 0.077002649 |
| *LATS1* | Serine/threonine-protein kinase LATS1 | http://www.genecards.org/cgi-bin/carddisp.pl?gene=LATS1 | 1.153205937 | 0.323111011 |
| *LBP* | Lipopolysaccharide-binding protein | http://www.genecards.org/cgi-bin/carddisp.pl?gene=LBP | 0.991491494 | 0.489780569 |
| *LDHA* | L-lactate dehydrogenase A chain | http://www.genecards.org/cgi-bin/carddisp.pl?gene=LDHA | 0.974722076 | 0.350883192 |
| *LDHB* | L-lactate dehydrogenase B chain | http://www.genecards.org/cgi-bin/carddisp.pl?gene=LDHB | 1.517809769 | 0.128328208 |
| *LGALS1* | Galectin-1 | http://www.genecards.org/cgi-bin/carddisp.pl?gene=LGALS1 | 1.370670207 | 0.161258298 |
| *LHFPL2* | Lipoma HMGIC fusion partner-like 2 protein | http://www.genecards.org/cgi-bin/carddisp.pl?gene=LHFPL2 | 1.215252224 | 0.14754461 |
| *LIMD1* | LIM domain-containing protein 1 | http://www.genecards.org/cgi-bin/carddisp.pl?gene=LIMD1 | 0.81367084 | 0.05550975 |
| *LIMS1* | LIM and senescent cell antigen-like-containing domain protein 1 | http://www.genecards.org/cgi-bin/carddisp.pl?gene=LIMS1 | 1.043020502 | 0.326613969 |
| *LLGL1* | Lethal(2) giant larvae protein homolog 1 | http://www.genecards.org/cgi-bin/carddisp.pl?gene=LLGL1 | 12.70886446 | 0.06242465 |
| *LNPEP* | Leucyl-cystinyl aminopeptidase | http://www.genecards.org/cgi-bin/carddisp.pl?gene=LNPEP | 2.694363241 | 0.074330849 |
| *LRP1* | Prolow-density lipoprotein receptor-related protein 1 | http://www.genecards.org/cgi-bin/carddisp.pl?gene=LRP1 | 1.893655498 | 0.119817644 |
| *LRPPRC* | Leucine-rich PPR motif-containing protein, mitochondrial | http://www.genecards.org/cgi-bin/carddisp.pl?gene=LRPPRC | 1.179734597 | 0.255313952 |
| *LRRIQ3* | Leucine-rich repeat and IQ domain-containing protein 3 | http://www.genecards.org/cgi-bin/carddisp.pl?gene=LRRIQ3 | 0.776185864 | 0.318938426 |
| *LYZ* | Lysozyme C | http://www.genecards.org/cgi-bin/carddisp.pl?gene=LYZ | 0.757432141 | 0.326060791 |
| *MAP2* | Microtubule-associated protein 2 | http://www.genecards.org/cgi-bin/carddisp.pl?gene=MAP2 | 1.02802131 | 0.485372407 |
| *MAP6* | Microtubule-associated protein 6 | http://www.genecards.org/cgi-bin/carddisp.pl?gene=MAP6 | 0.779721944 | 0.32283177 |
| *MARCKS* | Myristoylated alanine-rich C-kinase substrate | http://www.genecards.org/cgi-bin/carddisp.pl?gene=MARCKS | 1.353618766 | 0.100340987 |
| *MARCKSL1* | MARCKS-related protein | http://www.genecards.org/cgi-bin/carddisp.pl?gene=MARCKSL1 | 1.301560231 | 0.245403729 |
| *MMP14* | Matrix metalloproteinase-14 | http://www.genecards.org/cgi-bin/carddisp.pl?gene=MMP14 | 0.887109379 | 0.336190178 |
| *MMRN1* | Multimerin-1 | http://www.genecards.org/cgi-bin/carddisp.pl?gene=MMRN1 | 0.780109216 | 0.086337709 |
| *MMS22L* | Protein MMS22-like | http://www.genecards.org/cgi-bin/carddisp.pl?gene=MMS22L | 0.61841206 | 0.060736004 |
| *MSN* | Moesin | http://www.genecards.org/cgi-bin/carddisp.pl?gene=MSN | 1.042384848 | 0.369918374 |
| *MUC16* | Mucin-16 | http://www.genecards.org/cgi-bin/carddisp.pl?gene=MUC16 | 1.086831158 | 0.433132795 |
| *MUC20* | Mucin-20 | http://www.genecards.org/cgi-bin/carddisp.pl?gene=MUC20 | 0.86773334 | 0.329190537 |
| *MYH11* | Myosin-11 | http://www.genecards.org/cgi-bin/carddisp.pl?gene=MYH11 | 0.48400132 | 0.087289964 |
| *MYH14* | Myosin-14 | http://www.genecards.org/cgi-bin/carddisp.pl?gene=MYH14 | 1.38789817 | 0.082084342 |
| *MYLK* | Myosin light chain kinase, smooth muscle | http://www.genecards.org/cgi-bin/carddisp.pl?gene=MYLK | 1.11987958 | 0.296169251 |
| *MYO18B* | Unconventional myosin-XVIIIb | http://www.genecards.org/cgi-bin/carddisp.pl?gene=MYO18B | 1.605768465 | 0.222971429 |
| *MYO19* | Unconventional myosin-XIX | http://www.genecards.org/cgi-bin/carddisp.pl?gene=MYO19 | 2.287188787 | 0.113844012 |
| *MYO1B* | Unconventional myosin-Ib | http://www.genecards.org/cgi-bin/carddisp.pl?gene=MYO1B | 0.465918484 | 0.079094755 |
| *MYO1C* | Unconventional myosin-Ic | http://www.genecards.org/cgi-bin/carddisp.pl?gene=MYO1C | 0.583540751 | 0.096160692 |
| *MYO5B* | Unconventional myosin-Vb | http://www.genecards.org/cgi-bin/carddisp.pl?gene=MYO5B | 0.175797121 | 0.086730291 |
| *MYO9A* | Unconventional myosin-IXa | http://www.genecards.org/cgi-bin/carddisp.pl?gene=MYO9A | 0.682219566 | 0.121990611 |
| *MYT1L* | Myelin transcription factor 1-like protein | http://www.genecards.org/cgi-bin/carddisp.pl?gene=MYT1L | 1.318203539 | 0.137130173 |
| *NAPSA* | Napsin-A | http://www.genecards.org/cgi-bin/carddisp.pl?gene=NAPSA | 0.934247808 | 0.415638754 |
| *NCL* | Nucleolin | http://www.genecards.org/cgi-bin/carddisp.pl?gene=NCL | 0.91346745 | 0.231730559 |
| *NDUFS8* | NADH dehydrogenase [ubiquinone] iron-sulfur protein 8, mitochondrial | http://www.genecards.org/cgi-bin/carddisp.pl?gene=NDUFS8 | 2.5853481 | 0.138596215 |
| *NECTIN2* | Nectin-2 | http://www.genecards.org/cgi-bin/carddisp.pl?gene=NECTIN2 | 0.808059553 | 0.1877426 |
| *NEUROG3* | Neurogenin-3 | http://www.genecards.org/cgi-bin/carddisp.pl?gene=NEUROG3 | 0.848019254 | 0.296247441 |
| *NOTCH2* | Neurogenic locus notch homolog protein 2 | http://www.genecards.org/cgi-bin/carddisp.pl?gene=NOTCH2 | 1.111619171 | 0.38674407 |
| *NPR2* | Atrial natriuretic peptide receptor 2 | http://www.genecards.org/cgi-bin/carddisp.pl?gene=NPR2 | 3.16011646 | 0.060367391 |
| *NRIP1* | Nuclear receptor-interacting protein 1 | http://www.genecards.org/cgi-bin/carddisp.pl?gene=NRIP1 | 13.74344182 | 0.128045226 |
| *NRK* | Nik-related protein kinase | http://www.genecards.org/cgi-bin/carddisp.pl?gene=NRK | 5.863403769 | 0.05153497 |
| *OCC1* | Overexpressed in colon carcinoma 1 protein | http://www.genecards.org/cgi-bin/carddisp.pl?gene=OCC1 | 0.852129666 | 0.222188833 |
| *P4HB* | Protein disulfide-isomerase | http://www.genecards.org/cgi-bin/carddisp.pl?gene=P4HB | 0.981476511 | 0.451435674 |
| *PACSIN3* | Protein kinase C and casein kinase substrate in neurons protein 3 | http://www.genecards.org/cgi-bin/carddisp.pl?gene=PACSIN3 | 0.659878601 | 0.113180091 |
| *PAK2* | Serine/threonine-protein kinase PAK 2 | http://www.genecards.org/cgi-bin/carddisp.pl?gene=PAK2 | 4.812467134 | 0.080330603 |
| *PALD1* | Paladin | http://www.genecards.org/cgi-bin/carddisp.pl?gene=PALD1 | 1.601064578 | 0.124980299 |
| *PAN2* | PAB-dependent poly(A)-specific ribonuclease subunit PAN2 | http://www.genecards.org/cgi-bin/carddisp.pl?gene=PAN2 | 11.04988412 | 0.188351745 |
| *PARK7* | Protein DJ-1 | http://www.genecards.org/cgi-bin/carddisp.pl?gene=PARK7 | 0.584211688 | 0.179650202 |
| *PARN* | Poly(A)-specific ribonuclease PARN | http://www.genecards.org/cgi-bin/carddisp.pl?gene=PARN | 1.716644239 | 0.066572625 |
| *PCBP1* | Poly(rC)-binding protein 1 | http://www.genecards.org/cgi-bin/carddisp.pl?gene=PCBP1 | 1.095343097 | 0.273880577 |
| *PCDH11X* | Protocadherin-11 X-linked | http://www.genecards.org/cgi-bin/carddisp.pl?gene=PCDH11X | 2.166859459 | 0.050450877 |
| *PCDH15* | Protocadherin-15 | http://www.genecards.org/cgi-bin/carddisp.pl?gene=PCDH15 | 1.524019845 | 0.13881869 |
| *PCSK5* | Proprotein convertase subtilisin/kexin type 5 | http://www.genecards.org/cgi-bin/carddisp.pl?gene=PCSK5 | 0.582345023 | 0.258567499 |
| *PDAP1* | 28 kDa heat- and acid-stable phosphoprotein | http://www.genecards.org/cgi-bin/carddisp.pl?gene=PDAP1 | 0.603723578 | 0.163138375 |
| *PDCD6IP* | Programmed cell death 6-interacting protein | http://www.genecards.org/cgi-bin/carddisp.pl?gene=PDCD6IP | 1.050443912 | 0.439744911 |
| *PDGFC* | Platelet-derived growth factor C | http://www.genecards.org/cgi-bin/carddisp.pl?gene=PDGFC | 0.844675618 | 0.272490018 |
| *PDIA3* | Protein disulfide-isomerase A3 | http://www.genecards.org/cgi-bin/carddisp.pl?gene=PDIA3 | 0.878413933 | 0.200872592 |
| *PDZRN3* | E3 ubiquitin-protein ligase PDZRN3 | http://www.genecards.org/cgi-bin/carddisp.pl?gene=PDZRN3 | 1.224045494 | 0.292227716 |
| *PEBP1* | Phosphatidylethanolamine-binding protein 1 | http://www.genecards.org/cgi-bin/carddisp.pl?gene=PEBP1 | 0.581870962 | 0.054454318 |
| *PFN1* | Profilin-1 | http://www.genecards.org/cgi-bin/carddisp.pl?gene=PFN1 | 0.915640743 | 0.319058632 |
| *PGAM1* | Phosphoglycerate mutase 1 | http://www.genecards.org/cgi-bin/carddisp.pl?gene=PGAM1 | 1.435029542 | 0.153701039 |
| *PGM1* | Phosphoglucomutase-1 | http://www.genecards.org/cgi-bin/carddisp.pl?gene=PGM1 | 1.39711871 | 0.166291704 |
| *PHLDB1* | Pleckstrin homology-like domain family B member 1 | http://www.genecards.org/cgi-bin/carddisp.pl?gene=PHLDB1 | 0.432581964 | 0.082881937 |
| *PICALM* | Phosphatidylinositol-binding clathrin assembly protein | http://www.genecards.org/cgi-bin/carddisp.pl?gene=PICALM | 0.98535705 | 0.476772781 |
| *PIP* | Prolactin-inducible protein | http://www.genecards.org/cgi-bin/carddisp.pl?gene=PIP | 0.482988586 | 0.186720079 |
| *PKP2* | Plakophilin-2 | http://www.genecards.org/cgi-bin/carddisp.pl?gene=PKP2 | 1.512578039 | 0.101185581 |
| *PLG* | Plasminogen | http://www.genecards.org/cgi-bin/carddisp.pl?gene=PLG | 1.796793296 | 0.218351723 |
| *PLS3* | Plastin-3 | http://www.genecards.org/cgi-bin/carddisp.pl?gene=PLS3 | 1.228526543 | 0.052576567 |
| *POLR2H* | DNA-directed RNA polymerases I, II, and III subunit RPABC3 | http://www.genecards.org/cgi-bin/carddisp.pl?gene=POLR2H | 9.629902735 | 0.171753261 |
| *POLR3A* | DNA-directed RNA polymerase III subunit RPC1 | http://www.genecards.org/cgi-bin/carddisp.pl?gene=POLR3A | 2.159255501 | 0.277468849 |
| *PPFIBP1* | Liprin-beta-1 | http://www.genecards.org/cgi-bin/carddisp.pl?gene=PPFIBP1 | 0.866625436 | 0.328773004 |
| *PPIAL4A* | Peptidyl-prolyl cis-trans isomerase A-like 4A | http://www.genecards.org/cgi-bin/carddisp.pl?gene=PPIAL4A | 0.676588032 | 0.169772509 |
| *PPP1R16A* | Protein phosphatase 1 regulatory subunit 16A | http://www.genecards.org/cgi-bin/carddisp.pl?gene=PPP1R16A | 11.63006513 | 0.080730977 |
| *PPP2R1A* | Serine/threonine-protein phosphatase 2A 65 kDa regulatory subunit A alpha isoform | http://www.genecards.org/cgi-bin/carddisp.pl?gene=PPP2R1A | 1.240317207 | 0.218490196 |
| *PPP2R3A* | Serine/threonine-protein phosphatase 2A regulatory subunit B'' subunit alpha | http://www.genecards.org/cgi-bin/carddisp.pl?gene=PPP2R3A | 1.104213088 | 0.12326887 |
| *PRAMEF7* | PRAME family member 7 | http://www.genecards.org/cgi-bin/carddisp.pl?gene=PRAMEF7 | 2.45739892 | 0.199893805 |
| *PRDX2* | Peroxiredoxin-2 | http://www.genecards.org/cgi-bin/carddisp.pl?gene=PRDX2 | 0.965778981 | 0.343195962 |
| *PRG4* | Proteoglycan 4 | http://www.genecards.org/cgi-bin/carddisp.pl?gene=PRG4 | 0.468787413 | 0.064940949 |
| *PRSS3* | Trypsin-3 | http://www.genecards.org/cgi-bin/carddisp.pl?gene=PRSS3 | 0.520841348 | 0.1628895 |
| *PTPN13* | Tyrosine-protein phosphatase non-receptor type 13 | http://www.genecards.org/cgi-bin/carddisp.pl?gene=PTPN13 | 2.68134747 | 0.145157075 |
| *PTPRA* | Receptor-type tyrosine-protein phosphatase alpha | http://www.genecards.org/cgi-bin/carddisp.pl?gene=PTPRA | only present in RPEp53-/- FA | 0.091305828 |
| *PTPRK* | Receptor-type tyrosine-protein phosphatase kappa | http://www.genecards.org/cgi-bin/carddisp.pl?gene=PTPRK | 0.716440821 | 0.084486634 |
| *PTTG1IP* | Pituitary tumor-transforming gene 1 protein-interacting protein | http://www.genecards.org/cgi-bin/carddisp.pl?gene=PTTG1IP | 1.835946306 | 0.213906004 |
| *PTX3* | Pentraxin-related protein PTX3 | http://www.genecards.org/cgi-bin/carddisp.pl?gene=PTX3 | 1.083167005 | 0.422637887 |
| *QTRT2* | Queuine tRNA-ribosyltransferase accessory subunit 2 | http://www.genecards.org/cgi-bin/carddisp.pl?gene=QTRT2 | 1.061613236 | 0.447361511 |
| *RAB13* | Ras-related protein Rab-13 | http://www.genecards.org/cgi-bin/carddisp.pl?gene=RAB13 | 0.857573671 | 0.409203522 |
| *RAB1A* | Ras-related protein Rab-1A | http://www.genecards.org/cgi-bin/carddisp.pl?gene=RAB1A | 1.14775921 | 0.365272876 |
| *RAB21* | Ras-related protein Rab-21 | http://www.genecards.org/cgi-bin/carddisp.pl?gene=RAB21 | 1.151047607 | 0.175118726 |
| *RAB23* | Ras-related protein Rab-23 | http://www.genecards.org/cgi-bin/carddisp.pl?gene=RAB23 | 0.729985838 | 0.08626848 |
| *RAB5C* | Ras-related protein Rab-5C | http://www.genecards.org/cgi-bin/carddisp.pl?gene=RAB5C | 2.524030804 | 0.085881891 |
| *RAB7A* | Ras-related protein Rab-7a | http://www.genecards.org/cgi-bin/carddisp.pl?gene=RAB7A | 1.30592485 | 0.063772254 |
| *RACK1* | Receptor of activated protein C kinase 1 | http://www.genecards.org/cgi-bin/carddisp.pl?gene=RACK1 | 0.995572746 | 0.471838931 |
| *RALA* | Ras-related protein Ral-A | http://www.genecards.org/cgi-bin/carddisp.pl?gene=RALA | 2.848690364 | 0.126820401 |
| *RALB* | Ras-related protein Ral-B | http://www.genecards.org/cgi-bin/carddisp.pl?gene=RALB | 0.073263396 | 0.074797575 |
| *RAP2B* | Ras-related protein Rap-2b | http://www.genecards.org/cgi-bin/carddisp.pl?gene=RAP2B | 0.875436652 | 0.2792999 |
| *RASA2* | Ras GTPase-activating protein 2 | http://www.genecards.org/cgi-bin/carddisp.pl?gene=RASA2 | 0.872707767 | 0.38466334 |
| *RCC1* | Regulator of chromosome condensation | http://www.genecards.org/cgi-bin/carddisp.pl?gene=RCC1 | 0.876279229 | 0.245403984 |
| *RFNG* | Beta-1,3-N-acetylglucosaminyltransferase radical fringe | http://www.genecards.org/cgi-bin/carddisp.pl?gene=RFNG | 0.957871997 | 0.441280916 |
| *RHOG* | Rho-related GTP-binding protein RhoG | http://www.genecards.org/cgi-bin/carddisp.pl?gene=RHOG | 1.433883021 | 0.105106285 |
| *RNH1* | Ribonuclease inhibitor | http://www.genecards.org/cgi-bin/carddisp.pl?gene=RNH1 | 0.9669438 | 0.402681406 |
| *RNPEPL1* | Aminopeptidase RNPEPL1 | http://www.genecards.org/cgi-bin/carddisp.pl?gene=RNPEPL1 | 7.32314299 | 0.067574575 |
| *RORB* | Nuclear receptor ROR-beta | http://www.genecards.org/cgi-bin/carddisp.pl?gene=RORB | 0.430541513 | 0.117405679 |
| *RPL26* | 60S ribosomal protein L26 | http://www.genecards.org/cgi-bin/carddisp.pl?gene=RPL26 | 0.122661577 | 0.061575253 |
| *RPL27* | 60S ribosomal protein L27 | http://www.genecards.org/cgi-bin/carddisp.pl?gene=RPL27 | 1.088266637 | 0.179453745 |
| *RPL28* | 60S ribosomal protein L28 | http://www.genecards.org/cgi-bin/carddisp.pl?gene=RPL28 | 0.780998729 | 0.303413138 |
| *RPL3* | 60S ribosomal protein L3 | http://www.genecards.org/cgi-bin/carddisp.pl?gene=RPL3 | 1.122484512 | 0.350464798 |
| *RPL9* | 60S ribosomal protein L9 | http://www.genecards.org/cgi-bin/carddisp.pl?gene=RPL9 | 6.628271945 | 0.101151184 |
| *RPLP1* | 60S acidic ribosomal protein P1 | http://www.genecards.org/cgi-bin/carddisp.pl?gene=RPLP1 | 3.401101372 | 0.075621498 |
| *RPN1* | Dolichyl-diphosphooligosaccharide--protein glycosyltransferase subunit 1 | http://www.genecards.org/cgi-bin/carddisp.pl?gene=RPN1 | 1.203792013 | 0.106710053 |
| *RPS11* | 40S ribosomal protein S11 | http://www.genecards.org/cgi-bin/carddisp.pl?gene=RPS11 | 1.154945353 | 0.319722096 |
| *RPS15A* | 40S ribosomal protein S15a | http://www.genecards.org/cgi-bin/carddisp.pl?gene=RPS15A | 4.041039763 | 0.057653821 |
| *RPS17* | 40S ribosomal protein S17 | http://www.genecards.org/cgi-bin/carddisp.pl?gene=RPS17 | 2.662794994 | 0.171587968 |
| *RPS2* | 40S ribosomal protein S2 | http://www.genecards.org/cgi-bin/carddisp.pl?gene=RPS2 | 0.546161255 | 0.166508593 |
| *RPS20* | 40S ribosomal protein S20 | http://www.genecards.org/cgi-bin/carddisp.pl?gene=RPS20 | 0.913120184 | 0.179453582 |
| *RPS21* | 40S ribosomal protein S21 | http://www.genecards.org/cgi-bin/carddisp.pl?gene=RPS21 | 1.110926242 | 0.403170293 |
| *RPS28* | 40S ribosomal protein S28 | http://www.genecards.org/cgi-bin/carddisp.pl?gene=RPS28 | 1.138920733 | 0.317819912 |
| *RPS3* | 40S ribosomal protein S3 | http://www.genecards.org/cgi-bin/carddisp.pl?gene=RPS3 | 0.757594258 | 0.188897881 |
| *RPS3A* | 40S ribosomal protein S3a | http://www.genecards.org/cgi-bin/carddisp.pl?gene=RPS3A | 4.27139224 | 0.062802658 |
| *RPS8* | 40S ribosomal protein S8 | http://www.genecards.org/cgi-bin/carddisp.pl?gene=RPS8 | 0.784798749 | 0.103743799 |
| *RPSA* | 40S ribosomal protein SA | http://www.genecards.org/cgi-bin/carddisp.pl?gene=RPSA | 1.127247107 | 0.112539668 |
| *RRAS* | Ras-related protein R-Ras | http://www.genecards.org/cgi-bin/carddisp.pl?gene=RRAS | 7.942843247 | 0.058292613 |
| *RRAS2* | Ras-related protein R-Ras2 | http://www.genecards.org/cgi-bin/carddisp.pl?gene=RRAS2 | 1.171315453 | 0.171215392 |
| *RSU1* | Ras suppressor protein 1 | http://www.genecards.org/cgi-bin/carddisp.pl?gene=RSU1 | 1.489044339 | 0.051921201 |
| *S100A10* | Protein S100-A10 | http://www.genecards.org/cgi-bin/carddisp.pl?gene=S100A10 | 1.158952278 | 0.050557575 |
| *S100A13* | Protein S100-A13 | http://www.genecards.org/cgi-bin/carddisp.pl?gene=S100A13 | 1.184621505 | 0.199279569 |
| *S100A6* | Protein S100-A6 | http://www.genecards.org/cgi-bin/carddisp.pl?gene=S100A6 | 1.134899512 | 0.136430478 |
| *S100A7* | Protein S100-A7 | http://www.genecards.org/cgi-bin/carddisp.pl?gene=S100A7 | 0.760931671 | 0.21406294 |
| *S100A9* | Protein S100-A9 | http://www.genecards.org/cgi-bin/carddisp.pl?gene=S100A9 | 0.729839713 | 0.192378844 |
| *SBNO2* | Protein strawberry notch homolog 2 | http://www.genecards.org/cgi-bin/carddisp.pl?gene=SBNO2 | 0.772926695 | 0.218282469 |
| *SCAMP1* | Secretory carrier-associated membrane protein 1 | http://www.genecards.org/cgi-bin/carddisp.pl?gene=SCAMP1 | 1.394855348 | 0.186172869 |
| *SCRIB* | Protein scribble homolog | http://www.genecards.org/cgi-bin/carddisp.pl?gene=SCRIB | 1.297869032 | 0.186583875 |
| *SDPR* | Serum deprivation-response protein | http://www.genecards.org/cgi-bin/carddisp.pl?gene=SDPR | 1.52153522 | 0.146457937 |
| *SEMA3A* | Semaphorin-3A | http://www.genecards.org/cgi-bin/carddisp.pl?gene=SEMA3A | 0.275960286 | 0.0625338 |
| *SEP10* | Septin-10 | http://www.genecards.org/cgi-bin/carddisp.pl?gene=SEP10 | 3.314422401 | 0.064391883 |
| *SEPT6* | Septin-6 | http://www.genecards.org/cgi-bin/carddisp.pl?gene=SEPT6 | 0.548614147 | 0.072885993 |
| *SERPINA1* | Alpha-1-antitrypsin | http://www.genecards.org/cgi-bin/carddisp.pl?gene=SERPINA1 | 0.72684577 | 0.171714444 |
| *SERPINF2* | Alpha-2-antiplasmin | http://www.genecards.org/cgi-bin/carddisp.pl?gene=SERPINF2 | 1.475820859 | 0.111067984 |
| *SERPINH1* | Serpin H1 | http://www.genecards.org/cgi-bin/carddisp.pl?gene=SERPINH1 | 1.04129542 | 0.409115493 |
| *SETD3* | Histone-lysine N-methyltransferase setd3 | http://www.genecards.org/cgi-bin/carddisp.pl?gene=SETD3 | 2.524672964 | 0.058187612 |
| *SF3B2* | Splicing factor 3B subunit 2 | http://www.genecards.org/cgi-bin/carddisp.pl?gene=SF3B2 | 0.004555029 | 0.053028913 |
| *SH3BP4* | SH3 domain-binding protein 4 | http://www.genecards.org/cgi-bin/carddisp.pl?gene=SH3BP4 | 0.98391885 | 0.471729358 |
| *SH3GL2* | Endophilin-A1 | http://www.genecards.org/cgi-bin/carddisp.pl?gene=SH3GL2 | 0.996249381 | 0.498133193 |
| *SHROOM3* | Protein Shroom3 | http://www.genecards.org/cgi-bin/carddisp.pl?gene=SHROOM3 | 0.470817676 | 0.072434049 |
| *SIX6OS1* | Protein SIX6OS1 | http://www.genecards.org/cgi-bin/carddisp.pl?gene=SIX6OS1 | 1.390938178 | 0.184167297 |
| *SLC12A1* | Solute carrier family 12 member 1 | http://www.genecards.org/cgi-bin/carddisp.pl?gene=SLC12A1 | 17.28204115 | 0.066322339 |
| *SLC16A3* | Monocarboxylate transporter 4 | http://www.genecards.org/cgi-bin/carddisp.pl?gene=SLC16A3 | 0.95398841 | 0.396859281 |
| *SLC38A2* | Sodium-coupled neutral amino acid transporter 2 | http://www.genecards.org/cgi-bin/carddisp.pl?gene=SLC38A2 | 0.998327959 | 0.496652322 |
| *SLC39A10* | Zinc transporter ZIP10 | http://www.genecards.org/cgi-bin/carddisp.pl?gene=SLC39A10 | 0.662383978 | 0.154507516 |
| *SLC7A5* | Large neutral amino acids transporter small subunit 1 | http://www.genecards.org/cgi-bin/carddisp.pl?gene=SLC7A5 | 1.029007338 | 0.425913063 |
| *SLC9A2* | Sodium/hydrogen exchanger 2 | http://www.genecards.org/cgi-bin/carddisp.pl?gene=SLC9A2 | 1.185186745 | 0.202247877 |
| *SLIT1* | Slit homolog 1 protein | http://www.genecards.org/cgi-bin/carddisp.pl?gene=SLIT1 | 0.060804323 | 0.188010904 |
| *SMARCA5* | SWI/SNF-related matrix-associated actin-dependent regulator of chromatin subfamily A member 5 | http://www.genecards.org/cgi-bin/carddisp.pl?gene=SMARCA5 | 1.664236971 | 0.141055491 |
| *SNRPD2* | Small nuclear ribonucleoprotein Sm D2 | http://www.genecards.org/cgi-bin/carddisp.pl?gene=SNRPD2 | 0.393202327 | 0.119253644 |
| *SNTB2* | Beta-2-syntrophin | http://www.genecards.org/cgi-bin/carddisp.pl?gene=SNTB2 | 1.3826135 | 0.05705119 |
| *SORBS2* | Sorbin and SH3 domain-containing protein 2 | http://www.genecards.org/cgi-bin/carddisp.pl?gene=SORBS2 | 2.034587044 | 0.185563479 |
| *SPECC1* | Cytospin-B | http://www.genecards.org/cgi-bin/carddisp.pl?gene=SPECC1 | 24.64827776 | 0.156726003 |
| *SPEF2* | Sperm flagellar protein 2 | http://www.genecards.org/cgi-bin/carddisp.pl?gene=SPEF2 | 1.434661929 | 0.18636786 |
| *SPTAN1* | Spectrin alpha chain, non-erythrocytic 1 | http://www.genecards.org/cgi-bin/carddisp.pl?gene=SPTAN1 | 1.041347633 | 0.452275728 |
| *SPTB* | Spectrin beta chain, erythrocytic | http://www.genecards.org/cgi-bin/carddisp.pl?gene=SPTB | 2.80572661 | 0.15590641 |
| *SRP9* | Signal recognition particle 9 kDa protein | http://www.genecards.org/cgi-bin/carddisp.pl?gene=SRP9 | 0.758475653 | 0.096578692 |
| *SRPX* | Sushi repeat-containing protein SRPX | http://www.genecards.org/cgi-bin/carddisp.pl?gene=SRPX | 0.976061748 | 0.440005687 |
| *SSFA2* | Sperm-specific antigen 2 | http://www.genecards.org/cgi-bin/carddisp.pl?gene=SSFA2 | 0.709254094 | 0.065303541 |
| *STMN1* | Stathmin | http://www.genecards.org/cgi-bin/carddisp.pl?gene=STMN1 | 0.928785983 | 0.321556717 |
| *STRAP* | Serine-threonine kinase receptor-associated protein | http://www.genecards.org/cgi-bin/carddisp.pl?gene=STRAP | 1.310799346 | 0.265555474 |
| *STX12* | Syntaxin-12 | http://www.genecards.org/cgi-bin/carddisp.pl?gene=STX12 | 0.50068747 | 0.05771716 |
| *SVIP* | Small VCP/p97-interacting protein | http://www.genecards.org/cgi-bin/carddisp.pl?gene=SVIP | 0.07742318 | 0.086348293 |
| *SYCP1* | Synaptonemal complex protein 1 | http://www.genecards.org/cgi-bin/carddisp.pl?gene=SYCP1 | 0.941170628 | 0.394844803 |
| *SYK* | Tyrosine-protein kinase SYK | http://www.genecards.org/cgi-bin/carddisp.pl?gene=SYK | 1.022567429 | 0.467028916 |
| *SYNCRIP* | Heterogeneous nuclear ribonucleoprotein Q | http://www.genecards.org/cgi-bin/carddisp.pl?gene=SYNCRIP | 8.977925448 | 0.155327537 |
| *SYNE1* | Nesprin-1 | http://www.genecards.org/cgi-bin/carddisp.pl?gene=SYNE1 | 1.080114937 | 0.374973047 |
| *SYTL2* | Synaptotagmin-like protein 2 | http://www.genecards.org/cgi-bin/carddisp.pl?gene=SYTL2 | 1.229388981 | 0.301751396 |
| *TBC1D24* | TBC1 domain family member 24 | http://www.genecards.org/cgi-bin/carddisp.pl?gene=TBC1D24 | 1.14107317 | 0.312182966 |
| *TBC1D8* | TBC1 domain family member 8 | http://www.genecards.org/cgi-bin/carddisp.pl?gene=TBC1D8 | 0.752809476 | 0.060672496 |
| *TBC1D8B* | TBC1 domain family member 8B | http://www.genecards.org/cgi-bin/carddisp.pl?gene=TBC1D8B | 0.57615568 | 0.093417871 |
| *TERT* | Telomerase reverse transcriptase | http://www.genecards.org/cgi-bin/carddisp.pl?gene=TERT | 0.814574388 | 0.280197519 |
| *TGFBI* | Transforming growth factor-beta-induced protein ig-h3 | http://www.genecards.org/cgi-bin/carddisp.pl?gene=TGFBI | 1.047109898 | 0.272042588 |
| *THADA* | Thyroid adenoma-associated protein | http://www.genecards.org/cgi-bin/carddisp.pl?gene=THADA | 0.460968268 | 0.149220482 |
| *THSD4* | Thrombospondin type-1 domain-containing protein 4 | http://www.genecards.org/cgi-bin/carddisp.pl?gene=THSD4 | 1.051696451 | 0.200270756 |
| *TIPARP* | TCDD-inducible poly [ADP-ribose] polymerase | http://www.genecards.org/cgi-bin/carddisp.pl?gene=TIPARP | 0.332519747 | 0.056836099 |
| *TKFC* | Triokinase/FMN cyclase | http://www.genecards.org/cgi-bin/carddisp.pl?gene=TKFC | 2.752099843 | 0.071105617 |
| *TKT* | Transketolase | http://www.genecards.org/cgi-bin/carddisp.pl?gene=TKT | 0.771611671 | 0.056599555 |
| *TLDC1* | TLD domain-containing protein 1 | http://www.genecards.org/cgi-bin/carddisp.pl?gene=TLDC1 | 307.7901649 | 0.189951934 |
| *TLL2* | Tolloid-like protein 2 | http://www.genecards.org/cgi-bin/carddisp.pl?gene=TLL2 | 0.388743423 | 0.203892765 |
| *TMBIM1* | Protein lifeguard 3 | http://www.genecards.org/cgi-bin/carddisp.pl?gene=TMBIM1 | 0.801498371 | 0.156015257 |
| *TMC3* | Transmembrane channel-like protein 3 | http://www.genecards.org/cgi-bin/carddisp.pl?gene=TMC3 | 8.246849262 | 0.173042218 |
| *TMEM109* | Transmembrane protein 109 | http://www.genecards.org/cgi-bin/carddisp.pl?gene=TMEM109 | 1.386771363 | 0.192704722 |
| *TMEM30A* | Cell cycle control protein 50A | http://www.genecards.org/cgi-bin/carddisp.pl?gene=TMEM30A | 1.687674724 | 0.131776664 |
| *TNC* | Tenascin | http://www.genecards.org/cgi-bin/carddisp.pl?gene=TNC | 0.873468441 | 0.077774804 |
| *TNFRSF12A* | Tumor necrosis factor receptor superfamily member 12A | http://www.genecards.org/cgi-bin/carddisp.pl?gene=TNFRSF12A | 0.679856648 | 0.142234048 |
| *TNKS1BP1* | 182 kDa tankyrase-1-binding protein | http://www.genecards.org/cgi-bin/carddisp.pl?gene=TNKS1BP1 | 0.809163741 | 0.085335502 |
| *TNS4* | Tensin-4 | http://www.genecards.org/cgi-bin/carddisp.pl?gene=TNS4 | 0.824862168 | 0.295380782 |
| *TPI1* | Triosephosphate isomerase | http://www.genecards.org/cgi-bin/carddisp.pl?gene=TPI1 | 1.423951124 | 0.053497148 |
| *TPM2* | Tropomyosin beta chain | http://www.genecards.org/cgi-bin/carddisp.pl?gene=TPM2 | 1.772038048 | 0.077446339 |
| *TPT1* | Translationally-controlled tumor protein | http://www.genecards.org/cgi-bin/carddisp.pl?gene=TPT1 | 0.969227412 | 0.460106469 |
| *TRAK1* | Trafficking kinesin-binding protein 1 | http://www.genecards.org/cgi-bin/carddisp.pl?gene=TRAK1 | 3.332076714 | 0.195253881 |
| *TRAP1* | Heat shock protein 75 kDa, mitochondrial | http://www.genecards.org/cgi-bin/carddisp.pl?gene=TRAP1 | 1.418186838 | 0.09123469 |
| *TRHDE* | Thyrotropin-releasing hormone-degrading ectoenzyme | http://www.genecards.org/cgi-bin/carddisp.pl?gene=TRHDE | 0.781156344 | 0.19294898 |
| *TRIM58* | E3 ubiquitin-protein ligase TRIM58 | http://www.genecards.org/cgi-bin/carddisp.pl?gene=TRIM58 | 4.691117162 | 0.143770889 |
| *TRMT10B* | tRNA methyltransferase 10 homolog B | http://www.genecards.org/cgi-bin/carddisp.pl?gene=TRMT10B | 0.719805995 | 0.306964549 |
| *TRPC7* | Short transient receptor potential channel 7 | http://www.genecards.org/cgi-bin/carddisp.pl?gene=TRPC7 | 2.041987244 | 0.121805013 |
| *TSNAXIP1* | Translin-associated factor X-interacting protein 1 | http://www.genecards.org/cgi-bin/carddisp.pl?gene=TSNAXIP1 | 0.517666477 | 0.159296818 |
| *TSPOAP1* | Peripheral-type benzodiazepine receptor-associated protein 1 | http://www.genecards.org/cgi-bin/carddisp.pl?gene=TSPOAP1 | 2.705596112 | 0.140422423 |
| *TTBK1* | Tau-tubulin kinase 1 | http://www.genecards.org/cgi-bin/carddisp.pl?gene=TTBK1 | 1.150894873 | 0.36634045 |
| *TUBA1A* | Tubulin alpha-1A chain | http://www.genecards.org/cgi-bin/carddisp.pl?gene=TUBA1A | 0.343839541 | 0.056866548 |
| *TUBB4B* | Tubulin beta-4B chain | http://www.genecards.org/cgi-bin/carddisp.pl?gene=TUBB4B | 1.045434273 | 0.368740784 |
| *UBA1* | Ubiquitin-like modifier-activating enzyme 1 | http://www.genecards.org/cgi-bin/carddisp.pl?gene=UBA1 | 2.101516785 | 0.081230857 |
| *UBAP2* | Ubiquitin-associated protein 2 | http://www.genecards.org/cgi-bin/carddisp.pl?gene=UBAP2 | 0.637050002 | 0.119105671 |
| *UBAP2L* | Ubiquitin-associated protein 2-like | http://www.genecards.org/cgi-bin/carddisp.pl?gene=UBAP2L | 1.118444827 | 0.412123243 |
| *UGDH* | UDP-glucose 6-dehydrogenase | http://www.genecards.org/cgi-bin/carddisp.pl?gene=UGDH | 0.700187868 | 0.05734242 |
| *UHRF1* | E3 ubiquitin-protein ligase UHRF1 | http://www.genecards.org/cgi-bin/carddisp.pl?gene=UHRF1 | 0.711700919 | 0.187736463 |
| *VAMP3* | Vesicle-associated membrane protein 3 | http://www.genecards.org/cgi-bin/carddisp.pl?gene=VAMP3 | 1.467120835 | 0.0980829 |
| *VAT1* | Synaptic vesicle membrane protein VAT-1 homolog | http://www.genecards.org/cgi-bin/carddisp.pl?gene=VAT1 | 76.5768823 | 0.144483616 |
| *VAV1* | Proto-oncogene vav | http://www.genecards.org/cgi-bin/carddisp.pl?gene=VAV1 | 0.450328661 | 0.134218075 |
| *VCAN* | Versican core protein | http://www.genecards.org/cgi-bin/carddisp.pl?gene=VCAN | 1.048543207 | 0.344667432 |
| *VCP* | Transitional endoplasmic reticulum ATPase | http://www.genecards.org/cgi-bin/carddisp.pl?gene=VCP | 1.08323226 | 0.276534329 |
| *VDAC2* | Voltage-dependent anion-selective channel protein 2 | http://www.genecards.org/cgi-bin/carddisp.pl?gene=VDAC2 | 1.116039757 | 0.344899033 |
| *VTA1* | Vacuolar protein sorting-associated protein VTA1 homolog | http://www.genecards.org/cgi-bin/carddisp.pl?gene=VTA1 | 1.420737476 | 0.187659592 |
| *WASF2* | Wiskott-Aldrich syndrome protein family member 2 | http://www.genecards.org/cgi-bin/carddisp.pl?gene=WASF2 | 0.630711781 | 0.155348528 |
| *WNT5A* | Protein Wnt-5a | http://www.genecards.org/cgi-bin/carddisp.pl?gene=WNT5A | 1.231460744 | 0.07518431 |
| *WNT5B* | Protein Wnt-5b | http://www.genecards.org/cgi-bin/carddisp.pl?gene=WNT5B | 0.753130016 | 0.06806293 |
| *YES1* | Tyrosine-protein kinase Yes | http://www.genecards.org/cgi-bin/carddisp.pl?gene=YES1 | 1.386942897 | 0.283975316 |
| *YWHAG* | 14-3-3 protein gamma | http://www.genecards.org/cgi-bin/carddisp.pl?gene=YWHAG | 5.656419914 | 0.05599464 |
| *YWHAH* | 14-3-3 protein eta | http://www.genecards.org/cgi-bin/carddisp.pl?gene=YWHAH | 0.863989157 | 0.374879445 |
| *YWHAZ* | 14-3-3 protein zeta/delta | http://www.genecards.org/cgi-bin/carddisp.pl?gene=YWHAZ | 1.122749224 | 0.155675882 |
| *ZMYND15* | Zinc finger MYND domain-containing protein 15 | http://www.genecards.org/cgi-bin/carddisp.pl?gene=ZMYND15 | 1.203492235 | 0.178021933 |
| *ZNF248* | Zinc finger protein 248 | http://www.genecards.org/cgi-bin/carddisp.pl?gene=ZNF248 | 3.293824525 | 0.054372132 |
| *ZNF529* | Zinc finger protein 529 | http://www.genecards.org/cgi-bin/carddisp.pl?gene=ZNF529 | 0.469050535 | 0.051856497 |
| *ZYG11B* | Protein zyg-11 homolog B | http://www.genecards.org/cgi-bin/carddisp.pl?gene=ZYG11B | 5.655411685 | 0.115655769 |
