# Supplementary material for: Centrosome guides spatial activation of Rac to control cell polarization and directed cell migration
Source: Life Sci Alliance. 2019 Feb 8;2(1):e201800135. doi: 10.26508/lsa.201800135 (PMC6369537; doi:10.26508/lsa.201800135)
Supplement: Supplementary file 9 [file LSA-2018-00135_TableS6.docx]

**Table S6. Effects of GEFs, GAPs and GDIs on centrosome-regulated Rac1 activation.**

| **NO.** | **shRNA target gene** | **shRNA target protein** | **Ratio in Rac1 activity assay^a^** | **Effect^b^** | **Ratio in Rac1 activity assay^c^** | **Effect^b^** |
| --- | --- | --- | --- | --- | --- | --- |
| 1 | *-* | - | 2.94 | ↑ | 2.68 | ↑ |
| 2 | *GIT1* | ARF GTPase-activating protein GIT1 | 1.6 | ↑ | 1.6 | ↑ |
| 3 | *GIT2* | ARF GTPase-activating protein GIT2 | 1.39 | ↑ | 1.76 | ↑ |
| 4 | *TIAM1* | T Cell Lymphoma Invasion And Metastasis 1 (TIAM1) | 6.85 | ↑ | 3.75 | ↑ |
| 5 | *ARHGEF7* | β-PIX | 1.48 | ↑ | 1.38 | ↑ |
| 6 | *ARHGDIA* | Rho GDP-dissociation inhibitor alpha | 1.37 | ↑ | 1.58 | ↑ |
| 7 | *GDI2* | GDP dissociation inhibitor 2 (GDIB) | 1.66 | ↑ | 1.45 | ↑ |

^a^ Integrated band intensity (in Western blot) intensity ratio of RPEp53^-/-^SAS6^-/-^/RPEp53^-/-^. 1.25-fold was set as threshold of substantial change.

^b^ Symbols presented the effect from ratio. ↑ indicated that the Rac1 activity was increased in acentrosomal cells; − indicated that centrosome disruption did not alter Rac1 activity; ↓ indicated that the Rac1 activity was decreased in acentrosomal cells.

^c^ Integrated band intensity (in Western blot) intensity ratio of RPEp53^-/-^STIL^-/-^/RPEp53^-/-^. 1.25-fold was set as threshold of substantial change.
